# Upscaling Participatory Action and Videos for Agriculture and Nutrition (UPAVAN) trial comparing three variants of a nutrition-sensitive agricultural extension intervention to improve maternal and child nutritional outcomes in rural Odisha, India: study protocol for a cluster randomised controlled trial

**DOI:** 10.1186/s13063-018-2521-y

**Published:** 2018-03-09

**Authors:** Suneetha Kadiyala, Audrey Prost, Helen Harris-Fry, Meghan O’Hearn, Ronali Pradhan, Shibananth Pradhan, Naba Kishore Mishra, Suchitra Rath, Nirmala Nair, Shibanand Rath, Prasantha Tripathy, Sneha Krishnan, Peggy Koniz-Booher, Heather Danton, Diana Elbourne, Joanna Sturgess, Emma Beaumont, Hassan Haghparast-Bidgoli, Jolene Skordis-Worrall, Satyanarayan Mohanty, Avinash Upadhay, Elizabeth Allen

**Affiliations:** 1 London School of Hygiene & Tropical Medicine, Keppel Street, London, WC1E 7HT UK; 2 University College London, Institute for Global Health, 30 Guilford Street, London, WC1N 1EH UK; 3 Digital Green, S-26 to 28, 3rd Floor, Green Park Extension Market, New Delhi, 110016 India; 4 VARRAT (Voluntary Association for Rural Reconstruction and Appropriate Technology), Boulakani Baradang, Mahakalpara Kendrapad, Keonjhar, 754224 Odisha India; 5 Ekjut, 556 B, Ward No. 17, Potka, Chakradharpur, 833102 Jharkhand India; 6 Strengthening Partnerships, Results, and Innovations in Nutrition Globally, JSI Research & Training Institute, Inc., 1616 Fort Myer Drive, 16th Floor, Arlington, 22209 VA USA; 7 Development Corner Consulting Pvt. Ltd. (DCOR), 131(P), Punjabi Chhak, Satyanagar, Near Hotel Sungreen, Bhubaneshwar, 751007 India

**Keywords:** Maternal nutrition, Child nutrition, Dietary diversity, Agricultural extension, Women’s groups, Participatory Learning and Action, Digital technology, Videos, India, Trial

## Abstract

**Background:**

Maternal and child undernutrition have adverse consequences for pregnancy outcomes and child morbidity and mortality, and they are associated with low educational attainment, economic productivity as an adult, and human wellbeing. ‘Nutrition-sensitive’ agriculture programs could tackle the underlying causes of undernutrition.

**Methods/design:**

This study is a four-arm cluster randomised controlled trial in Odisha, India. Interventions are as follows: (1) an agricultural extension platform of women’s groups viewing and discussing videos on nutrition-sensitive agriculture (NSA) practices, and follow-up visits to women at home to encourage the adoption of new practices shown in the videos; (2) women’s groups viewing and discussing videos on NSA and nutrition-specific practices, with follow-up visits; and (3) women’s groups viewing and discussing videos on NSA and nutrition-specific practices combined with a cycle of Participatory Learning and Action meetings, with follow-up visits. All arms, including the control, receive basic nutrition training from government community frontline workers. Primary outcomes, assessed at baseline and 32 months after the start of the interventions, are (1) percentage of children aged 6–23 months consuming ≥ 4 out of 7 food groups per day and (2) mean body mass index (BMI) (kg/m^2^) of non-pregnant, non-postpartum (gave birth > 42 days ago) mothers or female primary caregivers of children aged 0–23 months. Secondary outcomes are percentage of mothers consuming ≥ 5 out of 10 food groups per day and percentage of children’s weight-for-height *z*-score < -2 standard deviations (SD).

The unit of randomisation is a cluster, defined as one or more villages with a combined minimum population of 800 residents. There are 37 clusters per arm, and outcomes will be assessed in an average of 32 eligible households per cluster. For randomisation, clusters are stratified by distance to nearest town (< 10 km or ≥ 10 km), and low (< 30%), medium (30–70%), or high (> 70%) proportion of Scheduled Tribe or Scheduled Caste (disadvantaged) households. A process evaluation will assess the quality of implementation and mechanisms behind the intervention effects. A cost-consequence analysis will compare incremental costs and outcomes of the interventions.

**Discussion:**

This trial will contribute evidence on the impacts of NSA extension through participatory, low-cost, video-based approaches on maternal and child nutrition and on whether integration with nutrition-specific goals and enhanced participatory approaches can increase these impacts.

**Trial registration:**

ISRCTN , ISRCTN65922679. Registered on 21 December 2016.

**Electronic supplementary material:**

The online version of this article (10.1186/s13063-018-2521-y) contains supplementary material, which is available to authorized users.

## Background

Maternal and child undernutrition are among the world’s most serious health, economic, and human development challenges. Undernutrition contributes to around 3.1 million (45%) child deaths annually [1]. In South Asia, more than a third of children are stunted and a third of women are underweight [2]. Maternal and child undernutrition have adverse consequences for pregnancy outcomes, children’s morbidity and mortality, their physical and cognitive development, and the incidence of chronic diseases in adulthood [3]. The impacts of undernutrition also extend beyond health, with negative consequences for educational attainment, economic productivity as adults, and human wellbeing [4].

India has made progress in reducing undernutrition, but the prevalence of child undernutrition remains strikingly high: 38% of children under 5 years of age are stunted (height-for-age z-score < -2 standard deviations, SD), 21% are wasted (weight-for-height *z*-score < -2 SD), and 58% are anaemic [5]. In addition, almost a quarter of Indian women aged 15–49 are underweight and more than half are anaemic [6].

### Making agriculture work for nutrition

Estimates suggest that implementing ten nutrition-specific interventions, addressing the immediate determinants of undernutrition, at 90% coverage could reduce deaths by 15% and stunting prevalence by 20% among children under 5 years old [3]. Accelerating reductions in undernutrition will entail coupling nutrition-specific interventions with nutrition-sensitive programs that address the underlying causes of undernutrition in other sectors, such as agriculture, education, and social welfare [7].

‘Making agriculture work for nutrition*’* is now a policy priority. The question is: How can it be done?

Agriculture can affect maternal and child nutrition through several interrelated pathways [8]. For example, agriculture is a source of income and food, and it has effects on food prices [9]. Women’s participation and decision-making in agriculture also affect their, and their children’s, nutritional outcomes [7, 9]. Between 2001 and 2013, more than a dozen literature reviews have sought to understand whether agricultural programs improve nutrition outcomes [7, 10–15]. These reviews highlighted several critical methodological and evidence gaps, calling for more rigorous, theory-driven evaluations of nutrition-sensitive agriculture (NSA) intervention impacts on nutrition outcomes. The findings of the most recent systematic review [16] testify to a swift response to this call in recent years. Their review finds that NSA interventions show positive impacts on maternal dietary diversity, micronutrient intakes, child wasting and maternal underweight, and reduction in anaemia among mothers and children, but not child stunting. However, there remains a paucity of studies assessing the impact on maternal nutrition outcomes (only two studies in the review assessed maternal anthropometry [17, 18]) and the cost-effectiveness of NSA interventions.

### Study justification

The review of Ruel et al. [16] shows encouraging progress, with the addition of recent studies on nutritional impacts of diverse agriculture interventions such as biofortification, homestead food production, livestock, nutrition-sensitive value chains, and irrigation. This review and several recent global reports [19, 20] highlight the critical need to continue to develop, improve, and assess innovative NSA approaches in this Decade of Action on Nutrition.

Agricultural extension services aim to foster smallholder farmers’ access to technical knowledge and information, improve their skills through training and demonstration, and facilitate access to credit and markets [21–23]. Nowadays, these services are also expected to play a broader role in improving rural livelihoods and food security and promoting farming techniques that are not harmful to the environment; these services could also provide a platform to improve nutritional outcomes [22]. Most agricultural extension services involve a worker going from door to door in villages and interacting with a select number of people, usually men with larger farms [22, 23]. Studies have shown that many farmers have limited access to extension services and, even when they do, farmers may not adopt extension workers’ advice if these agents do not possess location-specific, relevant knowledge [24]. Extension workers visit infrequently or erratically, and their information does not reach farmers with the lowest yields, many of whom are women and poor [24, 25]. Consequently, agricultural extension programs have been inefficient with mixed impacts on agricultural productivity [23, 26]. Recognising this, agricultural extension systems in India and worldwide are adopting pluralistic, decentralised, participatory, gender-sensitive, demand-driven approaches to improve coverage and relevance [27, 28].

A promising innovation is the use of digital platforms that can strengthen the delivery and uptake of extension services in agriculture, even in the poorest settings [29–32]. One such approach features low-cost participatory videos with women’s groups and farmers’ groups. In a pilot trial involving 16 villages, this approach increased the adoption of promoted agricultural practices sevenfold compared with the traditional extension approach [33]. On a cost-per-adoption basis, the method was ten times more effective per dollar spent than a classical extension system [33]. A trial in the Indian state of Bihar found that this intervention resulted in a 21% increase in agricultural productivity compared with the control areas [34]. By integrating nutrition-sensitive video content to improve yields of nutrient-rich foods, increase incomes, reduce women’s workloads, and promote more participation of women in agricultural decision-making, this video approach could leverage these agricultural improvements to improve dietary quality and nutritional status. However, to our knowledge, no studies have examined whether this approach, if made nutrition-sensitive, can improve nutrition outcomes for mothers and children.

As with agriculture, experimentation with digital platforms for health- and nutrition-related behaviour change has gained momentum worldwide. Published literature on the impact of digital interventions to improve nutrition is only just emerging, and the overall quality of evidence remains low [35]. Participatory interventions integrating mobile phone messages have been shown to improve infant and young child feeding practices in two trials in Nigeria and China [36, 37]. Building on this success and on the success of low-cost videos for agriculture, videos with nutrition-specific messages could provide viewers with knowledge and opportunities to adopt new nutrition practices. However, we did not identify any studies that evaluated the impact of any other digital technologies, such as videos shown in women’s groups, on undernutrition outcomes.

Another important development relevant to efforts to improve agricultural productivity, health, and nutrition is the spread of participatory women’s groups. Participatory interventions with women’s groups using a Participatory Learning and Action (PLA) approach have shown large positive impacts on essential newborn care practices and have reduced neonatal deaths [38]. These interventions have also shown large effects on women’s nutritional knowledge and dietary diversity [39, 40], but limited impacts on maternal and child nutritional status [40]. In PLA groups, women acquire new nutrition knowledge, receive social support from peers and the community, and exercise both problem-solving and decision-making power [41]. Coupled with a nutrition-sensitive, video-based agricultural extension approach, all of these PLA processes could enable women and their families to further capitalise on the nutrition benefits of an NSA intervention.

The challenge of making agriculture work for nutrition is twofold: it involves (1) strengthening agriculture programs in a way that is feasible and can therefore be effectively implemented at scale, and (2) making these programs ‘nutrition-sensitive’. The UPAVAN trial (Upscaling Participatory Action and Videos for Agriculture and Nutrition) aims to address this dual challenge by testing the nutritional and agricultural effects of (1) a participatory, NSA extension platform with women’s groups viewing videos and follow-up visits and (2) two variants of this intervention, integrating videos on nutrition-specific behaviour change and integrating the PLA methodology. Rigorous assessment of these interventions will determine the effects and cost-effectiveness of NSA extension through participatory, low-cost, video-based approaches on maternal and child nutrition and whether integration of nutrition-specific goals and enhanced participatory approaches can increase these effects.

## Methods/design

### Study aim

This study aims to estimate the nutritional and agricultural impacts and cost-effectiveness of (1) an agricultural extension platform of women’s groups viewing and discussing videos on NSA practices, with visits at women’s homes or farms to follow up on adoption of new practices shown in the videos (AGRI), (2) women’s groups viewing and discussing videos on NSA and nutrition-specific practices, also with follow-up visits (AGRI-NUT), and (3) women’s groups viewing and discussing videos on NSA combined with a PLA approach of meetings and nutrition-specific videos, with follow-up visits (AGRI-NUT+PLA). These three interventions will be compared to a control arm that does not receive these interventions, but might receive, along with the treatment arms, standard agriculture-, health- and nutrition-related services provided by the government or other organisations. Government frontline health and nutrition workers (*Anganwadi* workers and Accredited Social Health Activists) in all arms, including the control arm, will receive 2 days of training on nutrition.

### Study objectives

The objectives of the study are to:

1. Evaluate the impact of the AGRI, AGRI-NUT, and AGRI-NUT+PLA interventions, each compared to the control, on maternal and child nutrition outcomes
2. Evaluate the impact of the interventions, each compared to the control, on agriculture-related outcomes including production diversity and total and net value of agriculture production
3. Test the pathways through which the proposed interventions may improve maternal and child nutrition outcomes. The specific hypothesised pathways of interest are:
  Interventions → agricultural production → nutrition outcomes
  Interventions → household income → nutrition outcomes
  Interventions → women’s workload → nutrition outcomes
  Interventions → women’s decision-making power → nutrition outcomes
4. Assess the incremental cost-consequence of the three intervention approaches, each compared to the control
5. Explain the interventions’ effects by assessing the fidelity and quality of the interventions’ implementation, clarifying mechanisms behind the interventions’ effects using a theory of change, and identifying contextual factors associated with variation in effects on the primary and secondary outcomes

### Study setting

**Fig. 1:**
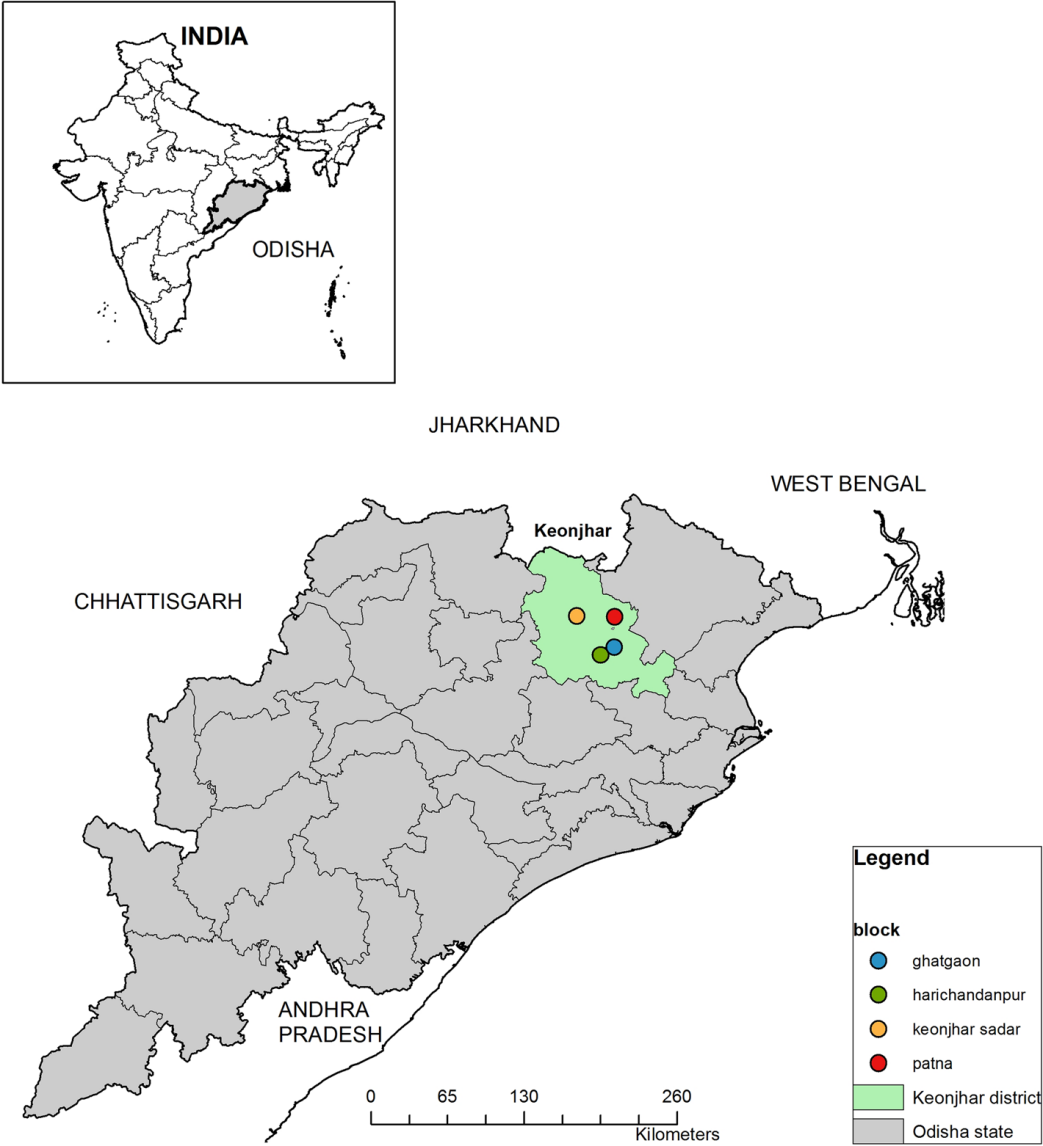
Map of UPAVAN study site

The study is located in four administrative blocks (Patna, Keonjhar, Harichandanpur, and Ghatgaon blocks) in Keonjhar District, Odisha, eastern India, as shown in Fig. 1.

Keonjhar has a population of around 1.8 million which is predominantly rural (86% of the population) and agrarian [42]. Scheduled Castes and Scheduled Tribes, the historically disadvantaged groups in India, comprise 57% of Keonjhar’s population (12% and 45% respectively) [42]. About 30% of women in the district are underweight and 40% are anaemic [6]. The prevalences of child (< 5 years of age) stunting and wasting are 45% and 19% respectively, and less than 10% of children 6–23 months of age are fed the minimum acceptable diet [6]. Around three quarters of people in rural areas earn their livelihood through traditional agriculture, selling wood, and collecting non-timber forest products [43].

The interventions are implemented and evaluated by a consortium of seven partners. (1) Digital Green, a global development organisation working in digital participatory extension services, coordinates the implementation of interventions. (2) The Voluntary Association for Rural Reconstruction and Appropriate Technology (VARRAT), an Odisha-based non-governmental organisation, is responsible for implementing the interventions in Keonjhar. (3) John Snow Inc. (JSI) Research and Training Institute, through its Strengthening Partnerships, Results, and Innovations in Nutrition Globally project, provides technical assistance for the development of interventions, and (4) Ekjut, an Indian non-governmental organisation, provides technical assistance on PLA in the AGRI-NUT+PLA arm. (5) The London School of Hygiene & Tropical Medicine (LSHTM) leads all research activities, in collaboration with (6) University College London’s Institute for Global Health and (7) Development Corner Consulting Pvt. Ltd.

### Study design

The study is a four-arm, cluster randomised controlled trial with 148 clusters randomly allocated to the four trial arms to give 37 clusters per arm (allocation ratio 1:1:1:1). The trial will run for a total of 53 months, from December 2015 to May 2020, including intervention development, setup, baseline and endline surveys, and intervention implementation for 32 months (from March 2017 to October 2019). The impact of the interventions will be assessed by repeat cross-sectional surveys of eligible households at baseline (November 2016 to January 2017) and endline (November 2019 to January 2020).

### Selection of clusters and unit of randomisation

Randomisation was undertaken remotely by the Clinical Trials Unit at the LSHTM. The unit of randomisation is a cluster, defined as one or two villages and surrounding hamlets to give a minimum population size of 800 per cluster. Clusters were selected from four administrative blocks (Patna, Keonjhar, Harichandanpur, and Ghatgaon blocks) in Keonjhar District (Fig. 1). One hundred forty-eight clusters were randomly allocated to four trial arms, stratified by distance to nearest town (< 10 km or ≥ 10 km) and low (< 30%), medium (30–70%), or high (> 70%) proportion of Scheduled Tribe or Scheduled Caste households in the cluster, giving six strata in total. The randomisation schedule was drawn up once cluster-level consent was obtained and the clusters completed the baseline survey, thus guarding against selection biases at entry of clusters to the trial. Randomisation occurred sequentially in two batches (December 2016 and January 2017) due to the time it took to complete the baseline survey.

### Trial participants

The interventions are delivered at the cluster level, and all women in the intervention clusters are eligible to participate (‘intervention participants’). The trial will evaluate impacts on outcomes in ‘trial participants’, who are children 0–23 months of age, their mothers or female primary caregivers where the mother is absent (aged 15 to 49 years), and their households. Trial outcomes are measured using baseline and endline repeat cross-sectional surveys on an average of 32 randomly selected eligible households per cluster. If more than one child aged 0–23 months is present in a randomly selected household, we will randomly select one of them for inclusion.

Exclusion criteria for trial participants are any disability impairing participation in the survey for mothers; any disability affecting weight, standing height, or recumbent length for children; and household members who are not permanent residents. Residents should have lived in the household at least half of the time during the past 12 months (e.g. 3–4 days of each week for 12 months, or 6 full months of the past 12), except for infants younger than 6 months, newly married women, and domestic help, lodgers, and agricultural labourers who are currently in the household and will be staying in the household for longer than this but arrived less than 12 months ago.

### Interventions

#### The foundational intervention approach

**Fig. 2:**
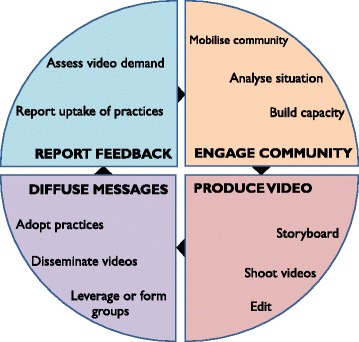
Digital Green’s approach to agricultural extension using facilitated video disseminations in community groups

All three interventions build on a participatory video-based approach designed by Digital Green. Their approach is a novel method of agricultural extension that shows low-cost participatory videos in women’s groups and farmers’ groups. The foundational Digital Green approach is illustrated in Fig. 2, described below, and shown in Digital Green’s explanatory video [44].

1. *Participatory identification of content*. Digital Green, with local implementing partners, consults with key community members to identify relevant video topics. Then, working with technical experts, they identify ‘packages of practices’ that relate to these topics and address key constraints in improving agricultural productivity.
2. *Training and local production of videos*. Selected community members are offered training on storyboarding, video production, and editing. The community video production crew create a storyboard that is reviewed by subject matter experts. The videos feature local community members demonstrating an agricultural practice in their local language. These ‘actors’ may be early adopters of these practices. If a new concept is to be introduced within the community, a government extension worker is often included in the video to increase the credibility of its messaging. All approved videos are also uploaded on Digital Green’s YouTube channel [44].
3. *Video dissemination*. Videos are disseminated through community-level groups such as self-help groups (SHGs, women’s groups involved in saving and lending activities). A trained facilitator (identified from within the community) consults group members to decide on the date, time, and venue of each video screening. The facilitator shows videos using low-cost, battery-operated, pocket-sized projectors (Pico projectors). During the screening, the facilitator pauses the video at strategic points and encourages viewers to discuss and reflect on the practices shown in the video.
4. *Follow-up visits*. After the screening, the facilitator conducts follow-up visits at the group members’ homes or farms to verify whether the viewer has adopted the key promoted practices and can recall the main messages shown in the video.
5. *Monitoring*. Details of each video, including its viewership, knowledge recall, and adoption of practices are collated in Digital Green’s monitoring system. Qualitative feedback from video disseminations and follow-up visits is also gathered during staff review meetings, and together with monitoring data, local implementers identify the content of future videos in an iterative feedback loop.

The key innovations under this trial are to (1) adapt the foundational approach of agricultural extension with videos to make it ‘nutrition-sensitive’, (2) integrate nutrition-specific video topics into an NSA extension intervention, and (3) enhance participatory approaches by incorporating nutrition-specific PLA approaches with an NSA extension intervention, to encourage uptake of nutrition-specific behaviours. Definitions of nutrition-specific and NSA interventions are given in Table 1.

**Table 1:** UPAVAN’s operational definition of nutrition-specific and nutrition-sensitive agriculture interventions

*Nutrition-specific* interventions or programs address the immediate determinants of nutrition status. For UPAVAN, this means age-appropriate feeding practices and adequate nutrient intake for children, maternal care to ensure rest and optimal nutrition, and care during child illness [7].	
*Nutrition-sensitive agriculture* addresses the underlying causes of undernutrition and incorporates specific nutrition goals. For UPAVAN, this means increasing physical, economic, and socio-cultural access to nutritious food year-round; increasing resources for health and nutrition at individual and household levels; improving women’s decision-making in agriculture activities, time use, and use of income; and reducing workload for pregnant and lactating women. Nutrition-sensitive agriculture does no harm to humans or the environment	

#### UPAVAN interventions

**Fig. 3:**
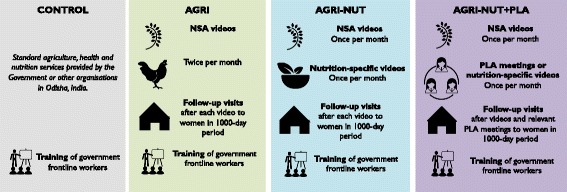
Overview of UPAVAN intervention components in each arm

A summary overview of the interventions is illustrated in Fig. 3.

NSA and nutrition-specific practices for UPAVAN are based on formative research undertaken by the UPAVAN team (focus group discussions, transect walks, and a seasonal calendar) [45] and the available global and local evidence. The UPAVAN team then prioritise the practices to promote in the interventions based on the following criteria: local relevance and acceptability, feasibility of adoption by participants, potential for uptake, and hypothesised impact on dietary diversity and nutritional status of mothers and children in the local context. A sample list of video and meeting topics, with rationale for the NSA topics, is provided in Additional file 1.

All arms, including the control arm, receive any services provided by the government or other non-governmental organisations. As a benefit to all participants, government frontline nutrition and health workers (Anganwadi workers and Accredited Social Health Activists) in all arms, including the control, receive a residential 2-day training course in maternal, infant, and young child nutrition.

##### Common features of all intervention arms

All intervention arms receive the following:

- Two facilitated group meetings per month per group over 32 months, of varying content between arms, that are run by local, trained, paid facilitators.
- In at least half of the meetings (all meetings in AGRI; half of the meetings in AGRI-NUT and AGRI-NUT+PLA), groups will view and discuss NSA videos on a pre-defined set of prioritised practices.
- Follow-up visits to all participating pregnant women and mothers of children aged 0–23 months after each video viewing, or PLA meeting where applicable. The purpose of these visits is to maintain rapport with the participant, check if the participant recalls or has adopted the key practices discussed in the video, reinforce messages shown in the videos and clarify if needed, when relevant strengthen the link between participants and community frontline workers, and encourage attendance at the next meeting.

##### Distinguishing features

The distinguishing features between arms are as follows:

- *AGRI*. Women’s groups view and discuss two NSA videos each month, following the foundational approach.
- *AGRI-NUT*. Women’s groups view and discuss two participatory videos each month, following the foundational approach but with 50% of the videos covering NSA topics (half of the videos shown in the AGRI arm) and 50% covering nutrition-specific behaviour change topics.
- *AGRI-NUT+PLA*. Each month, women’s groups view one NSA video (half of the videos from the AGRI arm) and have one PLA meeting on nutrition-specific topics. Some of these PLA meetings are discussion-based and some are facilitated disseminations of videos on nutrition-specific topics. The videos produced in this arm arise out of relevant discussions from the PLA meetings and so are different from the nutrition-specific videos in AGRI-NUT that use the foundational approach of identifying content described earlier. Facilitators will also only conduct follow-up visits when relevant — that is, after all NSA videos and PLA meetings that contain clear nutrition-specific messages.

**Fig. 4:**
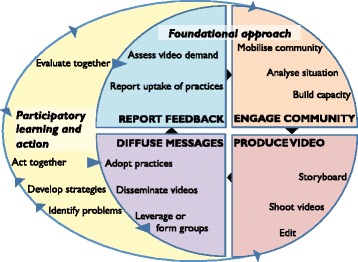
Illustration of how the four phases of the Participatory Learning and Action cycle feed into the foundational approach of facilitated video dissemination in groups in AGRI-NUT+PLA

The linkages between the foundational model and the PLA cycle are illustrated in Fig. 4.

The PLA cycle comprises four phases. In the first phase, group members identify and prioritise locally salient maternal and child nutrition problems by discussing local practices using games and picture cards. In the second phase, they explore the causes and effects of the prioritised problems using storytelling and discussions. Groups plan locally feasible strategies to address the problems, decide on roles and responsibilities for implementing the strategies, and share their learning with the wider community. In the third phase, groups implement their strategies. In the fourth phase, groups informally evaluate their achievements and plan for the future. The PLA cycle is embedded into the group-based video dissemination and follow-up visits approach described above, so that the discussions in the PLA group meetings inform the content of nutrition-specific videos in arm 3 (AGRI-NUT+PLA).

#### Intended coverage

For the video disseminations (including videos from the PLA cycle), there will be approximately five to six women’s groups per 1000 population, with each group comprising approximately 20–25 members. Where possible, groups will be formed by linking existing women’s SHGs, typically merging two SHGs to create one video-viewing group. Where SHGs do not exist, new women’s groups will be formed. For the PLA group meetings, there will be two women’s groups per 1000 population, or more where clusters include remote hamlets or demand is high.

Although we plan to conduct fortnightly group meetings, we have also planned for some implementation breaks due to festivals and seasonal and logistical factors. We plan to implement a minimum of 54 meetings per group over the 32-month implementation period.

Pregnant women and mothers with children 0–23 months (1000-day period) who have attended the meetings will receive a follow-up visit. Other women in the 1000-day period who are not attending will be identified by facilitators through communication with the Anganwadi worker and encouraged to attend.

**Fig. 5:**
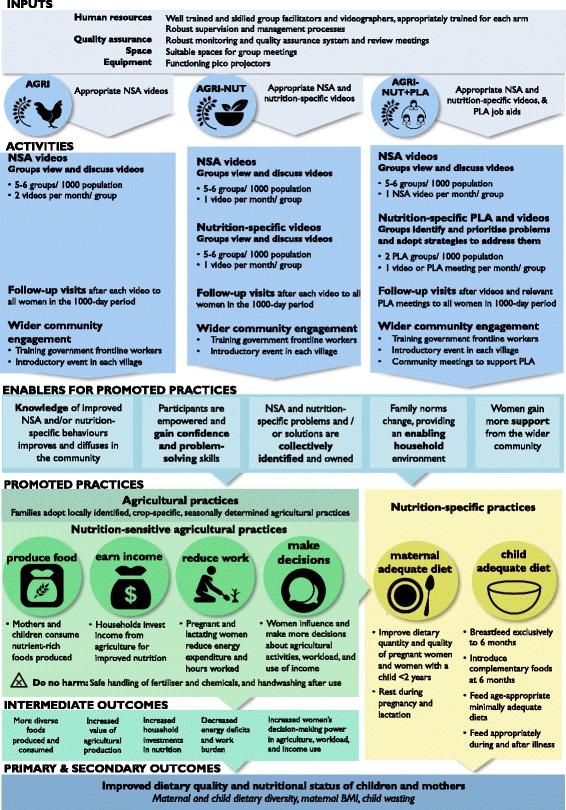
UPAVAN theory of change

The outline of nutrition-sensitive and nutrition-specific intervention topics, inputs, processes, and the mechanisms by which the interventions are hypothesised to affect dietary diversity and nutritional status is described in the theory of change in Fig. 5.

### Impact evaluation

The impact of the interventions will be evaluated through repeat cross-sectional household surveys at the same time of year (November to January) at baseline (2016–2017) and endline (2019–2020), in the same intervention and control clusters.

#### Trial outcomes

The primary outcomes are:

- Percentage of children (6–23 months of age) consuming ≥ 4 out of 7 food groups per day, based on a 24-h dietary recall answered by the mother or female primary caregiver
- Mean maternal BMI (kg/m^2^) of non-pregnant, non-postpartum (gave birth > 42 days ago) mothers or female primary caregivers of children aged 0–23 months

Secondary outcomes are:

- Percentage of mothers or female primary caregivers consuming ≥ 5 out of 10 food groups per day, based on a 24-h dietary recall
- Percentage of children with a weight-for-height z-score < -2 SD

Maternal BMI and child wasting are selected as outcome measures of chronic energy deficiency for adults and acute undernourishment for children. Both indicators predict mortality and morbidity [46, 47], vary seasonally so may change within the intervention timeframe, and are globally used so facilitate comparisons with other studies [47].

Dietary diversity scores for adults and children are selected as validated indicators of dietary adequacy of multiple micronutrients [48, 49]. These scores capture short-term dietary improvements; thus, they may also be amenable to change within the timeframe of this trial. Indicators for maternal and child actute malnutrition [50–53], women’s time use [54], and household agriculture production [55] are also included.

All trial outcomes are listed in Table 2.

**Table 2:** UPAVAN trial outcomes

Outcome	Indicator
*Primary outcomes*	
Child dietary diversity	• Percentage of children (6–23 months of age) consuming ≥ 4 out of 7 food groups per day (assessed by 24-h recall answered by the mother or female primary caregiver)
Maternal underweight	• Mean body mass index (BMI) (kg/m^2^) of non-pregnant, non-postpartum (gave birth > 42 days ago) mothers or female primary caregivers of children aged 0–23 months
*Secondary outcomes*	
Maternal dietary diversity	• Percentage of mothers or female primary caregivers consuming ≥ 5 out of 10 food groups per day (24-h recall) [50]
Child wasting	• Percentage of children (aged 0–23 months) who are wasted (weight for height < -2 SD)
*Other outcomes*	
Maternal wasting	• Percentage of pregnant and non-pregnant mothers or female primary caregivers with mid-upper arm circumference (MUAC) < 230 mm [51]
Child acute malnutrition	• Percentage of children (aged 6–23 months) with acute malnutrition (MUAC < 125 mm) [52]
Maternal and child haemoglobin (Hb) concentrations	• Mean Hb (g/dl) of children (6–23 months of age)
	• Mean Hb (g/dl) of non-pregnant mothers or female primary caregivers
Infant and young child feeding practices	• Percentage of children (aged 6–23 months) receiving the World Health Organization-recommended Minimum Acceptable Diet [53]
Women’s decision-making	• Percentage of women ‘empowered’ in women’s decision-making in productive and health-related domains, aggregated, measured using the Women’s Empowerment in Agriculture Index
Women’s time use	• Percentage of women ‘empowered’ in the women’s time use domain of the Women’s Empowerment in Agriculture Index [54]
Gender parity in agriculture	• Percentage of women achieving gender parity between themselves and a male household member, defined using the Women’s Empowerment in Agriculture Index
Household economic status and food security	• Mean per capita household share of food expenditures
	• Mean per capita total household expenditures
Household agriculture production	• Mean production diversity (count of the number of crops or livestock produced) [55] • Total value of agricultural production • Net value of agricultural production (= *value of agricultural production – costs*)

#### Sample size

The trial has 148 clusters, allocated in the ratio 1:1:1:1 (Control: AGRI: AGRI-NUT: AGRI-NUT+PLA), with on average 32 mother-child dyads (children aged 0–23 months; including 24 mother-child dyads aged 6–23 months) per cluster, giving a total sample size of 4736 mother-child dyads. Assuming a conservative intra-cluster correlation of 0.06, based on data from a trial conducted in Keonjhar [56], this sample size has 80% power with a 5% level of significance to detect a 9% absolute difference in child dietary diversity [57] between each intervention arm separately and the control arm, assuming a baseline of 22% of children with minimum dietary diversity (consumption ≥ 4 out of 7 food groups in the last 24 h [58]), and would allow us to detect a difference in mean maternal BMI of 0.3 kg/m^2^ [59] between each intervention arm separately and the control arm [60]. As the analysis will be restricted to comparisons between each intervention arm and the control arm, no adjustments were made for multiple comparisons.

#### Blinding

As with most interventions of this nature, participants cannot be ‘blinded’ to allocation status. Information on allocation of clusters to the respective implementation teams (the implementation team of Digital Green, VARRAT, and Ekjut including the project manager, project coordinator, arm coordinators, supervisors, video production teams, and facilitators) were only given after the initial NSA and basic nutrition training was given to the facilitation team. To minimise any contamination of nutrition-specific messages or PLA components across the arms, group facilitators and their supervisors, overseen by an arm coordinator, will not work across arms, and the arm-wise review meetings with the implementing teams will be held separately. The data manager conducted the randomisation, but the data collection team, principal investigator, and trial statistician are blinded to allocation. The trial statistician will conduct final analyses blinded to allocation.

#### Data collection and management

Before the baseline survey, a team of trained enumerators conducted a census in all 148 clusters, using standardised and documented procedures, to list all households with a child aged 0–23 months. In each cluster, well-trained data collectors invited a random sample of eligible households to participate in the survey and obtained individual written informed consent from participants.

The household survey comprised questionnaires for women and men (available in Additional file 2). Mothers or female primary caregivers were interviewed about nutrition, health, and empowerment in agriculture. Height, weight, mid-upper arm circumference (MUAC), and haemoglobin measurements were taken for each mother-child dyad. A male household member (if possible the caregiver’s husband, otherwise a male decision-maker, or a female decision-maker in a female-only household) was interviewed about agriculture and household socio-economic status. In addition, due to the length of the questionnaire and to reduce the burden on respondents, half of the male participants, randomly selected, were asked questions on their empowerment in agriculture while the other half were asked about household consumption and expenditure. The questionnaires were pilot tested before use.

Data collectors, ‘lab technicians’ (certified to take blood spots), and supervisors were organised into teams and collected data over the 3 months prior to implementation start; this will be repeated for 3 months for the endline survey. Supervisors and lab technicians took anthropometric measurements, and data collectors conducted the rest of the interview. Before data collection, measurements of maternal and child height, MUAC, and haemoglobin concentrations by supervisors and lab technicians were standardised against a ‘gold standard’ measurer, and we calculated inter- and intra-technical error of measurement and re-trained weaker teams. Data collectors were given 3 weeks of training, and we standardised the child dietary diversity score by comparing data collectors against a ‘gold standard’ interviewer.

Child length was measured using Seca 417 Infantometers, adult height using Seca 213 Stadiometers, maternal and child weight using PLAX-Cruzer scales, MUAC using MUAC tapes for children and Seca head circumference tapes for adults, and haemoglobin concentrations using HemoCue Hb 301 machines. Severely undernourished children (MUAC < 115 mm and/or bilateral pitting oedema) or mothers (MUAC < 210 mm or haemoglobin < 7 g/dl) were advised to consult the government frontline nutrition worker or Anganwadi worker for referral. Scales were calibrated weekly, and the stadiometers, infantometers, and HemoCues were calibrated at the start, middle, and end of the survey period. These procedures will be repeated at the endline survey.

The Quality Assurance team completed observation checklists on 10% of households to ensure that teams adhered to protocols, and they took duplicate readings on a subset of questions by revisiting 20% of households to check consistency.

For baseline, anthropometric data were entered using tablets (iBall Slide Snap 4G2) and transferred to a password-protected computer at the field office each day, and the rest of the survey was conducted on paper forms that were stored in a locked office. We reviewed the quality of anthropometric data by checking plausibility of values, digit preference, and missing values. Paper forms were submitted to the Quality Assurance team at the field office for a review of the logic, completeness, and plausibility of values, and errors were resolved by discussion with the data collector or revisit to the household. Data from paper questionnaires were double entered by trained data operators and discrepancies resolved by referring to the original source document.

The same procedures will be applied for the endline survey, although we plan to use tablets for all data capture.

The study team will make the full datasets available in anonymised form within 3 years of project completion. Before depositing into the data repository (LSHTM Data Compass, an institutional research data repository), data will be anonymised to protect participant confidentiality and converted into a file format suitable for long-term access, based on the recommendations of the UK Data Service [61]. The results will be published in a peer-reviewed journal.

#### Analysis plan

The analysis and presentation of results will be in line with the Consolidated Standards of Reporting Trials (CONSORT) Statement for cluster randomised controlled trials [62]. Descriptive statistics of demographic and outcome measures at baseline will be tabulated to ascertain any imbalance between arms.

The main analysis of the primary and secondary outcomes will be based on cross-sectional analyses that will compare the outcome at endline between the control arm and each of the intervention arms. All analysis will be carried out on groups as randomised (‘intention to treat’). All analyses will account for the nature of the distribution of the relevant outcome, and results will be presented as appropriate effects sizes (difference in means between arms; odds ratios) with a measure of precision (95% confidence intervals). Generalised estimating equations will be used to account for clustering. Analyses will adjust for baseline by inclusion of the cluster mean of the outcome as a covariate in statistical models.

Unadjusted and adjusted results will be presented for all analyses. Covariates in adjusted analyses will be specified *a priori* and will include the strata (distance to the nearest town and proportion of Scheduled Castes/Scheduled Tribes).

This is a multi-arm trial and, to restrict the number of comparisons, no statistical comparisons will be made directly between intervention arms. We will restrict formal testing, and reporting of *p* values, to the primary and secondary outcomes and will only compare each of the intervention arms with the control arm. Thus, no formal adjustment for multiple comparisons will need to be made. All other outcomes will be reported and interpreted with caution.

Planned subgroup analyses will include the impact of mothers’ exposure to the interventions, caste, and wealth. All subgroup analyses will be performed by including a variable (or variables, as appropriate) for the subgroup and its interaction with the treatment in the model. Results will be interpreted with due caution. Full details of all analyses, including any additional covariates to be included in the adjusted models, will be set out *a priori* in a Statistical Analysis Plan that will be uploaded as an appendix prior to the start of the endline survey.

As we will conduct repeat cross-sectional surveys to assess the trial outcomes, loss to follow-up of specific individuals is not an issue. Questionnaire completion rates are expected to be high, so only a small proportion of missing data is expected, and it is unlikely that it will have to be accounted for in any analysis. Sensitivity analyses (such as best- and worst-case scenarios and inverse probability weighting) will be conducted for the primary outcomes.

#### Adverse events

We do not expect any adverse effects of the intervention, but the trial will be monitored for severe adverse events. Information on these events is collected and compiled on a quarterly basis. The principal investigator will review the quarterly severe adverse events report to assess the level of relatedness to intervention. These data, with allocation concealed, will be shared annually with the independent statistician on the Trial Steering Committee for review. If any problems arise, the Trial Steering Committee can request the data to be unblinded. The independent statistician will share his/her assessment in writing with the Trial Steering Committee and research team.

### Process evaluation

Our process evaluation has three objectives: (1) to assess the fidelity and quality of the interventions’ implementation, (2) to clarify mechanisms behind the intervention’s effect, and (3) to identify contextual factors associated with variation in effects on the primary outcomes.

#### Assessing fidelity and quality of the intervention’s implementation

We will report data on three domains related to fidelity and quality of implementation in the main trial results paper. First, we will assess intervention workers’ *knowledge* of NSA (all intervention arms) and maternal, infant, and young child nutrition (in the AGRI-NUT and AGRI-NUT+PLA arms), as well as their facilitation skills. We will test intervention workers’ knowledge during the implementation period and calculate average test scores per arm. Second, we will assess the *quality* of video disseminations and PLA meetings (AGRI-NUT+PLA), including quality of facilitation, through structured observation forms (all intervention arms). Finally, we will measure intervention *coverage* and exposure by arm, as specified below:

1. Percentage of pregnant woman and mothers of children younger than 2 years in the endline survey who are members of SHGs
2. Percentage of planned intervention events that were actually implemented
3. Percentage of pregnant woman and mothers of children younger than 2 years who have attended/received any planned intervention activities (video disseminations, PLA meetings, home visits) in the 6 months before the start of the endline survey
4. Mean number of total intervention activities (video disseminations, PLA meetings, follow-up visits) attended/received by women in the last 6 months before the start of the endline survey

#### Clarifying mechanisms behind the interventions’ effects

We will explore mechanisms behind the interventions’ effects through two main methods: (1) an assessment of which intervention components were activated, and which were not, using the theory of change; (2) mediation analyses.

##### Assessment of component activation

We will gather information from the interventions’ monitoring systems, the endline survey, and qualitative data described below to conduct a systematic assessment of the components activated by the interventions, using the theory of change as a guide. If the final trial analyses detect no effect on the primary and secondary outcomes, this systematic assessment will also allow us to investigate what shortcomings may have compromised the interventions’ effects.

We will use quantitative and qualitative data sources for the assessment. The intervention monitoring systems and endline survey provide data on exposure to the interventions. The trial’s endline survey also provides data on other intermediate outcomes in the theory of change. To further understand women’s experiences of, and responses to, the intervention, we will conduct semi-structured interviews with pregnant women (*n* = 5), mothers of children younger than 2 years (*n* = 5), their husbands (*n* = 10), and mothers-in-law (*n* = 10) per arm, or around a total of 30 interviews in each of the three intervention arms. Participants will be sampled purposively to represent the mix of caste and tribal groups present in the intervention areas. We will supplement the interviews with participant observations in a subsample of three participant observations per arm.

We will conduct around four focus group discussions per arm with women’s groups to understand reasons for attendance patterns, the groups’ ability to plan and implement strategies together, and dissemination of information beyond the group. We will sample women’s groups purposively, based on the trial strata and their longevity. We will also conduct two focus group discussions per arm with the frontline UPAVAN implementation staff to understand factors that supported or hindered the interventions’ implementation. We may also conduct one focus group discussion per arm with key government frontline health and nutrition workers.

We will collect data in two waves of around 3 months each, and may sample additional women or use new data collection techniques if analyses generate questions that require further exploration. A data collection group independent of the implementation team will collect the qualitative data. We will use a thematic approach to data analysis in order to capture themes related to the theory of change and other emergent themes.

##### Mediation analyses

We will explore the role of four hypothesised mediators of the effects of the interventions on maternal and child nutrition: agricultural production, household income, women’s workload, and women’s decision-making. Data on these potential mediators will be captured by the endline survey. We will explore, if appropriate, the role of these mediators through causal mediation analyses using the potential outcomes framework [63].

#### Identifying contextual factors associated with variations in outcomes

In addition to the pre-specified subgroup analyses, as a part of the process evaluation we will explore the role of other factors such as women’s group longevity associated with primary and secondary outcomes.

### Economic evaluation

The economic evaluation will be conducted from a societal perspective, taking into account all costs incurred by the implementing agencies (program costs), community and health care providers, and users or households, and all associated and measurable outcomes or benefits associated with the intervention.

The program costs will be estimated using a combination of activity-based costing and an ingredients approach [64]. Program costs will be collected prospectively from the project accounts of all implementing partners. These costs will be entered into an MS Excel data capture tool designed for this purpose. Costs incurred by government frontline workers and community level institutions such as *gram panchayat* (local, elected body of representatives) and health facilities will be collected retrospectively using key informant interviews and publicly available salary information. Costs incurred by households affected by the UPAVAN interventions will be collected using household surveys.

All costs will be estimated from an economic perspective, assessing the full impact on all parties of direct and indirect costs, including time costs and donated goods. All costs will be adjusted for inflation using the Indian Consumer Price Index and converted to International Dollars to adjust for purchasing power parity.

The economic evaluation will take the form of a cost-consequence analysis, comparing incremental costs of the interventions and incremental changes in the trial outcomes [65]. Incremental costs and significant outcomes will be listed separately, allowing policymakers to compare the incremental costs with the incremental consequences of the different UPAVAN interventions. Cost-consequence analysis has been recommended for complex interventions, such as UPAVAN, that have multiple health and non-health effects which are difficult to measure in a common unit [65, 66]. Robustness of the analysis results will be assessed using sensitivity analyses. A full protocol will be developed *a priori* in an economic evaluation plan.

## Discussion

### Limitations

Our study is not powered to test the differences between interventions due to the large sample size required and the resources available for this trial. The intervention components are not additive, and therefore the study will not be able disentangle the effects of individual components within arms. The reason is that we kept the number of meetings (video disseminations or PLA) at two per month to minimise the burden on participants and to standardise the number of meetings per month across all intervention arms. Had we made the intervention strictly additive, the number of meetings would have also been additive, making it difficult to distinguish between the effect of the number of meetings and the meeting content.

### Study implications

By examining the role of innovative agricultural interventions for improving maternal and child nutrition, the trial will inform ways in which nutrition- and agriculture-related services can be strengthened in rural communities. Specifically, our study will provide robust evidence on (1) the extent to which improving agricultural extension through participatory, low-cost, video-based approaches can improve agricultural productivity and diversity; household income and investments in nutrition-related expenditures; women’s decision-making; and reducing women’s workload and thus maternal and child nutrition outcomes; (2) whether integration of explicit nutrition-specific messages enhances the impact of agriculture interventions on maternal and child nutrition; and (3) the optimal level of participatory action required to improve nutrition outcomes. The trial will provide insights on whether women’s groups can provide an effective platform for integrated agriculture and nutrition interventions. Our process and economic evaluations will provide critical information for translational feasibility and scale-up. The lessons from this trial can be readily adopted at scale in UPAVAN partners’ existing and future collaborations with non-governmental organisations and governments across South Asia and Africa.

### Trial timeline and status

**Fig. 6:**
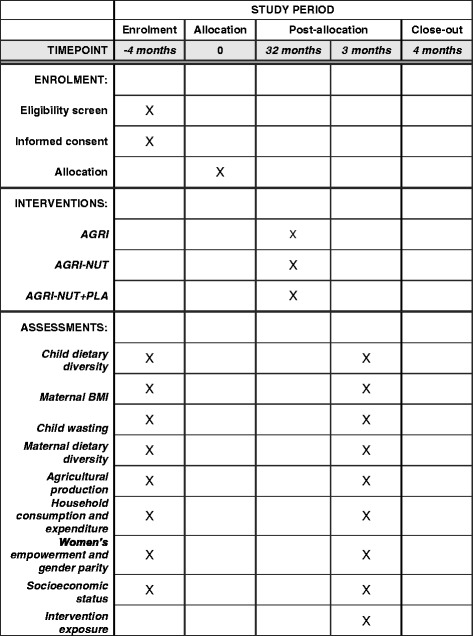
SPIRIT figure illustrating the schedule of enrolment, interventions, and assessments in UPAVAN

Ethics approvals and informed consent were obtained in October 2016 and clusters were randomised. The baseline survey was conducted between November 2016 and January 2017, and the interventions started in March 2017. With the endline survey starting in November 2019, the duration of intervention exposure is 32 months. The Standard Protocol Items: Recommendations for Interventional Trials (SPIRIT) timeline showing key trial time points is shown in Fig. 6, and the SPIRIT checklist is available in Additional file 3.

Version number 2; 21 December 2017.

## Additional files

Additional file 1: Sample list of video and meeting topics for 7 fortnights of implementation. (PDF 62 kb)

Additional file 2: Baseline questionnaires. (ZIP 6604 kb)

Additional file 3:  Standard Protocol Items: Recommendations for Interventional Trials checklist. (DOC 121 kb)

Additional file 4: Consent forms. (ZIP 1740 kb)

## Abbreviations

AGRI: Nutrition-sensitive agricultural extension intervention
AGRI-NUT: Nutrition-sensitive agricultural extension and nutrition-specific behaviour change intervention
AGRI-NUT+PLA: Nutrition-sensitive agricultural extension and nutrition-specific behaviour change intervention with a Participatory Learning and Action approach
BMI: Body mass index
MUAC: Mid-upper arm circumference
NSA: nutrition-sensitive agriculture
PLA: Participatory Learning and Action
SPIRIT: Standard Protocol Items: Recommendations for Interventional Trials
UPAVAN: Upscaling Participatory Action and Videos for Agriculture and Nutrition
VARRAT: Voluntary Association for Rural Reconstruction and Appropriate Technology

## References

[1] Black RE (2013). Maternal and child undernutrition and overweight in low-income and middle-income countries. Lancet.

[2] UNICEF (2016). The State of the World’s Children 2016. A fair chance for every child.

[3] Bhutta ZA (2013). Evidence-based interventions for improvement of maternal and child nutrition: what can be done and at what cost?. Lancet.

[4] Horton S, Steckel RH, Lomborg B (2013). Malnutrition: global economic losses attributable to malnutrition 1900–2000 and projections to 2050. How much have global problems cost the earth? A score card from 1900 to 2050.

[5] Avula R (2016). Reducing stunting in India: what investments are needed?. Matern Child Nutr.

[6] NFHS-4 (2016). National Family Health Survey, India.

[7] Ruel MT (2013). Nutrition-sensitive interventions and programmes: how can they help to accelerate progress in improving maternal and child nutrition?. Lancet.

[8] Headey D, Chiu A, Kadiyala S (2011). Agriculture's role in the Indian enigma: help or hindrance to the crisis of undernutrition?. Food Security.

[9] Kadiyala S (2014). Agriculture and nutrition in India: mapping evidence to pathways. Ann N Y Acad Sci.

[10] Ruel MT (2001). Can food-based strategies help reduce vitamin A and iron deficiencies? A review of recent evidence. Policy Review 5.

[11] Masset E (2012). Effectiveness of agricultural interventions that aim to improve nutritional status of children: systematic review. BMJ.

[12] Leroy JL, Frongillo EA (2007). Can interventions to promote animal production ameliorate undernutrition?. J Nutr.

[13] Girard AW (2012). The effects of household food production strategies on the health and nutrition outcomes of women and young children: a systematic review. Paediatr Perinat Epidemiol.

[14] Department for International Development (DFID) (2014). Can agriculture interventions promote nutrition?.

[15] Webb P. Impact pathways from agricultural research to improved nutrition and health: literature analysis and research priorities. Rome: Food and Agriculture Organization and Geneva: World Health Organization; 2013.

[16] Ruel MT, Quisumbing A, Balagamwala M. Nutrition-sensitive agriculture: what have we learned and where do we go from here? IFPRI Discussion Paper 01681. 2017.

[17] Olney DK (2016). A 2-year integrated agriculture and nutrition program targeted to mothers of young children in Burkina Faso reduces underweight among mothers and increases their empowerment: a cluster-randomized controlled trial. J Nutr.

[18] Osei A (2017). Combining home garden, poultry, and nutrition education program targeted to families with young children improved anemia among children and anemia and underweight among nonpregnant women in Nepal. Food Nutr Bull.

[19] Global Panel on Agriculture and Food Systems for Nutrition (2014). How can agriculture and food system policies improve nutrition? Technical Brief.

[20] FAO and WHO (2013). Overview of nutrition sensitive food systems: policy options and knowledge gaps.

[21] Anderson J (2008). Background paper for the World Development Report 2008: Agricultural advisory services.

[22] Swanson BE (2008). Global review of good agricultural extension and advisory service practices.

[23] Anderson JR (2006). The rise and fall of training and visit extension: an Asian mini-drama with an African epilogue. World Bank Policy Research Working Paper 3928.

[24] Glendenning CJ, Babu S (2011). Decentralization of public-sector agricultural extension in India: the case of the district-level Agricultural Technology Management Agency (ATMA).

[25] Food and Agriculture Organization (FAO) (2011). The state of food and agriculture 2010–11. Women in agriculture: closing the gender gap for development.

[26] Rivera WM (2001). Agricultural and rural extension worldwide: options for institutional reform in the developing countries.

[27] Birner R, Anderson JR (2007). How to make agricultural extension demand-driven? The case of India's agricultural extension policy. IFPRI Discussion Paper 00729, vol. 729.

[28] Davis K (2008). Extension in sub-Saharan Africa: overview and assessment of past and current models and future prospects. J Int Agric Ext Educ.

[29] Chapman R, Slaymaker T (2002). ICTs and rural development: review of the literature, current interventions and opportunities for action. Working Paper 192.

[30] Aker JC (2011). Dial “A” for agriculture: a review of information and communication technologies for agricultural extension in developing countries. Agric Econ.

[31] Xiaolan F, Akter S (2011). The impact of ICT on agricultural extension services delivery: evidence from the rural e-services project in India. TMD Working Paper Series.

[32] Casaburi L, Kremer M, Mullainathan S, Ramrattan R. Harnessing ICT to increase agricultural production: Evidence from Kenya. Harvard University. Draft working paper; 2014. https://arefiles.ucdavis.edu/uploads/filer_public/2014/03/27/casaburi_et_al_ict_agriculture_20140306.pdf.

[33] Gandhi R (2009). Digital green: participatory video and mediated instruction for agricultural extension. Inf Technol Int Dev.

[34] Toyama K (2017). Digital Green RCT: final report with preliminary analysis.

[35] Vodopivec-Jamsek V (2012). Mobile phone messaging for preventive health care. Cochrane Database Syst Rev.

[36] Flax VL (2014). Integrating group counseling, cell phone messaging, and participant-generated songs and dramas into a microcredit program increases Nigerian women’s adherence to international breastfeeding recommendations. J Nutr.

[37] Jiang H (2014). Effect of short message service on infant feeding practice: findings from a community-based study in Shanghai, China. JAMA Pediatr.

[38] Prost A (2013). Women's groups practising participatory learning and action to improve maternal and newborn health in low-resource settings: a systematic review and meta-analysis. Lancet.

[39] Harris-Fry HA (2016). Formative evaluation of a participatory women's group intervention to improve reproductive and women's health outcomes in rural Bangladesh: a controlled before and after study. J Epidemiol Community Health.

[40] Nair N (2017). Effect of participatory women’s groups and counselling through home visits on children’s linear growth in rural eastern India (CARING trial): a cluster-randomised controlled trial. Lancet Glob Health.

[41] Morrison J (2010). Understanding how women’s groups improve maternal and newborn health in Makwanpur, Nepal: a qualitative study. Int Health.

[42] Census of India, D.o.C.O.i. Orissa (2011). Census of India 2011, Odisha, District Census Handbook, Kendujhar, Village and Town Wise Primary Census Abstract (PCA).

[43] Odisha district website. http://ordistricts.nic.in/district_profile/economy.php. Accessed 26 Oct 2017.

[44] Digital Green (2014). The Digital Green Story.

[45] Aakesson A, Cunningham S, Granger K (2017). Using farming families’ perspectives to inform recommended priority practices: UPAVAN formative research report.

[46] Sauvaget C (2008). Body mass index, weight change and mortality risk in a prospective study in India. Int J Epidemiol.

[47] World Health Organization (2000). Physical status: use and interpretation of anthropometry.

[48] Working Group on Infant and Young Child Feeding Indicators (2006). Developing and validating simple indicators of dietary quality and energy ntake of infants and young children in developing countries: summary of findings from analysis of 10 data sets.

[49] Arimond M (2010). Simple food group diversity indicators predict micronutrient adequacy of women’s diets in 5 diverse, resource-poor settings. J Nutr.

[50] FAO and FHI 360 (2016). Minimum dietary diversity for women: a guide to measurement.

[51] Tang AM (2013). Use of cutoffs for mid-upper arm circumference (MUAC) as an indicator or predictor of nutritional and health-related outcomes in adolescents and adults: a systematic review.

[52] WHO and UNICEF (2009). WHO child growth standards and the identification of severe acute malnutrition in infants and children: joint statement by the World Health Organization and the United Nations Children’s Fund.

[53] WHO (2010). Indicators for assessing infant and young child feeding practices part 2: measurement.

[54] Malapit H (2015). Instructional Guide on the Abbreviated Women’s Empowerment in Agriculture Index (A-WEAI).

[55] Sibhatu KT, Krishna VV, Qaim M (2015). Production diversity and dietary diversity in smallholder farm households. Proc Natl Acad Sci.

[56] Nair N (2015). Participatory women’s groups and counselling through home visits to improve child growth in rural eastern India: protocol for a cluster randomised controlled trial. BMC Public Health.

[57] Olney DK (2015). A 2-year integrated agriculture and nutrition and health behavior change communication program targeted to women in Burkina Faso reduces anemia, wasting, and diarrhea in children 3–12.9 months of age at baseline: a cluster-randomized controlled trial. J Nutr.

[58] NFHS-3. National Family Health Survey. 2005–6. http://rchiips.org/NFHS/nfhs3.shtml. Accessed 26 Oct 2017.

[59] Kumar N, Quisumbing AR (2011). Access, adoption, and diffusion: understanding the long-term impacts of improved vegetable and fish technologies in Bangladesh. J Dev Effectiveness.

[60] Donner A (1998). Some aspects of the design and analysis of cluster randomization trials. Appl Stat.

[61] Corti L, Metzler K (2014). Managing and sharing research data: a guide to good practice.

[62] Campbell MK (2012). Consort 2010 statement: extension to cluster randomised trials. BMJ.

[63] Kosuke I, Keele L, Tingley D (2010). A general approach to causal mediation analysis. Psychol Methods.

[64] Johns B, Baltussen R, Hutubessy R (2003). Programme costs in the economic evaluation of health interventions. Cost Effect Res Allocation.

[65] Drummond MF (2015). Methods for the economic evaluation of health care programmes.

[66] NICE (2013). How NICE measures value for money in relation to public health interventions.

