# Supplementary material for: Upscaling Participatory Action and Videos for Agriculture and Nutrition (UPAVAN) trial comparing three variants of a nutrition-sensitive agricultural extension intervention to improve maternal and child nutritional outcomes in rural Odisha, India: study protocol for a cluster randomised controlled trial
Source: Trials. 2018 Mar 9;19:176. doi: 10.1186/s13063-018-2521-y (PMC5845188; doi:10.1186/s13063-018-2521-y)
Supplement: Supplementary file 1 — Sample list of video and meeting topics for 7 fortnights of implementation. (PDF 62 kb) [file 13063_2018_2521_MOESM1_ESM.pdf]

**UPAVAN plan**

**Example of 7 fortnights**

| | AGRI | | | | AGRI-NUT | AGRI-NUT+PLA |
|---|---|---|---|---|---|---|
| **Month** | **Topic** | **Agriculture practice** | **NSA pathway and practice** | **Rationale** | **Topic** | **Topic** |
| 15 Sep 2017 | Carrot | Seed treatment and planting | PRODUCE FOOD<br><br>Women in 1000-day period and children consume carrots | • Carrot grown in this season, in kitchen gardens (the last video).<br>• Carrots are nutrient rich.<br>• At this time of year, women are engaged in paddy harvesting and weeding so carrots are a good crop to promote because they are not labour intensive | Nutrition during pregnancy and lactation | 5th PLA meeting in which groups prioritise and vote on the problems that they would like to address. |
| 5 Oct 2017 | Cowpea | Combatting cowpea diseases (jau poka and sahebi poka) | PRODUCE FOOD<br><br>Women in 1000-day period and children consume cowpea | • Cow pea is protein rich and the only pulse that is commonly grown in this area.<br>• Cowpea is grown at this time, mainly in kitchen gardens.<br>• Farmers struggle with cowpea diseases - Oct is the right time to address this. | Cow pea | Cow pea |
| 25 Oct 2017 | Composting | Digging the pit, and the key ingredients required for composting | DO NO HARM<br><br>Hand-washing and environmental safety during composting | • Composting is important at this time of year for future planting in the next year. | Adding one extra snack between lunch and dinner | 6th PLA meeting in which map locally available resources |
| 10 Nov 2017 | Paddy drying and storage | Ways to store paddy and detect fungus | REDUCE WORK<br><br>Women are involved in decisions about labour sharing for harvesting by | • Appropriate time of year for drying paddy<br>• Farmers experience post-harvest | Paddy drying and storage | Paddy drying and storage |

| | | | | | | |
|---|---|---|---|---|---|---|
| | | | pregnant and lactating women. | losses due to fungus | | |
| 25 Nov 2017 | Spending decisions | All NSA | MAKE DECISIONS<br><br>Women are involved in decisions about how much rice to consume, sell, process (for value addition), and save for seeds. Women are involved in decisions about using income for nutrition | • Budget planning is important at this time of year because rice has just been harvested, and there are many festivals. | Spending decisions | Spending decisions |
| 10 Dec 2017 | Oyster mushroom cultivation | | MAKE DECISIONS<br><br>Joint decision-making around how to spend income for nutrition (with emphasis on what should be bought) | • Topic demanded by community.<br>• Straw is easily available to build a mushroom bed; other inputs are chea.<br>• Good for income (not particularly nutrient-rich), around 100-120 Rs/kg.<br>• There is a high market demand.<br>• Cheap inputs.<br>• Easy for women to do herself, whilst taking care of children.<br>• Helps with the income flow shortages in January. | Overcoming concerns about eating less during pregnancy (fear of having a large baby, eating down, fasting, restriction, morning sickness, feeling full) | 7$^{th}$ PLA meeting in which groups understand the causes and effects of two prioritized problems |
| 26 Dec 2017 | Cultivation of different species of Amaranthus | Planting and spacing techniques | PRODUCE FOOD<br><br>Consume Amaranthus year-round | • Topic demanded by community.<br>• Appropriate time of year, during food and income flow shortages | Cultivation of different species of Amaranthus | Cultivation of different species of Amaranthus |
