# Supplementary material for: Upscaling Participatory Action and Videos for Agriculture and Nutrition (UPAVAN) trial comparing three variants of a nutrition-sensitive agricultural extension intervention to improve maternal and child nutritional outcomes in rural Odisha, India: study protocol for a cluster randomised controlled trial
Source: Trials. 2018 Mar 9;19:176. doi: 10.1186/s13063-018-2521-y (PMC5845188; doi:10.1186/s13063-018-2521-y)
Supplement: Supplementary file 2 — Baseline questionnaires. (ZIP 6604 kb) [file 13063_2018_2521_MOESM2_ESM.zip › Baseline_Female_finalR1.pdf]

Upscaling Participation and Videos for Agriculture and Nutrition (UPAVAN)
କୃଷି ଓ ପୋଷଣ ନିମନ୍ତେ ସହଯୋଗୀତାର ମାତ୍ରାକୁ ଅଧିକ କରିବା ଏବଂ ଭିଡିଓ କରିବା (ଉପବନ)

Baseline survey | ବେସଲାଇନ ସର୍ବେକ୍ଷଣ

Respondent: mother/primary caregiver | ଉତ୍ତରଦାତା: ମା/ପ୍ରାଥମିକ ଯତ୍ନକାରୀ

1. OPERATIONAL DEFINITIONS | କାର୍ଯ୍ୟକାରିଣୀ ପରିଭାଷା

Instructions. The operational definitions are very important! The criteria could be different from other studies you may be familiar with. For the definition of household especially, you should keep in mind that you should not include those people who do not meet these criteria. Please discuss any questions with your supervisor.
ଏଠାରେ କାର୍ଯ୍ୟକାରିତାର ପରିଭାଷା ବହୁତ ଗୁରୁତପୁର୍ଣ ଅଟେ। ଏହି ସର୍ଭେର ନୀତି/ମାନଦଣ୍ଡ ଆପଣ ଜାଣିଥିବା ଅନ୍ୟାନ୍ୟ ସର୍ଭେ ଠାରୁ ଭିନ୍ନ ଅଟେ । ବିଶେଷ କରି ପରିବାରର ପରିଭାଷା ନିମନ୍ତେ ଆପଣ ଏହା ମନରେ ରଖିବା ଉଚିତ ଯେ ଯେଉଁମାନେ ଏହି ମାନଦଣ୍ଡ/ନୀତି ମଧ୍ୟକୁ ଆସୁନାହାନ୍ତି ଆମେ ସେମାନଙ୍କୁ ସମ୍ପୃକ୍ତ କରୁନାହୁଁ । ଯଦି କୌଣସି ପ୍ରଶ୍ନ ଅଛି ତେବେ ସୁପରଭାଇଜର ସହ ଆଲୋଚନା କରନ୍ତୁ

**Index child**<br>***ଚୟନ ଶିଶୁ***	A child 0-23 completed months of age with a female primary caregiver aged 15 to 49 years inclusive<br>ଯେଉଁ ଶିଶୁର ବୟସ ଜନ୍ମ ହେବା ଦିନ ଠାରୁ ୨୩ ମାସ ବୟସ ସମ୍ପୂର୍ଣ ହେଉଥିବ ଓ ୧୫ ବର୍ଷ ବୟସରୁ ଅଧିକ ୪୯ ବର୍ଷ ବୟସରୁ କମ ହେଉଥିବା ଜଣେ ପ୍ରାଥମିକ ଯତ୍ନକାରୀ ସେହି ଶିଶୁର ଯତ୍ନ ନେଉଥିବେ ।<br>The index child is chosen randomly from eligible children within the household<br>ଚୟନ ଶିଶୁଟି ପରିବାର ମଧ୍ୟରୁ ରାଣ୍ଡମ ପ୍ରକ୍ରିୟାରେ ଚୟନ କରାଯିବ ।<br>- This can include a child who is a twin, triplet or quadruplet/ଏହି ଶିଶୁ ଯାଁଳା ଶିଶୁ, ଏକାଠି ତିନୋଟି ଜନ୍ମ ହୋଇଥିବା ଶିଶୁ ବା ଚାରୋଟି ଜନ୍ମ ହୋଇଥିବା ଶିଶୁ ମଧ୍ୟରୁ ଗୋଟିଏ ହୋଇପାରେ<br>- Exclude any children with a discernible disability that impairs them from participating in the study, or affects their standing height or recumbent length/ଯେଉଁ ଶିଶୁର ଆଖିକୁ ଦେଖା ଯାଉଥିବା ଭଳି କୌଣସି ଭିନ୍ନକ୍ଷମତା ଥିବ (ଯେପରିକି ଛିଡା ହୋଇଥିବା ବେଳେ ଉଚ୍ଚତା କିମ୍ବା ଶୋଇଥିବା ବେଳେ ଲମ୍ବ) ଯାହାକି ଆମ ସର୍ଭେରେ ସହଭାଗିତାରେ ବାଧା ସୃଷ୍ଟି କରୁଥିବ ସେପରି ଶିଶୁକୁ ଏହି ସର୍ଭେ ଅନ୍ତର୍ଭୁକ୍ତ କରାଯିବ ନାହିଁ
**Mother**<br>ମା	Mother of the index child (so has at least one living child 0-23 completed months of age) with no discernible disability that affects their height or prevents them from participating in the study<br>ସୁଚିତ ହୋଇଥିବା ଶିଶୁର ମା (ଅତିକମ୍ ରେ 0-23 ମାସ ବୟସ ର ଗୋଟିଏ ଜୀବିତ ଶିଶୁ ଥିବା) ଯିଏ କି କୌଣସି ପ୍ରକାର ଦୃଷ୍ଟିଗୋଚର ହେଉଥିବା ଭିନ୍ନକ୍ଷମ ହୋଇନଥିବେ ଯାହା ସେମାନଙ୍କ ଉଚ୍ଚତାକୁ ପ୍ରଭାବିତ କରୁଥିବ କିମ୍ବା ଏହି ଅଧ୍ୟୟନରେ ଅଂଶ ଗ୍ରହଣ କରିବାରେ ବାଧା ସୃଷ୍ଟି କରୁଥିବ ।
**Female Primary Caregiver**<br>**ମହିଳା ପ୍ରାଥମିକ ଯତ୍ନକାରୀ**	A female primary caregiver who/ଜଣେ ମହିଳା ପ୍ରାଥମିକ ଯତ୍ନକାରୀ ଯିଏକି:<br>- Is responsible for caring for the child, AND/ଶିଶୁଟିର ଯତ୍ନ ନେବାର ଦାୟିତ୍ୱ ନେଇଥିବେ, ଏବଂ<br>- Is aged 15 to 49 years inclusive, AND/ତାଙ୍କ ବୟସ ୧୫ ବର୍ଷରୁ ୪୯ ବର୍ଷ ମଧ୍ୟରେ ହୋଇଥିବ, ଏବଂ<br>- has no discernible disability that affects their height or prevents them from participating in the study/ ତାଙ୍କର କୌଣସି ଦେଖାଯାଉଥିବା ଭଳି ଭିନ୍ନକ୍ଷମ ନଥିବ, ଯାହା ସେମାନଙ୍କ ଉଚ୍ଚତାକୁ ପ୍ରଭାବିତ କରୁଥିବ କିମ୍ବା ଏହି ଅଧ୍ୟୟନରେ ଅଂଶ ଗ୍ରହଣ କରିବାରେ ବାଧା ସୃଷ୍ଟି କରୁଥିବ ।<br>The primary caregiver is interviewed only where the mother is absent/dead etc. (Absent means not available after 3 attempts of visits)/ଯେଉଁଠାରେ ମା ଅନୁପସ୍ଥିତ/ମୃତ ଅଛନ୍ତି ତେବେ ସେହି କ୍ଷେତ୍ରରେ ପ୍ରାଥମିକ ଯତ୍ନକାରୀଙ୍କ ସାକ୍ଷାତକାର କରାଯାଇପାରିବ ।<br>Questions on pregnancy and initiation of breastfeeding will not be administered to this respondent/ ଉତ୍ତରଦାତା ଗର୍ଭାବସ୍ଥା କିମ୍ବା ସ୍ତନ୍ୟପାନ ଆରମ୍ଭ କରିବା ସମ୍ବନ୍ଧୀୟ ପ୍ରଶ୍ନର ଉତ୍ତର ଦେବେ ନାହିଁ ।
**Male Respondent/**<br>**ପୁରୁଷ ଉତ୍ତରଦାତା**	Spouse of the sample mother with no discernible disability that prevents them from responding to the study; if not available, another adult male who is responsible for making major household economic decisions/ଚୟନ କରାଯାଇଥିବା ମା' ଙ୍କ ସ୍ୱାମୀ ଯାହାଙ୍କର ଅଧ୍ୟୟନରେ ବାଧା ସୃଷ୍ଟି କଲାଭଳି ଦୃଶ୍ୟମାନ ହେଉଥିବା ଶାରିରୀକ ଅକ୍ଷମତା ନ ଥିବ ଏବଂ ଯଦି ସ୍ୱାମୀ ନ ଥିବେ ତେବେ ପରିବାରର ଆର୍ଥିକ ନିଷ୍ପତ୍ତି ନେବା ଦାୟୀତ୍ୱରେ ଥିବା ଅନ୍ୟଜଣେ ବୟସ୍କ ପୁରୁଷ ସଦସ୍ୟଙ୍କୁ ଚୟନ କରନ୍ତୁ ।<br>If there are no male decision-makers in the household, the 'male questionnaire' will be administered to another woman in the household who is responsible for, or participates in, agriculture-related decisions. In these cases, she will not respond to the WEAI questions/ଯଦି ସେହି ପରିବାରରେ କୌଣସି ପୁରୁଷ ନିଷ୍ପତ୍ତି ଗ୍ରହଣକାରୀ "ପୁରୁଷ ଉତ୍ତରଦାତା" ଉପସ୍ଥିତ ନଥାନ୍ତି, ତେବେ ଏହି ପ୍ରଶ୍ନାବଳୀ ସେହି ପରିବାରର ଜଣେ ମହିଳାଙ୍କୁ ପଚରାଯାଇ ପାରେ, ଯିଏକି ପରିବାରର କୃଷି ସମ୍ବନ୍ଧୀୟ ନିଷ୍ପତ୍ତି ନେବାରେ ଅଂଶଗ୍ରହଣ କରନ୍ତି । ଏପରି କ୍ଷେତ୍ରରେ ସେ **WEAI** ର ଉତ୍ତର ଦେବେନାହିଁ।

**Discernible disability/ ଆଖିକୁ ଦେଖାଯାଉଥିବା ଭିନ୍ନକ୍ଷମ**	Major impairment, including hearing impairment, or severe physical or mental illness that prevents the respondent from answering a survey. ଗୁରୁତର ଅକ୍ଷମତା ଯଥା : ବଧିରତା କିମ୍ବା ଗୁରୁତର ଶାରୀରିକ/ମାନସିକ ଅକ୍ଷମତା ଯାହା ଅଧ୍ୟୟନର ଉତ୍ତର ଦେବାରେ ବାଧା ସୃଷ୍ଟି କରୁଥିବ ।
**Household/ ପରିବାର**	A group of people who live together under the same roof and take food from the "same pot."/ ଏକ ବର୍ଗର ଲୋକ ଯେଉଁମାନେ ଗୋଟିଏ ଛାତ ତଳେ ରହନ୍ତି ଏବଂ ଗୋଟିଏ ହାଣ୍ଡିରୁ ଖାଆନ୍ତି ।
**Household member/ ପରିବାର ସଦସ୍ୟ**	Someone who has done ALL of the following/ଯେଉଁବ୍ୟକ୍ତି ନିମ୍ନଲିଖିତ ସମସ୍ତ ମାନଦଣ୍ଡ ଭିତରେ ଆସୁଥିବେ :<br>a) Lived in the household in this cluster for the past 12 months, and/ ଅତିକମରେ ସେହି କ୍ଲଷ୍ଟରର ସେହି ପରିବାରରେ12 ମାସବାସ କରୁଥିବ, ଏବଂ<br>b) Shares food from the same pot, and/ ସେହି ଛାତ ତଳେ ରହୁଥିବା ଅନ୍ୟ ସଦସ୍ୟ ମାନେ ଖାଉଥିବ ହାଣ୍ଡିରୁ ଖାଉଥିବ, ଏବଂ<br>c) Resided in the household regularly at least half of the time during the past 12 months (e.g. 3-4 days of each week for 12 months, or 6 full months of past 12 months)/ ଅତିକମରେ 12 ମାସ ମଧ୍ୟରେ ଅଧିକାଂଶ ସମୟ ନିୟମିତ ଭାବରେ ବାସ କରୁଥିବେ (12 ମାସ ମଧ୍ୟରେ ସାପ୍ତାହରୁ 3 ରୁ 4 ଦିନ କିମ୍ବା 12 ମାସ ମଧ୍ୟରୁ 6 ଟି ସମ୍ପୂର୍ଣ ମାସ ରହିଥିବ।<br>Even persons who are not blood relatives (such as servants, lodgers, guests or agricultural labourers) are included if they meet these three requirements/ ଏପରିକି ଚାକର, ଭଡାଟିଆ, ଅତିଥି କିମ୍ବା କୃଷି ଶ୍ରମିକ ଯେଉଁମାନେ କି ରକ୍ତ ସମ୍ପର୍କୀୟ ନହୋଇଥିଲେ ମଧ୍ୟ ସେମାନଙ୍କୁ ପରିବାର ସଦସ୍ୟ ରୂପେ ପରିଗଣିତ କରାଯିବ । ଯଦି ସେମାନେ ଏହି ତିନୋଟି ଆବଶ୍ୟକତା ପୂରଣ କରୁଥିବେ ।<br><u>Exceptions</u>: All of the following will be considered as a household member/:<br>A. An infant less than six months old/ ୬ ମାସରୁ କମ ବୟସର ଶିଶୁ<br>B. Someone who has joined the household through marriage less than 12 months ago/ ଯିଏ କି 12 ମାସରୁ କମ ସମୟରେ ବିବାହ କରି ପରିବାରରେ ସାମିଲ ହୁଅନ୍ତି ।<br>C. Servants, lodgers, and agricultural laborers currently in the household and will be staying in the household for a longer period but arrived less than 12 months ago/ଚାକର, ଭଡାଟିଆ, କୃଷି ଶ୍ରମିକ ପରିବାରକୁ 12 ମାସ ପୂର୍ବରୁ ଆସିଛନ୍ତି ମାତ୍ର ଦୀର୍ଘ ଦିନଧରି ରହିବେ ।
**Non-household member/** ଅଣ-ପରିବାର ସଦସ୍ୟ	Someone who stays in the same household but the household does not incur any costs for their food or the member does not take food from the same pot/ ଯେ କେହି ପରିବାରରେ ରହୁଛନ୍ତି କିନ୍ତୁ ତାଙ୍କର ଖାଇବା ଖର୍ଚ୍ଚ କେହି ବହନ କରନ୍ତି ନାହିଁ କିମ୍ବା ସେହି ହାଣ୍ଡିରୁ ଖାଆନ୍ତି ନାହିଁ ।<br>- *For example, if two brothers stay in the same house with their families but they do not share food costs and they cook separately, then they are considered two separate households/* ଉଦାହରଣ, ଯଦି ଦୁଇ ଭାଇ ପରିବାର ସହିତ ଗୋଟିଏ ଘରେ ରହୁଛନ୍ତି କିନ୍ତୁ ସେମାନେ ଅଲଗା ରୋଷେଇ କରୁଛନ୍ତି ତେବେ ସେମାନଙ୍କୁ ୨ ଟି ପରିବାର ଭାବରେ ବିଚାରକୁ ନିଆଯିବ ।<br><br>Generally, if one person stays more than 6 months out of the last 12 months outside the household, they are not considered household members even if others consider them as household members. Do not consider as household member/ସାଧାରଣତଃ, ଯଦି ଜଣେ ବ୍ୟକ୍ତି ପରିବାର ଠାରୁ ଗତ 12 ମାସରୁ 6 ମାସ ବାହାରେ ରହୁଛନ୍ତି ତାଙ୍କୁ ପରିବାରର ସଦସ୍ୟ ଭାବେ ବିଚାରକୁ ନିଆଯିବ ନାହିଁ:<br>- A person who died very recently/ ନିକଟରେ ଯେଉଁ ବ୍ୟକ୍ତିଙ୍କର ମୃତ୍ୟୁ ହୋଇଛି<br>- Someone who has left the household through marriage even if they are temporarily at home/ ଯଦି କେହି ବିବାହକରି ପରିବାର ଠାରୁ ଦୂରେଇ ଯାଇଛନ୍ତି, ତଥାପି ସେ ଅସ୍ଥାୟୀ ରୂପେ ପରିବାରରେ ରହୁଛନ୍ତି<br>- Servants, lodgers, guests and agricultural labourers who have left the household even if they are temporarily at home/ ଚାକର, ଭଡାଟିଆ, କୃଷି ଦିନ ମଜୁରିଆ ଘର ଛାଡିଛନ୍ତି ତଥାପି ଅସ୍ଥାୟୀ ରୂପେ ପରିବାରରେ ରହୁଛନ୍ତି ।<br>A married mother who is temporarily visiting her parental home, and so has not lived at the parental home for the past 12 months/ଜଣେ ବିବାହିତା ମା ଯିଏକି ଅସ୍ଥାୟୀ ଭାବେ ତାଙ୍କର ବାପଘରକୁ ଆସିଛନ୍ତି ଏବଂ ଗତ ୧୨ ମାସ ସେ ବାପଘରେ ରହିନାହାନ୍ତି ।

2. Household Identification / ପରିବାରରପରିଚୟ

Enter Village Code: | V | Household Unique Identification Code (HUIC): | Bl | C | V | St | Hh |

Variable name	Question	Answer
block	**2.1 Block**<br>Ghatagaon------------------------ 1<br>Harichandanpur ---------------- 2<br>Patna -------------------------------3<br>Keonjhar (Sadar) ---------------- 4	☐
cluster	2.2 Cluster	☐☐☐
village	2.3 Village	
hamlet	2.4 Hamlet	
st_number	2.5 Structure Number	☐☐☐
hh_number	2.6 HH Number	☐☐☐
**2.7 GIS Coordinates**		
gis_long	2.7.1 Longitude	☐☐.☐☐☐
gis_lat	2.7.2 Latitude	☐☐.☐☐☐
gis_alt	2.7.3 Altitude	☐☐.☐☐

3. Survey Team Identification / ସର୍ବେକ୍ଷଣ ଦଳର ପରିଚୟ

Variable name	Question	Name
interviewer	3.1 Interviewer / Field Investigator	
lt	3.2 Lab Technician	
supervisor	3.3 Field Supervisor	

4. **Date and Time of Interviewer Visits to the Household / ଅନୁସନ୍ଧାନ କାରୀଙ୍କ ପରିବାର ପରିଦର୍ଶନ**

Variable name	Question	Code	Answer
**4.1. 1st Visit to the household/** ପରିବାରକୁ ପ୍ରଥମ ପରିଦର୍ଶନ			
date_1	4.1.1 Date/ତାରିଖ	d d / m m / y y y y	
time_1	4.1.2 Time/ସମୟ	☐☐:☐☐	
respondent_present_1	4.1.3 Are any mothers / primary caregivers of children under 2 years available at home/ ଘରେ ୨ବର୍ଷରୁ କମ ଶିଶୁର ମା/ପ୍ରାଥମିକ ଯତ୍ନକାରୀ ଉପସ୍ଥିତ ଅଛନ୍ତି କି ?	Yes/ହଁ------------------------------ A<br>→ *go to Q4.4*<br>No-one at home / temporarily unavailable/ ନା ଘରେ କେହି ନାହାନ୍ତି/ଅସ୍ଥାୟୀ ଭାବେ ଉପସ୍ଥିତ ନାହାନ୍ତି ----------------------------------- B<br>→ *end the interview (go to Q4.2.1)*<br>Home not occupied / extended absence/ ଘର ବ୍ୟବହାର ହେଉନାହିଁ/ଦୀର୍ଘଦିନ ଧରି ଅନୁପସ୍ଥିତ ଅଛନ୍ତି -----------------------------------.C<br>→ *end the interview*	☐
**4.2. 2nd Visit to the household/** ପରିବାରକୁ ଦ୍ୱିତୀୟ ପରିଦର୍ଶନ			
date_2	4.2.1. Date/ତାରିଖ	d d / m m / y y y y	
time_2	4.2.2. Time/ସମୟ	☐☐:☐☐	
respondent_present_2	4.2.3. Are any mothers / primary caregivers of children under 2 years available at home/ ଘରେ ୨ବର୍ଷରୁ କମ ଶିଶୁର ମା/ପ୍ରାଥମିକ ଯତ୍ନକାରୀ ଉପସ୍ଥିତ ଅଛନ୍ତି କି ?	Yes/ହଁ------------------------------ A<br>→ *go to Q4.4*<br>No-one at home / temporarily unavailable/ ନା ଘରେ କେହି ନାହାନ୍ତି/ଅସ୍ଥାୟୀ ଭାବେ ଉପସ୍ଥିତ ନାହାନ୍ତି ----------------------------------- B<br>→ *end the interview (go to Q4.2.1)*<br>Home not occupied / extended absence/ ଘର ବ୍ୟବହାର ହେଉନାହିଁ/ଦୀର୍ଘଦିନ ଧରି ଅନୁପସ୍ଥିତ ଅଛନ୍ତି -----------------------------------.C<br>→ *end the interview*	☐
**4.3. 3rd Visit to the household/** ପରିବାରକୁ ତୃତୀୟ ପରିଦର୍ଶନ			
date_3	4.3.1. Date/ତାରିଖ	d d / m m / y y y y	
time_3	4.3.2. Time/ସମୟ	☐☐:☐☐	

Variable name	Question	Code	Answer
respondent_present_3	4.3.3. Are any mothers / primary caregivers of children under 2 years available at home/ ଘରେ ୨ବର୍ଷରୁ କମ ଶିଶୁର ମା/ପ୍ରାଥମିକ ଯତ୍ନକାରୀ ଉପସ୍ଥିତ ଅଛନ୍ତି କି ?	Yes/ହଁ-- A<br>→ *go to Q4.4*<br>No-one at home / temporarily unavailable/ ନା ଘରେ କେହି ନାହାନ୍ତି/ଅସ୍ଥାୟୀ ଭାବେ ଉପସ୍ଥିତ ନାହାନ୍ତି ---------------------------------- B<br>→ *end the interview*<br>Home not occupied / extended absence/ ଘର ବ୍ୟବହାର ହେଉନାହିଁ/ଦୀର୍ଘଦିନ ଧରି ଅନୁପସ୍ଥିତ ଅଛନ୍ତି ----------------------------------.C<br>→ *end the interview*	
mother_name	4.4. What is the name of the mother / primary caregiver/ ମା/ପ୍ରାଥମିକ ଯତ୍ନକାରୀଙ୍କ ନାମ କଣ?<br>If more than one mother or primary caregiver, select one for the child listing process/ଯଦି ଏକାଧିକ ମା କିମ୍ବା ପ୍ରାଥମିକ ଯତ୍ନକାରୀ ଅଛନ୍ତି ତେବେ ଶିଶୁ ତାଲିକା କରଣ ପ୍ରକ୍ରିୟାରେ ଜଣଙ୍କୁ ଚୟନ କରନ୍ତୁ I.		

5. Informed Consent and Selection of Index Child / ସହମତି ପତ୍ର ଏବଂ ଶିଶୁର ସୁଚୀ ଚୟନ

Consent: Provide information sheet and explain the purpose, process, risks and benefits of participating in the survey to the respondent. Take care to be very detailed on confidentiality and the voluntary nature of the study. Consent MUST BE obtained for all respondents at this stage including on behalf of children under two years, the mothers or primary caregivers of children under two, and the spouse/male respondent/main decision-maker. One paper copy of the information sheet and signed consent form must be provided to and retained by the household, and another signed copy of the consent forms must be retained with the enumerator and submitted to your supervisor

ସହମତ୍ତି ପତ୍ର: ସୂଚନା ପତ୍ରଟି ତାଙ୍କୁ ପ୍ରଦାନକରନ୍ତୁ ଓ ତାଙ୍କୁ ଏହି ସର୍ଭେର ଉଦ୍ଦେଶ୍ୟ, ପ୍ରକ୍ରିୟା, ବିପଦ ଓ ଲାଭ ବିଷୟରେ ବର୍ଣନା କରନ୍ତୁ । ସବିଶେଷ ବର୍ଣନା କଲାବେଳେ ଏହି ଆନୁଧ୍ୟାନର ଗୋପନୀୟତା ଓ ସ୍ୱେଚ୍ଛାକୃତତାର ଯତ୍ନନେବା ଉଚିତ । ଏହି ସ୍ତରରେ ସମସ୍ତ ଉତ୍ତରଦାତାଙ୍କ ସହମତି ସହିତ ଦୁଇବର୍ଷରୁ କମ ଶିଶୁଙ୍କ ନିମନ୍ତେ ସହମତି ମଧ୍ୟ ଜରୁରୀ ଅଟେ । ସୁଚନା ପତ୍ର ଓ ସହମତ୍ତି ପତ୍ରର ଅବିକଳ ନକଲ ପରିବାରର ସଦସ୍ୟଙ୍କୁ ପ୍ରଦାନ ଓ ସହମତ୍ତି ପତ୍ରରେ ପରିବାରର ସଦସ୍ୟଙ୍କର ଦସ୍ତଖତ କରି ସେଥିରୁ ଗୋଟିଏ ନକଲ ପରିବାର ସଭ୍ୟଙ୍କୁ ଜିମାଦେଇ ଅନ୍ୟଟି ଅନୁସନ୍ଧାନକାରୀ ନିଜ ପର୍ଯ୍ୟବେକ୍ଷକଙ୍କ ନିକଟରେ ଜମା କରିବେ ।

Variable name	Question	Code	Answer
consent_mother	5.1 Did the mother/primary caregiver give consent/<br>ମା/ପ୍ରାଥମିକ ଯତ୍ନକାରୀ ସହମତି ଦେଲେ କି?	Yes, written / thumbprint/ ହଁ,ଦସ୍ତଖତ / ଅଙ୍ଗୁଠି ଛାପ--------------------------1<br>Yes, verbal (witnessed by someone else e.g. neighbour or relative)/ ହଁ, ମୁହଁରେ କହିଲେ(ସାକ୍ଷୀଙ୍କ ଉପସ୍ଥିତିରେ, ଅନ୍ୟ କେହି ଯଥା ପଡୋଶୀ ବା ସାଙ୍ଗ ସାଥି)-----2<br>No/ ନା-------------------------------0<br>→ *If no, end the interview*	

Read aloud: *"I would like to ask you about all the children 0-23 completed months of age who are residents of this house".*
Start with the oldest child (aged 23 months or younger). Do not list any children with any discernible disability.
ଉଚ୍ଚ ସ୍ୱରରେ ପଢନ୍ତୁ: ମୁଁ ଆପଣଙ୍କ ଘରେ ରହୁଥିବା ୦-୨୩ ମାସର ସମସ୍ତ ପିଲାମାନଙ୍କ ବିଷୟରେ ପଚାରିବି । ବଡ ଶିଶୁ ଠାରୁ ଆରମ୍ଭ କରନ୍ତୁ (ବୟସ ୨୩ ମାସ କିମ୍ବା ଅଧିକ)ଦୃଷ୍ଟିଗୋଚର ହଉଥିବା ଭିର୍ନକ୍ଷମ ଶିଶୁଙ୍କୁ ଗଣନ୍ତୁ ନାହିଁ

Variable name	Question	Code	Answer
child_number	**5.1.1.** How many children do you have aged within 0-23 months?<br>ଆପଣଙ୍କ ଘରେ 0-୨୩ ମାସ ମଧ୍ୟରେ କେତେଜଣ ପିଲା ଅଛନ୍ତି?		
	Repeat for each child aged 23 months or younger		
	**Child 1**		

child_name0	5.2. Child name / ଶିଶୁର ନାମକଣ		
card0	5.3. Do you have mother and child protection (MCP) card for you and this child?<br>ଟିକାକରଣ କାର୍ଡବାମା ଓ ଶିଶୁ ସୁରକ୍ଷା କାର୍ଡ(MCP) ଅଛି କି ?	Yes/ହଁ -------------------------------- 1<br>No/ନା --------------------------------- 0<br>→ *(go to Q 5.6)*<br>Not Available or not up-to-date/ କାର୍ଡ ନାହିଁ/ଲେଖା ହୋଇ ନାହିଁ--------------------2<br>→ *(go to Q 5.6)*	
child_dob_card0	5.4. Date of birth of child on the card/ କାର୍ଡରେ ଥିବା ଶିଶୁର ଜନ୍ମ ତାରିଖ	d d / m m / y y y y	
child_dob_card_y0	5.5. Is the date of the birth on the card correct/କାର୍ଡରେ ଥିବା ଜନ୍ମ ତାରିଖଠିକ ଅଛି କି?	Yes/ହଁ -------------------------------- 1<br>No/ନା --------------------------------- 0	
child_mths0	5.6. What is the age of this child in completed months, to the best of your knowledge/ ଆପଣଙ୍କ ଶିଶୁର ବୟସ କେତେ?<br>(if child is less than one month old then record'0' months)<br>ଯଦିଶିଶୁରବୟସ୧ ମାସରୁକମହୁଏତେବେ "0"ଲେଖନ୍ତୁ।		
child_age_diff0	5.7. If date of birth is available, check the difference between age in months and date of birth. If there is more than 1 month between the age in months of date of birth, go back and verify which is correct, and amend the incorrect answer/ଯଦି ଜନ୍ମ ପ୍ରମାଣ ପତ୍ର ଅଛି ତେବେ ମାସରେ ବୟସ ଓ ଜନ୍ମ ତାରିଖ ମଧ୍ୟରେ ପାର୍ଥକ୍ୟ ଯାଞ୍ଚ କରନ୍ତୁ । ଯଦି ମାସରେ ବୟସ ଓ ଜନ୍ମ ତାରିଖ ମଧ୍ୟରେ ୧ ମାସରୁ ଅଧିକ ଫରକ ହୁଏ ତେବେ ଯାଞ୍ଚ କରନ୍ତୁ କେଉଁଟି ଠିକ ଏବଂ ଭୁଲ ବୟସର ସଂଶୋଧନ କରନ୍ତୁ ।	Date of birth and age in months were correct/ ମାସରେ ବୟସ ଓ ଜନ୍ମ ତାରିଖ ଠିକ ଅଛି---------------------- 1<br>Date of birth and age in months were not correct – amendment was needed/ ମାସରେ ବୟସ ଓ ଜନ୍ମ ତାରିଖ ଭୁଲ ଅଛି-ସଂଶୋଧନର ଆବଶ୍ୟକ ରହିଛି------------- --- ---------------------- 0<br>No date of birth provided/ଜନ୍ମ ତାରିଖ ଦିଆଯାଇନାହିଁ-----------2	
child_eligible0	5.8. Calculate if the child is eligible or not/ହିସାବ କରନ୍ତୁ ଶିଶୁଟି ଯୋଗ୍ୟ କି ନୁହେଁ (Less than 23 months/ ୨୩ ମାସରୁ କମ)	Yes, eligible/ହଁ, ଯୋଗ୍ୟ------------ 1<br>No, not eligible/ନା, ଯୋଗ୍ୟ ନୁହେଁ- 0	
	**Child 2**		
child_name1	5.9. Child name /ଶିଶୁର ନାମ		
card1	5.9.1 Do you have mother and child protection (MCP) card for you and <child_name> ଟିକାକରଣ କାର୍ଡବାମା ଓ ଶିଶୁ ସୁରକ୍ଷା କାର୍ଡ(MCP) ଅଛି କି ?	Yes/ହଁ -------------------------------- 1<br>No/ନା --------------------------------- 0<br>→ *(go to Q 5.9.4)*<br>Not Available or not up-to-date/କାର୍ଡ ନାହିଁ/ଲେଖା ହୋଇ ନାହିଁ-------------2<br>→ *(go to Q 5.9.4)*	
child_dob_card1	5.9.2 Date of birth of child on the card/ କାର୍ଡରେ ଥିବା ଶିଶୁର ଜନ୍ମ ତାରିଖ	d d / m m / y y y y	
child_dob_card_y1	5.9.3 Is the date of the birth on the card correct/କାର୍ଡରେ ଥିବା ଜନ୍ମ ତାରିଖ ସଠିକ କି?	Yes/ହଁ -------------------------------- 1<br>No/ନା --------------------------------- 0	

child_mths1	5.9.4 What is the age of this child in completed months, to the best of your knowledge/ ଆପଣ ଜାଣିବାରେପୂର୍ଣ ମାସରେ ଶିଶୁର ବୟସ କେତେ?<br>(if child is less than one month old then record '0' months) ଯଦିଶିଶୁରବୟସ6 ମାସରୁକମହୁଏତେବେ "0"ଲେଖନ୍ତୁ।		☐☐
child_age_diff1	5.9.5 If date of birth is available, check the difference between age in months and date of birth. If there is more than 1 month between the age in months of date of birth, go back and verify which is correct, and amend the incorrect answer/ଯଦି ଜନ୍ମ ପ୍ରମାଣ ପତ୍ର ଅଛି ତେବେ ମାସରେ ବୟସ ଓ ଜନ୍ମ ତାରିଖ ମଧ୍ୟରେ ପାର୍ଥକ୍ୟ ଯାଞ୍ଚ କରନ୍ତୁ । ଯଦି ମାସରେ ବୟସ ଓ ଜନ୍ମ ତାରିଖ ମଧ୍ୟରେ ୧ ମାସରୁ ଅଧିକ ଫରକ ହୁଏ ତେବେ ଯାଞ୍ଚ କରନ୍ତୁ କେଉଁଟି ଠିକ ଏବଂ ଭୁଲ ବୟସର ସଂଶୋଧନ କରନ୍ତୁ ।	Date of birth and age in months were correct/ ମାସରେ ବୟସ ଓ ଜନ୍ମ ତାରିଖ ଠିକ ଅଛି---------------------- 1<br>Date of birth and age in months were not correct – amendment was needed/ ମାସରେ ବୟସ ଓ ଜନ୍ମ ତାରିଖ ଭୁଲ ଅଛି-ସଂଶୋଧନର ଆବଶ୍ୟକ ରହିଛି---------------- ----------------------- 0<br>No date of birth provided/ଜନ୍ମ ତାରିଖ ଦିଆଯାଇନାହିଁ-----------2	☐
child_eligible1	5.9.6 Calculate if the child is eligible or not/ ହିସାବକରନ୍ତୁଶିଶୁଟିଯୋଗ୍ୟକିନୁହେଁ (Less than 23 months/୨୩ ମାସରୁ କମ)	Yes, eligible/ହଁ, ଯୋଗ୍ୟ------------ 1<br>No, not eligible/ନା, ଯୋଗ୍ୟ ନୁହେଁ- 0	☐
	**Child 3**		
child_name2	5.10 Child name /ଶିଶୁର ନାମ	☐	
card2	5.10.1 Do you have mother and child protection (MCP) card for you and <child_name>/ ଟିକାକରଣ କାର୍ଡବାମା ଓ ଶିଶୁ ସୁରକ୍ଷା କାର୍ଡ (MCP) ଅଛି କି ?	Yes/ହଁ -------------------------------- 1<br>No/ନା --------------------------------- 0<br>→ *(go to Q 5.10.4)*<br>Not Available or not up-to-date/କାର୍ଡ ନାହିଁ/ଲେଖା ହୋଇ ନାହିଁ-------------2<br>→ *(go to Q 5.10.4)*	☐
child_dob_card2	5.10.2 Date of birth of child on the card/ କାର୍ଡରେ ଥିବା ଶିଶୁର ଜନ୍ମ ତାରିଖ	d d / m m / y y y y	
child_dob_card_y2	5.10.3 Is the date of the birth on the card correct/କାର୍ଡରେ ଥିବା ଜନ୍ମ ତାରିଖ ସଠିକ କି?<br>(If card0 ==1)	Yes/ହଁ -------------------------------- 1<br>No/ନା --------------------------------- 0	☐
child_mths2	5.10.4 What is the age of this child in completed months, to the best of your knowledge/ ଆପଣ ଜାଣିବାରେପୁର୍ଣ୍ଣମାସରେ ଶିଶୁର ବୟସ କେତେ?<br>(if child is less than one month old then record '0' months) ଯଦିଶିଶୁରବୟସ6 ମାସରୁକମହୁଏତେବେ "0"ଲେଖନ୍ତୁ।		☐☐

child_age_diff2	5.10.5 If date of birth is available, check the difference between age in months and date of birth. If there is more than 1 month between the age in months of date of birth, go back and verify which is correct, and amend the incorrect answer/ଯଦି ଜନ୍ମ ପ୍ରମାଣ ପତ୍ର ଅଛି ତେବେ ମାସରେ ବୟସ ଓ ଜନ୍ମ ତାରିଖ ମଧ୍ୟରେ ପାର୍ଥକ୍ୟ ଯାଞ୍ଚ କରନ୍ତୁ । ଯଦି ମାସରେ ବୟସ ଓ ଜନ୍ମ ତାରିଖ ମଧ୍ୟରେ ୧ ମାସରୁ ଅଧିକ ଫରକ ହୁଏ ତେବେ ଯାଞ୍ଚ କରନ୍ତୁ କେଉଁଟି ଠିକ ଏବଂ ଭୁଲ ବୟସର ସଂଶୋଧନ କରନ୍ତୁ ।	Date of birth and age in months were correct/ ମାସରେ ବୟସ ଓ ଜନ୍ମ ତାରିଖ ଠିକ ଅଛି---------------------- 1<br>Date of birth and age in months were not correct – amendment was needed/ ମାସରେ ବୟସ ଓ ଜନ୍ମ ତାରିଖ ଭୁଲ ଅଛି-ସଂଶୋଧନର ଆବଶ୍ୟକ ରହିଛି---------------- ---------------------- 0<br>No date of birth provided/ଜନ୍ମ ତାରିଖ ଦିଆଯାଇନାହିଁ-----------2	☐
child_eligible2	5.10.6 Calculate if the child is eligible or not/ହିସାବକରନ୍ତୁଶିଶୁଟିଯୋଗ୍ୟକିନୁହେଁ (Less than 23 months/୨୩ ମାସରୁ କମ)	Yes, eligible---------------------- 1<br>No, not eligible------------------ 0	☐
	**Child 4**		
child_name3	5.11 Child name /ଶିଶୁର ନାମ	☐	
Card3	5.11.1 Do you have mother and child protection (MCP) card for you and this child? /ଆପଣଙ୍କରଟିକ କାରଣ କାର୍ଡ ବା ମା ଏବଂ ଶିଶୁ ସୁରକ୍ଷା କାର୍ଡ (MCP) ଅଛି କି ?	Yes/ହଁ -------------------------------- 1<br>No/ନା --------------------------------- 0<br>→ *(go to Q 5.11.4)*<br>Not Available or not up-to-date/ କାର୍ଡ ନାହିଁ/ଲେଖା ହୋଇ ନାହିଁ-------------------2<br>→ *(go to Q 5.11.4)*	☐
child_dob_card3	5.11.2 Date of birth of child on the card/ କାର୍ଡରେ ଥିବା ଶିଶୁର ଜନ୍ମ ତାରିଖ	d d / m m / y y y y	
child_dob_card_y3	5.11.3 Is the date of the birth on the card correct/କାର୍ଡରେ ଥିବା ଜନ୍ମ ତାରିଖ ସଠିକ କି?	Yes/ହଁ -------------------------------- 1<br>No/ନା -------------------------------- 0	☐
child_mths3	5.11.4 What is the age of this child in completed months, to the best of your knowledge/ଆପଣଙ୍କ ଶିଶୁର ବୟସ କେତେ? (ମାସରେ)<br>(if child is less than one month old then record '0' months) ଯଦିଶିଶୁରବୟସ୧ ମାସରୁକମହୁଏତେବେ "0"ଲେଖନ୍ତୁ।		☐☐
child_age_diff3	5.11.5. If date of birth is available, check the difference between age in months and date of birth. If there is more than 1 month between the age in months of date of birth, go back and verify which is correct, and amend the incorrect answer/ଯଦି ଜନ୍ମ ପ୍ରମାଣ ପତ୍ର ଅଛି ତେବେ ମାସରେ ବୟସ ଓ ଜନ୍ମ ତାରିଖ ମଧ୍ୟରେ ପାର୍ଥକ୍ୟ ଯାଞ୍ଚ କରନ୍ତୁ । ଯଦି ମାସରେ ବୟସ ଓ ଜନ୍ମ ତାରିଖ ମଧ୍ୟରେ ୧ ମାସରୁ ଅଧିକ ଫରକ ହୁଏ ତେବେ ଯାଞ୍ଚ କରନ୍ତୁ କେଉଁଟି ଠିକ ଏବଂ ଭୁଲ ବୟସର ସଂଶୋଧନ କରନ୍ତୁ ।	Date of birth and age in months were correct/ ମାସରେ ବୟସ ଓ ଜନ୍ମ ତାରିଖ ଠିକ ଅଛି---------------------- 1<br>Date of birth and age in months were not correct – amendment was needed/ ମାସରେ ବୟସ ଓ ଜନ୍ମ ତାରିଖ ଭୁଲ ଅଛି-ସଂଶୋଧନର ଆବଶ୍ୟକ ରହିଛି---------------- ---------------------- 0<br>No date of birth provided/ଜନ୍ମ ତାରିଖ ଦିଆଯାଇନାହିଁ-----------2	☐
child_eligible3	5.11.6. Calculate if the child is eligible or not/ହିସାବକରନ୍ତୁଶିଶୁଟିଯୋଗ୍ୟକିନୁହେଁ (Less than 23 months/୨୩ ମାସରୁ କମ)	Yes, eligible/ହଁ, ଯୋଗ୍ୟ----------- 1<br>No, not eligible/ନା, ଯୋଗ୍ୟ ନୁହେଁ- 0	☐
5.12 End the interview if there are no eligible children/ଯଦି ଶିଶୁଟି ଯୋଗ୍ୟ ନୁହେଁ ତେବେ ସାକ୍ଷାତକାର ବନ୍ଦ କରନ୍ତୁ ।			
**Randomly select one eligible child from the household/**ପରିବାରରୁ ଅନିର୍ଦ୍ଦିଷ୍ଟ ଭାବରେ ଗୋଟିଏ ଯୋଗ୍ୟ ଶିଶୁ ଚୟନ କରନ୍ତୁ।<br>**Exclude any children who are >23 months** 23 ମାସରୁ ଅଧିକ ବୟସର ପିଲାଙ୍କୁ ବାଦ ଦିଅନ୍ତୁ			

index_child name	5.13 What is the name of the index child/ଚୟନ ଶିଶୁର ନାମ କଣ?		
Now I will ask you questions about this child/ ବର୍ତ୍ତମାନ ମୁ ଆପଣଙ୍କୁ ଏହି ଶିଶୁ ବିଷୟରେ ପଚାରିବାକୁ ଚାହୁଁଛି।			
child_gender	5.14Is <child_namex>a boy or a girl/ଶିଶୁଟି ପୁଅ ନା ଝିଅ?	Boy/ ପୁଅ ---------------------------- 0<br>Girl/ ଝିଅ ---------------------------- 1	
mother_consented	5.15Is the mother / primary giver the same person as the mother / primary caregiver you have already obtained consent from?<br>ଆପଣ ଏହି ଶିଶୁର ମା ଅଟନ୍ତି କି ?	Yes, the same person ହଁ, ସେହି ଲୋକ ଅଟନ୍ତି କି ------------ 1<br>→ *(go to Q 6.1)*<br>No, someone different ନା, ଅନ୍ୟ କେହି ------------------------- 0<br>→ *(Take consent of the mother/primary caregiver of the index child & go to Q5.14)*	
Ask to interview the mother / primary caregiver of the index child. Provide information sheet and explain the purpose, process, risks and benefits of participating in the survey to the respondent. Take care to be very detailed on confidentiality and the voluntary nature of the study. Consent MUST BE obtained, including on behalf of the index child. One paper copy of the information sheet and signed consent form must be provided to and retained by the household, and another signed copy of the consent forms must be retained with the enumerator and submitted to your supervisor<br>ଶିଶୁର ମା/ପ୍ରାଥମିକ ଯତ୍ନକାରୀଙ୍କୁ ପଚାରନ୍ତୁ । ସୂଚନା ପତ୍ରଟି ତାଙ୍କୁ ପ୍ରଦାନକରନ୍ତୁ ଓ ତାଙ୍କୁ ଏହି ସର୍ଭେର ଉଦ୍ଦେଶ୍ୟ, ପ୍ରକ୍ରିୟା, ବିପଦ ଓ ଲାଭ ବିଷୟରେ ବର୍ଣନା କରନ୍ତୁ । ସବିଶେଷ ବର୍ଣ୍ଣନା କଲାବେଳେ ଏହି ଆନୁଧାନର ଗୋପନୀୟତା ଓ ସ୍ୱେଚ୍ଛାକୃତତାର ଯତ୍ନନେବା ଉଚିତ । ଏହି ସ୍ତରରେ ସମସ୍ତ ଉତ୍ତରଦାତାଙ୍କ ସହମତି ସହିତ ଦୁଇବର୍ଷରୁ କମ ଶିଶୁର ନିମନ୍ତେ ସହମତି ଜରୁରୀ ଅଟେ । ସୂଚନା ପତ୍ର ଓ ସହମତ୍ତି ପତ୍ରର ଅବିକଳ ନକଲ ପରିବାରର ସଦସ୍ୟଙ୍କୁ ପ୍ରଦାନ ଓ ସହମତ୍ତି ପତ୍ରରେ ଦସ୍ତଖତ କରି ସେଥିରୁ ଗୋଟିଏ ନକଲ ପରିବାର ସଭ୍ୟଙ୍କୁ ଜିମାଦେଇ ଅନ୍ୟଟି ସାକ୍ଷତକାରୀ ନିଜ ସୁପରଭାଇଜର ପାଖରେ ଜମା କରିବେ ।			
**Variable name**	**Question**	**Code**	**Answer**
consent_motherofchild	5.16Did the mother/primary caregiver give consent? ମା /ପ୍ରାଥମିକ ଯତ୍ନକାରୀ ସହମତୀ ଦେଲେକି?	Yes, written / thumbprint ହଁ , ଦସ୍ତଖତ/ଟିପଚିହ୍ନ ଦେଲେ --------------- 1<br>Yes, verbal (witnessed by someone else e.g. neighbour or relative) ସାକ୍ଷୀଙ୍କ ଉପସ୍ଥିତିରେ, ଅନ୍ୟ ଜଣଙ୍କ ଦ୍ୱାରା ଯଥା ପଡୋଶୀ ବା ସାଙ୍ଗ ସାଥି) --------------------------- 2<br>No ନା ---------------------------------- 0<br>→ *end the interview*	

6 Background Information of mother / primary caregiver of the index child/ ଶିଶୁର ମା/ପ୍ରାଥମିକ ଯତ୍ନକାରୀଙ୍କ ମୌଳିକ ସୁଚନା

**Variable name**	**Question**	**Code**	**Answer**
**Questions to the mother / primary caregiver of the index child**			
motherofchild_name	6.1 What is the name of the mother or primary caregiver for this child/ ଏହି ଶିଶୁର ମା/ପ୍ରାଥମିକ ଯତ୍ନକାରୀଙ୍କ ନାମ କଣ?		
mother_carer	6.2 Is the primary caregiver the mother or someone else in the household/ପ୍ରାଥମିକ ଯତ୍ନକାରି ନିଜେ ମା ନା ପରିବାରର ଅନ୍ୟ କେହି ?	Mother ମାଆ------------- 0<br>Not the mother (any other female) ମା ନୁହଁ (ଅନ୍ୟ ଜଣେ ମହିଳା)-----------1<br>→ *skip Qs. 7.2 & 7.3*	
mother_age	6.3 How old were you on your last birthday? (Age to be taken in completed years)/ଆପଣଙ୍କ ବୟସ କେତେ?<br><br>(What was your age on last birthday/ *ଶେଷ ଜନ୍ମ ଦିନରେ ଆପଣଙ୍କ ବୟସ କେତେ ଥିଲା ?)*		

Variable name	Question	Code	Answer
mother_married	6.4 Are you married/ଆପଣ ବିବାହିତ କି? (Select the marital status from the list based on the response)<br><br>Cross check for option<br>ଉତ୍ତରକୁ ଆଧାରକରି ସଠିକ ବୈବାହିକ ସ୍ଥିତିକୁ ଚୟନ କରନ୍ତୁ।(ବିକଳ୍ପ ଗୁଡିକୁ ପୁନଃ ଯାଞ୍ଚକରନ୍ତୁ)	<single answer/ଗୋଟିଏ ଉତ୍ତର ଲେଖନ୍ତୁ><br>1=Single/ଏକାକୀ<br>→ *skip Q 6.7*<br>2=Married/ବିବାହିତା<br>3=Divorced/ଛାଡପତ୍ର<br>4=Separated/ଅଲଗା ରହୁଛନ୍ତି<br>5=Widowed/ବିଧବା	☐
mother_preglac	6.5 Are you pregnant, postpartum, or a lactating mother now? / ଆପଣ ବର୍ତ୍ତମାନ ଗର୍ଭବତୀ ଅଛନ୍ତି, ପ୍ରସୁତୀ ମା, କିମ୍ବା ମା କ୍ଷୀର ଖୁଆଉଛନ୍ତି କି?	Select all that apply<br>ସମସ୍ତ ଉତ୍ତର ଉଲେଖ କରନ୍ତୁ<br>☐ Yes, pregnant, ହଁ, ଗର୍ଭବତୀ------------1<br>☐ Yes, postpartum (gave birth less than 6 completed weeks ago) ହଁ, ପ୍ରସୁତୀ ମା (୬ ସପ୍ତାହ ପୁର୍ବରୁ ଜନ୍ମ ଦେଇଛନ୍ତି) ----------2<br>☐ Yes, lactating/ସ୍ତନ୍ୟପାନ କରାଉଅଛି ------3<br>☐ No, not pregnant, postpartum or lactating/ ନା, ଗର୍ଭବତୀନାହିଁ , ପ୍ରସୁତୀ ନାହିଁ ଏବଂ ସ୍ତନ୍ୟପାନ କରାଉନାହିଁ-----------------0	
mother_edulevel	6.6 Years of formal education completed/passed by mother / primary caregiver/ଆପଣ କେତେ ପାଠ ପଢିଛନ୍ତି<br>(Complete education) if respondent has passed 10th year then write 10; and if failed 10th year then write 9. (ସମ୍ପୂର୍ଣ ଶିକ୍ଷା) ଯଦି ଉତ୍ତରଦାତା ଦଶମ ଶ୍ରେଣୀ ପାସ କରିଛନ୍ତି ତେବେ 10 କୋଡ କରନ୍ତୁ ଏବଂ ଯଦି ଦଶମ ଶ୍ରେଣୀରେ ଫେଲ ହୋଇଥାନ୍ତି ତେବେ ୨ କୋଡ କରନ୍ତୁ।		☐
spouse_edulevel	6.7 Years of formal education completed/ passed by spouse/ ଆପଣଙ୍କ ସ୍ୱାମୀ କେତେ ପାଠ ପଢିଛନ୍ତି?		☐
hh_size0	6.8 How many household members live in your household, including yourself? (aged 10 completed years or older)/ ଆପଣଙ୍କୁ ମିଶାଇ ଆପଣଙ୍କ ପରିବାରରେ କେତେଜଣ ସଦସ୍ୟ ରହୁଛନ୍ତି? (10 ବର୍ଷ କିମ୍ବା ତାଠାରୁ ଅଧିକ ବୟସ ହୋଇଥିବା ସଦସ୍ୟ)		☐
hh_size1	6.9 How many children live in your household, including the (child_name)? (aged under 10 completed years)/ ଏହି ଶିଶୁକୁ ମିଶାଇ ଆପଣଙ୍କ ପରିବାରରେ 10 ବର୍ଷ ବୟସରୁ କମ ହୋଇଥିବା କେତେଜଣ ଶିଶୁ ରହୁଛନ୍ତି ।		☐

7 Infant & young child feeding (IYCF) practices

Verify that you are speaking with the correct respondent and about the correct child by/ନିଶ୍ଚିତ କରନ୍ତୁ ଆପଣ ସଠିକ ଉତ୍ତରଦାତାଙ୍କୁ ସଠିକ ଶିଶୁ ବିଷୟରେ ପଚାରୁଛନ୍ତି :

1. Checking that the respondent's name is <mother_name>/ଉତ୍ତରଦାତାଙ୍କ ନାମ ଯାଞ୍ଚ କରନ୍ତୁ<ମା' ଙ୍କ ନାମ>.
2. Checking that the respondent is the mother or primary caregiver of <child_name>/ଏହା ଯାଞ୍ଚ କରନ୍ତୁ ଯେ ଉତ୍ତରଦାତା ହେଉଛନ୍ତି ମା/ପ୍ରାଥମିକ ଯତ୍ନକାରୀ <ଶିଶୁର ନାମ>.

I would now like to ask you some questions about <child_name>/ମୁଁ ବର୍ତ୍ତମାନ ଆପଣଙ୍କୁ ଶିଶୁ ବିଷୟରେ କିଛି ପ୍ରଶ୍ନ ପଚାରିବି <ଶିଶୁର ନାମ>

Variable name	Question	Code	Answer
**Ask all the index children:** ଚୟନହୋଇଥିବାସମସ୍ତଶିଶୁଙ୍କବିଷୟରେପଚାରନ୍ତୁ			

Variable name	Question	Code	Answer
bf_ever	7.1 Was (child_name) ever breastfed<br>ଆପଣ କେବେବି (ଶିଶୁର ନାମ) ଶିଶୁକୁ ମା କ୍ଷୀର ଦେଇଥିଲେ କି ?	Yes/ହଁ ------------------------------ 1<br>No/ନା ------------------------------ 0<br>*→ Go to Q7.6. Also skip 7.7 and 7.16*<br>Don't know/ଜାଣି ନାହାନ୍ତି --------- 98<br>*→ Go to Q7.6. Also skip 7.7 and 7.16*	
bf_initiate	**Do not ask if caregiver is not the mother**<br>7.2 Think back to the birth of child (child_name).<br>ଜନ୍ମ ହେବାର କେତେ ସମୟପରେ ଶିଶୁକୁ ପ୍ରଥମେ ମାଆ କ୍ଷୀର ଦିଆଯାଇଥିଲା?<br>How long after birth did you first put (child_name) to the breast? It does not matter whether or not the mother's milk had arrived at the time she gave the child the breast.<br>ଜନ୍ମ ହେବାର କେତେ ସମୟ ବ୍ୟବଧାନରେ ଆପଣ ଶିଶୁକୁ(ଶିଶୁର ନାମ)ପ୍ରଥମଥରମା କ୍ଷୀରଖୁଆଇଥିଲେ ?ଏହା ଆବଶ୍ୟକ ନୁହଁ ଯେ ସେହି ସମୟ ରେ ମା ରକ୍ଷୀର ଆସୁଥାଉ କି ନଆସୁଥାଉ କିନ୍ତୁ ସେ ସେହି ସମୟ ରେ କ୍ଷୀର ଖୁଆଇ ଥିଲେ କି? ।	<single answer/ଗୋଟିଏ ଉତ୍ତର ଲେଖନ୍ତୁ><br>Immediately/ତୁରନ୍ତ--------------- 0<br>Less than 1 hour/ଏକ ଘଣ୍ଟାରୁ କମ ବ୍ୟବଧାନରେ ------------------------ 1<br>Between 1 and 24 hours/ଏକ ଘଣ୍ଟାରୁ ୨୪ ଘଣ୍ଟା ମଧ୍ୟରେ------------------ 2<br>After one day up to 3 days/ଗୋଟିଏ ଦିନପରେ ୩ଦିନ ମଧ୍ୟରେ------------ 3<br>After 3 days/୩ ଦିନ ପରେ------- 4<br>Can't remember / don't know /ମନେ ପଡୁନାହିଁ/ଜଣା ନାହିଁ--------- 98	
bf_colostrum	**Do not ask if caregiver is not the mother**<br>7.3 Did you give the colostrum (the thick yellow milk from the breast) to (child_name) in the first 3 days?<br>ଆପଣ ପ୍ରଥମ ୩ଦିନ ମଧ୍ୟରେ ଶିଶୁକୁ କଷ କ୍ଷୀର (ସ୍ତନରୁ ଆସୁଥିବା ଗାଢ ହଳଦିଆ କ୍ଷୀର) (ଶିଶୁର ନାମ)ଦେଇଥିଲେ କି ?	Yes/ହଁ ------------------------------ 1<br>No/ନା ------------------------------ 0<br>Don't know/ଜାଣି ନାହାନ୍ତି --------- 98	

Next I would like to ask you about how (child_name) was fed in the last 24 hours, during the day or at night/ବର୍ତ୍ତମାନ ମୁଁ ଆପଣଙ୍କୁ ପଚାରିବାକୁ ଚାହୁଁଛି ଯେ ଗତ 24 ଘଣ୍ଟା ମଧ୍ୟରେଶିଶୁକୁ କଣ କଣ ଖାଇବାକୁ ଦିଆଯାଇଥିଲା(ଦିନ ହେଉ କିମ୍ବା ରାତିରେ ହେଉ), (ଶିଶୁର ନାମ) ।ନୋଟ-ଗତକାଲି ସକାଳ 6 ଠାରୁ ଆରମ୍ଭ କରି ଆଜି ସକାଳ ପର୍ଯ୍ୟନ୍ତ)

Variable name	Question	Code	Answer
bf_24	7.4 Was (child_name) breastfed in the last 24 hours, during the day or at night?ଗତ ୨୪ଘଣ୍ଟା ମଧ୍ୟରେ ଶିଶୁକୁ(ଶିଶୁର ନାମ)ମା କ୍ଷୀର ଦେଇଥିଲେ କି ? (ଦିନରେ ହେଉ କିମ୍ବା ରାତି ହେଉ)	Yes/ହଁ ---------------------------- 1<br>No/ନା ---------------------------- 0<br>*→ If no, go to Q7.6*	
bf_24times	7.5 How many times was (child_name) breastfed in the last 24 hours, during the day or at night?<br>ଗତ ୨୪ଘଣ୍ଟା ମଧ୍ୟରେ ଶିଶୁକୁ(ଶିଶୁର ନାମ) କେତେଥର ମା କ୍ଷୀର ଦିଆଯାଇଥିଲା?(ଦିନରେ ହେଉ କିମ୍ବା ରାତି ହେଉ)		
bf_bottle	7.6 Did (child_name) drink anything from a bottle with a nipple/teat yesterday during the day or night?<br>ଗତକାଲି ଶିଶୁଟି(ଶିଶୁର ନାମ) ବୋତଲରୁ କିଛି ପିଇଥିଲା କି **?**(ଦିନରେ ହେଉ କିମ୍ବା ରାତି ହେଉ)	<single answer/ଗୋଟିଏ ଉତ୍ତର ଲେଖନ୍ତୁ><br>Yes/ହଁ ------------------------------ 1<br>No/ନା ------------------------------ 0<br>Don't know/ଜାଣି ନାହାନ୍ତି -------- 98	

Variable name	Question	Code	Answer
bf_liquids_ever	**Do not ask if child has never been breastfed**<br>7.7 Has the (child_name) ever been given anything other than breast milk to eat or drink, including water?<br>ମା କ୍ଷୀର ଛଡା ଶିଶୁଟିକୁ (ଶିଶୁର ନାମ)ଆଉ କିଛି ଖାଇବାକୁ ବା ପିଇବାକୁ(ଜଳ ମଧ୍ୟ) ଦିଆଯାଉଛି କି ?	<single answer/ଗୋଟିଏ ଉତ୍ତର ଲେଖନ୍ତୁ><br><br>Yes/ହଁ -------------------------- 1<br>No/ନା -------------------------- 0<br>Don't know/ଜାଣି ନାହାନ୍ତି ------- 98	☐
bf_liquids_24	7.8 Did <child_name> have any of the following in the last 24 hours?<br>ଗତ ୨୪ ଘଣ୍ଟା ମଧ୍ୟରେ ନିମ୍ନଲିଖିତ ମଧ୍ୟରୁ କିଛି ଦିଆଯାଇଥିଲା କି ?<br><br>*Read the list one by one/*ତାଲିକାରୁ ଗୋଟିଏ ପରେ ଗୋଟିରେ ପଢନ୍ତୁ	<multiple answer/ବହୁ ଉତ୍ତର ସମ୍ଭବ> Tick all that apply/ଉପଯୁକ୍ତ ଉତ୍ତରରେ ଚିହ୍ନ ଦିଅନ୍ତୁ<br>☐ Nothing other than breast milk/ମା କ୍ଷୀର ଛଡା ଅନ୍ୟ କିଛି ନାହିଁ = A<br>☐ Plain water/ସାଧାପାଣି = B<br>☐ Infant formula such as Cerelac, lactogen and lactodex/ଡବା କ୍ଷୀର ଯେପରିକି ସେରେଲାକ, ଲାକ୍ଟୋଜେନ, ଲାକ୍ଟୋଡେକ୍ସ = C<br>*→ ask Q. 7.9*<br>☐ Non- breast milk such as tinned, powdered, or fresh animal milk /ସ୍ତନ୍ୟପାନ ଛଡା ଯେପରିକି ଗୁଣ୍ଡକ୍ଷୀର, ତାଜା ପଶୁ କ୍ଷୀର = D<br>*→ ask Q. 7.10*<br>☐ Juice or juice drinks/ଫଳ ରସ = E<br>☐ Clear broth/ସୁପ = F<br>☐ Yogurt / curd/ଦହି = G<br>*→ ask Q. 7.11*<br>☐ Thin porridge/ପତଳା ଡାଲି = H<br>☐ Any other liquids such as Tea, Honey, Herbal concoctions, *maad paani*/ଅନ୍ୟାନ୍ୟ ପାନୀୟ ଦ୍ରବ୍ୟ ଯେପରିକି ଚା, ମହୁ, ଚେରମୂଳି, ମଦ ପାଣି = I<br>☐ ORS and vitamin and/or mineral supplements/ଓଂଆରଂଏସ, ଭିଟାମିନ ଓ ଅନୁପୂରକ ଖାଦ୍ୟ = J<br>☐ Any other liquids/ଅନ୍ୟାନ୍ୟ ତରଳ ଦ୍ରବ୍ୟ = K	
bf_formula_freq	7.9 How many times yesterday during the day or at night did (child_name) consume any formula?<br>ଗତକାଲିଶିଶୁକୁ କେତେଥର କୌଣସି ଡବା ଖାଦ୍ୟ ଯେପରି ସେରେଲାକ ଦିଅଯାଇଥିଲା?(ଦିନହେଉ ବା ରାତିହେଉ)		☐
bf_milk_freq	7.10 How many times yesterday during the day or at night did (child_name) consume any non- breast milk (like tinned, powdered, or fresh animal milk)?<br>ଗତକାଲିଶିଶୁ ମା" କ୍ଷୀର ଛଡାଅନ୍ୟାନ୍ୟ ଯେପରିକି ଡବାକ୍ଷୀରଗୁଣ୍ଡକ୍ଷୀର, ତାଜାପଶୁକ୍ଷୀର ଖାଇଥିଲା କି ?		☐
bf_curd_freq	7.11 How many times yesterday during the day or at night did (child_name) consume any yoghurt or curd?<br>ଗତକାଲି(ଶିଶୁର ନାମ)କେତେଥରଘୋଳ ଦହି ବା ଦହି ଖାଇଥିଲା?(ଦିନରେ ହେଉ କିମ୍ବା ରାତିହେଉ)		☐

Variable name	Question	Code	Answer
cf	7.12 Have you started regularly/daily feeding your child anything other than breastmilk?<br>ଆପଣଶିଶୁକୁ ମା" କ୍ଷୀର ବ୍ୟତିତ ନିୟମିତ ଭାବେ ଆନ୍ୟକିଛି ଖୁଆଇବା ଆରମ୍ଭ କରିଛନ୍ତି କି?	Yes/ହଁ ----------------------------- 1<br>No/ନା ----------------------------- 0	☐
cf_any	7.13 Did <child_name> eat any solid, semi-solid, or soft foods (e.g. mashed potato, banana, kichidi, sattu, biscuits, peetha, kheer) yesterday during the day or at night?<br>ଗତକାଲି ଶିଶୁଟି (ଶିଶୁର ନାମ) କୌଣସି ବହଳିଆ ,ଟାଣ ଖାଦ୍ୟ,କିମ୍ବା ନରମ ଖାଦ୍ୟ (ଯଥା ଆଳୁ ଚକଟା, କଦଳୀ, ଖେଚୁଡି, ଛତୁଆ, ବିସ୍କୁଟ, ପିଠା, ଖିରୀ ଇତ୍ୟାଦି) ଖାଇଥିଲା କି ?(ଦିନରେ ହେଉ କିମ୍ବା ରାତି ହେଉ)	Yes/ହଁ -------------------------- 1<br>No/ନା -------------------------- 0<br>→ *If no, go to Q. 7.15*	☐
cf_times	**Ask if child is 6 months or older**<br>7.14 How many times did your child receive soft, semi-solid or solid foods (e.g. mashed potato, banana, kichidi, sattu, biscuits, peetha, kheer) other than liquids in the last 24 hours?<br>ଗତ 24 ଘଣ୍ଟା ମଧ୍ୟରେଶିଶୁ(ଶିଶୁର ନାମ) ଆଳୁ ଚକଟା, କଦଳୀ, ଖେଚୁଡି, ଛତୁଆ, ବିସ୍କୁଟ, ପିଠା, ଖିରୀ ଇତ୍ୟାଦିକେତେଥର ଖାଇଥିଲା?<br>Do not count small snacks, e.g. 1-2 bites from mothers' or siblings' food).<br>ଶିଶୁଟି ଅଳ୍ପକିଛି ଖାଉଥିବା ଖାଦ୍ୟକୁ ହିସାବରେ ନିଅନ୍ତୁ ନାହିଁ ଯଥା ମା କିମ୍ବା ଭାଇ ଭଉଣୀଙ୍କଠାରୁ ଗୁଣ୍ଡେ ଦୁଇ ଗୁଣ୍ଡା ଖାଇବା ।		☐
cf_age	**Ask if child is 6 months or older**<br>7.15 At which month did you start regularly/daily feeding your child anything other than breastmilk? (completed months)<br>/ଶିଶୁକୁ କେତେ ମାସରେ (ସମ୍ପୂର୍ଣ ମାସ)ଆପଣ ମା" କ୍ଷୀରଛଡା ଅନ୍ୟ କିଛି ଖୁଆଇବା ଆରମ୍ଭ କଲେ ?		☐
bf_frequency_24	**Ask if child is less than 6 months and the child has ever been breastfed**<br>7.16 How do you know it is time to breastfeed your child (if you are breastfeeding your child)? ଶିଶୁକୁ ମାଆ କ୍ଷୀର ଖୁଆଇବା ସମୟ ହେଲା ବୋଲି ଆପଣ କିପରି ଜାଣନ୍ତି ?<br>*Don't read the list/*ତାଲିକା ପଢନ୍ତୁ ନାହିଁ	<single answer/ଗୋଟିଏ ଉତ୍ତର ଲେଖନ୍ତୁ><br>Whenever the baby is hungry/Criesଯେତେବେଳେ ଶିଶୁକୁ ଭୋକ ହୁଏ/ଯେତେବେଳେ ଶିଶୁ କାନ୍ଦେ --------1<br>Whenever I am free/ଯେତେବେଳେ ମୋତେ ସମୟ/ଫୁରୁସତ ମିଳେ-----------2<br>At certain time intervals/କିଛି ସମୟର ବ୍ୟବଧାନରେ ------------------3<br>Other/ଅନ୍ୟାନ୍ୟ, ଦର୍ଶାଅ-------------- 4<br>Child not being breastfed/ଶିଶୁକୁ ସ୍ତନ୍ୟପାନ କରାଯାଉନାହିଁ------ ---------5	☐

8 CHILD DIETARY DIVERSITY/ଶିଶୁ ଖାଦ୍ୟର ବିଭିନ୍ନତା

Only ask children over 6 months/କେବଳ ୬ ମାସରୁ ଉର୍ଦ୍ଧ ଶିଶୁଙ୍କ ପାଇଁ ପଚାରନ୍ତୁ

I would like you to describe everything that (child_name) ate yesterday either during the day or at night, whether at home or outside the home.

ବର୍ତ୍ତମାନ ମୁଁ ଆପଣଙ୍କୁ ବିଶେଷ କରି ଗତକାଲି ଦିନରେ ବା ରାତିରେ ଘରେ ହେଉ ବା ବାହାରେ ହେଉ ଶିଶୁ (ଶିଶୁର ନାମ) କଣ କଣ ଖାଇଥିଲା ଓ ପିଇଥିଲା ସେ ସମସ୍ତ ବିଷୟ ବର୍ଣନା କରିବାକୁ କହିବି।

Think about when (child_name) first woke up yesterday. Did (child_name) eat anything at that time?
IF YES: Please tell me everything (child_name) ate at that time.
PROBE: Anything else? Until respondent says nothing else
Did (child_name) eat anything from when it woke up in the morning till midday?
Did (child_name) have anything in the afternoon?
Did (child_name) eat anything from afternoon onwards till the evening?
Did (child_name) have anything in the evening?
Did (child_name) eat anything at night? Repeat probes until respondent says nothing else (child went to sleep until the next day)
Did (child_name) have any other solid, semi-solid foods or soft foods not already mentioned, e.g. mashed potato, banana, kichidi, sattu, biscuits, peetha, kheer?
Did (child_name) have any other foods eaten outside of the home?
Did (child_name) have any other foods that were foraged / wild foods?
Did (child_name) drink anything in the last 24 hours?

ମନେ ପକାନ୍ତୁ ଗତକାଲି ଶିଶୁ (ଶିଶୁର ନାମ) ଯେତେବେଳେ ନିଦରୁ ଉଠିଲାସେହି ସମୟରେ କିଛି ଖାଇଥିଲା କି ?
ଯଦି ହଁ ଦୟାକରି ମୋତେ କୁହନ୍ତୁ ଶିଶୁ ସେହି ସମୟରେ କଣ ସବୁ ଖାଇଲା?
ଦର୍ଶାନ୍ତୁ: ଆଉ କିଛି ? ଯେ ପର୍ଯ୍ୟନ୍ତ ଉତ୍ତରଦାତା "ଆଉ କିଛି ନାହିଁ " କହିଲେ
ଶିଶୁ (ଶିଶୁର ନାମ)ସକାଳେ ଯେତେବେଳେ ନିଦରୁ ଉଠିଲାଠାରୁ ମଧ୍ୟାନ୍ନ ପର୍ଯ୍ୟନ୍ତ କିଛି ଖାଇଥିଲା କି ?
ଶିଶୁ (ଶିଶୁର ନାମ)ଉପରବେଳା କିଛି ଖାଇଥିଲା କି ?
ଶିଶୁ (ଶିଶୁର ନାମ)ଅପରାହ୍ନରୁ ସନ୍ଧ୍ୟା ପର୍ଯ୍ୟନ୍ତ କିଛି ଖାଇଥିଲା କି ?
ଶିଶୁ (ଶିଶୁର ନାମ)ସନ୍ଧ୍ୟାରେ କିଛି ଖାଇଥିଲା କି ?
ଶିଶୁ (ଶିଶୁର ନାମ)ରାତିରେ କିଛି ଖାଇଥିଲା କି ? "ଆଉ କିଛି ନାହିଁ " କହିବା ପର୍ଯ୍ୟନ୍ତ ପଚାରନ୍ତୁ (ଶିଶୁଟି ବିଛଣା କୁ ଶୋଇବାକୁ ଯିବା ପର୍ଯ୍ୟନ୍ତ)
ଶିଶୁ (ଶିଶୁର ନାମ) ଅନ୍ୟ କିଛି କଠିନ, ଅର୍ଦ୍ଧ କଠିନ କିମ୍ବା ନରମ ଖାଦ୍ୟ ଖାଇ ଥିଲା କି ? ତଥା ଯାହା ଆଗରୁ ଉଲ୍ଲେଖ କରିନାହାନ୍ତି ଯଥା: ଆଳୁ ଚକଟା,କଦଳୀ,ଖେଚୁଡି, ଛତୁଆ, ବିସ୍କୁଟ, ପିଠା, ଖିରୀ
ଶିଶୁ (ଶିଶୁର ନାମ)ଶିଶୁଟି ବାହାରେ କିଛି ଖାଦ୍ୟ ଖାଇଥିଲା କି ?
ଶିଶୁ (ଶିଶୁର ନାମ)କିଛି ଜଙ୍ଗଲୀ ଖାଦ୍ୟ ଖାଇଥିଲା କି?
ଶିଶୁ (ଶିଶୁର ନାମ)ଗତ ୨୪ ଘଣ୍ଟା ମଧ୍ୟରେ କିଛି ପାନୀୟ ପିଇଥିଲା କି ?

IF RESPONDENT MENTIONS MIXED DISHES LIKE A PORRIDGE, SAUCE OR STEW, PROBE:
ଯଦି ଉତ୍ତରଦାତା ମିଶ୍ରିତ ଖାଦ୍ୟ ଉଲ୍ଲେଖ କରନ୍ତି ଦର୍ଶାନ୍ତୁ:ଯଥା ଛତୁଆ,ସିଝା,ମଣ୍ଡ,ଇତ୍ୟାଦି
What ingredients were in that (MIXED DISH)/ମିଶ୍ରିତ ଖାଦ୍ୟରେ କଣ କଣ ଦ୍ରବ୍ୟ ଥିଲା?
PROBE: Anything else? UNTIL RESPONDENT SAYS NOTHING ELSE.
ଦର୍ଶାନ୍ତୁ: ଆଉ କିଛି ? ଯେ ପର୍ଯ୍ୟନ୍ତ ଉତ୍ତରଦାତା "ଆଉ କିଛି ନାହିଁ " କହିଲେ

*** Ask about any food groups that were not reported in the free recall ***
ଖାଦ୍ୟ ମନେ ପକାଇଲେ ବେଳେ ଛାଡି ଯାଇଥିବା ଖାଦ୍ୟ ଗୁଡିକ ଆଉଥରେ ପଚାରିଲେ

No.	Answers (space for writing recalled information)	Food group
1.		
2.		
3.		
4.		
5.		
6.		
7.		
8.		
9.		
10.		
11.		
12.		
13.		
14.		
15.		
16.		
17.		
18.		
19.		
20		
21.		
22.		
23.		

Variable name	Question	Code	Answer
	**Using the recalled information, record whether <child_name> had any of the following food groups.**<br>ନିମ୍ନଲିଖିତ ଖାଦ୍ୟ ତାଲିକାରେ ଉତ୍ତରଦାତା ମନେପକାଇ କହୁଥିବା ଶିଶୁର ଖାଦ୍ୟ ଅନ୍ତର୍ଭୁକ୍ତ କି		
dds_fast_child	Was yesterday a special day, like a celebration or feast day or a fast day where the child ate special foods or more or less than usual or did not eat because of fasting/ଗତକାଲି କିଛି ବିଶେଷ ଦିନ ଥିଲା କି, ଯେପରି କୌଣସି ଉତ୍ସବ,ଭୋଜି କିମ୍ବା ଉପବାସର ଦିନ ଥିଲା କି ଯେଉଁଥିରେ ଶିଶୁ କିଛି ବିଶେଷ, ଅଧିକ ଖାଦ୍ୟ ଖାଇଥିଲେ କିମ୍ବା ଉପବାସ ଯୋଗୁଁ ଖାଇ ନ ଥିଲେ ?	No/ନା------------------------------------0<br>Yes, celebration feast day/ହଁ,ଉତ୍ସବ ପାଳନ ପର୍ବ - 1<br>Yes, fast day/ହଁ, ଉପବାସର ଦିନ ------------- 2	
dds_child_1	8.1 Roti, rice (any type including puffed or beaten), peetha, pastry (e.g. grain used in singada), noodles, Chowmein, powdered grain mixture, other food made from grains<br>ରୁଟି, ଭାତ, ମୁଢି, ଚୂଡା, ପିଠା, ସିଙ୍ଗଡା, ନୁଡୁଲ୍ସ, ଚାଓମିନ, ଛତୁଆ, ଅନ୍ୟାନ୍ୟ ଶସ୍ୟରୁ ତିଆରି ହୋଇଥିବା ଗୁଣ୍ଡ ଇତ୍ୟାଦି	Yes/ହଁ -- 1<br>No/ନା --- 0<br>Don't know/ଜାଣି ନାହାନ୍ତି ---------------------- 98	
dds_child_2	8.2 Any foods made with dal, beans, dried / mature peas (including besan / gram flour), soyabean,<br>କିଛିଖାଦ୍ୟଯାହାଡାଲି,ବିନ୍ସ,ମଟର,ବେସନ,ସୋୟାବିନ ଇତ୍ୟାଦିରୁତିଆରି?	Yes/ହଁ -- 1<br>No/ନା --- 0<br>Don't know/ଜାଣି ନାହାନ୍ତି ---------------------- 98	
dds_child_3	Yellow pumpkin, carrot, sweet potato (orange/yellow inside) /ବୋଇତି କଖାରୁ, ଗାଜର, କନ୍ଦମୂଳ, କମଳା ଓ ହଳଦିଆ ଅଂଶଥିବା ପରିବା	Yes/ହଁ -- 1<br>No/ନା --- 0<br>Don't know/ଜାଣି ନାହାନ୍ତି ---------------------- 98	
dds_child_4	8.3 White potatoes, white yams, elephant foot, aluwa, taro, or any other starchy roots<br>ଆଳୁ,ଖମ୍ବଆଳୁ,ମାଟିଆଳୁ,ସାରୁ,ବହଳିଆମଣ୍ଡହେଉଥିବାପରିବା	Yes/ହଁ -- 1<br>No/ନା --- 0<br>Don't know/ଜାଣି ନାହାନ୍ତି ---------------------- 98	
dds_child_5	8.4 Dark green leafy vegetables/ସବୁଜ ପନିପରିବା	Yes/ହଁ -- 1<br>No/ନା --- 0<br>Don't know/ଜାଣି ନାହାନ୍ତି ---------------------- 98	
dds_child_6	8.5 Ripe mango, ripe papaya, ripe jackfruit/ ପାଚିଲା ଆମ୍ବ, ପାଚିଲା ଅମୃତଭଣ୍ଡା, ପାଚିଲା ପଣସ	Yes/ହଁ -- 1<br>No/ନା --- 0<br>Don't know/ଜାଣି ନାହାନ୍ତି ---------------------- 98	
dds_child_7	8.6 Unripe mango, unripe papaya, unripe jackfruit / କଞ୍ଚା ଆମ୍ବ, କଞ୍ଚା ଅମୃତଭଣ୍ଡା, କଞ୍ଚା ପଣସ	Yes/ହଁ -- 1<br>No/ନା --- 0<br>Don't know/ଜାଣି ନାହାନ୍ତି ---------------------- 98	
dds_child_8	8.7 Any other fruits or vegetables/ ଅନ୍ୟ କୌଣସି ଫଳମୂଳ କିମ୍ବା ପନିପରିବା	Yes/ହଁ -- 1<br>No/ନା --- 0<br>Don't know/ଜାଣି ନାହାନ୍ତି ---------------------- 98	

Variable name	Question	Code	Answer
dds_child_9	8.8 Liver, kidney, heart, or other organ meats/କଲିଜା,ହୃଦୟ, ବୃକକ, ମାଂସର ଅନ୍ୟାନ୍ୟ ଅଂଶ	Yes/ହଁ -- 1<br>No/ନା -- 0<br>Don't know/ଜାଣି ନାହାନ୍ତି --------------------- 98	☐
dds_child_10	8.9 Any meat, such as chicken, duck or other birds, pork, lamb, goat, buffalo, rabbit, or mouse / rat/କୌଣସି ମାଂସ ଯେପରିକି କୁକୁଡା, ବତକ, ଅନ୍ୟାନ୍ୟ ପକ୍ଷୀ, ଘୁଷୁରି, ମେଣ୍ଢା, ଛେଳି, ମହିଷୀ, ଠେକୁଆ, ମୂଷା ଇତ୍ୟାଦି	Yes/ହଁ -- 1<br>No/ନା -- 0<br>Don't know/ଜାଣି ନାହାନ୍ତି --------------------- 98	☐
dds_child_11	8.10Egg (e.g. chicken egg or duck egg) /ଅଣ୍ଡା(କୁକୁଡା ବା ବତକ)	Yes/ହଁ -- 1<br>No/ନା -- 0<br>Don't know/ଜାଣି ନାହାନ୍ତି --------------------- 98	☐
dds_child_12	8.11Fresh or dried fish, shellfish, or seafood / ମାଛ ବା ଶୁଖୁଆ, ଶାମୁକା ଓ ଅନ୍ୟାନ୍ୟ ସମୁଦ୍ର ଜାତ ଆମିଷ	Yes/ହଁ -- 1<br>No/ନା -- 0<br>Don't know/ଜାଣି ନାହାନ୍ତି --------------------- 98	☐
dds_child_13	8.12Grubs, snails or insects / ଗେଣ୍ଡା, ଶାମୁକା ବା ଅନ୍ୟାନ୍ୟ ପୋକ	Yes/ହଁ -- 1<br>No/ନା -- 0<br>Don't know/ଜାଣି ନାହାନ୍ତି --------------------- 98	☐
dds_child_14	8.13Nuts or seeds e.g. peanut, cashew, sunflower, Dori, Mahua, Kudrum, Tisi, Ramtia, Linseed, Sesame, Chahar, mustard, mahua , or any foods made with these<br>ବିଭିନ୍ନ ପ୍ରକାର ମଞ୍ଜି ଯଥା ବାଦାମ, କାଜୁ, ସୂର୍ଯ୍ୟମୁଖୀ, ମହୁଲ,ତିଳ,ଅଳସି, ସୋରିଷ,ଚହର,ମହୁଆ ଇତ୍ୟାଦିରୁ ତିଆରି	Yes/ହଁ -- 1<br>No/ନା -- 0<br>Don't know/ଜାଣି ନାହାନ୍ତି --------------------- 98	☐
dds_child_15	8.14Paneer or other cheese, yogurt / curd, any kind of animal milk, or other milk products<br>ଛେନା ଜାତୀୟ ଖାଦ୍ୟ, ଦହି, କ୍ଷୀର ଜାତୀୟ ଦ୍ରବ୍ୟ (ଘିଅ, ଲହୁଣୀ ଓ ଆଇସକ୍ରିମକୁ ଛାଡି)	Yes/ହଁ -- 1<br>No/ନା -- 0<br>Don't know/ଜାଣି ନାହାନ୍ତି --------------------- 98	☐
dds_child_16	8.15Any food with oil, ghee, fat or butter, or foods made with any of these<br>ଘିଅ ବା ତେଲରେ ପ୍ରସ୍ତୁତ ହୋଇଥିବା ଖାଦ୍ୟ	Yes/ହଁ -- 1<br>No/ନା -- 0<br>Don't know/ଜାଣି ନାହାନ୍ତି --------------------- 98	☐
dds_child_17	8.16Sugary foods (chocolates, Chena poda, sweets, candies, cake, biscuits), e.g. jilli / jilebi, laddu<br>ମିଠା ଜାତୀୟ ଖାଦ (ଚକୋଲେଟ,ଛେନା ପୋଡ,କେକ,ଛେନା ଝିଲି,ଜିଲାପି,ଲଡୁ,ବିସ୍କୁଟ)	Yes/ହଁ -- 1<br>No/ନା -- 0<br>Don't know/ଜାଣି ନାହାନ୍ତି ---------------------98	☐

9 CHILD HEALTHCARE/ଶିଶୁ ସ୍ୱାସ୍ଥ୍ୟର ଯତ୍ନ

Variable name	Question	Code	Answer
care_chw_child	9.1 In the past 6 months, did <index child> receive any of the following health care from anganwadis, ASHA or ANM?<br><br>*Read out all of the options.*<br>ଗତ ୬ମାସ ମଧ୍ୟରେ ଶିଶୁଟି (ଶିଶୁର ନାମ) ଆଶା ବା ଏ.ଏନ.ଏମଙ୍କ ଠାରୁଏହିସବୁ ସେବା ପାଇଥିଲା କି ?<br>ସମସ୍ତ ବିକଳ୍ପ ପଢନ୍ତୁ	<multiple answer/ଏକାଧିକ ଉତ୍ତର ସମ୍ଭବ><br>Tick all that apply/ଉପଯୁକ୍ତ ଉତ୍ତର ଗୁଡିକରେ ଚିହ୍ନ ଦିଅନ୍ତୁ<br>☐ Vitamin A supplement/ଭିଟାମିନ ଏ = A<br>☐ Iron supplement for the child/ଆଇରନ ବଟିକା = B<br>☐ Take Home Ration/ଛତୁଆ = C<br>☐ Multiple micronutrient powder (sprinkles)/ = D<br>☐ Deworming medicine/କୃମି ପାଇଁ ଔଷଧ = E<br>☐ Immunisation/vaccinationପ୍ରତିଷେଧକ/ଟୀକାକରଣ = F<br>☐ Referral for (child_name) if SAM/ଶିଶୁ ଦୁର୍ବଳ ହୋଇଥିଲେ ଡାକ୍ତରଖାନାକୁ ପଠାଇଥାନ୍ତି = G<br>☐ None/କୌଣସିଟି ନୁହେଁ = X	
care_institution_child	9.2 In the past 6 months, did <index child> receive any of the following health care from the community health centre, primary health centre, or district hospital?<br>*Read out all of the options.*<br><br>ଗତ ୬ମାସ ମଧ୍ୟରେ ଶିଶୁଟି **(ଶିଶୁର ନାମ) ଆପଣଙ୍କ ଅଞ୍ଚଳରେ ଥିବା ଗୋଷ୍ଠୀ ସ୍ୱାସ୍ଥ୍ୟ କେନ୍ଦ୍ର ବା ପ୍ରାଥମିକ ସ୍ୱାସ୍ଥ୍ୟ କେନ୍ଦ୍ର ରୁ ବା ଜିଲ୍ଲା ମୁଖ୍ୟ ଡାକ୍ତରଖାନାରୁ ଏହିସବୁ ସେବାପାଇଥିଲେ କି** *?*<br>(ସବୁ ବିକଳ୍ପ ପଢନ୍ତୁ)	<multiple answer/ଏକାଧିକ ଉତ୍ତର ସମ୍ଭବ><br>Tick all that apply/ ଉପଯୁକ୍ତ ଉତ୍ତର ଗୁଡିକରେ ଚିହ୍ନ ଦିଅନ୍ତୁ<br>☐ Vitamin A supplement/ଭିଟାମିନ ଏ = A<br>☐ Iron supplement for the child/ଆଇରନ ବଟିକା = B<br>☐ Take Home Ration/ଛତୁଆ = C<br>☐ Multiple micronutrient powder (sprinkles)/ = D<br>☐ Deworming medicine/କୃମି ପାଇଁ ଔଷଧ = E<br>☐ Immunisation/vaccination/ପ୍ରତିଷେଧକ/ଟୀକାକରଣ = F<br>☐ Treatment/therapeutic foods of SAM child ଦୁର୍ବଳ ଶିଶୁ ପାଇଁ ଚିକିତ୍ସା ଓ ଖାଦ୍ୟ = G<br>☐ Treatment/medicines for other illnesses/ଅସୁସ୍ଥତା ପାଇଁ ଚିକିତ୍ସା/ଔଷଧ = H<br>☐ Referral services/ଶିଶୁ ଦୁର୍ବଳ ହେଲେ ଡାକ୍ତରଖାନା ପଠାଇଥାନ୍ତି = I<br>☐ None/କୌଣସିଟି ନୁହେଁ = X	

Variable name	Question	Code	Answer
child_ill	9.3 Has (child_name) had any of the following illnesses in the last 2 weeks (read aloud all options)<br>ଗତ ୨ ସପ୍ତାହ ମଧ୍ୟରେ ଶିଶୁଟିର ଏହି ଭିତରୁ କିଛି ଦେହ ଖରାପ ଥିଲା କି ?<br>(ସବୁ ବିକଳ୍ପ ପଢନ୍ତୁ)	<multiple answer/ଏକାଧିକ ଉତ୍ତର ସମ୍ଭବ><br>Tick all that apply/ ଉପଯୁକ୍ତ ଉତ୍ତର ଗୁଡିକରେ ଚିହ୍ନ ଦିଅନ୍ତୁ<br>☐ Diarrhoea /*julab* (more than 3 watery stools in a day/loose motion)/ଡାଇରିଆ (୩ ଥରରୁ ଅଧିକ ପତଳା ଝାଡା) = A<br>☐ Fever/ଜ୍ୱର = B<br>☐ Cough/କଫ = C<br>☐ Breathing problems: faster than usual with short, rapid breaths or difficulty breathing/ନିଶ୍ୱାସନେବାରେ ଅସୁବିଧା (ସାଧାରଣ ନିଶ୍ୱାସ ପ୍ରଶାସ କମ ନେବା,କଷ୍ଟ ଅନୁଭବ କରିବା) = D<br>☐ Other illness, e.g. common cold/ଅନ୍ୟାନ୍ୟ ଅସୁସ୍ଥତା ଯଥା ସାଧାରଣ ଥଣ୍ଡା = E<br>☐ No illness/କୌଣସି ଅସୁସ୍ଥତା ନାହିଁ = F<br>→ *If no illness, go to Q9.8*	
child_ill_feeding	9.4 When (child_name) had their illness/es, was he/she given less than usual to eat (food or breastmilk), about the same amount, more than usual, or nothing to eat?<br>ଶିଶୁ ଦେହ ଖରାପ ସମୟରେ ତାକୁ ଆଗ ଅପେକ୍ଷା କମ/ସମାନ/ଅଧିକ ଖାଦ୍ୟ ଦେଉଥିଲେ ନା କିଛି ଦେଉ ନଥିଲେ<br>IF LESS, PROBE: Was he/she given much less than usual to eat or somewhat less?ଦର୍ଶାନ୍ତୁ ଯଦି କମ ଦିଅ ଯାଉଛି ତେବେ କେତେ କମ ଦିଆଯାଉଛି	Nothing to eat/କିଛି ଖାଇବାକୁ ଦିଆଯାଉ ନଥିଲା --0<br>Much less/ବହୁତ କମ -------------------------- 1<br>Somewhat less/କିଛି ମାତ୍ରାରେ କମ-------------- 2<br>Same/ସମ ପରିମାଣ------------------------------ 3<br>More than usual/ସାଧାରଣ ଠାରୁ ଅଧିକ---------4	☐
child_careseeking_y	9.5 Did you seek advice or treatment from for the <illness>? ଯେବେ ଶିଶୁର ଦେହ ଖରାପହୋଇଥିଲାସେହି ସମୟରେ ସ୍ୱାସ୍ଥ୍ୟ ପରାମର୍ଶ କିମ୍ବା ଚିକିତ୍ସା କରିଥିଲେ କି ?	Yes/ହଁ--1<br>No /ନା-- 0<br>→ *If no, go to Q. 9.7*	☐

Variable name	Question	Code	Answer
child_careseeking_where	9.6 From whom did you seek advice or treatment from for the <illness> for (child_name)?<br>ଶିଶୁ ଦେହାଖରାପ ହେଲେ କେଉଁଠିକି ନିଅନ୍ତି?	<multiple answer/ଏକାଧିକ ଉତ୍ତର ସମ୍ଭବ><br>Tick all that apply/ ଉପଯୁକ୍ତ ଉତ୍ତର ଗୁଡିକରେ ଚିହ୍ନ ଦିଅନ୍ତୁ<br>☐ Traditional health practitioners, quacks/ ବୈଦ୍ୟ/ଝଡା ଫୁଙ୍କା = 1<br>☐ Community health service providers (ASHA, ANM, AWW)/ ସାମୁଦାୟିକ ସ୍ୱାସ୍ଥ୍ୟ ସେବା ପ୍ରଦାନକାରୀ (ଆଶା,ଅଙ୍ଗନବାଡି, ଏଂଏନଂଏମ) = 2<br>☐ Institutional medical practitioners (Doctors, RMP)/ ଡାକ୍ତର = 3<br>☐ Local medicine shop/lab technician/pharmacist/ ସ୍ଥାନୀୟ ଔଷଧ ଦୋକାନ/ଲ୍ୟାବ ଟେକନିସିଆନ/ଫର୍ମାଶିଷ୍ଟ = 4<br>☐ No one/କୌଣସିଟି ନୁହେଁ = 0	☐
child_treatment	9.7 Was he/she given any of the following at any time since he/she started having the diarrhoea?<br>ଶିଶୁକୁ ଡାଇରିଆ/ତରଳଝାଡା ଆରମ୍ଭ ହେବା ସମୟରେ ଏହି ମଧ୍ୟରୁ କିଛି ଦିଆଯାଇଥିଲା କି ?<br>(ସମସ୍ତ ବିକଳ୍ପ ଗୁଡିକୁ ପଢନ୍ତୁ)	<multiple answer/ଏକାଧିକ ଉତ୍ତର ସମ୍ଭବ><br>Tick all that apply/ ଉପଯୁକ୍ତ ଉତ୍ତର ଗୁଡିକରେ ଚିହ୍ନ ଦିଅନ୍ତୁ<br>☐ Gruel made from rice [or other grain]/ଭାତରୁ ତିଆରି ହୋଇଥିବା ପାନୀୟ = 1<br>☐ ORS/ଓଂଆରଂଏସ = 2<br>☐ Pill / antibiotic, syrup or injection/ବଟିକା/ଆଣ୍ଟିବାଇଓଟିକ/ଶିରପ/ଇଂଜେକସନ = 3<br>☐ Intravenous (iv)/ଶିରରେ ଇଞ୍ଜେକ୍ସନ = 4<br>☐ Home remedy/herbal medicine/ଘରୋଇ ଚିକିତ୍ସା/ଚେରମୂଳି ଔଷଧ = 5<br>☐ Nothing given/କିଛି ଦିଆ ହୋଇନାହିଁ = 0	

MATERNAL HEALTH /ମାତୃ ସ୍ୱାସ୍ଥ୍ୟ

Variable name	Question	Code	Answer
Note: Now ask the mother directly about her antenatal care during her pregnancy with (child name)<br>ଏବେ ମା କୁ ସିଧାସଳଖ ସେ ଗର୍ଭବତୀ ଥିବା ସମୟରେ ପାଇଥିବା ପ୍ରସବ ପୂର୍ବବତୀ ସେବା ଗୁଡିକ ବିଷୟରେ ପଚାରନ୍ତୁ<br>Be clear that this is not necessarily the mother's latest pregnancy; the questions refer to the time when the mother was pregnant with child_name<br>ଠିକରେଜାଣନ୍ତୁଯେ ଯେତେବେଳେ ମା ଚୟନ ହୋଇଥିବା ଶିଶୁ ପାଇଁ ଗର୍ଭବତୀ ଥିଲେ ସେହି ସମ୍ବନ୍ଧୀୟ ପ୍ରଶ୍ନ ପଚାରନ୍ତୁ(ବର୍ତମାନର ଗର୍ଭଅବସ୍ଥା ବିଷୟରେ ପଚାରନ୍ତୁ ନହିଁ।			

Variable name	Question	Code	Answer
care_chw_mother	**Do not ask if primary caregiver is not the mother/ପ୍ରାଥମିକ ଯତ୍ନକାରୀ ଯଦି ମା ନୁହନ୍ତି ତେବେ ପଚାରନ୍ତୁ ନାହିଁ**<br>*9.8* Did you receive any of the following health care for yourself when pregnant with <child_name> from anganwadis, ASHA or ANM? *Read out all of the options.*<br>ଆପଣ ଏହି ଶିଶୁ (ଶିଶୁର ନାମ) ପାଇଁ ଗର୍ଭବତୀ ଥିବା ସମୟରେ ଆଶା ବା ଏ.ଏନ.ଏମଙ୍କ ପାଖରୁ ଏହି ସେବା ଗୁଡିକ ପାଇଥିଲେ କି ?<br>(ସମସ୍ତ ବିକଳ୍ପ ଗୁଡିକୁ ପଢନ୍ତୁ)	<multiple answer/ବହୁ ଉତ୍ତର ସମ୍ଭବ><br>Tick all that apply/ ଉପଯୁକ୍ତ ଉତ୍ତର ଗୁଡିକରେ ଚିହ୍ନ ଦିଅନ୍ତୁ<br>☐ Vitamin A supplement during pregnancy with <child_name>/ଗର୍ଭବତୀ ଥିବା ସମୟରେ ଭିଟାମିନ ଏ = A<br>☐ Iron folate during pregnancy with <child_name>/ଆଇରନ ବଟିକା = B<br>☐ Deworming tablets during pregnancy with <child_name>/କୃମି ଔଷଧ = C<br>☐ Antenatal care / check up/ଗର୍ଭବତୀ ସମୟରେ ସେବା/ ଯାଞ୍ଚ (ଯେପରିକି ଓଜନ,ରକ୍ତଚାପ,ପେଟ ପରୀକ୍ଷା ଇତ୍ୟାଦି) = D<br>☐ Delivery of the (child_name)/ପ୍ରସବ ସମୟରେ = E<br>☐ Spot feeding/ପ୍ରସବ ପରେ ସ୍ତନ୍ୟପାନ = F<br>☐ TT injections/ଟି.ଟି ଇଂଜେକସନ = G<br>☐ Testing for anaemia /ରକ୍ତହୀନତା ନିମନ୍ତେ ରକ୍ତପରୀକ୍ଷା = H<br>☐ Testing for night vision/ଅନ୍ଧାରକଣା ନିମନ୍ତେ ପରୀକ୍ଷା = I<br>☐ Testing for malaria/ମ୍ୟାଲେରିଆ ନିମନ୍ତେ ରକ୍ତପରୀକ୍ଷା = J<br>☐ None/କୌଣସିଟି ନୁହେଁ = X<br>☐ Don’t know / can’t remember/ଜାଣି ନାହିଁ/ମନେ ନାହିଁ = Y	
care_institution_mother	**Do not ask if primary caregiver is not the mother/ ପ୍ରାଥମିକ ଯତ୍ନକାରୀ ଯଦି ମା ନୁହନ୍ତି ତେବେ ପଚାରନ୍ତୁ ନାହିଁ**<br>9.9 Did you receive any of the following health care for yourself when pregnant with <child_name> from the community health centre, primary health centre, or district hospital?<br>*Read out all of the options.*<br>ଆପଣ ଏହି ଶିଶୁ (ଶିଶୁର ନାମ) ପାଇଁ ଗର୍ଭବତୀ ଥିବା ସମୟରେ ଆପଣଙ୍କ ଅଞ୍ଚଳରେ ଥିବା ଗୋଷ୍ଠୀ ସ୍ୱାସ୍ଥ୍ୟ କେନ୍ଦ୍ର ବା ପ୍ରାଥମିକ ସ୍ୱାସ୍ଥ୍ୟ କେନ୍ଦ୍ର ରୁ କିମ୍ବା ଜିଲ୍ଲା ମୁଖ୍ୟ ଡାକ୍ତରଖାନା ରୁ ସେବା ଗୁଡିକ ଗ୍ରହଣ କରିଥିଲେ କି ?<br>(ସମସ୍ତ ବିକଳ୍ପ ଗୁଡିକୁ ପଢନ୍ତୁ)	<multiple answer/ବହୁ ଉତ୍ତର ସମ୍ଭବ><br>Tick all that apply/ ଉପଯୁକ୍ତ ଉତ୍ତର ଗୁଡିକରେ ଚିହ୍ନ ଦିଅନ୍ତୁ<br>☐ Vitamin A supplement during pregnancy with <child_name>/ଗର୍ଭବତୀ ଥିବା ସମୟରେ ଭିଟାମିନ ଏ = A<br>☐ Iron folate during pregnancy with <child_name>/ଆଇରନ ବଟିକା = B<br>☐ Deworming tablets during pregnancy with <child_name>/କୃମି ଔଷଧ = C<br>☐ Antenatal care / check up/ଗର୍ଭବତୀ କାଳୀନ ସେବା/ଯାଞ୍ଚ(ଯେପରିକି ଓଜନ,ରକ୍ତଚାପ,ପେଟର ପରୀକ୍ଷା,ଇତ୍ୟାଦି) = D<br>☐ Delivery of the (child_name)/ପ୍ରସବକାଳୀନ ସେବା = E<br>☐ Spot feeding/ପ୍ରସବ ପରେ ସ୍ତନ୍ୟପାନ = F<br>☐ T injections/ଟି.ଟି ଇଂଜେକସନ = G<br>☐ Testing for anaemia /ରକ୍ତହୀନତା ନିମନ୍ତେ ରକ୍ତପରୀକ୍ଷା = H<br>☐ Testing for night vision/ଅନ୍ଧାରକଣା ନିମନ୍ତେ ପରୀକ୍ଷା = I<br>☐ Testing for malaria/ମ୍ୟାଲେରିଆ ନିମନ୍ତେ ରକ୍ତପରୀକ୍ଷା = J<br>☐ None/କୌଣସିଟି ନୁହେଁ = X<br>Don’t know / can’t remember ଜାଣି ନାହିଁ/ମନେ ନାହିଁ = Y	

Variable name	Question	Code	Answer
anc_times	**Do not ask if primary caregiver is not the mother/ ପ୍ରାଥମିକ ଯତ୍ନକାରୀ ଯଦି ମା ନୁହନ୍ତି ତେବେ ପଚାରନ୍ତୁ ନାହିଁ**<br>9.10How many times did you receive antenatal care during this pregnancy?<br>**ଆପଣ ଏହି ଶିଶୁ (ଶିଶୁର ନାମ) ପାଇଁ ଗର୍ଭବତୀ ଥିବା ସମୟରେ** କେତେଥରଚେକ ଅପ କରିଥିଲେ?<br>Probe: like weight check, blood pressure, abdomen check<br>Probe:ଯେପରିକି ଓଜନ ମାପିବା , ରକ୍ତ ଚାପ, ପେଟର ଯାଞ୍ଚ ଇତ୍ୟାଦି		☐
anc_iron	**Do not ask if primary caregiver is not the mother/ ପ୍ରାଥମିକ ଯତ୍ନକାରୀ ଯଦି ମା ନୁହନ୍ତି ତେବେ ପଚାରନ୍ତୁ ନାହିଁ**<br>9.11During this pregnancy with (child_name), were you given or did you buy any iron folic acid tablets or syrup?<br>**ଆପଣ ଏହି ଶିଶୁ (ଶିଶୁର ନାମ) ପାଇଁ ଗର୍ଭବତୀ ଥିବା ସମୟରେ ଆପଣଙ୍କୁ ଆଇରନ ବଟିକା ବା ସିରପ ମିଳିଥିଲା କି?ନା ଆପଣ ଅନ୍ୟ କେଉଁଠାରୁ କିଣିଥିଲେ କ** *?*	Yes/ହଁ --- 1<br>No/ନା -- 0	☐
anc_iron_freq	**Do not ask if primary caregiver is not the mother/ ପ୍ରାଥମିକ ଯତ୍ନକାରୀ ଯଦି ମା ନୁହନ୍ତି ତେବେ ପଚାରନ୍ତୁ ନାହିଁ**<br>9.12During the whole pregnancy with (child_name), for how many days (if any) did you take iron-folate? ଗର୍ଭାବସ୍ଥା ସମୟରେ ଆପଣ କେତେଦିନ ଆଇରନ ବଟିକା ଖାଇଛନ୍ତି ?<br><br>Probe: Think about how many packets or bottles you received, how much you took, and how much was left over (if any). Also think about how many months (or which trimesters) you took the supplement for.ମନେ ପକାନ୍ତୁ ଆପଣ କେତୋଟି ଔଷଧ ପକେଟ/ବୋତଲ ପାଇଥିଲେ, ସେଥିରୁ ଆପଣ କେତୋଟି ଖାଇଥିଲେ, କେତୋଟି ଖଇନଥିଲେ, ଏହା ମଧ ଚିନ୍ତା କରନ୍ତୁ ଗର୍ଭାବସ୍ଥା ସମୟରେ କେତେ ମାସ ପରିପୂରକ ଖାଦ୍ୟ ଖାଇଥିଲେ (କେଉଁ ତ୍ରୟମାସିକରେ)।		☐
preg_eat	**Do not ask if primary caregiver is not the mother/ ପ୍ରାଥମିକ ଯତ୍ନକାରୀ ଯଦି ମା ନୁହନ୍ତି ତେବେ ପଚାରନ୍ତୁ ନାହିଁ**<br>9.13Overall, did you eat more, less, or about the same amount during your pregnancy with (child_name) than before you were pregnant?<br>ସମ୍ପୂର୍ଣ ଗର୍ଭବସ୍ଥା ସମୟରେ ଆପଣ ଅଧିକ ଖାଦ୍ୟ ଖାଉଥିଲେ ନା କମ ଖାଉଥିଲେ ନା ସମାନ ପରିମାଣର ଖାଉଥିଲେ?	Ate less/କମ ଖାଉଥିଲି-------------------------------- 1<br>Ate the same/ସମାନ ପରିମାଣର ଖାଉଥିଲି ---------- 2<br>Ate more/ଅଧିକ ଖାଉଥିଲି -------------------------- 3	☐
preg_rest	**Do not ask if primary caregiver is not the mother/ ପ୍ରାଥମିକ ଯତ୍ନକାରୀ ଯଦି ମା ନୁହନ୍ତି ତେବେ ପଚାରନ୍ତୁ ନାହିଁ**<br>9.14Overall, did you takeless rest/ sleep, extra rest/sleep, or about the same amount of rest/ sleep during your pregnancy with (child_name) than before you were pregnant?<br>ସମ୍ପୂର୍ଣ ଗର୍ଭବସ୍ଥା ସମୟରେ ଆପଣ ଅଧିକ ବିଶ୍ରାମ ନେଉଥିଲେ ନା କମ ନେଉଥିଲେ ନା ସମାନ ପରିମାଣର ନେଉଥିଲେ?	Took extra rest/sleep/ଅଧିକ ବିଶ୍ରାମ/ଶୋଉଥିଲି--- 1<br>No change/କୌଣସି ପରିବର୍ତ୍ତନ ନାହିଁ ----------------- 2<br>Took less rest/sleep/ କମ ବିଶ୍ରାମ/ଶୋଉଥିଲି ----- 3	☐

10 MATERNAL DIETARY DIVERSITY (for mothers)/ମା ଖାଦ୍ୟର ବିଭିନ୍ନତା

dds_fast	10.1	Was yesterday a special day, like a celebration or feast day or a fast day where you ate special foods or more or less than usual or did not eat because of fasting/ଗତକାଲି କିଛି ବିଶେଷ ଦିନ ଥିଲା କି, ଯେପରି କୌଣସି ଉତ୍ସବ,ଭୋଜି କିମ୍ବା ଉପବାସର ଦିନ ଥିଲା କି ଯେଉଁଥିରେ ଆପଣ କିଛି ବିଶେଷ, ଅଧିକ ଖାଦ୍ୟ ଖାଇଥିଲେ କିମ୍ବା ଉପବାସ ଯୋଗୁଁ ଖାଇ ନ ଥିଲେ ?	No/ନା---0<br>Yes, celebration feast day/ହଁ,ଉତ୍ସବ ପାଳନ ପର୍ବ---1<br>Yes, fast day/ହଁ, ଉପବାସର ଦିନ --------------------2	☐

Write the responses into CAPI.
Read aloud: Now I'd like to ask you to describe everything that you ate or drank yesterday during the day or night, whether you ate it at home or anywhere else. *(Be clear that now we are asking about the mother's intakes and not the child)*
ବର୍ତ୍ତମାନ ମୁଁ ଆପଣଙ୍କୁ ବିଶେଷ କରି ଗତକାଲି ଦିନରେ ବା ରାତିରେ କଣ କଣ ଖାଇଥିଲେ ଓ ପିଇଥିଲେ ସେ ସମସ୍ତ ବିଷୟ ବର୍ଣନା କରିବାକୁ କହିବି ।(ନିଶ୍ଚିତ ହୁଅନ୍ତୁ ଯେ ବର୍ତ୍ତମାନ ଆମେ ମା ର ଖାଦ୍ୟ ବିଷୟରେ ପଚାରୁଛନ୍ତି ଶିଶୁ ବିଷୟରେ ନୁହେଁ)

Please include all foods and drinks, any snacks or small meals, as well as any main meals. Remember to include all foods you may have eaten while preparing meals or preparing food for others. Please also include food you ate even if it was eaten elsewhere, away from your home. Let's start with the first food or drink consumed yesterday.
ଦୟାକରି ସମସ୍ତ ଖାଦ୍ୟକୁ ସମ୍ପୃକ୍ତ କରନ୍ତୁ ଯେପରି ବିସ୍କୁଟ, ଜଳଖିଆ ଓ ପ୍ରଧାନ ଖାଦ୍ୟ । ଆଉ ଯଦି କିଛି ରୋଷେଇ କରି ଖାଉଥିବେ, ଅନ୍ୟ ମାନଙ୍କ ପାଇଁ ଖାଦ୍ୟ ପ୍ରସ୍ତୁତ କଲାବେଳେ,ଯଦି ବାହାରେ ଖାଉଥିବେ ତାହା ମଧ ଉଲ୍ଲେଖ କରନ୍ତୁ । ଗତକାଲି ପ୍ରଥମେ ଖାଇଥିବା ଖାଦ୍ୟ ଓ ପାନୀୟରୁ ଆରମ୍ଭ କରିବା ।

- PROBE: Did you have anything to eat or drink when you woke up? If yes, what? Anything else?*
- Did you have anything to eat or drink later in the morning? If yes, what? Anything else?*
- Did you eat or drink anything at mid-day? If yes, what? Anything else?*
- Did you have anything to eat or drink during the afternoon? If yes, what? Anything else?*
- Did you have anything to eat in the evening? If yes, what? Anything else?*
- Did you have anything else to eat or drink in the evening before going to bed or during the night?
- If yes, what? Anything else?*
- ଦର୍ଶାନ୍ତୁ: ଯେତେବେଳେ ଆପଣ ବିଛଣା ଛାଡିଲେ ସେତେବେଳେ ଆପଣ କିଛି ଖାଇଲେ କି? ଯଦି ହଁ କଣ ଖାଇଲେ ?
- ସକାଳେ ଆପଣ କିଛି ଖାଇଲେ କି ? ଯଦି ହଁ କଣ ଖାଇଲେ ?
- ମଧ୍ୟାହ୍ନରେ ଆପଣ କିଛି ଖାଇଲେ କି ? ଯଦି ହଁ କଣ ଖାଇଲେ, ତା ବ୍ୟତୀତ ଅନ୍ୟ କିଛି ଖାଇଥିଲେ କି ?
- ଅପରାହ୍ନରେ ଆପଣ କିଛି ଖାଇଲେ କି ? ଯଦି ହଁ କଣ ଖାଇଲେ, ତା ବ୍ୟତୀତ ଅନ୍ୟ କିଛି ଖାଇଥିଲେ କି ?
- ସନ୍ଧ୍ୟା ସମୟରେ ଆପଣ କିଛି ଖାଇଲେ କି ? ଯଦି ହଁ କଣ ଖାଇଲେ, ତା ବ୍ୟତୀତ ଅନ୍ୟ କିଛି ଖାଇଥିଲେ କି ?
- ରାତିରେ ଶୋଇବାକୁ ଯିବା ପୂର୍ବରୁ ଆପଣ କିଛି ଖିଆପିଆ କରିଥିଲେ କି କିମ୍ବା ରାତିରେ ଆପଣ କିଛି ଖାଇଥିଲେ କି ? ଯଦି ହଁ କଣ ଖାଇଲେ, ତା ବ୍ୟତୀତ ଅନ୍ୟ କିଛି ଖାଇଥିଲେ କି ?

* For each eating episode, after the respondent mentions foods and drinks, probe to ask if she ate or drank anything else. Continue probing until she says "no, nothing else". If the respondent mentions a mixed dish like a soup or stew, ask for all the ingredients in the mixed dish. For mixed dishes where it is possible to pick out ingredients or consume only broth, ask if she herself ate each ingredient or if she only had the broth. Continue to probe about ingredients until she says "nothing else".
ଉତ୍ତରଦାତା ପ୍ରତ୍ୟେକ ଖାଦ୍ୟ ଓ ପାନୀୟର ବର୍ଣନା କଲାପରେ ତାଙ୍କୁ ଆଉ ଥରେ ପଚାରନ୍ତୁ ଏହି ଖାଦ୍ୟ ବ୍ୟତୀତ ସେ ଆଉ କିଛି ଖାଇଛନ୍ତି କି ?ସେ ପର୍ଯ୍ୟନ୍ତ ପଚାରନ୍ତୁ ଯେ ପର୍ଯ୍ୟନ୍ତ ସେ କହିନାହାନ୍ତି "ଆଉ କିଛି ଖାଇନାହିଁ "। ଯଦି ଉତ୍ତରଦାତା କୌଣସି ସୀଝା ସଂତୁଳା ବା ତରକାରୀ ବା ଘାଣ୍ଟ ତରକାରୀ ଇତ୍ୟାଦି କୁହନ୍ତି ତେବେ ସେହି ଖାଦ୍ୟରେ ମିଶା ଯାଇଥିବା ପନିପରିବା ଓ ଅନ୍ୟାନ୍ୟ ଜିନିଷ ବିଷୟରେ ପଚାରନ୍ତୁ । ଯଦି ସେ କୌଣସି ମିଶାମିଶି ଖାଦ୍ୟ ବା ତରକାରୀ କଥା କୁହନ୍ତି ତେବେ ତାଙ୍କୁ ପଚାରନ୍ତୁ ସେ ଝୋଳ ଆକାରରେ ଖାଇଲେ ନା ପଡିଥିବା ସମସ୍ତ ଦ୍ରବ୍ୟ ଖାଇଲେ । ଯେଉଁ ଯେଉଁ ଦ୍ରବ୍ୟ ସେ ଖାଇଲେ ତାହା ତାଙ୍କୁ ପଚାରନ୍ତୁ ଓ ପଚାରିଚାଲନ୍ତୁ ସେ ପର୍ଯ୍ୟନ୍ତ ସେ "ନା ଆଉ କିଛି ନୁହେଁ " ନ କହିଛନ୍ତି ।

***** Ask about any food groups that were not reported in the free recall *****
ଖାଦ୍ୟ ମନେ ପକାଇଲେ ବେଳେ ଛାଡି ଯାଇଥିବା ଖାଦ୍ୟ ଗୁଡିକ ଆଉଥରେ ପଚାରିଲେ

No.	Answers (space for writing recalled information)	Food group
1.		
2.		
3.		

4.		
5.		
6.		
7.		
8.		
9.		
10.		
11.		
12.		
13.		
14.		
15.		
16.		
17.		
18.		
19.		
20		
21.		
22.		
23.		

Variable name	Food item	Code	Answer
Using the recalled information, record whether <mother_name> had any of the following food groups/ଉତ୍ତରଦାତା (ମା ର ନାମ) ଙ୍କୁ ପଚାରନ୍ତୁ ନିମ୍ନଲିଖିତ ଖାଦ୍ୟ ମଧ୍ୟରୁ ସେ କୌଣସି ଖାଦ୍ୟ ଖାଇଥିଲେ କି ?			
dds_mother_1	10.1.1 Roti, rice (any type including puffed or beaten), peetha, pastry (e.g. grain used in singada), noodles, Chowmein, powdered grain mixture, other food made from grains<br>ରୁଟି, ଭାତ, ମୁଢି, ଚୂଡା, ପିଠା, ସିଙ୍ଗଡା, ନୁଡୁଲ୍ସ,ଚାଓମିନ, ଛତୁଆ, ଅନ୍ୟାନ୍ୟ ଶସ୍ୟରୁ ତିଆରି ହୋଇଥିବା ଗୁଣ୍ଡ ଇତ୍ୟାଦି	Yes/ହଁ --------------------------------- 1<br>No/ନା --------------------------------- 0<br>Don't know/ଜାଣି ନାହାନ୍ତି ------------- 98	☐
dds_mother_2	10.1.2 Dal, beans, dried / mature peas (including besan / gram flour), or soyabean<br>ଡାଲି,ବିନ୍ସ, ମଟର, ବେସନ, ସୋୟାବିନ ଇତ୍ୟାଦି	Yes/ହଁ --------------------------------- 1<br>No/ନା --------------------------------- 0<br>Don't know/ଜାଣି ନାହାନ୍ତି ------------- 98	☐
dds_mother_3	10.1.3 Yellow pumpkin, carrot, sweet potato (orange/yellow inside)<br>ବୋଇତି କଖାରୁ, ଗାଜର, କନ୍ଦମୂଳ, କମଳା ଓ ହଳଦିଆ ଅଂଶଥିବା ପରିବା	Yes/ହଁ --------------------------------- 1<br>No/ନା --------------------------------- 0<br>Don't know/ଜାଣି ନାହାନ୍ତି ------------- 98	☐
dds_mother_4	10.1.4 White potatoes, white yams, elephant foot (Ol), aluwa, taro, or any other foods made from starchy roots<br>ଆଳୁ, ଖମ୍ବଆଳୁ, ମାଟିଆଳୁ, ସାରୁ, ବହଳିଆ ମଣ୍ଡ ହେଉଥିବା ପରିବା	Yes/ହଁ --------------------------------- 1<br>No/ନା --------------------------------- 0<br>Don't know/ଜାଣି ନାହାନ୍ତି ------------- 98	☐
dds_mother_5	10.1.5 Dark green leafy vegetable /ସବୁଜ ପନିପରିବା	Yes/ହଁ --------------------------------- 1<br>No/ନା --------------------------------- 0<br>Don't know/ଜାଣି ନାହାନ୍ତି ------------- 98	☐
dds_mother_6	10.1.6 Ripe mango, ripe papaya, ripe jackfruit /ପାଚିଲା ଆମ୍ବ, ପାଚିଲା ଅମୃତଭଣ୍ଡା, ପାଚିଲା ପଣସ	Yes/ହଁ --------------------------------- 1<br>No/ନା --------------------------------- 0<br>Don't know/ଜାଣି ନାହାନ୍ତି ------------- 98	☐
dds_mother_7	10.1.7 Unripe mango, unripe papaya, unripe jackfruit / କଞ୍ଚା ଆମ୍ବ, କଞ୍ଚା ଅମୃତଭଣ୍ଡା, କଞ୍ଚା ପଣସ	Yes/ହଁ --------------------------------- 1<br>No/ନା --------------------------------- 0<br>Don't know/ଜାଣି ନାହାନ୍ତି ------------- 98	☐
dds_mother_8	10.1.8 Any other fruits /ଅନ୍ୟ କୌଣସି ଫଳମୂଳ	Yes/ହଁ --------------------------------- 1<br>No/ନା --------------------------------- 0<br>Don't know/ଜାଣି ନାହାନ୍ତି ------------- 98	☐
dds_mother_9	10.1.9 Any other vegetables/ଅନ୍ୟ କୌଣସି ପନିପରିବା	Yes/ହଁ --------------------------------- 1<br>No/ନା --------------------------------- 0<br>Don't know/ଜାଣି ନାହାନ୍ତି ------------- 98	☐
dds_mother_10	10.1.10 Liver, kidney, heart, or other organ meats/କଲିଜା, ବୃକକ,ହୃଦୟ, ମାଂସର ଅନ୍ୟାନ୍ୟ ଅଂଶ	Yes/ହଁ --------------------------------- 1<br>No/ନା --------------------------------- 0<br>Don't know/ଜାଣି ନାହାନ୍ତି ------------- 98	☐

Variable name	Food item	Code	Answer
dds_mother_11	10.1.11 Any meat, such as chicken, duck or other birds, pork, lamb, goat, buffalo, rabbit, or mouse / rat କୌଣସି ମାଂସ ଯେପରିକି କୁକୁଡା, ବତକ, ଅନ୍ୟାନ୍ୟ ପକ୍ଷୀ, ଘୁଷୁରି, ମେଣ୍ଢା, ଛେଳି, ମହିଷୀ, ଠେକୁଆ, ମୂଷା ଇତ୍ୟାଦି	Yes/ହଁ --------------------------------- 1<br>No/ନା --------------------------------- 0<br>Don't know/ଜାଣି ନାହାନ୍ତି ------------- 98	☐
dds_mother_12	10.1.12 Egg (chicken or duck) /ଅଣ୍ଡା(କୁକୁଡା ବା ବତକ)	Yes/ହଁ --------------------------------- 1<br>No/ନା --------------------------------- 0<br>Don't know/ଜାଣି ନାହାନ୍ତି ------------- 98	☐
dds_mother_13	10.1.13 Fresh or dried fish, shellfish, or seafood/ମାଛ ବା ଶୁଖୁଆ, ଶାମୁକା ଓ ଅନ୍ୟାନ୍ୟ ସମୁଦ୍ର ଜାତ ଆମିଷ	Yes/ହଁ --------------------------------- 1<br>No/ନା --------------------------------- 0<br>Don't know/ଜାଣି ନାହାନ୍ତି ------------- 98	☐
dds_mother_14	10.2Grubs, snails or insects /ଗେଣ୍ଡା, ଶାମୁକା ବା ଅନ୍ୟାନ୍ୟ ପୋକ	Yes/ହଁ --------------------------------- 1<br>No/ନା --------------------------------- 0<br>Don't know/ଜାଣି ନାହାନ୍ତି ------------- 98	☐
dds_mother_15	10.2.1 Nuts or seeds e.g. peanut, cashew, sunflower, Dori, Mahua, Kudrum, Tisi, Ramtia, Linseed, Sesame, Chahar, mustard, mahua ବିଭିନ୍ନ ପ୍ରକାର ମଞ୍ଜି ଯଥା ବାଦାମ, କାଜୁ,ସୂର୍ଯ୍ୟମୁଖୀ, ମହୁଲ,ଶୋରିଷ, ମହୁଆ ଇତ୍ୟାଦି	Yes/ହଁ --------------------------------- 1<br>No/ନା --------------------------------- 0<br>Don't know/ଜାଣି ନାହାନ୍ତି ------------- 98	☐
dds_mother_16	10.2.2 Paneer or other cheese, yogurt / curd, any kind of animal milk, or other milk products but not including butter, cream, or ice cream ଛେନା ଜାତୀୟ ଖାଦ୍ୟ, ଦହି, କ୍ଷୀର ଜାତୀୟ ଦ୍ରବ୍ୟ (ଘିଅ, ଲହୁଣୀ ଓ ଆଇସକ୍ରିମକୁ ଛାଡି)	Yes/ହଁ --------------------------------- 1<br>No/ନା --------------------------------- 0<br>Don't know/ଜାଣି ନାହାନ୍ତି ------------- 98	☐
dds_mother_17	10.2.3 Any food with oil, ghee, fat or butter, includingdeep-fried or pan-fried foods cooked with oil ଘିଅ ବା ତେଲରେ ପ୍ରସ୍ତୁତ ଖାଦ୍ୟ	Yes/ହଁ --------------------------------- 1<br>No/ନା --------------------------------- 0<br>Don't know/ଜାଣି ନାହାନ୍ତି ------------- 98	☐
dds_mother_18	10.2.4 Sugary foods (chocolates, Chena poda, sweets, candies, cake, biscuits), e.g. jilli / jilebi, laddu ମିଠା ଜାତୀୟ ଖାଦ୍ୟ ଯଥା ଚକୋଲେଟ, ଛେନାପୋଡ, ସନ୍ଦେଶ, ମିଠେଇ, ପିଠା, କେକ, ବିସ୍କୁଟ, ଲଡୁ, ଝିଲ୍ଲୀ ଇତ୍ୟାଦି	Yes/ହଁ --------------------------------- 1<br>No/ନା --------------------------------- 0<br>Don't know/ଜାଣି ନାହାନ୍ତି ------------- 98	☐

Variable name	Food item		Code	Answer
dds_mother_19	10.2.5	Condiments for flavour (chillies, spices, herbs, fish powder, or seeds) = U<br>ସ୍ୱାଦ ବଢାଇବା ନିମନ୍ତେ ଲଙ୍କା, ମସଲା, ଚେରମୂଳ, ମାଛଗୁଣ୍ଡ ବା ମଞ୍ଜି	Yes/ହଁ --------------------------------- 1<br>No/ନା --------------------------------- 0<br>Don't know/ଜାଣି ନାହାନ୍ତି ------------- 98	☐
dds_mother_20	10.2.6	Other beverages and foods (tea or coffee if not sweetened, clear broth)<br>ଅନାନ୍ୟ ପାନୀୟ ଓ ଖାଦ୍ୟ	Yes/ହଁ --------------------------------- 1<br>No/ନା --------------------------------- 0<br>Don't know/ଜାଣି ନାହାନ୍ତି ------------- 98	☐
dds_mother_21	*10.2.7*	Alcoholic beverages (*handiya*)<br>ନିଶା ଦ୍ରବ୍ୟ	Yes/ହଁ --------------------------------- 1<br>No/ନା --------------------------------- 0<br>Don't know/ଜାଣି ନାହାନ୍ତି ------------- 98	☐

11.0 HOUSEHOLD FOOD PROVISIONING / (MAHFP) ପରିବାରର ଖାଦ୍ୟ ପେୟ

Months of adequate household food provisioning/ (MAHFP)
Now I would like to ask you about your household's food supply during different months of the year. When responding to these questions, please think back over the last 12 months, from now to the same time last year./ଏବେ ମୁଁ ଆପଣଙ୍କୁ ବର୍ଷର ବିଭିନ୍ନ ମାସରେ ଆପଣଙ୍କ ପରିବାରର ଖାଦ୍ୟ ଯୋଗାଣ ସ୍ଥିତି ବିଷୟରେ ପଚାରିବି। ଏହି ସବୁ ପ୍ରଶ୍ନର ଉତର ଦେବାପାଇଁ ଦୟାକରି ଗତ ବାର ମାସର କଥା ମାନେ ପକାନ୍ତୁ। ବର୍ତମାନ ଠାରୁ ଆରମ୍ଭ କରି ଗଲା ବର୍ଷ ଏହି ସମୟ ପର୍ଯ୍ୟନ୍ତ ।

Variable name	Question	Code	Answer
mahfp_any	11.1 Were there months, in the past 12 months, in which you did not have enough food to meet your family's needs?<br>ଗତ ୧୨ମାସରେ ଏଭଳି କୌଣସି ମାସ ଥିଲା କି ଯେଉଁ ମାସରେ ଆପଣଙ୍କ ପରିବାର ପାଇଁ ଯେତିକି ଖାଦ୍ୟ ଦରକାର ଥିଲା ତା ଠାରୁ କମ ଥିଲାକି?This includes any kind of food from any source, such as own production, purchase or exchange, food aid, or borrowing.<br>ଏଥିରେ ନିଜେ ଉତ୍ପାଦିତ, କିଣିଥିବା,ଅଦଳବଦଳ, ଉଧାରରେ ଆଣିଥିବା ଇତ୍ୟାଦି ଖାଦ୍ୟ ରହିଛି ।(ଜୁନ 2015 ରୁ ମେ ମେ 2016)	Yes/ହଁ --- 1<br>→ *Go to 11.2*<br>No/ନା --- 0<br>→ *Go to 11.3*	☐

Variable name	Question	Code	Answer
mahfp	11.2 If yes, which were the months in the past 12 months during which you did not have enough food to meet your family's needs?<br>ଯଦି ହଁ, ତେବେ କେଉଁ ମାସ ଗୁଡିକରେ ଆପଣଙ୍କ ପରିବାରରେ ଖାଦ୍ୟ କମ ଥିଲା?Do not read the list of months aloud.<br>ମାସର ତାଲିକା ପଢନ୍ତୁ ନାହିଁ<br><br>Probe to make sure the respondent has thought about the entire past 12 months.<br>ନିଶ୍ଚିତ ହୁଅନ୍ତୁ ଯେ ଉତ୍ତରଦାତା ଗତ ୧୨ ମାସ ବିଷୟରେ ଭାବୁଛନ୍ତି।	<multiple answer/ବହୁ ଉତ୍ତର ସମ୍ଭବ><br>Tick all the apply<br><br>☐ Chaitra/ଚୈତ୍ର = A<br>☐ Vaisakha/ବୈଶାଖ = B<br>☐ Jyestha/ଜ୍ୟେଷ୍ଠ = C<br>☐ Asadha/ଅଷାଢ = D<br>☐ Shrawan/ଶ୍ରାବଣ = E<br>☐ Bhadra/ଭାଦ୍ରବ = F<br>☐ Ashvin/ଆଶ୍ୱିନ = G<br>☐ Kartika/କାର୍ତ୍ତିକ = H<br>☐ Agrahayana/ମାର୍ଗଶିର = I<br>☐ Pausa/ପୌଷ = J<br>☐ Magha/ମାଘ = K<br>☐ Phalguna/ଫାଲଗୁନ = L	
hfias_worry	11.3 In the coming four weeks, are you worried that there will not be enough food in the household due to lack of resources/ସମ୍ବଳ ଅଭାବରୁ ଆସନ୍ତା 4 ସାପ୍ତାହରେ ଆପଣଙ୍କ ପରିବାର ପାଇଁ ଯଥେଷ୍ଟ ଖାଦ୍ୟ ନଥିବାକୁ ନେଇ ଆପଣ ଚିନ୍ତିତ ଅଛନ୍ତି କି?	Yes/ହଁ-- 1<br>No/ନା--- 0<br>Can't say/କହି ପାରିଲେ ନାହିଁ-------------------- 98	☐
hfias_prefer	11.4 In the coming four weeks, do you anticipate you may not be able to eat the food you prefer due to lack of resources/ଆପଣ ଅନୁମାନ କରୁଛନ୍ତି କି ଆସନ୍ତା 4 ସାପ୍ତାହରେ ସମ୍ବଳ ଅଭାବରୁ ଆପଣଙ୍କପସନ୍ଦର ଖାଦ୍ୟ ଖାଇପାରିବେନି?	Yes/ହଁ-- 2<br>Maybe/ହୋଇପାରେ--------------------------------- 1<br>No/ନା--- 0<br>Can't say/କହି ପାରିଲେ ନାହିଁ----------------------- 98	☐

12.0 Entitlements/ସତ୍ତ୍ୱାଧିକାର/ହିତାଧିକାରୀ

Variable name	Question	Code	Answer

Variable name	Question	Code	Answer
jsy	12.1 Did you receive any money for institutional birth for (child_name) under the Janani Suraksha Yojana/ ଶିଶୁଟି ଡାକ୍ତରଖାନାରେ ଜନ୍ମହେଲା ପରେ ଆପଣଙ୍କୁ ଜନନୀ ସୁରକ୍ଷା ଯୋଜନାରେ କିଛି ଟଙ୍କା ମିଳିଥିଲା କି?<br>Probe: If unsure, involve other household members who might know.<br>ଦର୍ଶାନ୍ତୁ: ଯଦି କହିନପରିବେ ତେବେ ପରିବାରରଅନ୍ୟସଦଶ୍ୟମାନଙ୍କୁସାମିଲ କରନ୍ତୁଯେଉଁମାନେଜାଣିଥିବେ।	Yes/ହଁ---------------------------- 1<br>No/ନା---------------------------- 0<br>→ *go to 12.3*<br>Don't know-/ ଜଣା ନାହିଁ ----------2<br>→ *go to 12.3*	☐
jsy_rupees	12.2 How much money did you receive under JSY/ଜନନି ସୁରକ୍ଷା ଯୋଜନାରେ ଆପଣଙ୍କୁ କେତେ ଟଙ୍କା ମିଳିଥିଲା?	Enter value in Rupees. If respondent doesn't know, enter '98'/ମୂଲ୍ୟକୁ ଟଙ୍କାରେ ଲେଖନ୍ତୁ । ଯଦି ଉତ୍ତରଦାତା ଜାଣି ନାହାନ୍ତି ୨୮ ଲେଖନ୍ତୁ	☐
mamata_enrol	12.3 Did you enrol in the MAMATA scheme when you were pregnant with (child_name)/ଆପଣ ଯେତେବେଳେ ଗର୍ଭବତୀ ଥିଲେ ମମତା ଯୋଜନାରେ ଆପଣଙ୍କ ନାମ ଲେଖା ହୋଇଥିଲା କି?<br>Probe: If unsure, involve other household members who might know.<br>ଦର୍ଶାନ୍ତୁ: ଯଦି କହିନପରିବେ ତେବେ ପରିବାରରଅନ୍ୟସଦଶ୍ୟମାନଙ୍କୁସାମିଲ କରନ୍ତୁଯେଉଁମାନେଜାଣିଥିବେ	Yes/ହଁ---------------------------- 1<br>No/ନା---------------------------- 0<br>→ *go to 13.1*<br>Don't know/ଜଣା ନାହିଁ-------------2<br>→ *go to 13.1*	☐
mamata0	12.4 Did you receive /have you received money from the Mamata scheme when you were pregnant with (child_name)/ ଆପଣ ଯେତେବେଳେ ଗର୍ଭବତୀ ଥିଲେ ମମତା ଯୋଜନାରୁ ଟଙ୍କା ପାଇଥିଲେ କି?<br>Probe: If unsure, involve other household members who might know.<br>ଦର୍ଶାନ୍ତୁ: ଯଦି କହିନପରିବେ ତେବେ ପରିବାରରଅନ୍ୟସଦଶ୍ୟମାନଙ୍କୁସାମିଲ କରନ୍ତୁଯେଉଁମାନେଜାଣିଥିବେ	Yes/ହଁ--------------------------- 1<br>No/ନା--------------------------- 0<br>→ *go to 13.1*<br>Don't know/ଜଣା ନାହିଁ-----------2<br>→ *go to 13.1*	☐
mamata_cashinstal	12.5 How many cash instalments did you receive/have you received from the MAMATA scheme/ ମମତା ଯୋଜନା ଅଧୀନରେ ଆପଣ କେତେ କିସ୍ତିରେ ଟଙ୍କା ପାଇଥିଲେ?	Enter value in Rupees. If respondent doesn't know, enter '98'/ମୂଲ୍ୟକୁ ଟଙ୍କାରେ ଲେଖନ୍ତୁ । ଯଦି ଉତ୍ତରଦାତା ଜାଣି ନାହାନ୍ତି ୨୮ ଲେଖନ୍ତୁ	☐

13.0 WATER, SANITATION& HYGIENE/ଜଳ, ପରିମଳ ଏବଂ ସ୍ୱାସ୍ଥ୍ୟ ରକ୍ଷା (WASH)

Variable name	Question	Code	Answer
watersource	13.1 If the child drinks water, what is the main source of drinking water for (child_name)?<br>13.2 ଯଦି ଶିଶୁପାଣିପିଉଥାଏ,ତେବେଶିଶୁ(ଶିଶୁର ନାମ) ପିଉଥିବା ପାଣି କେଉଁଠାରୁ ଆଣନ୍ତି ?	<Single answer/ଗୋଟିଏ ଉତ୍ତର ଲେଖନ୍ତୁ ><br>A. Piped water into dwelling/ଘରେ ପାଇପ ପାଣିର ବ୍ୟବସ୍ଥା ରହିଛି<br>B. Piped water to yard/plot/ଘରଅଗଣାକୁ/ବାଡିରେ ପାଇପ ପାଣିର ସୁବିଧା ରହିଛି<br>C. Public tap/standpipe/ସର୍ବସାଧାରଣ କଳ/ଟ୍ୟାପ ପାଣି<br>D. Tubewell/borehole/ନଳକୂପ/ବୋରୱେଲ<br>E. Protected dug wel/ଘୋଡା ହୋଇଥିବା କୂଅ<br>F. Unprotected dug well/ଖୋଲା କୂଅ<br>G. Protected spring/ସୁରକ୍ଷିତ ଝରଣା<br>H. Unprotected spring/ ଅସୁରକ୍ଷିତ ଝରଣା<br>I. Rainwater collection/ବର୍ଷାଜଳ ସଂରକ୍ଷଣ<br>J. Bottled water/ବୋତଲ ପାଣି<br>K. Cart with small tank/drum/ଶଗଡ ଦ୍ୱାରା ଡ୍ରମରେ ପାଣି ଅଣୁଛନ୍ତି<br>L. Tanker-truck/ଜଳବାହୀ ଗାଡି<br>M. Surface water (river, dam, lake, pond, stream, canal, irrigation channels)/ଭୂ-ପୃଷ୍ଠ ଜଳ (ନଦୀ, ଡ୍ୟାମ, ହ୍ରଦ, ପୋଖରୀ, ଝରଣା, କେନାଲ, ଜଳସେଚନ ପାଇଁ ଥିବା କେନାଲ<br>X. Other/ଅନ୍ୟାନ୍ୟ<br>Y. Child does not drink water ଶିଶୁଟି ପାଣି ପିଉନାହିଁ<br>→ *If Y, go to 13.5*	☐
water_treat	13.3 Do you normally do anything to your water to make it safer to drink (for <child_name>)?<br>ଶିଶୁ (ଶିଶୁର ନାମ)ର ପିଇବା ପାଣିକୁ ବିଶୁଦ୍ଧ କରିବା ପାଇଁ ଆପଣ କିଛି କରନ୍ତିକି?	Yes/ହଁ---------------------------------- 1<br>No/ନା---------------------------------- 0<br>→ *If no, go to 13.5*	☐

Variable name	Question	Code	Answer
water_treat_methods	13.4What do you normally do?<br>(Do not read the list aloud)<br>Probe: Anything else?<br><br>ଆପଣ ସାଧାରଣତଃ କଣ କରନ୍ତି ? (ତାଲିକା ପଢନ୍ତୁ ନାହିଁ)<br>ଦର୍ଶାନ୍ତୁ: ଆଉ କିଛି ?<br>*Record all items mentioned/*ସମସ୍ତ ଉତ୍ତର ଉଲ୍ଲେଖ କରନ୍ତୁ	<multiple answer/ଏକାଧିକ ଉତ୍ତର ସମ୍ଭବ><br>Tick all that apply.<br>☐ Boil/ସିଝା ପାଣି = A<br>☐ Add bleach/chlorine/ବ୍ଲିଚିଂ ପାଉଡର ପକାନ୍ତି = B<br>☐ Iodine/ଆୟୋଡିନ = C<br>☐ Strain it through a cloth/କପଡ଼ାରେ ଛାଣି ରଖନ୍ତି = D<br>☐ Use a water filter (ceramic, sand, composite, etc.)/ଫିଲଟର ବ୍ୟବହାର କରନ୍ତି = E<br>☐ Solar disinfection/ସୌରଶକ୍ତି ଦ୍ୱାରା ଜୀବାଣୁମୁକ୍ତ କରନ୍ତି = F<br>☐ Let it stand and settle/ଜଳକୁ ସ୍ଥିରକରି ରଖନ୍ତି = G<br>☐ Alum (aluminium sulphate)/ଫିଟିକିରି/ଆଲମ ପକାନ୍ତି = H<br>☐ Other/ଅନ୍ୟାନ୍ୟ = X	
toilet	13.5What kind of toilet facility do you own?/ ଆପଣଙ୍କର କେଉଁ ପ୍ରକାର ପାଇଖାନା ସୁବିଧାଅଛି?	<Single answer/ ଗୋଟିଏ ଉତ୍ତର ଲେଖନ୍ତୁ ><br>1. Flush toilet/ଫ୍ଲସ ପାଇଖାନା<br>2. Piped sewer system/ପାଇପ ଦ୍ୱାରା ସଂଯୋଗ କରାଯାଇଥିବା ପାଇଖାନା<br>3. Septic tank/ସେପ୍ଟିକ ଟାଙ୍କି ପାଇଖାନା<br>4. Flush/pour flush to pit latrine/ପାଣିଦ୍ୱାରା ସଫା କରାଯାଉଥିବା ପାଇଖାନା<br>5. Ventilated improved pit latrine (VIP)/ଭି.ଆଇ.ପି ପାଇଖାନା<br>6. Pit latrine with slab/ସ୍ଲାବ ସହିତ ଫିଟ ଥିବା ପାଇଖାନା<br>7. Composting toilet/ଖତ ବନେଇବା ପାଇଖାନା<br>8. Flush/pour flush to elsewhere/ ଫ୍ଲସ ପାଇଖାନା<br>9. Pit latrine without slab/ସ୍ଲାବ ନ ଥିବା ପାଇଖାନା<br>10. Bucket/ବାଲଟି<br>11. Hanging toilet or hanging latrine/ଝୁଲନ୍ତା ପାଇଖାନା<br><br>12. No facility owned (may use others' facility, or bush or field/ସୁବିଧା ନାହିଁ ବା ବୁଦା/ପଡିଆ<br>→ *If no facility, go to 13.7*	☐
toilet_share	13.6Do you share this facility with other households/ ଆପଣଙ୍କ ପାଇଖାନାକୁଅନ୍ୟ ଘରର ଲୋକମାନେ ବ୍ୟବହାର କରନ୍ତି କି?	Yes/ହଁ--------------------------------- 1<br>No/ନା--------------------------------- 0	☐

Variable name	Question	Code	Answer
toilet_child	13.7 The last time [child_name] passed stools, what was done to dispose of the stools/ଶେଷଥର ଶିଶୁ ଝାଡାକଲାପରେ ଆପଣ ଝାଡାକୁ କଣ କଲେ ?	A. Child used toilet/latrine/ଶିଶୁ ପାଇଖାନା ବ୍ୟବହାର କରେ<br>B. Put/rinsed into toilet or latrine/ପାଇଖାନାରେ ଢଳାଯାଏ<br>C. Put/rinsed into drain or ditch/ନାଳ ବା ଗାତରେ ପକାଯାଏ<br>D. Thrown into garbage/ଅଳିଆ ଗଦାରେ ଫୋପାଡି ଥାଉ<br>E. Buried/cat ପୋତି ଦେଉ/ମାଟିରେ ଘୋଡାଇ ଦଉ<br>F. Left in the open/field/ଖୋଲା ପଡିଆରେ ଫୋପାଡି ଦେଉ<br>G. Left near a waterbody/ପାଣି ଥିବା ସ୍ଥାନରେ ଫୋପାଡି ଦେଉ<br>X. Other/ଅନ୍ୟାନ୍ୟ	☐
handwash_materials	13.8 What materials do you usually use when you wash your hands?/<br>ହାତ ଧୋଇବା ପାଇଁ ଆପଣ ସାଧାରଣତଃ କଣ ବ୍ୟବହାର କରନ୍ତି?	<multiple answer/ ଏକାଧିକ ଉତ୍ତର ସମ୍ଭବ><br>Tick all that apply<br>☐ Water/ପାଣି = A<br>☐ Soap/ସାବୁନ = B<br>→*go to 13.9*<br>☐ Ash/ପାଉଁଶ = C<br>☐ Other/ଅନ୍ୟାନ୍ୟ = X	

Variable name	Question	Code	Answer
handwash	13.9 When do you usually wash your hands with soap?<br>Probe the mother by asking about the daily activities after which she washes her hands.<br>କେଉଁ କେଉଁ କାମ କଲାପରେ ଆପଣ ସାବୁନରେ ହାତ ଧୁଅନ୍ତି ?<br>Do not read the list aloud. *Record all items mentioned/*ତାଲିକା ପଢନ୍ତୁ ନାହିଁ, ଯାହା କହୁଛନ୍ତି ଉଲ୍ଲେଖ କରନ୍ତୁ	<multiple answer/ଏକାଧିକ ଉତ୍ତର ସମ୍ଭବ><br>Tick all that apply/ଉପଯୁକ୍ତ ଉତ୍ତର ଗୁଡିକରେ ଚିହ୍ନ ଦିଅନ୍ତୁ<br>☐ After defecation/ମଳତ୍ୟାଗ ପରେ = A<br>☐ After handling child’s faeces (cleaning a young child's bottomor washing soiled clothes)/ଶିଶୁର ମଳ ସଫା କଲା ପରେ/ଶିଶୁର ପଛ ଧୋଇବାପରେ/ଶିଶୁର ମଳ କପଡା ଧୋଇବା ପରେ = B<br>☐ Before cooking/preparing food/ରୋଷେଇ କରିବା ପୂର୍ବରୁ = C<br>☐ Before eating/ଖାଇବା ପୂର୍ବରୁ = D<br>☐ Before feeding children/ପିଲାକୁ ଖୁଆଇବା ପୂର୍ବରୁ = E<br>☐ After cooking/eating/ରୋଷେଇ/ଖାଇବା ପରେ = F<br>☐ After feeding children/ପିଲାକୁ ଖୁଆଇବା ପରେ = G<br>☐ After cleaning the house/compound/ଘର ଦ୍ୱାର ସଫା କରିବା ପରେ = H<br>☐ After disposing garbage/ଅଳିଆ ସଫା କରିବା ପରେ = I<br>☐ After handling animals/animal dung/ଗୁହାଳ ସଫା କରିବା ପରେ = J<br>☐ Before picking up the child/ପିଲାଙ୍କୁ ଧରିବା ପୂର୍ବରୁ = K<br>☐ Don’t know/ଜାଣି ନାହିଁ = X	
wash	13.10 Observation spot check: Can you observe any animals/livestock/dung (fresh) in the cooking area?<br>ଗୃହର ପରିବେଶ ନିରୀକ୍ଷଣ କରନ୍ତୁ: ରୋଷେଇ କରୁଥିବା ସ୍ଥାନରେ କୌଣସି ପଶୁ/ପକ୍ଷୀ/ତାଙ୍କର ସଦ୍ୟ ମଳ ପଡିଥିବାର ଦେଖି ଥିଲେ କି?<br>The dung used for wall plaster or cooking fuel is not included. /କାନ୍ଥରେ ଦିଆଯାଇଥିବା ଗୋବର ଲେପ ବା ଗୋବର ଜାଳେଣୀକୁ ନିରୀକ୍ଷଣରେ ଅନ୍ତରଭୁକ୍ତ କରନ୍ତୁ ନାହିଁ ।	Yes/ହଁ---------------------------------- 1<br>No/ନା---------------------------------- 0	☐

14.0 Abbreviated Women's Empowerment in Agriculture Index /ସଂକ୍ଷେପରେକୃଷି କାର୍ଯ୍ୟରେ ମହିଳା ମାନଙ୍କ ସଶକ୍ତିକରଣ

For this part of the interview, try to interview the individual in private or where other members of the household cannot overhear or contribute answers. Do not attempt to make responses between the primary male decision-maker and the primary female decision-maker the same; it is ok for them to be different.

ଏହି ଭାଗର ସାକ୍ଷାତକାର ନିମନ୍ତେ, ସାକ୍ଷାତକାରଟିକୁ ଯଥାସମ୍ଭବ ଏକାନ୍ତରେ କରିବାକୁ ଚେଷ୍ଟା କରନ୍ତୁ ଯେଉଁଠାରେ ପରିବାରର ଅନ୍ୟ ସଦସ୍ୟମାନେ ଶୁଣି ପାରିବେନି ବା ଉପରେ ପଡି ଉତ୍ତର ନଦିଅନ୍ତି । ପରିବାରର ମୁଖ୍ୟ ମହିଳା ସଦସ୍ୟ ଓ ମୁଖ୍ୟ ପୁରୁଷ ସଦସ୍ୟଙ୍କ ଉତ୍ତର ସମାନ କରିବାକୁ ଚେଷ୍ଟା କରନ୍ତୁ ନାହିଁ ସେମାନଙ୍କ ଉତ୍ତର ଭିନ୍ନ ବି ହୋଇପାରେ ।

Question	Options	Answer	Question	Options	Answer
weai_int_alone<br>14.1 Ability to be interviewed alone:<single answer><br>ଏକାକୀ ସାକ୍ଷାତକାରରେ ଭାଗ ନେବା ପାଇଁ ଦକ୍ଷ (ଗୋଟିଏ ଉତ୍ତର ଲେଖନ୍ତୁ)	1. Alone/ଏକୁଟିଆ<br>2. With adult females present/ ଅନ୍ୟାନ୍ୟ ଉପସ୍ଥିତ ବୟସ୍କ ମହିଳାଙ୍କ ସହିତ<br>3. With adult males present/ଅନ୍ୟାନ୍ୟ ଉପସ୍ଥିତ ବୟସ୍କ ପୁରୁଷଙ୍କ ସହିତ<br>4. With adults mixed sex present/ଚିକିତ୍ସା<br>5. With children present/ଉପସ୍ଥିତ ପିଲାମାନଙ୍କ ସହିତ<br>6. With adults mixed sex and children present/ଉପସ୍ଥିତ ମହିଳା, ପୁରୁଷ ଓ ପିଲାମାନଙ୍କ ସହିତ				
weai_asset_land<br>14.2 Does your household own any agricultural land?<br>ଆପଣଙ୍କ ପରିବାରର ନିଜର ଚାଷ ଜମି ଅଛି କି ?<br>Note: Note that this does not include land of extended family like grandfather if the extended family is not a household member.<br>ମନେରଖନ୍ତୁ:ଏଥିରେ ବର୍ଦ୍ଧିତ ପରିବାରର ସଦସ୍ୟ ବା ଜେଜେବାପା ସମ୍ପୃକ୍ତ ହେବେ ନାହିଁ ଯଦି ସେମାନେ ଏହି ପରିବାରର ସଦସ୍ୟ ନୁହନ୍ତି।	1. Yes, has legal ownership with Record of Rights (RoRs)/ ହଁ,ପଟ୍ଟା ଅଛି<br>2. Yes, has a share of land but in ancestral name/ ହଁ,ଭାଗ ଅଛି କିନ୍ତୁ ପୈତୃକଙ୍କ ନାମରେ ରହିଛି<br>3. Yes, has a share of land but no record/ ହଁ,ଭାଗ ଅଛି କିନ୍ତୁ କାଗଜପତ୍ର ନାହିଁ<br>0. No/ନା<br>→*If no, skip to Q14.3*	Record all that apply	weai_asset_landagri_own<br>14.2.1. Do you personally (jointly or solely) own any of the agriculture land that your household has? ପରିବାରର ଯେଉଁ ଜମି ଅଛି ତାହା ଆପଣଙ୍କ ନାମରେ ଅଛି କି(ଏକାକୀ ବା ମିଳିତ ଭାବରେ)?	Yes, solely/ହଁ, ଏକାକୀ---------1<br>Yes, jointly/ ହଁ,ମିଳିତ ଭାବେ---2<br>No/ନା------------------------0	

Question	Options	Answer	Question	Options	Answer
weai_asset_land<br>14.3 Does your household own any land not used for agriculture (pieces/plots, residential or commercial land)?<br>ଆପଣଙ୍କ ପରିବାରର ଏପରି କିଛି ଜମି ଅଛିକି, ଯାହାଚାଷକାମ ନିମନ୍ତେ ବ୍ୟବହାର ହେଉନାହିଁ ? (ପ୍ଲଟ, ଗୃହ କିମ୍ବା ବ୍ୟବସାୟ ଭିତ୍ତିକ ଜମି)?<br>Note: Note that this does not include land of extended family like grandfather if the extended family is not a household member.	1. Yes, has legal ownership with Record of Rights (RoRs)/ହଁ,ପଟ୍ଟା ଅଛି<br>2. Yes, has a share of land but in ancestral name/ହଁ,ଭାଗ ଅଛି କିନ୍ତୁ ପୈତୃକଙ୍କ ନାମରେ ରହିଛି<br>3. Yes, has a share of land but no record/ହଁ,ଭାଗ ଅଛି କିନ୍ତୁ କାଗଜପତ୍ର ନାହିଁ<br>0. No/ନା<br>→*If no, skip to Q14.4*	Record all that apply<br>☐<br>☐<br>☐	weai_asset_land_ot_own<br>14.3.1 Do you personally (jointly or solely) own any other land not used for agriculture (pieces/plots, residential or commercial land)<br>ଆପଣଙ୍କ ନାମରେ ଆଉ ଅଛି ଜମି ଅଛି କି ଯାହା ଚାଷ ପାଇଁ ବ୍ୟବହାର ହଉ ନାହିଁ(ଏକାକୀ ବା ମିଳିତ ଭାବରେ)?	Yes, solely/ହଁ, ଏକାକୀ---------1<br>Yes, jointly/ ହଁ,ମିଳିତ ଭାବେ---2<br>No/ନା------------------------0	☐
weai_asset_house<br>14.4 Do you, your household, or anyone in your household currently own a house or other structures?<br>ବର୍ତମାନ ଆପଣ, କିମ୍ବା ଆପଣଙ୍କ ପରିବାରର ଅନ୍ୟ କୌଣସି ସଦସ୍ୟଙ୍କରଘର ଅଛି କି(ଗୁହାଳ,ଖଳା ଘର)?	Yes/ହଁ----1<br>No/ନା----0<br>→*If no, skip to Q14.5*	☐	weai_asset_house_own<br>14.4.1 Do you own any of the item, either solely or jointly with any other person?<br>ଏହି ଘର ବା ଗୁହାଳ,ଖଳା ଘର ଆପଣଙ୍କ ନାମରେ ଅଛି କି?(ଏକାକୀ ବା ମିଳିତ ଭାବରେ)	Yes, solely/ହଁ, ଏକାକୀ---------1<br>Yes, jointly/ ହଁ,ମିଳିତ ଭାବେ---2<br>No/ନା------------------------0	☐
weai_asset_livestockl<br>14.5 Do you, your household, or anyone in your household currently own large livestock (e.g. oxen, cattle, buffalo)?<br>ଏବେ ଆପଣଙ୍କର କିମ୍ବା ଆପଣଙ୍କ ପରିବାରର ବଡ ଆକାରର ଗୃହପାଳିତ ପଶୁଅଛନ୍ତି କି ଯେପରୀ ବଳଦ,ଗାଈ,ମଇଁଷୀ?	Yes/ହଁ----1<br>No/ନା----0<br>→*If no, skip to Q14.6*	☐	weai_asset_livestockl_own<br>14.5.1 Do you own any of the item, either solely or jointly with any other person?<br>ଆପଣଙ୍କର ନିଜେ ଏକୁଟିଆ ହେଉ କିମ୍ବା ମିଳିତ ଭାବରେ ହଉ ଏହି ପଶୁ ଗୁଡିକର ମାଲିକ ଅଟନ୍ତି କି?	Yes, solely/ହଁ, ଏକାକୀ---------1<br>Yes, jointly/ ହଁ,ମିଳିତ ଭାବେ---2<br>No/ନା------------------------0	☐
weai_asset_livestocks<br>14.6 Do you, your household, or anyone in your household currently own small livestock (goats, pigs, sheep, chickens, ducks, pigeons)?<br>ଏବେ ଆପଣଙ୍କର କିମ୍ବା ଆପଣଙ୍କ ପରିବାରର ଛୋଟ ଆକାରରପଶୁପକ୍ଷୀ ଅଛନ୍ତି କି ଯେପରି ଛେଳି,ଘୁଷୁରି,ମେଣ୍ଢା,କୁକୁଡା,ବତକ,ପାରା?	Yes/ହଁ----1<br>No/ନା----0<br>→*If no, skip to Q14.7*	☐	weai_asset_livestocks_own<br>14.6.1 Do you own any of the item, either solely or jointly with any other person?<br>ଆପଣ ନିଜେ ଏକୁଟିଆ ହେଉ କିମ୍ବା ମିଳିତ ଭାବରେ ହଉ ଏହି ପଶୁପକ୍ଷୀ ଗୁଡିକର ମାଲିକ ଅଟନ୍ତି କି?	Yes, solely/ହଁ, ଏକାକୀ---------1<br>Yes, jointly/ ହଁ,ମିଳିତ ଭାବେ---2<br>No/ନା------------------------0	☐

Question	Options	Answer	Question	Options	Answer
weai_asset_equip_mechanised<br>14.7 Do you, your household, or anyone in your household currently own mechanised farm equipment? (e.g. tractor, power tiller, treadle pump)?<br>ବର୍ତମାନ ଆପଣକିମ୍ବା ଆପଣଙ୍କ ପରିବାରର ଚାଷ ନିମନ୍ତେ ମେଶିନ(ଯନ୍ତ୍ର ଚାଳିତ) ଅଛି କି ଯଥା; ଟ୍ରାକ୍ଟର, ପାୱାର ଟିଲର, ଟ୍ରିଡଲ ପମ୍ପ?	Yes/ହଁ----1<br>No/ନା----0<br>→*If no, skip to Q14.8*	☐	weai_asset_equip_mechanised_own<br>14.7.1 Do you own any of the item, either solely or jointly with any other person?<br>ଆପଣ ନିଜେ ଏକୁଟିଆ ହଉ କିମ୍ବା ମିଳିତ ଭାବରେ ହଉ ଏହି ମେଶିନ ଗୁଡିକର ମାଲିକ ଅଟନ୍ତି କି ?	Yes, solely/ହଁ, ଏକାକୀ---------1<br>Yes, jointly/ ହଁ,ମିଳିତ ଭାବେ---2<br>No/ନା------------------------0	☐
weai_asset_equip_nonmechanised<br>14.8 Do you, your household, or anyone in your household currently own farm non-mechanised equipment? (e.g. hand tools or animal-drawn plough or cart)<br>ବର୍ତମାନ ଆପଣକିମ୍ବା ଆପଣଙ୍କ ପରିବାରର ଚାଷନିମନ୍ତେଅଣ-ଯାନ୍ତ୍ରିକ ଉପକରଣ ଅଛି କି ?(ଯେପରି ହଳ, ଲଙ୍ଗଳ କିମ୍ବା ଶଗଡ)	Yes/ହଁ----1<br>No/ନା----0<br>→*If no, skip to Q14.9*	☐	weai_asset_farm nonmecha equip_own<br>14.8.1 Do you own any of the item, either solely or jointly with any other person?<br>ଆପଣ ନିଜେ ଏକୁଟିଆ ହଉ କିମ୍ବା ମିଳିତ ଭାବରେ ହଉ ଏହି ଜିନିଷ ଗୁଡିକର ମାଲିକ ଅଟନ୍ତି କି?	Yes, solely/ହଁ, ଏକାକୀ---------1<br>Yes, jointly/ ହଁ,ମିଳିତ ଭାବେ---2<br>No/ନା------------------------0	☐
weai_asset_equip_ business<br>14.9 Do you, your household, or anyone in your household currently own any nonfarm business equipment (solar panels used for recharging, sewing machine, brewing equipment, fryers)?<br>ବର୍ତମାନ ଆପଣକିମ୍ବା ଆପଣଙ୍କ ପରିବାରର ଅନ୍ୟ କୌଣସି ସଦସ୍ୟଙ୍କର ଚାଷ ଛଡା ଅନ୍ୟାନ୍ୟ ବୈବସାୟିକ ଉପକରଣର ଅଛି କି ? (ଯଥା; ସୋଲାର ପ୍ୟାନେଲ,ସିଲେଇ ମେସିନ, ମଦ୍ୟ ପ୍ରସ୍ତୁତ ଉପକରଣ ଓ ଭଜା ଭଜି କରିବା ଉପକରଣ)	Yes/ହଁ----1<br>No/ନା----0<br>→*If no, skip to Q14.10*	☐	weai_asset__equip_ business_own<br>14.9.1 Do you own any of the item, either solely or jointly with any other person?<br>ଆପଣ ନିଜେ ଏକୁଟିଆ ହଉ କିମ୍ବା ମିଳିତ ଭାବରେ ହଉ ଏହି ବୈବସାୟିକ ଉପକରଣର ମାଲିକ ଅଟନ୍ତି କି?	Yes, solely/ହଁ, ଏକାକୀ---------1<br>Yes, jointly/ ହଁ,ମିଳିତ ଭାବେ---2<br>No/ନା------------------------0	☐

Question	Options	Answer	Question	Options	Answer
weai_asset_highcost_durables<br>14.10 Do you, your household, or anyone in your household currently own any high cost consumer durables e.g. refrigerator, TV, sofa, expensive bed etc.?<br>ବର୍ତମାନ ଆପଣକିମ୍ବା ଆପଣଙ୍କ ପରିବାରର ଅନ୍ୟ କୌଣସି ସଦସ୍ୟଙ୍କର ଦାମୀ ଜିନିଷ ଅଛି କି?<br>(ଯଥା;ଫ୍ରିଜ,ଟିଭି,ସୋଫା,ଦାମୀ ବିଛଣା ଇତ୍ୟାଦି)	Yes/ହଁ----1<br>No/ନା----0<br>→*If no, skip to Q14.11*	☐	weai_asset_highcost_durables_own<br>14.10.1 Do you own any of the item, either solely or jointly with any other person?<br>ଆପଣ ନିଜେ ଏକୁଟିଆ ହଉ କିମ୍ବା ମିଳିତ ଭାବରେ ହଉ ଏହି ଦାମୀ ଜିନିଷର ମାଲିକ ଅଟନ୍ତି କି?	Yes, solely/ହଁ, ଏକାକୀ---------1<br>Yes, jointly/ ହଁ,ମିଳିତ ଭାବେ---2<br>No/ନା------------------------0	☐
weai_asset_lowcost_durables<br>14.11 Do you, your household, or anyone in your household currently own any low cost consumer durables e.g. mattress (gadda), cot (charpai), radio, fan, watch or clock, or cookware?<br>ବର୍ତମାନ ଆପଣଆପଣଙ୍କ ଘରେ ଅଳ୍ପ ଦାମରଜିନିଷ ରଖିଛନ୍ତି କି ? (ଯଥା;ଗଦି,ଖଟ,ରେଡିଓ,ପଙ୍ଖା,କାନ୍ଥ ଘଣ୍ଟା, ହାତ ଘଣ୍ଟା କିମ୍ବା ରୋଷେଇ ଉପକରଣ ଇତ୍ୟାଦି)	Yes/ହଁ----1<br>No/ନା----0<br>→*If no, skip to Q14.12*	☐	weai_asset_lowcost_durables_own<br>14.11.1 Do you own any of the item, either solely or jointly with any other person?<br>ଆପଣ ନିଜେ ଏକୁଟିଆ ହଉ କିମ୍ବା ମିଳିତ ଭାବରେ ହଉ ଏହି ଜିନିଷ ଗୁଡିକର ମାଲିକ ଅଟନ୍ତି କି?	Yes, solely/ହଁ, ଏକାକୀ---------1<br>Yes, jointly/ ହଁ,ମିଳିତ ଭାବେ---2<br>No/ନା------------------------0	☐
weai_asset_ jewellery<br>14.12 Do you, your household, or anyone in your household currently own any metal jewellery (gold, silver, brass or white metal)?<br>ବର୍ତମାନ ଆପଣକିମ୍ବା ଆପଣଙ୍କ ପରିବାରର କୌଣସି ସଦସ୍ୟଙ୍କର ଗହଣା ଅଛି କି ? (ଯଥା;ସୁନା,ରୁପା,ପିତ୍ତଳ,ଧଳା ଧାତୁ ଇତ୍ୟାଦି)	Yes/ହଁ----1<br>No/ନା----0<br>→*If no, skip to Q14.13*	☐	weai_asset_ jewellery_own<br>14.12.1 Do you own any of the item, either solely or jointly with any other person?<br>ଆପଣ ନିଜେ ଏକୁଟିଆ ହଉ କିମ୍ବା ମିଳିତ ଭାବରେ ହଉଗହଣାର ମାଲିକ ଅଟନ୍ତି କି?	Yes, solely/ହଁ, ଏକାକୀ---------1<br>Yes, jointly/ ହଁ,ମିଳିତ ଭାବେ---2<br>No/ନା------------------------0	☐
weai_asset_phone<br>14.13 Do you, your household, or anyone in your household currently own a mobile phone?<br>ବର୍ତମାନ ଆପଣକିମ୍ବା ଆପଣଙ୍କ ପରିବାରର କୌଣସି ସଦସ୍ୟଙ୍କ ମୋବାଇଲ ଫୋନ ଅଛି କି ?	Yes/ହଁ----1<br>No/ନା----0<br>→*If no, skip to Q15.1*	☐	weai_asset_phone_own<br>14.13.1 Do you own any of the item, either solely or jointly with any other person?<br>ଆପଣ ନିଜେ ଏକୁଟିଆ ହଉ କିମ୍ବା ମିଳିତ ଭାବରେ ହଉ ମୋବାଇଲ ଫୋନର ମାଲିକ ଅଟନ୍ତି କି?	Yes, solely/ହଁ, ଏକାକୀ---------1<br>Yes, jointly/ ହଁ,ମିଳିତ ଭାବେ---2<br>No/ନା------------------------0	☐

15.0 HOUSEHOLD DECISION-MAKING / ପରିବାରର ନିଷ୍ପତ୍ତି ଗ୍ରହଣ

Decision-Making: Now I'd like to ask you some questions about your participation in certain types of work activities and on making decisions on various aspects of household life/ନିଷ୍ପତ୍ତି ଗ୍ରହଣ – ବର୍ତ୍ତମାନ ମୁଁ ଆପଣଙ୍କୁ ଆପଣଙ୍କର କିଛି କାର୍ଯ୍ୟକଳାପରେ ଭାଗୀଦାରୀ ଓ ଆପଣଙ୍କ ପରିବାରର ବିଶେଷ ପ୍ରସଙ୍ଗ ବିଷୟରେ ନିଷ୍ପତ୍ତି ଗ୍ରହଣ ସମ୍ବନ୍ଧୀୟ ପ୍ରଶ୍ନ ପଚାରିବି ।

Variable name	Question	Options	Answer
weai_careseeking_decide	15.1 Who usually decides whether you can or cannot go to hospital / clinic / doctor (to seek health service)<br>ଆପଣ ଡାକ୍ତରଖାନା/କ୍ଲିନିକ (ସ୍ୱାସ୍ଥ୍ୟ ସେବା ପାଇଁ) ଯିବେ ନ ଯିବେ କିଏ ନିଷ୍ପତି ନିଅନ୍ତି?	Tick all that apply/ଉପଯୁକ୍ତ ଉତ୍ତର ଗୁଡିକରେ ଚିହ୍ନ ଦିଅନ୍ତୁ<br>☐ Self/ନିଜେ = 1<br>☐ Spouse/ସ୍ୱାମୀ/ସ୍ତ୍ରୀ = 2<br>☐ Other HH member/ପରିବାରର ଅନ୍ୟ ସଦସ୍ୟ = 3<br>☐ Other non-HH member /ବାହାରଲୋକ = 4<br>☐ Not applicable/ଉପଯୁକ୍ତ ନୁହେଁ = 98	
weai_careseeking_alone	15.2 Does your husband/partner or other household member object to you going alone to hospital / clinic / doctor (to seek health service)?<br>ଆପଣଙ୍କୁ ଏକୁଟିଆ ଡାକ୍ତରଖାନା/କ୍ଲିନିକ/ଡାକ୍ଟରପାଖକୁ(ସ୍ୱାସ୍ଥ୍ୟ ସେବା ପାଇଁ)ଯିବାପାଇଁ ଆପଣଙ୍କର ସ୍ୱାମୀ/ପରିବାରର ଅନ୍ୟ କୌଣସି ସଦସ୍ୟ ମନାକରନ୍ତି କି?	Yes/ହଁ-------------------------------- 1<br>No/ନା--------------------------------0	☐
weai_market_decide	15.3 Who usually decides whether you can or cannot go to market / haat / bazaar<br>ଆପଣ ବଜାର/ହାଟକୁ ଯିବେ କି ନଯିବେ ସାଧାରଣତ କିଏ ନିଷ୍ପତି ନିଅନ୍ତି?	Tick all that apply/ଉପଯୁକ୍ତ ଉତ୍ତର ଗୁଡିକରେ ଚିହ୍ନ ଦିଅନ୍ତୁ<br>☐ Self/ନିଜେ = 1<br>☐ Spouse/ସ୍ୱାମୀ/ସ୍ତ୍ରୀ = 2<br>☐ Other HH member/ପରିବାରର ଅନ୍ୟ ସଦସ୍ୟ = 3<br>☐ Other non-HH member /ବାହାରଲୋକ = 4<br>☐ Not applicable/ଉପଯୁକ୍ତ ନୁହେଁ = 98	
weai_market_alone	15.4 Does your husband/partner or other household member object to you going alone to market / haat / bazaar?<br>ଆପଣଙ୍କୁ ଏକୁଟିଆ ବଜାର/ହାଟକୁ ଯିବାପାଇଁଆପଣଙ୍କ ସ୍ୱାମୀ /ପରିବାବର ଅନ୍ୟ କୌଣସି ସଦସ୍ୟ ଆପଣଙ୍କୁ ମନା କରନ୍ତି କି ?	Yes/ହଁ-------------------------------- 1<br>No/ନା--------------------------------0	☐

PRODUCTIVE DECISION-MAKING/ ଉତ୍ପାଦନ ଭିତ୍ତିକ ନିଷ୍ପତ୍ତି ଗ୍ରହଣ

**Now I'd like to ask you some questions about your participation in certain types of work activities and on making decisions on various aspects of household life/**<br>ଉତ୍ପାଦନ ଭିତ୍ତିକ ନିଷ୍ପତ୍ତି ଗ୍ରହଣ – ବର୍ତ୍ତମାନ ମୁଁ ଆପଣଙ୍କୁ ଆପଣଙ୍କ ପରିବାରର କାମଦାମରେ ଭାଗୀଦାରୀ ଓଏହାରନିଷ୍ପତ୍ତି ନେବା ବିଷୟରେ ପଚାରିବି ।<br>Did you yourself participate in [activity] in the past 3 agricultural seasons (June 2015 to May 2016)?ଗତ ବର୍ଷ ବର୍ଷାଦିନିଆ ଫସଲ ଠାରୁ ଖରାଟିଆ ଫସଲ ପର୍ଯ୍ୟନ୍ତ(ଜୁନ 2015 ରୁ ମେ 2016 ପର୍ଯ୍ୟନ୍ତ) ହୋଇଥିବା ଚାଷକାମରେ ଆପଣ ଭାଗ ନେଇଥିଲେ କି **?**			How much input or influence did you have in making decisions about [activity], including any decisions about income from [activity]? *Input means gave an opinion, was asked an opinion, or was able to influence an action.* Probe: Did your spouse or others in the household consult / ask with you when making decisions about the activity or what to do with the income from that activity?<br>ପରିବାରର ଆର୍ଥିକ ଆୟ ସମ୍ବନ୍ଧୀୟ ନିଷ୍ପତ୍ତି ନେବାରେ ଆପଣଙ୍କକଥା କେତେ ରହିଥିଲା ଏହାର ଅର୍ଥ ଆପଣଙ୍କୁ ପଚରା ଯାଏକି, ଆପଣଙ୍କ ମତାମତକୁ ବିଚାରକୁ ନିଆଯାଏ କି ? ଦର୍ଶାନ୍ତୁ: ଆପଣଙ୍କ ସ୍ୱାମୀ କିମ୍ବା ପରିବାରର ଅନ୍ୟାନ୍ୟ ସଦସ୍ୟ ପରିବାରର ଆର୍ଥିକ ଆୟ ସମ୍ବନ୍ଧୀୟ ନିଷ୍ପତ୍ତି ନେବାବେଳେ ଆପଣଙ୍କ ସହିତ ପରାମର୍ଶ କରନ୍ତି କି ?	
**Activity**	**Options**	**Answer**	**Options**	**Answer**
15.5 Food production for household consumption: these are crops, livestock or fish etc. that are grown primarily for household food consumption ପରିବାର ନିମନ୍ତେ ଖାଦ୍ୟ ଉତ୍ପାଦନ(ଚାଷ,ପଶୁପାଳନ କିମ୍ବା ମାଛ ଚାଷ)।	Yes/ହଁ---------------------- 1<br>No/ନା----------------------0<br>→ go to next activity ପରବର୍ତ୍ତୀ ପର୍ଯ୍ୟାୟକୁ ଯାଆନ୍ତୁ	☐	No input or input in few decisions/ କୌଣସି ଅଂଶଗ୍ରହଣ ନାହିଁ ବା ଅଳ୍ପକିଛି ନିଷ୍ପତ୍ତିରେ ଅଂଶଗ୍ରହଣ-------- 1<br>Input into some decisions/ କିଛି ନିଷ୍ପତ୍ତିରେ ଅଂଶଗ୍ରହଣ-- 2<br>Input into most or all decisions/ ଅଧିକାଂଶ ବା ସମସ୍ତ ନିଷ୍ପତ୍ତିରେ ଅଂଶଗ୍ରହଣ-------------------------- 3<br>No decision made/ କୌଣସି ନିଷ୍ପତ୍ତି ନିଅନ୍ତି ନାହିଁ -- 98	☐
15.6 Cash crop farming: crops grown primarily for income generation. These can be food or non-food items, and may also be used partly (but not mainly) for home consumption. ଅର୍ଥକାରୀ:ଯେଉଁ ଫସଲ ମୁଖ୍ୟତ ଆୟ ସୃଷ୍ଟି କରେ ସେଗୁଡିକ ଖାଦ୍ୟ ବା ଅନ୍ୟ ଖାଦ୍ୟ ହୋଇପାରେ ।	Yes/ହଁ---------------------- 1<br>No/ନା----------------------0<br>→ go to next activity ପରବର୍ତ୍ତୀ ପର୍ଯ୍ୟାୟକୁ ଯାଆନ୍ତୁ	☐	No input or input in few decisions/ କୌଣସି ଅଂଶଗ୍ରହଣ ନାହିଁ ବା ଅଳ୍ପକିଛି ନିଷ୍ପତ୍ତିରେ ଅଂଶଗ୍ରହଣ-------- 1<br>Input into some decisions/ କିଛି ନିଷ୍ପତ୍ତିରେ ଅଂଶଗ୍ରହଣ-- 2<br>Input into most or all decisions/ ଅଧିକାଂଶ ବା ସମସ୍ତ ନିଷ୍ପତ୍ତିରେ ଅଂଶଗ୍ରହଣ-------------------------- 3<br>No decision made/ କୌଣସି ନିଷ୍ପତ୍ତି ନିଅନ୍ତି ନାହିଁ -- 98	☐
15.7 Livestock raising (large and small livestock)<br>ପଶୁପାଳନ(ବଡ ଏବଂ ସାନ)	Yes/ହଁ---------------------- 1<br>No/ନା----------------------0<br>→ go to next activity ପରବର୍ତ୍ତୀ ପର୍ଯ୍ୟାୟକୁ ଯାଆନ୍ତୁ	☐	No input or input in few decisions/ କୌଣସି ଅଂଶଗ୍ରହଣ ନାହିଁ ବା ଅଳ୍ପକିଛି ନିଷ୍ପତ୍ତିରେ ଅଂଶଗ୍ରହଣ-------- 1<br>Input into some decisions/ କିଛି ନିଷ୍ପତ୍ତିରେ ଅଂଶଗ୍ରହଣ-- 2<br>Input into most or all decisions/ ଅଧିକାଂଶ ବା ସମସ୍ତ ନିଷ୍ପତ୍ତିରେ ଅଂଶଗ୍ରହଣ-------------------------- 3<br>No decision made/ କୌଣସି ନିଷ୍ପତ୍ତି ନିଅନ୍ତି ନାହିଁ -- 98	☐
15.8 Non-farm economic activities: this includes things like running a small business, self-employment, buy-and-sell.<br>ଚାଷ ଛଡା ଅନ୍ୟ କିଛି କାମ : ଯେମିତିକି ଛୋଟ ଦୋକାନ,ବିକା କିଣା,ଅନ୍ୟ କିଛି କାମ କରିବା ।	Yes/ହଁ---------------------- 1<br>No/ନା----------------------0<br>→ go to next activityପରବର୍ତ୍ତୀ ପର୍ଯ୍ୟାୟକୁ ଯାଆନ୍ତୁ	☐	No input or input in few decisions/ କୌଣସି ଅଂଶଗ୍ରହଣ ନାହିଁ ବା ଅଳ୍ପକିଛି ନିଷ୍ପତ୍ତିରେ ଅଂଶଗ୍ରହଣ-------- 1<br>Input into some decisions/ କିଛି ନିଷ୍ପତ୍ତିରେ ଅଂଶଗ୍ରହଣ-- 2<br>Input into most or all decisions/ ଅଧିକାଂଶ ବା ସମସ୍ତ ନିଷ୍ପତ୍ତିରେ ଅଂଶଗ୍ରହଣ-------------------------- 3<br>No decision made/ କୌଣସି ନିଷ୍ପତ୍ତି ନିଅନ୍ତି ନାହିଁ -- 98	☐
15.9 Minor household expenditures e.g. food for daily consumption or other household needs<br>ପରିବାରର ନୀତି ଦିନିଆ ଖର୍ଚ୍ଚଯେପରି ଖାଇବା ଖର୍ଚ୍ଚ ଓ ଅନ୍ୟ ସବୁ ଖର୍ଚ୍ଚ?			No input or input in few decisions/ କୌଣସି ଅଂଶଗ୍ରହଣ ନାହିଁ ବା ଅଳ୍ପକିଛି ନିଷ୍ପତ୍ତିରେ ଅଂଶଗ୍ରହଣ-------- 1<br>Input into some decisions/ କିଛି ନିଷ୍ପତ୍ତିରେ ଅଂଶଗ୍ରହଣ-- 2<br>Input into most or all decisions/ ଅଧିକାଂଶ ବା ସମସ୍ତ ନିଷ୍ପତ୍ତିରେ ଅଂଶଗ୍ରହଣ-------------------------- 3<br>No decision made/ କୌଣସି ନିଷ୍ପତ୍ତି ନିଅନ୍ତି ନାହିଁ -- 98	☐

16.0 ACCESS TO CREDIT/ଋଣ ପାଇଁ ସୁବିଧା

Question	Options	Answer	Variable name	Question	Answer
Access to credit: “Next I’d like to ask about your household’s experience with borrowing money or other items in the past 12 months.”/ଋଣ ପାଇଁ ସୁବିଧା :ଗତ 12 ମାସ ଭିତରେ ଘର ପାଇଁ ଟଙ୍କା ବା ଜିନିଷ ଧାରା ଉଧାର କରିବାର ଅନୁଭୂତି ବିଷୟରେ ମୁ ଆପଣଙ୍କୁ ପଚାରିବାକୁ ଚାହୁଁଛି ।					
weai_credit_formal<br>16.1 Have you taken any loans or borrowed cash/in-kind from a formal institution, such as financial institution, bank or a non-governmental organisation in the past 12 months? ଗତ 12 ମାସରେ ଆପଣକୌଣସି ବ୍ୟାଙ୍କ ରୁ କିମ୍ବା ବେସରକାରୀ ଜଗାରୁ ଋଣ ଆଣିଛନ୍ତି କି?	<single answer/ଗୋଟିଏ ଉତ୍ତର ଲେଖନ୍ତୁ><br>Yes, in my own name/ହଁ, ମୋ ନିଜ ନାମରେ ---1<br>Yes, jointly/ହଁ, ମିଳିତ ଭାବରେ-------------------2<br>No/ନା---0<br>Don’t know/ଜାଣିନାହିଁ---------------------------98<br>→If 0 or 98, go to 17.2	☐	weai_credit_formal_decide	16.1.1 If yes, who decided how to spend this credit most of the time?<br>ଯଦିହଁ,ଅଧିକାଂଶ ସମୟ ସେ ଅର୍ଥକୁ କିପରି ଖର୍ଚ୍ଚ କରାଯିବ ତାର ନିଷ୍ପତି କିଏ ନିଅନ୍ତି ?<br><multiple answer/ବହୁ ଉତ୍ତର ସମ୍ଭବ><br>Self/ନିଜେ--------------------------- 1<br>Spouse/ସ୍ୱାମୀ/ସ୍ତ୍ରୀ-------------------- 2<br>Other HH member/ପରିବାରର ଅନ୍ୟ ସଦସ୍ୟ-- 3<br>Other non-HH member/ଅନ୍ୟ ବାହାର ବ୍ୟକ୍ତି---- 4<br>Not applicable/ଉପଯୁକ୍ତ ନୁହେଁ---- 98	☐ ☐ ☐ ☐
weai_credit_group<br>16.2 Have you taken any loans or borrowed cash/in-kind from a group based micro-finance or lending, including informal saving or credit community groups or societies ଗତ 12 ମାସ ମଧ୍ୟରେ ଆପଣ କୌଣସି ମାଇକ୍ରୋଫାଇନାନ୍ସ ବା ଘାରୋଇ ସଞ୍ଚୟକାରୀ ସଙ୍ଘ ଗୋଷ୍ଠୀ ସ୍ତରର ସୋସାଇଟୀ ଠାରୁ ଋଣ କରିଛନ୍ତି କି?	<single answer/ଗୋଟିଏ ଉତ୍ତର ଲେଖନ୍ତୁ><br>Yes, in my own name/ହଁ, ମୋ ନିଜ ନାମରେ ---1<br>Yes, jointly/ହଁ, ମିଳିତ ଭାବରେ-------------------2<br>No/ନା---0<br>Don’t know/ଜାଣିନାହିଁ---------------------------98<br>→If 0 or 98, go to 17.3	☐	weai_credit_group_decide	16.2.1 If yes, who decided how to spend this credit most of the time?<br>ଯଦି ହଁ,ଅଧିକାଂଶ ସମୟ ସେ ଅର୍ଥକୁ କିପରି ଖର୍ଚ୍ଚ କରାଯିବ ତାର ନିଷ୍ପତି କିଏ ନିଅନ୍ତି ?<br><multiple answer/ବହୁ ଉତ୍ତର ସମ୍ଭବ><br>Self/ନିଜେ--------------------------- 1<br>Spouse/ସ୍ୱାମୀ/ସ୍ତ୍ରୀ-------------------- 2<br>Other HH member/ପରିବାରର ଅନ୍ୟ ସଦସ୍ୟ-- 3<br>Other non-HH member/ଅନ୍ୟ ବାହାର ବ୍ୟକ୍ତି---- 4<br>Not applicable/ଉପଯୁକ୍ତ ନୁହେଁ---- 98	☐ ☐ ☐ ☐
weai_credit_informal<br>16.3 Have you taken any loans or borrowed cash/in-kind from an informal lender, including local middleman (mahajan), or friends, neighbours or relatives<br>ଆପଣ କୌଣସି ଘରୋଇ ସୁଧ ବେପାରୀ,ମହାଜନ, ସାଙ୍ଗ ବା ବନ୍ଧୁବାନ୍ଧବଙ୍କ ଠାରୁ ଋଣ କରିଛନ୍ତି କି ?	<single answer/ଗୋଟିଏ ଉତ୍ତର ଲେଖନ୍ତୁ><br>Yes, in my own name/ହଁ, ମୋ ନିଜ ନାମରେ ---1<br>Yes, jointly/ହଁ, ମିଳିତ ଭାବରେ-------------------2<br>No/ନା---0<br>Don’t know/ଜାଣିନାହିଁ---------------------------98<br>→If 0 or 98, go to 17.4	☐	weai_credit_informal_decide	16.3.1 If yes, who decided how to spend this credit most of the time?<br>ଯଦି ହଁ ,ଅଧିକାଂଶ ସମୟ ସେ ଅର୍ଥକୁ କିପରି ଖର୍ଚ୍ଚ କରାଯିବ ତାର ନିଷ୍ପତି କିଏ ନିଅନ୍ତି?<br><multiple answer/ବହୁ ଉତ୍ତର ସମ୍ଭବ><br>Self/ନିଜେ--------------------------- 1<br>Spouse/ସ୍ୱାମୀ/ସ୍ତ୍ରୀ-------------------- 2<br>Other HH member/ପରିବାରର ଅନ୍ୟ ସଦସ୍ୟ-- 3<br>Other non-HH member/ଅନ୍ୟ ବାହାର ବ୍ୟକ୍ତି---- 4<br>Not applicable/ଉପଯୁକ୍ତ ନୁହେଁ---- 98	☐ ☐ ☐ ☐

Question	Options	Answer	Variable name	Question	Answer
credit_stress Only ask if any loans taken:<br>16.4 Based on your experience of taking a loan, would you have taken this / these loans in hindsight? ପୂର୍ବ ଋଣ ନେବା ଅନୁଭୂତିରୁ ଆପଣଙ୍କୁ ଲାଗୁଛି କି ଆପଣ ସେହି ଜାଗାରୁ ଆଉ ଥରେ ଋଣ ଆଣିବେ କି?<br>Probe: Do you feel like it was a good decision?<br>ଦର୍ଶାନ୍ତୁ: ଆପଣ ଭାବୁଛନ୍ତି କି ଏହା ଏକ ଭଲ ନିଷ୍ପତ୍ତି ?	Yes/ହଁ------------- 1<br>No/ନା------------- 0<br>Don't know / can't say/ଜାଣି ନାହିଁ/କହି ପାରିଲେନି<br>---------------------98	☐			

17.0 **TIME USE/ସମୟର ଉପଯୋଗ**

<u>**Instructions:**</u> If yesterday was a typical day ask the respondent about yesterday. If yesterday was atypical, but the day before typical, please ask the respondent to consider the day before's activities. If both days were atypical, then please ask the respondent to consider yesterday's activities.

ଯଦି ଗତକାଲି ଗୋଟିଏ ବିଶେଷ ଦିନ ହୋଇଥିବ ତେବେ ଉତ୍ତରଦାତାଙ୍କୁ ଗଲା କାଲି ବିଷୟରେ ପଚାରନ୍ତୁ । ଯଦି ଗଲା କାଲିର ପୂର୍ବଦିନଟି ବିଶେଷ ଦିନ ହୋଇ ଥାଏ ତେବେ ଉତ୍ତରଦାତାଙ୍କୁ ସେହି ଦିନର କାର୍ଯ୍ୟକଳାପ ବିଷୟରେ ପଚାରନ୍ତୁ।ଯଦି ଉଭୟ ୨ ଦିନ କୌଣସି ବିଶେଷ ଦିନ ହୋଇଥାଏ, ତେବେ ଉତ୍ତରଦାତାଙ୍କୁ ଗତକାଲି ବିଷୟରେ ପଚାରନ୍ତୁ ।

Please record a log of the activities for the individual in the last complete 24 hours (starting yesterday morning at 4 am, finishing 3:59 am of the current day). The time intervals are marked in 30 min intervals. <u>Mark one activity for each time period</u> by entering the corresponding activity code in the box.Check the box below if the respondent was caring for children while performing each activity.

Now I'd like to ask you about how you spent your time during the past 24 hours. We'll begin from yesterday morning, and continue through to this morning. This will be a detailed accounting. I'm interested in everything you did (i.e. resting, eating, personal care, work inside and outside the home, caring for children, cooking, shopping, socializing, etc.), even if it didn't take you much time.I'm particularly interested in agricultural activities such as farming, gardening, and livestock raising whether in the field or on the homestead. I'm also interested in how much time you spent caring for children, especially if it happened while you did some other activity (e.g., collecting water while carrying a child or cooking while watching after a sleeping child).

ଦୟାକରି ସମ୍ପୂର୍ଣ ୨୪ ଘଣ୍ଟାର କାର୍ଯ୍ୟକଳାପର ଏକ ସାରଣୀ ରଖନ୍ତୁ (ଗତକାଲି ରାତି ୪ଟାରୁ ଆରମ୍ଭହୋଇ ଆଜିର ୩.୫୯ ପର୍ଯ୍ୟନ୍ତ) ସମୟ ନିର୍ଦ୍ଧାରଣ ୩୦ ମିନିଟ ଅନ୍ତରରେ ରଖାଯାଇଅଛି । ପ୍ରତ୍ୟେକ କାର୍ଯ୍ୟକଳାପ ନିମନ୍ତେ ପୃଥକ ପୃଥକ ସମୟ ନିର୍ଦ୍ଧାରଣ ହୋଇଛି ।

ପ୍ରତ୍ୟେକ କାର୍ଯ୍ୟ ନିମନ୍ତେ ନିର୍ଦ୍ଧାରିତ କୋଡକୁ ନିମ୍ନରେ ଦିଆଯାଇଥିବା କୋଠରୀରେ ଲେଖନ୍ତୁ ଓ ଦେଖନ୍ତୁ ଏହି କାର୍ଯ୍ୟ କଲାବେଳେ ସେ ଶିଶୁର ଯତ୍ନ ନେଉଛନ୍ତି କି ?

ବର୍ତ୍ତମାନ ମୁଁ ଆପଣଙ୍କୁ ଗତ ୨୪ ଘଣ୍ଟା ମଧ୍ୟରେ ଆପଣ କେଉଁ କେଉଁ କାମ କରିଛନ୍ତି ସେ ବିଷୟରେ ପଚାରିବି । ଆମେ ଗତକାଲି ସକାଳରୁ ଆରମ୍ଭ କରିବା ଓ ଧୀରେ ଧୀରେ ଆଜି ସକାଳ ପର୍ଯ୍ୟନ୍ତ ଯିବା । ଏହା ଏକ ସମ୍ପୂର୍ଣ ବିବରଣୀ/ଆକଳନ ହେବ । ମୁଁ ଆପଣଙ୍କ ସବୁ ଟିକିନିଖି କାର୍ଯ୍ୟକଳାପ ଜାଣିବାକୁ ଚାହେଁ । ଯଥା: ବିଶ୍ରାମ ନେବା, ଖାଇବା, ନିଜର ଯତ୍ନ ନେବା, ଘରେ ଓ ବାହାରେ କାର୍ଯ୍ୟ କରିବା, ପିଲାମାନଙ୍କ ଯତ୍ନ ନେବା, ରୋଷେଇ କରିବା, ବଜାର କରିବା ଓ ସାମାଜିକ କାମ କରିବା। ଏଗୁଡିକରେ ମୁଁ ବେଶି ସମୟ ନେବି ନାହିଁ । ଆପଣଙ୍କ କୃଷି କାର୍ଯ୍ୟକଳାପ ଯଥା: ଚାଷ କରିବା, ବଗିଚା କରିବା, ପଶୁ ସମ୍ପଦ ପାଳନ କରିବା କାମ ଜାଣିବାରେ ମୁ ବେସୀ ଆଗ୍ରହୀ ଏହା ବିଲରେ ହେଉ ବା ଘରେ ହେଉ । ମୁଁ ଏହା ମଧ୍ୟ ଜାଣିବାକୁ ଚାହେଁ ଆପଣ ପିଲାଙ୍କ ଯତ୍ନ ନେବାରେ କେତେ ସମୟ ଦେଉଛନ୍ତି ବିଶେଷକରି ଆପଣ ଯେତେବେଳେ ଅନ୍ୟକିଛି କାର୍ଯ୍ୟ କରୁଛନ୍ତି ତା ସହିତ ପିଲାର ଯତ୍ନ କିପରି ନେଉଛନ୍ତି । ଯଥା: ପିଲାର ଯତ୍ନ ନେବାବେଳେ ପାଣି ଆଣିବା ବା ରୋଷେଇ କଲାବେଳେ ଶୋଇଲା ପିଲାପ୍ରତି ନଜର ରଖିବା ।

Variable name	Question	Answer
weai_time_yday<br>18.1 Was yesterday (the last 24 hours) a typical day where you worked (either at home or outside the home) about the same as usual? ଗତକାଲି (ଗତ 24 ଘଣ୍ଟା) ଏକ ବିଶେଷ ଦିନ ଥିଲା କି ଯେଉଁ ଦିନ ଆପଣ ଘରେ ହେଉ ବା ବାହାରେ ହେଉ ଅନ୍ୟ ଦିନ ଭଳି ସମାନ କାମ କରିଥିଲେ ?	Yes/ହଁ----------------- 1<br>→*Go to 18.3*<br>No/ନା---------------- 0<br>→*Go to 18.2*	☐
weai_time_daybefore<br>18.2 Was the day before yesterday a typical day where you worked (either at home or outside the home) about the same as usual? ପହରଦିନ ଏକ ବିଶେଷ ଦିନ ଥିଲା କି ଯେଉଁ ଦିନ ଆପଣ ଘରେ ହେଉ ବା ବାହାରେ ହେଉ ଅନ୍ୟ ଦିନ ଭଳି ସମାନ କାମ କରିଥିଲେ ?	Yes/ହଁ----------------- 1<br>→*Go to 18.3*<br>No/ନା---------------- 0<br>→*Go to 18.3*	☐

	**Night**		**Morning**	**Day**								
	04:00	05:00	06:00	07:00	08:00	09:00	10:00	11:00	12:00	13:00	14:00	15:00
Activity **(WRITE ACTIVITY CODE)**<br>(କାର୍ଯ୍ୟକଳାପର କୋଡ ଲେଖନ୍ତୁ)												
18.3Did you also care for children/ଆପଣ ପିଲାଙ୍କ ଯତ୍ନ ମଧ୍ୟ ନିଅନ୍ତି କି?<br>Yes/ହଁ--------------------- 1<br>No/ନା--------------------- 0												

	**Day**		**Evening**	**Night**								
	16:00	17:00	18:00	19:00	20:00	21:00	22:00	23:00	24:00	01:00	02:00	03:00
Activity **(WRITE ACTIVITY CODE)**<br>(କାର୍ଯ୍ୟକଳାପର କୋଡ ଲେଖନ୍ତୁ)												
Did you also care for children/ଆପଣ ପିଲାଙ୍କ ଯତ୍ନ ମଧ୍ୟ ନିଅନ୍ତି କି?<br>Yes/ହଁ--------------------- 1<br>No/ନା--------------------- 0												

A= Sleeping and resting/ଶୋଇବା ଏବଂ ବିଶ୍ରାମ ନେବା<br>B= Eating, drinking, or other relaxing leisure activities including social or religious activities/ଖାଇବା, ପିଇବା, ଫୁର୍ତ୍ତି କରିବା, ପୂଜା କରିବା<br>C= Personal care (e.g. dressing, showering)/ନିଜର ଯତ୍ନ (ଯଥା: ସଜବାଜ ହେବା, ଗାଧୋଇବା)<br>D= School / studies/ବିଦ୍ୟାଳୟ/ପଢିବା<br>E= Office / shop business work or other light non-farm wage labour e.g. barber, shop, blacksmith/ଅଫିସ/ଦୋକାନ ବ୍ୟବସାୟ ବା ଅନ୍ୟାନ୍ୟ ଅଣ-କୃଷି ଶ୍ରମ ଯଥା: ଭଣ୍ଡାରୀ, ଦୋକାନ, କମାର<br>F= Physically strenuous non-farm labour, e.g. wage labour like construction, work in mines or delivering heavy goods./ଶାରୀରିକ ବଳ ପ୍ରୟୋଗକରି ଅଣ-କୃଷି ଶ୍ରମ, ଯଥା: ଦିନ ମଜୁରିଆ ଯେପରି ନିର୍ମାଣ କାର୍ଯ୍ୟ, ଖଣିରେ କାମ କରିବା, ଭାରି ଜିନିଷ ଉଠାଇବା<br>G = Traveling / commuting/ଯାତ୍ରା କରିବା/ ଯିବା ଆସିବା କରିବା<br>H = Heavy / strenuous agricultural work (e.g. digging, hoeing, heavy lifting, spraying, threshing)/ଭାରି କୃଷିକାର୍ଯ୍ୟ ଯଥା: ଖୋଳିବା, ଭାରି ଜିନିଷ ଉଠାଇବା, ସ୍ପ୍ରେ କରିବା,ଧାନ କାଟିବା I	I = Mild / moderately strenuous agricultural work (e.g. bundling rice, harvesting, planting / transplanting, weeding, winnowing, ploughing with tractor or bullock)/ଅଳ୍ପ ଶ୍ରମଦ୍ୱାରା କୃଷିକାର୍ଯ୍ୟ(ଯଥା: ଧାନ ଗୋଛା କରିବା, ଅମଳ କରିବା, ଧାନ ରୋଇବା, ତଳି ପକାଇବା, ବଳଦ କିମ୍ବା ଟ୍ରାକ୍ଟର ଦ୍ୱାରା ହଳ କରିବା)<br>J = Livestock raising and fishpond culture/ପଶୁସମ୍ପଦ ପାଳନ କରିବା ଏବଂ ମାଛ ଚାଷ କରିବା<br>K = Foraging or hunting /ଶିକାର କରିବା<br>L = Collecting water / wood / other load/ ପାଣି/କାଠ/ ଅନ୍ୟନ ସାମଗ୍ରୀ ସଂଗ୍ରହ କରିବା<br>M = Food preparation / cooking/ଖାଦ୍ୟ ପ୍ରସ୍ତୁତ କରିବା/ରୋଷେଇ କରିବା<br>N = Other paid or unpaid domestic work (e.g. cleaning, caring for children or others)/ ଅନ୍ୟାନ୍ୟ ଦେୟ ଓ ଅଣଦେୟ ଘରୋଇ କାର୍ଯ୍ୟ (ସଫା କରିବା, ପିଲାଙ୍କ ଯତ୍ନନେବା ଓ ଅନାନ୍ୟ)<br>O= Defecation (include time taken to travel to field if open defecation)/ମଳତ୍ୟାଗ କରିବାକୁ ଯିବା(ଯିବା ଆସିବା ସମୟକୁ ମିଶାଇ)<br>X = Other/ ଅନ୍ୟାନ୍ୟ (ଦର୍ଶାନ୍ତୁ)

19 Group membership/ଗୋଷ୍ଠୀ ସଦସ୍ୟତା

Now I'm going to ask you about groups in the community. These can either be formal or informal or customary groups/ବର୍ତ୍ତମାନ ମୁଁ ଆପଣଙ୍କୁ ଗ୍ରାମରେ ଥିବା ଗୋଷ୍ଠୀ ଗୁଡିକ ବିଷୟରେ ପଚାରିବି । ଏହା ଆନୁଷ୍ଠାନିକ କିମ୍ବା ଅଣ-ଆନୁଷ୍ଠାନିକ ହୋଇପାରେ ।.

Group	Options	Answer	Question	Answer
Are there any of the following groups/cooperatives in your village or nearby? ଆପଣଙ୍କ ଗ୍ରାମରେ/ଗ୍ରାମ ପାଖରେ କୌଣସି ଗୃପବା ସୋସାଇଟିଅଛିକି ?<br><br>Read out all of the options/ ବିକଳ୍ପ ଗୁଡିକୁ ପଢନ୍ତୁ			If yes, have you been an active member of this group in the last 3 agricultural seasons (June 2015 to May 2016)?<br>ଯଦି ହଁ, ଅପଣ ସେଥିରେ ଗତ ଜୁନ, ୨୦୧୫ ରୁ ମେ, ୨୦୧୬ ମଧ୍ୟରେ ଜଣେ ସଦସ୍ୟ ଅଛନ୍ତିକି ? Please explain that "active member" means one who attends meetings, participates in discussions, and volunteers. ସଦସ୍ୟ ଅର୍ଥାତ, ସଭାରେ ଉପସ୍ଥିତ ରହୁଥିବେ, ନିଷ୍ପତ୍ତି ଗ୍ରହଣରେ ଅଂଶଗ୍ରହଣ କରୁଥିବେ ।	
weai_groups_a<br>19.1. Farmers' club (NABARD), milk producer's cooperatives or large area multi-purpose cooperative society (LAMPS)/କୃଶକ ସଙ୍ଘ (ନାବାର୍ଡ),ଦୁଗ୍ଧ ଉତ୍ପାଦନ ସମବାୟ ସଙ୍ଘ କିମ୍ବା ଲ୍ୟାମ୍ପ	Yes/ହଁ--------- 1<br>No/ନା-------- 0<br>Don't know / ଜଣା ନାହିଁ-----98	☐	19.1.1.<br><br>Yes/ହଁ--------- 1<br>No/ନା-------- 0	☐
weai_groups_b<br>19.2 Water or sanitation group (Pani Panchayat), watershed committee or Gaon Kalyan Samiti (GKS) / Village Health Sanitation Committee/ପାଣି ପଞ୍ଚାୟତ, ଜଳବିଭାଜିକା, ଗାଁ କଲ୍ୟାଣ ସମିତି/ସ୍ୱାସ୍ଥ୍ୟ ପରିମଳ ସମିତି	Yes/ହଁ--------- 1<br>No/ନା-------- 0<br>Don't know / ଜଣା ନାହିଁ-----98	☐	19.2.1.<br><br>Yes/ହଁ--------- 1<br>No/ନା-------- 0	☐
weai_groups_c<br>19.3 Forest users' or protection group (including marketing)/ ଜଙ୍ଗଲ ସଂରକ୍ଷଣ ସମିତି (ଜଙ୍ଗଲଜାତ ଦ୍ରବ୍ୟରର ବିକା କିଣା)	Yes/ହଁ--------- 1<br>No/ନା-------- 0<br>Don't know / ଜଣା ନାହିଁ-----98	☐	19.3.1<br><br>Yes/ହଁ--------- 1<br>No/ନା-------- 0	☐
weai_groups_d<br>19.4 Credit or micro-finance institution/ଋଣ ଦେବା ସଂସ୍ଥା କିମ୍ବା ମାଇକ୍ରୋଫାଇନାନ୍ସ ସଂସ୍ଥା	Yes/ହଁ--------- 1<br>No/ନା-------- 0<br>Don't know / ଜଣା ନାହିଁ-----98	☐	19.4.1<br><br>Yes/ହଁ--------- 1<br>No/ନା-------- 0	☐
weai_groups_e<br>19.5 Village development group/ଗ୍ରାମ ଉନ୍ନୟନ ଗୋଷ୍ଠୀ	Yes/ହଁ--------- 1<br>No/ନା-------- 0<br>Don't know / ଜଣା ନାହିଁ-----98	☐	19.5.1<br><br>Yes/ହଁ--------- 1<br>No/ନା-------- 0	☐
weai_groups_f<br>19.6 Religious groups like Bhajan Mandali or Satsang/ଧାର୍ମିକ ଅନୁଷ୍ଠାନ ଯେପରି ଭଜନ ମଣ୍ଡଳ, ସତସଙ୍ଗ	Yes/ହଁ--------- 1<br>No/ନା-------- 0<br>Don't know / ଜଣା ନାହିଁ-----98	☐	19.6.1<br><br>Yes/ହଁ--------- 1<br>No/ନା-------- 0	☐

Group	Options	Answer	Question	Answer
Are there any of the following groups/cooperatives in your village or nearby? ଆପଣଙ୍କ ଗ୍ରାମରେ/ଗ୍ରାମ ପାଖରେ କୌଣସି ଗୃପବା ସୋସାଇଟିଅଛିକି ?<br><br>Read out all of the options/ ବିକଳ୍ପ ଗୁଡିକୁ ପଢନ୍ତୁ			If yes, have you been an active member of this group in the last 3 agricultural seasons (June 2015 to May 2016)?<br>ଯଦି ହଁ, ଅପଣ ସେଥିରେ ଗତ ଜୁନ, ୨୦୧୫ ରୁ ମେ, ୨୦୧୬ ମଧ୍ୟରେ ଜଣେ ସଦସ୍ୟ ଅଛନ୍ତିକି ? Please explain that "active member" means one who attends meetings, participates in discussions, and volunteers. ସଦସ୍ୟ ଅର୍ଥାତ, ସଭାରେ ଉପସ୍ଥିତ ରହୁଥିବେ, ନିଷ୍ପତ୍ତି ଗ୍ରହଣରେ ଅଂଶଗ୍ରହଣ କରୁଥିବେ ।	
weai_groups_g<br>19.7 SHG (Women's / men's self-help group)/ସ୍ୱୟଂ ସହାୟକ ଗୋଷ୍ଠୀ (ମହିଳା/ପୁରୁଷଙ୍କ ସ୍ୱୟଂ ସହାୟକ ଗୋଷ୍ଠୀ)	Yes/ହଁ--------- 1<br>No/ନା-------- 0<br>Don't know / ଜଣା ନାହିଁ-----98	☐	19.7.1<br><br>Yes/ହଁ--------- 1<br>No/ନା-------- 0	☐
weai_groups_h<br>19.8 School-based groups such as mother & teacher association, or parent & teacher association, or a school committee/ବିଦ୍ୟାଳୟ ଭିତ୍ତିକ ଗୋଷ୍ଠୀ ଯେପରି ମା ଏବଂ ଶିକ୍ଷକ ସଂଘ କିମ୍ବା ପିତାମାତା ଏବଂ ଶିକ୍ଷକ ସଂଘ, ସ୍କୁଲ କମିଟି	Yes/ହଁ--------- 1<br>No/ନା-------- 0<br>Don't know / ଜଣା ନାହିଁ-----98	☐	19.8.1<br><br>Yes/ହଁ--------- 1<br>No/ନା-------- 0	☐
weai_groups_i<br>19.9 Nutrition-related groups like mothers' committee or Jaanch (audit) committee/ ପୋଷଣ ସମନ୍ଧୀୟ ଗୋଷ୍ଠି, ମାତୃ କମିଟି/ଯାଞ୍ଚ କମିଟି	Yes/ହଁ--------- 1<br>No/ନା-------- 0<br>Don't know / ଜଣା ନାହିଁ-----98	☐	19.9.1<br><br>Yes/ହଁ--------- 1<br>No/ନା-------- 0	☐
weai_groups_j<br>19.10 Youth club/ଯୁବକ ସଂଘ	Yes/ହଁ--------- 1<br>No/ନା-------- 0<br>Don't know / ଜଣା ନାହିଁ-----98	☐	19.10.1<br><br>Yes/ହଁ--------- 1<br>No/ନା-------- 0	☐

20 EXPOSURE TO THE INTERVENTIONS/ପ୍ରକଳ୍ପ କାର୍ଯ୍ୟକାରିତା ପ୍ରଦର୍ଶିତ କରିବା

Variable name	Question	Code	Answer
exposure_vidag_any	20.1 In the past 6 months, have you or any other family members ever watched any videos about agriculture disseminated at the SHG or screened in the community?<br>ଗତ ୬ମାସରେ ଆପଣ ବା ଆପଣଙ୍କ ପରିବାରର ଅନ୍ୟକେହି ସଦସ୍ୟ କେବେବି ସ୍ୱୟଂ ସହାୟକ ଗୋଷ୍ଠୀରେ ବା ଗ୍ରାମର ଅନ୍ୟ କୌଣସି ସ୍ଥାନରେ କୃଷି ବିଷୟରେ ଭିଡିଓ ପ୍ରଚାର ଦେଖିଛନ୍ତି କି ?<br>Some videos may have also talked about nutrition (as well as agriculture) in the same video. Include these videos in your answer.<br>ଏପରିକିଛିଭିଡିଓଦେଖିଛନ୍ତିଯେଉଁଥିରେକୃଷି ସହିତପୋଷଣବିଷୟରେ ମଧ୍ୟକୁହା ଯାଇଥିଲା।ଏହିଭିଡିଓ ଗୁଡିକୁ ଅନ୍ତର୍ଭୁକ୍ତକରନ୍ତୁ।	Yes/ହଁ--------------------------------- 1<br>*→Go to 20.2*<br>No/ନା----------------------------------- 0<br>*→Go to 20.4*<br>Don't know-------------------------2<br>*→Go to 20.4*	☐

Variable name	Question	Code	Answer
exposure_vidag_n	20.2 How many different agriculture video disseminations / screenings have you or your family members attended over the past 6 months?<br>ଗତ ୬ମାସ ମଧ୍ୟରେ ଆପଣ ବା ଆପଣଙ୍କ ପରିବାରର ଅନ୍ୟକେହି ସଦସ୍ୟ କେତେଥର ବିଭିନ୍ନ ପ୍ରକାରର କୃଷି ବିଷୟରେ ଭିଡିଓ ପ୍ରଚାର ଦେଖିଛନ୍ତି ?<br>Some videos may have also talked about nutrition (as well as agriculture) in the same video. Include these videos in your answer. ଏପରିକିଛିଭିଡିଓଦେଖିଛନ୍ତିଯେଉଁଥିରେକୃଷି ସହିତପୋଷଣବିଷୟରେ ମଧ୍ୟକୁହାଯାଇଥିଲା।ଏହିଭିଡିଓ ଗୁଡିକୁ ଅନ୍ତର୍ଭୁକ୍ତକରନ୍ତୁ।		
expsoure_vidag_hh	20.3 Which family members attended the agriculture video dissemination / screening?<br>ଆପଣଙ୍କ ପରିବାରର କେଉଁମାନେ ଏହି କୃଷି ବିଷୟରେ ଭିଡିଓ ପ୍ରଚାର ଦେଖିଛନ୍ତି ?	Mother / primary caregiver/ମା/ପ୍ରାଥମିକ ଯତ୍ନକାରୀ---------------------------------- 1<br>Husband/ସ୍ୱାମୀ--------------------------- 2<br>Mother-in-law/ଶାଶୁ--------------------- 3<br>Other female household member/ପରିବାରର ଅନ୍ୟ ମହିଳା ସଦସ୍ୟ---4<br>Other male household member / ପରିବାରର ଅନ୍ୟ ପୁରୁଷ ସଦସ୍ୟ--------------- 5	
exposure_vidag_diffused	20.4 In the past 6 months, has anyone in the community (including your family members) talked to you about agriculture-related videos that they watched?<br>ଗତ ୬ମାସ ମଧ୍ୟରେ ଗ୍ରାମର କୌଣସି ଲୋକ ବା ଆପଣଙ୍କ ପରିବାର ସଦସ୍ୟ,ସେମାନେ ଦେଖିଥିବା କୃଷି ଉପରେ ଭିଡିଓ ପ୍ରଚାର ବିଷୟରେ ଆପଣଙ୍କୁ କହିଥିଲେ କି ?	Yes/ହଁ-------------------------------- 1<br>No/ନା---------------------------------- 0<br>Don't know/ଜଣା ନାହିଁ------------------2	
exposure_vidnut_any	20.5 In the past 6 months, have you or any other family members ever watched any videos about nutrition disseminated at the SHG or screened in the community?<br>ଗତ ୬ମାସ ମଧ୍ୟରେ ଆପଣ ବା ଆପଣଙ୍କ ପରିବାରର ଅନ୍ୟକେହି ସଦସ୍ୟ କେବେବି ସ୍ୱୟଂ ସହାୟକ ଗୋଷ୍ଠୀରେ ବା ଗ୍ରାମର ଅନ୍ୟ କୌଣସି ସ୍ଥାନରେ ପୋଷଣବିଷୟରେ ଭିଡିଓ ପ୍ରଚାର ଦେଖିଛନ୍ତି କି ?<br>Exclude any videos that mentioned agriculture. Here, only include videos that are all about nutrition (and do not have anything on agriculture).କୃଷିସମ୍ବନ୍ଧୀୟଭିଡିଓଗୁଡିକୁବାଦଦିଅନ୍ତୁ,କେବଳପୋଷଣସମ୍ବନ୍ଧୀୟଭିଡିଓ ଗୁଡିକୁ ଅନ୍ତର୍ଭୁକ୍ତକରନ୍ତୁ।(କୌଣସି କୃଷିବିଷୟରେନୁହେଁ)	Yes/ହଁ-------------------------------- 1<br>→ *Go to 20.6*<br>No/ନା---------------------------------- 0<br>→ *Go to 20.8*	
exposure_vidnut_n	20.6 How many different nutrition video disseminations / screenings have you or your family members attended over the past 6 months?<br>ଗତ ୬ମାସ ମଧ୍ୟରେ ଆପଣ ବା ଆପଣଙ୍କ ପରିବାରର ଅନ୍ୟକେହି ସଦସ୍ୟ କେତେଥର ବିଭିନ୍ନ ପ୍ରକାରର ପୋଷଣ ବିଷୟରେ ଭିଡିଓ ପ୍ରଚାର ଦେଖିଛନ୍ତି ? Exclude any videos that mentioned agriculture. Here, only include videos that are all about nutrition (and do not have anything on agriculture). କୃଷି ସମ୍ବନ୍ଧୀୟ ଭିଡିଓ ଗୁଡିକୁ ବାଦ ଦିଅନ୍ତୁ,କେବଳ ପୋଷଣ ସମ୍ବନ୍ଧୀୟ ଭିଡିଓ ଗୁଡିକୁ ଅନ୍ତର୍ଭୁକ୍ତ କରନ୍ତୁ।(କୌଣସି କୃଷିବିଷୟରେନୁହେଁ)		
expsoure_vidnut_hh	20.7 Which family members attended the nutrition video dissemination / screening?<br>ଆପଣଙ୍କ ପରିବାରର କେଉଁମାନେ ଏହି କୃଷିବିଷୟରେ ଭିଡିଓ ପ୍ରଚାର ଦେଖିଛନ୍ତି ?<br><Multiple answer>	Mother / primary caregiver/ମା/ପ୍ରାଥମିକ ଯତ୍ନକାରୀ---------------------------------- 1<br>Husband/ସ୍ୱାମୀ-------------------------- 2<br>Mother-in-law/ଶାଶୁ--------------------- 3<br>Other female household member/ପରିବାରର ଅନ୍ୟ ମହିଳା ସଦସ୍ୟ---4<br>Other male household member / ପରିବାରର ଅନ୍ୟ ପୁରୁଷ ସଦସ୍ୟ---------------5	

Variable name	Question	Code	Answer
exposure_vidnut_diffused	20.8 In the past 6 months, has anyone in the community (including your family members) talked to you about nutrition-related videos that they watched?<br>ଗତ ୬ମାସ ମଧ୍ୟରେ ଗ୍ରାମର କୌଣସି ଲୋକ ବା ଆପଣଙ୍କ ପରିବାର ସଦସ୍ୟ,ସେମାନେ ଦେଖିଥିବା ପୋଷଣଉପରେ ଭିଡିଓ ପ୍ରଚାର ବିଷୟରେ ଆପଣଙ୍କୁ କହିଥିଲେ କି ?	Yes/ହଁ--------------------------------- 1<br>No/ନା---------------------------------- 0	☐
exposure_pla_any	20.9 In the past 6 months have you ever attended any participatorywomen's groups meetings by VARRAT where videos were not shown?<br>ଗତ ୬ମାସ ମଧ୍ୟରେ ଆପଣ କେବେବି ଭାରତ ଦ୍ୱାରା ହୋଇଥିବା ମହିଳା ମାନଙ୍କ ସହଭାଗିତା ସଭାରେ ଅଂଶଗ୍ରହଣ କରିଛନ୍ତି କି ? ଯେଉଁଠାରେ କୌଣସି ଭିଡିଓ ଦେଖା ଯାଇନାହିଁ)	Yes/ହଁ--------------------------------- 1<br>→ *go to 20.10*<br>No/ନା---------------------------------- 0<br>→ *Go to 20.12*	☐
exposure_pla_n	20.10 How many participatory women's groups meetings by VARRAT (where videos were not shown) have you attended in the past 6 months?<br>ଗତ ୬ମାସ ମଧ୍ୟରେ ଆପଣ କେତେଥର ଭାରତ ଦ୍ୱାରା ହୋଇଥିବା ମହିଳା ମାନଙ୍କ ସହଭାଗୀତା ସଭାରେ ଭାଗ ନେଇଥିଲେ ? ଯେଉଁଠାରେ କୌଣସି ଭିଡିଓ ଦେଖା ଯାଇନାହିଁ)		☐
expsoure_pla_hh	20.11 Which family members attended these participatorywomen's groups meetings by VARRAT (that did not show any videos)?<br>ପରିବାରର କେଉଁ ସଦସ୍ୟ ଭାରତ ଦ୍ୱାରା ଆୟୋଜିତ ମହିଳା ମାନଙ୍କସହଭାଗିତା ସଭାରେ ଭାଗ ନେଇଥିଲେ ?(ଯେଉଁଠାରେ କୌଣସି ଭିଡିଓ ଦେଖା ଯାଇନାହିଁ)	Mother / primary caregiver/ମା/ପ୍ରାଥମିକ ଯତ୍ନକାରୀ-----------------------------1<br>Husband/ସ୍ୱାମୀ---------------------2<br>Mother-in-law/ଶାଶୁ---------------3<br>Other female household member/ପରିବାରର ଅନ୍ୟ ମହିଳା ସଦସ୍ୟ---4<br>Other male household member / ପରିବାରର ଅନ୍ୟ ପୁରୁଷ ସଦସ୍ୟ--------- 5	☐<br>☐<br>☐<br>☐<br>☐
exposure_homevisits	20.12 Have you/ your home / your family membersever been visited by VARRAT community support persons at your home to discuss nutrition and/or agriculture?<br>ଭାରତର ଗ୍ରାମ ସହଯୋଗୀ ପୋଷଣ କିମ୍ବା କୃଷି ବିଷୟରେ ଆଲୋଚନା ପାଇଁ କେବେବି ଆପଣଙ୍କ ଘରକୁ ଆସିଥିଲେ କି ?	Yes/ହଁ----------------------------------- 1<br>No/ନା----------------------------------- 0<br>→ *Go to end of interview*	☐
exposure_homevisits_n	How many times did a VARRAT community support person visit you / your family members / your home? ଭାରତର ଗ୍ରାମ ସହଯୋଗୀଆପଣଙ୍କ ଘରକୁ କେତେ ଥର ଆସିଛନ୍ତି?		☐

21. Shocks

In the past 90 days, did the following events affect your or your child's intake? Overall, did you or your child eat more than usual, less than usual, or about the same/ଗତ ୯୦ ଦିନ ମଧ୍ୟରେ ନିମ୍ନଲିଖିତ ଘଟନା ପାଇଁ ଆପଣଙ୍କ ଶିଶୁର ଖାଦ୍ୟ ପ୍ରଭାବିତ ହୋଇଥିଲା କି? ଏହି ଘଟଣା ଯୋଗୁଁ ମୋଟାମୋଟି ଆପଣଙ୍କ ଶିଶୁ ସାଧାରଣରୁ ଅଧିକ, ସାଧାରଣରୁ କମ କିମ୍ବା ପାଖାପାଖି ସମାନ ଖାଇଥିଲା ?			
Shock_diet_death_diet	21.1 Death or serious illness of a household member/ପରିବାରର କୌଣସି ସଦସ୍ୟଙ୍କ ମୃତ୍ୟୁ କିମ୍ବା ଗୁରୁତର ଅସୁସ୍ଥତା	Less than usual food intake/ସାଧାରଣ ଖାଦ୍ୟରୁ କମ------------------------------1<br>More than usual food intake/ ସାଧାରଣ ଖାଦ୍ୟରୁ ଅଧିକ-----------------2<br>The same food intake/ସମପରିମାଣର ଖାଦ୍ୟ----------------------------------3<br>No death or illness/କୌଣସି ମୃତ୍ୟୁ କିମ୍ବା ଅସୁସ୍ଥତା ନାହିଁ------------------------0	☐

Shock_diet_livelihood	21.2 Loss of livelihood (e.g. job loss, crop failure, harvest loss)/ବୃତ୍ତିର କ୍ଷତି (ଯଥା: କାମ ହରାଇବା, ଚାଷରେ ଅସଫଳ, ଅମଳରେ କ୍ଷତି)	Less than usual food intake/ସାଧାରଣ ଖାଦ୍ୟରୁ କମ-------------------------1<br>More than usual food intake/ ସାଧାରଣ ଖାଦ୍ୟରୁ ଅଧିକ------------------2<br>The same food intake/ସମପରିମାଣର ଖାଦ୍ୟ-----------------------------------3<br>No loss of livelihood/ବୃତ୍ତିର କୌଣସି କ୍ଷତି ନାହିଁ--------------------------------0	☐
shock_diet_celebrations	21.3 Celebrations, including weddings and festivals/ଉତ୍ସବ ପାଳନ କରିବା, ପର୍ବପର୍ବାଣୀ ଏବଂ ବିଭାଘର ଅନ୍ତର୍ଭୁକ୍ତ	Less than usual food intake/ସାଧାରଣ ଖାଦ୍ୟରୁ କମ-------------------------1<br>More than usual food intake/ ସାଧାରଣ ଖାଦ୍ୟରୁ ଅଧିକ------------------2<br>The same food intake/ସମପରିମାଣର ଖାଦ୍ୟ-----------------------------------3<br>No celebrations/କୌଣସି ଉତ୍ସବ ନାହିଁ---0	☐
shock_diet_govt	21.4New government policy affecting the use of 500 and 1000 INR notes/ ୫୦୦ ଓ ୧୦୦୦ ଟଙ୍କିଆ ନୋଟ ବ୍ୟବହାର ଉପରେ ସରକାରଙ୍କ ନୂଆ ନିୟମ ପ୍ରଭାବିତ କରୁଛି	Less than usual food intake/ସାଧାରଣ ଖାଦ୍ୟରୁ କମ-------------------------1<br>More than usual food intake/ ସାଧାରଣ ଖାଦ୍ୟରୁ ଅଧିକ------------------2<br>The same food intake/ସମପରିମାଣର ଖାଦ୍ୟ-----------------------------------3<br>→ If the same food intake, ask 21.4.1	☐
shock_diet_govknow	21.4.1 Did you know/ have you heard about this policy/ଆପଣ ଏହି ନିୟମ ବିଷୟରେ ଜାଣିଛନ୍ତି/ଶୁଣିଛନ୍ତି କି?	Yes /ହଁ--1<br>No/ନା --0	☐
shock_diet_calamity	21.5. Natural calamity, and calamity related damage to crop, livestock , food stored, homestead and productive assets/ପ୍ରାକୃତିକ ବିପର୍ଯ୍ୟୟ, ଏବଂ ଫସଲ, ପଶୁ ସମ୍ପଦ, ଖାଦ୍ୟ ସଂରକ୍ଷଣ, ଘରବାରି ଏବଂ ଉତ୍ପାଦନକ୍ଷମ ସାମଗ୍ରୀର ବିପର୍ଯ୍ୟୟ ସମ୍ବନ୍ଧୀୟ କ୍ଷତି	Less than usual food intake/ସାଧାରଣ ଖାଦ୍ୟରୁ କମ-------------------------1<br>More than usual food intake/ ସାଧାରଣ ଖାଦ୍ୟରୁ ଅଧିକ------------------2<br>The same food intake/ସମପରିମାଣର ଖାଦ୍ୟ-----------------------------------3<br>No natural calamity/କୌଣସି ପ୍ରାକୃତିକ ବିପର୍ଯ୍ୟୟ ନାହିଁ-------------------------0	☐
