# Supplementary material for: Upscaling Participatory Action and Videos for Agriculture and Nutrition (UPAVAN) trial comparing three variants of a nutrition-sensitive agricultural extension intervention to improve maternal and child nutritional outcomes in rural Odisha, India: study protocol for a cluster randomised controlled trial
Source: Trials. 2018 Mar 9;19:176. doi: 10.1186/s13063-018-2521-y (PMC5845188; doi:10.1186/s13063-018-2521-y)
Supplement: Supplementary file 2 — Baseline questionnaires. (ZIP 6604 kb) [file 13063_2018_2521_MOESM2_ESM.zip › Baseline_Male_Empowerment -FinalR1.pdf]

Upscaling Participation and Videos for Agriculture and Nutrition (UPAVAN)

କୃଷି ଓ ପୋଷଣ ନିମନ୍ତେ ସହଯୋଗୀତାର ମାତ୍ରାକୁ ଅଧିକ କରିବା ଏବଂ ଭିଡିଓ କରିବା (ଉପବନ)

Baseline survey

ବେସଲାଇନ ସର୍ବେକ୍ଷଣ

MALE RESPONDENTS

ପୁରୁଷ ଉତ୍ତରଦାତା

***** Empowerment version *****

- **Year-long reference period** for agricultural income is from June 2015 to May 2016 (includes all three agriculture seasons)
  କୃଷିରୁ ହେଉଥିବା ଆୟ ଆକଳନ ନିମନ୍ତେ ଠିକ ବର୍ଷେ ପଛର ସମୟକୁ ଧରନ୍ତୁ, ଯାହାକି ଜୁନ, ୨୦୧୫ ଠାରୁ ମେ, ୨୦୧୬ ପର୍ଯ୍ୟନ୍ତ (ଯେଉଁଥିରେ ୩ଟି କୃଷିଋତୁ ଆସୁଥିବ)

Respondent identification

- If spouse of the primary caregiver of the index child (typically the father of index child) is available, interview him.
- If the spouse is not available, interview another adult male decision-maker who is involved in major household economic decisions.
- If all adult males who are involved in major household economic decisions are temporarily unavailable, revisit the household (make 3 attempts to interview a male household member involved in major decisions).
- If there are no adult male decision-makers living at home (or you have made 3 attempts to find a male decision maker) interview a woman in the household who is responsible for, or participates in, agriculture-related and income earning decisions.
- ଯଦିପ୍ରାଥମିକ ଯତ୍ନକାରୀଙ୍କ ସ୍ୱାମୀ(ଚୟନ ହୋଇଥିବା ଶିଶୁର ପିତା ଉପସ୍ଥିତ ଥାନ୍ତି ତେବେ ତାଙ୍କୁ ସାକ୍ଷାତକାର କରନ୍ତୁ।
- ଯଦି ସ୍ୱାମୀଉପସ୍ଥିତ ନଥାନ୍ତି ତେବେ ଅନ୍ୟ ଜଣେ ବୟସ୍କ ପୁରୁଷଙ୍କୁ ସାକ୍ଷାତକାର କରନ୍ତୁ ଯିଏ କି ଘରର ମୁଖ୍ୟ ଅର୍ଥନୈତିକ ନିଷ୍ପତି ନେବାରେ ସାମିଲ ହୁଅନ୍ତି
- ଯଦି ଘରର ମୁଖ୍ୟ ଅର୍ଥନୈତିକ ନିଷ୍ପତି ନେବାରେ ସାମିଲ ହେଉଥିବା ସମସ୍ତ ବୟସ୍କ ପୁରୁଷମାନେ କିଛି କାଳ ପାଇଁ ଉପସ୍ଥିତ ନଥାନ୍ତି ତେବେ ସେହି ଘରକୁ ପୁନର୍ବାର ଯାଆନ୍ତୁ (ଘରର ମୁଖ୍ୟ ଅର୍ଥନୈତିକ ନିଷ୍ପତି ନେବାରେ ଭାଗ ନେଉଥିବା ପୁରୁଷ ମାନଙ୍କୁ ସାକ୍ଷାତକାର କରିବାପାଇଁ 3 ଥର ପ୍ରୟାସ କରନ୍ତୁ)
- ଯଦି ସେହି ଘରେ କୌଣସି ପୁରୁଷ ଉତ୍ତରଦାତା ନଥାନ୍ତି (କିମ୍ବା ଅନ୍ୟଜଣେ ପୁରୁଷ ନିଷ୍ପତି ଗ୍ରହଣକାରିଙ୍କୁ ପାଇବା ନିମନ୍ତେ 3 ଥର ପ୍ରୟାସ କରିଛନ୍ତି)ତେବେ ସେହି ଘରର ମହିଳାଙ୍କୁ ପଚାରନ୍ତୁ ଯିଏକି କୃଷି କାର୍ଯ୍ୟ ଓ ଆୟ ସମ୍ବନ୍ଧୀୟ ନିଷ୍ପତି ନେବା ରେ ଦାଇତ୍ୱ ନିଅନ୍ତି ବା ଅଂଶ ଗ୍ରହଣ କରନ୍ତି।

1. Household Identification/ ପରିବାରରପରିଚୟ

Enter Village Code: V   Household Unique Identification Code (HUIC): Bl | C | V | St | Hh

Variable name	Question	Answer
block	**1.1 Block**<br><br>Ghatagaon------------------------ 1<br>Harichandanpur ----------------- 2<br>Patna --------------------------------3<br>Keonjhar (Sadar) ----------------- 4	☐
cluster	1.2 Cluster	☐☐☐
village	1.3 Village	
hamlet	1.4 Hamlet	
st_number	1.5 Structure Number	☐☐☐
hh_number	1.6 HH Number	☐☐☐

Variable name	Question	Code	Answer
survey_type	1.7 Which version of this survey did your supervisor allocate to you?<br>ଆପଣଙ୍କୁ ଆପଣଙ୍କ ସୁପରଭାଇଜର ସର୍ଭେ ର କେଉଁ ଭାଗ ବଣ୍ଟନ କରିଛନ୍ତି	Household consumption ଘରୋଇ ଉପଯୋଗ ----------- 1<br>Male empowerment ପୁରୁଷ ସଶକ୍ତି କରଣ ---------------- 0	☐
index_child_name	1.8 Name of index child<br>ଚୟନ କରାଯାଇଥିବା ଶିଶୁର ନାମ		
mother_name	1.9 Name of mother / primary caregiver of index child<br>ଚୟନ କରାଯାଇଥିବା ଶିଶୁର ମା/ପ୍ରାଥମିକ ଯତ୍ନକରୀଙ୍କ ନାମ		
**1.10 GIS Coordinates**			
gis_long	1.10.1 Longitude	☐☐.☐☐☐	
gis_lat	1.10.2 Latitude		

		☐☐.☐☐☐
gis_alt	1.10.3 Altitude	☐☐.☐☐

2. Survey Team Identification / ସର୍ବେକ୍ଷଣ ଦଳର ପରିଚୟ

Variable name	Question	Name
interviewer	2.1 Interviewer / Field Investigator	
supervisor	2.2. Field Supervisor	

3. **Date and Time of Interviewer Visits to the Household**
ଅନୁସନ୍ଧାନ କାରୀଙ୍କ ପରିବାର ପରିଦର୍ଶନର ସମୟ ଓ ତାରିଖ

Variable name	Question	Code	Answer
**3.1 1st Visit to the household**/ ପରିବାରକୁ ପ୍ରଥମ ପରିଦର୍ଶନ			
date_1	3.1.1 Date/ତାରିଖ	d d / m m / y y y y	
time_1	3.1.2 Time/ସମୟ	__ __ : __ __	
respondent_present_1	3.1.3 Is the spouse of the primary caregiver, or another male decision-maker, available at home?<br>ପ୍ରାଥମିକ ଯତ୍ନକାରୀଙ୍କ ସ୍ୱାମୀବା ଅନ୍ୟ ପୁରୁଷ ନିଷ୍ପତି ଗ୍ରହଣକାରି ଘରେ ଉପସ୍ଥିତ ଅଛନ୍ତି କି?	Yes: spouse is availableହଁ: ସ୍ୱାମୀ ଉପସ୍ଥିତ ଅଛନ୍ତି ------------- A<br>→ *Go to Q4.1*<br>Yes: another male decision-maker is available/ ହଁ: ଅନ୍ୟ ପୁରୁଷ ନିଷ୍ପତି ଗ୍ରହଣକାରି ଉପସ୍ଥିତ ଅଛନ୍ତି-------------------------- B<br>→ *Go to Q4.1*<br>No: male decision-makers do live at this household but they are not at home / temporarily unavailable | ନାଁ : ପୁରୁଷ ନିଷ୍ପତି ଗ୍ରହଣକାରି ଘରେ ରହୁଛନ୍ତି କିନ୍ତୁ ବର୍ତମାନ ଉପସ୍ଥିତ ନାହାନ୍ତି --- C<br>→ *Revisit the household & go to Q3.2*<br>No, there are no male decision-makersliving in this household/ ନାଁ: କୌଣସି ପୁରୁଷ ନିଷ୍ପତି ଗ୍ରହଣକାରି ଘରେ ରହୁନାହାନ୍ତି -- D<br>→ *Interview a female decision-maker & go to Q4.1*	☐
**3.2 2nd Visit to the household/**ପରିବାରକୁ ଦ୍ୱିତୀୟ ପରିଦର୍ଶନ			
date_2	3.2.1. Date/ତାରିଖ	d d / m m / y y y y	
time_2	3.2.2. Time/ସମୟ	__ __ : __ __	
respondent_present_2	3.2.3. Is the spouseof the primary caregiver, or another male decision-maker, available at home?<br>ପ୍ରାଥମିକ ଯତ୍ନକାରୀଙ୍କ ସ୍ୱାମୀ ବା ଅନ୍ୟ ପୁରୁଷ ନିଷ୍ପତି ଗ୍ରହଣକାରି ଘରେ ଉପସ୍ଥିତ ଅଛନ୍ତି କି?	Yes: spouse is availableହଁ: ସ୍ୱାମୀ ଉପସ୍ଥିତ ଅଛନ୍ତି ------------- A<br>→ *Go to Q4.1*<br>Yes: another male decision-maker is available/ ହଁ: ଅନ୍ୟ ପୁରୁଷ ନିଷ୍ପତି ଗ୍ରହଣକାରି ଉପସ୍ଥିତ ଅଛନ୍ତି-------------------------- B<br>→ *Go to Q4.1*<br>No: male decision-makers do live at this household but they are not at home / temporarily unavailable | ନାଁ : ପୁରୁଷ ନିଷ୍ପତି ଗ୍ରହଣକାରି ଘରେ ରହୁଛନ୍ତି କିନ୍ତୁ ବର୍ତମାନ ଉପସ୍ଥିତ ନାହାନ୍ତି --- C<br>→ *Revisit the household & go to Q3.2*<br>No, there are no male decision-makersliving in this household/ ନାଁ: କୌଣସି ପୁରୁଷ ନିଷ୍ପତି ଗ୍ରହଣକାରି ଘରେ ରହୁନାହାନ୍ତି -- D<br>→ *Interview a female decision-maker & go to Q4.1*	☐

Variable name	Question	Code	Answer
**3.3 3rd Visit to the household/** ପରିବାରକୁ ତୃତୀୟ ପରିଦର୍ଶନ			
date_3	3.3.1 Date/ତାରିଖ	d d / m m / y y y y	
time_3	3.3.2. Time/ସମୟ	_ _ : _ _	
respondent_present_3	3.3.3. Is the spouse of the primary caregiver, or another male decision maker, available at home?<br>ପ୍ରାଥମିକ ଯତ୍ନକାରୀଙ୍କ ସ୍ୱାମୀ ବା ଅନ୍ୟ ପୁରୁଷ ନିଷ୍ପତି ଗ୍ରହଣକାରି ଘରେ ଉପସ୍ଥିତ ଅଛନ୍ତି କି?	Yes: spouse is availableହଁ: ସ୍ୱାମୀ ଉପସ୍ଥିତ ଅଛନ୍ତି ------------- A<br>→ *Go to Q4.1*<br>Yes: another male decision-maker is available/ ହଁ: ଅନ୍ୟ ପୁରୁଷ ନିଷ୍ପତି ଗ୍ରହଣକାରି ଉପସ୍ଥିତ ଅଛନ୍ତି-------------------------- B<br>→ *Go to Q4.1*<br>No: male decision-makers do live at this household but they are not at home / temporarily unavailable | ନାଁ : ପୁରୁଷ ନିଷ୍ପତି ଗ୍ରହଣକାରି ଘରେ ରହୁଛନ୍ତି କିନ୍ତୁ ବର୍ତମାନ ଉପସ୍ଥିତ ନାହାନ୍ତି --- C<br>→ *Revisit the household & go to Q3.2*<br>No, there are no male decision-makersliving in this household/ ନାଁ: କୌଣସି ପୁରୁଷ ନିଷ୍ପତି ଗ୍ରହଣକାରି ଘରେ ରହୁନାହାନ୍ତି --- D<br>→ *Interview a female decision-maker & go to Q4.1*	☐

4. Informed Consent/ ସହମତି ପତ୍ର

Consent: Introduce yourself and ask the respondent their name (you only need to record it later).
ନିଜର ପରିଚୟ ଦିଅନ୍ତୁ ଏବଂଉତ୍ତରଦାତାଙ୍କୁ ତାଙ୍କ ନାମ ପଚାରନ୍ତୁ(ଏହାକୁ ଆପଣ ପରେ ଲେଖିବେ)

Provide information sheet and explain the purpose, process, risks and benefits of participating in the survey to the respondent. Take care to be very detailed on confidentiality and the voluntary nature of the study. Consent MUST BE obtained for the survey. One paper copy of the information sheet and signed consent form must be provided to and retained by the household member, and another signed copy of the consent form must be retained with the enumerator and submitted toyour supervisor.

ସହମତ୍ତି ପତ୍ର: ସୂଚନା ପତ୍ରଟି ତାଙ୍କୁ ପ୍ରଦାନକରନ୍ତୁ ଓ ତାଙ୍କୁ ଏହି ସର୍ଭେର ଉଦ୍ଦେଶ୍ୟ, ପ୍ରକ୍ରିୟା, ବିପଦ ଓ ଲାଭ ବିଷୟରେ ବର୍ଣ୍ଣନା କରନ୍ତୁ । ସବିଶେଷ ବର୍ଣ୍ଣନା କଲାବେଳେ ଏହି ଆନୁଧ୍ୟାନର ଗୋପନୀୟତା ଓ ସ୍ୱେଚ୍ଛାକୃତତାର ଯତ୍ନନେବା ଉଚିତ । ଏହି ସର୍ବେକ୍ଷଣ ନିମନ୍ତେ ସହମତ୍ତି ନେବା ନିହାତି ଜରୁରୀ ଅଟେ ।ସୂଚନା ପତ୍ର ଓ ସହମତ୍ତି ପତ୍ରର ଅବିକଳ ନକଲ ପ୍ରଦାନ ଓ ସହମତ୍ତି ପତ୍ରରେ ଦସ୍ତଖତ କରି ସେଥିରୁ ଗୋଟିଏ ନକଲ ପରିବାର ସଭ୍ୟଙ୍କ ଜିମାଦେଇ ଅନ୍ୟଟି ଅନୁସନ୍ଧାନକାରୀ ନିଜ ସୁପରଭାଇଜରଙ୍କ ନିକଟରେ ଜମା କରନ୍ତୁ ।

consent_male0	4.1. Did a respondent give consent?<br>ଉତ୍ତରଦାତା ସହମତି ଦେଲେକି ?	Yes, written / thumbprint/ ହଁ, ହସ୍ତାକ୍ଷର / ଅଙ୍ଗୁଠି ଛାପ------ 1<br>Yes, verbal (witnessed)/ ହଁ, ମୁହଁରେ କହିଲେ(ସାକ୍ଷୀଙ୍କ ଉପସ୍ଥିତିରେ,ପଡୋଶୀ ବା ସାଙ୍ଗ ସାଥି)------------------------------ 2<br>No/ ନା--0	☐

5. Background Information of respondent/ ପରିବାରର ମୌଳିକ ତଥ୍ୟ

Variable name	Question	Code	Answer
male _name	5.1. What is the name of the respondent/ ଉତ୍ତରଦାତାଙ୍କ ନାମ କଣ?		
male_identity	5.2. Is the respondent the father of the index child, another malemain decision maker, or a female decision-maker? ଉତ୍ତରଦାତା ଜଣଙ୍କ ଚୟନ କରାଯାଇଥିବା ଶିଶୁର ପିତା ନା ଅନ୍ୟ ପୁରୁଷ ନିଷ୍ପତି ଗ୍ରହଣକାରୀ ବା ମହିଳା ନିଷ୍ପତି ଗ୍ରହଣକାରୀ ଅଟନ୍ତି କି?	Spouse of the primary caregiver / ପ୍ରାଥମିକ ଯତ୍ନକାରୀଙ୍କ ସ୍ୱାମୀ---------------------------------- 1<br>Another male decision maker /ଅନ୍ୟ ଜଣେ ପୁରୁଷ ନିସ୍ୱତି ଗ୍ରହଣକରି-------------------------------------- 2<br>Female decision-maker/ମହିଳା ନିସ୍ୱତି ଗ୍ରହଣକାରୀ --- 3	
male_marital	5.3. Are you married? (Select the marital status from the list based on the response) ଆପଣ ବିବାହିତା କି? (ସଠିକ ଉତ୍ତର ଅନୁସାରେ ବୈବାହିକ ସ୍ଥିତି ଚୟନ କରନ୍ତୁ)	<single answer><br>Single/ଏକାକୀ ରହୁଛନ୍ତି--------------------- 1<br>Married/ବିବାହିତ-------------------------- 2<br>Divorced/ଛାଡପତ୍ର------------------------- 3<br>Separated/ଅଲଗା ରହୁଛନ୍ତି---------------- 4<br>Widowed/ବିଧବା-------------------------- 5	
male_head	5.4. What is your relationship to the head of the household? (ଘରର ମୁଖ୍ୟଙ୍କ ସହ ଉତ୍ତରଦାତାଙ୍କ ସମ୍ପର୍କ କଣ)(ଘରର ମୁଖ୍ୟ ଆପଣଙ୍କର କଣ ହୁଅନ୍ତି)	Household head/ ନିଜେ ମୁଖ୍ୟ----------------- 1<br>Wife/husband/ସ୍ତ୍ରୀ/ସ୍ୱାମୀ---------------------- 2<br>Son/daughter/ପୁଅ/ଝିଅ----------------------- 3<br>Son-in-law/daughter-in-law/ଜୋଇଁ/ବହୁ----4<br>Granddaughter/grandson/ନାତୁଣୀ/ନାତି-----5<br>Other/ଅନ୍ୟାନ୍ୟ-----------------------------------6	
male_age	5.5. What is your age? (in completed years)/ ଆପଣଙ୍କ ବୟସ କେତେ? (ସମ୍ପୂର୍ଣ ହୋଇଥିବାବର୍ଷରେ)		
male_educ	5.6. Years of formal education completed and passed by respondent/ଆପଣ କେତେ ପାଠ ପଢିଛନ୍ତି<br><br>(Complete education) if respondent has passed 10th year then write 10; and if failed 10th year then write 9. (ସମ୍ପୂର୍ଣ ଶିକ୍ଷା) ଯଦି ଉତ୍ତରଦାତା ଦଶମ ଶ୍ରେଣୀ ପାସ କରିଛନ୍ତି ତେବେ 10 କୋଡ କରନ୍ତୁ ଏବଂ ଯଦି ଦଶମ ଶ୍ରେଣୀରେ ଫେଲ ହୋଇଥାନ୍ତି ତେବେ ୨ କୋଡ କରନ୍ତୁ।		
hh_gender	5.7. Ask the respondent: are there men and women living in the household, or only women? ଉତ୍ତରଦାତାଙ୍କୁ ପଚାରନ୍ତୁ ଏହି ଘରେ ପୁରୁଷ ଓ ମହିଳା ଉଭୟ ରହୁଛନ୍ତି ନା କେବଳ ମହିଳା ରହୁଛନ୍ତି।	Contains male and female adults/ବୟସ୍କ ପୁରୁଷ ଓ ମହିଳା ଅଛନ୍ତି ------------------ 0<br>Contains female adults only/କେବଳ ବୟସ୍କ ମହିଳା ଅଛନ୍ତି ------------------------ 1	

Variable name	Question	Code	Answer
caste	5.8. Do you belong to any of the following?<br>ନିମ୍ନଲିଖିତ ମଧ୍ୟରୁ ଆପଣ କେଉଁ ବର୍ଗର ଅଟନ୍ତି ?	Scheduled caste/ଅନୁସୂଚିତ ଜାତି ------------------ 1<br>→Go to Q 5.10<br>Scheduled tribe/ଅନୁସୂଚିତ ଜନଜାତି---------------- 2<br>→Go to Q 5.9<br>Other Backward Caste (OBC)/ଅନ୍ୟାନ୍ୟ ପଛୁଆ ବର୍ଗ --- 3<br>→Go to Q 5.10<br>None of the above/କୌଣସିଟି ନୁହେଁ-------------- 4<br>→Go to Q 5.10	☐
tribe	5.9. Which of the following tribes do you belong to?<br>ନିମ୍ନଲିଖିତ ମଧ୍ୟରୁ ଆପଣ କେଉଁ ଜନଜାତିର ଅଟନ୍ତି ?	<Single answer><br><br>Ho/ହୋ -----------------------1<br>Santha/ସାନ୍ତାଳ---------------2<br>Bhuiyan/ଭୂୟାଁ----------------3<br>Bhumij/ଭୂମିଜ----------------4<br>Oraon/ଓରାନ-----------------5<br>Gond/ଗଣ୍ଡ--------------------6<br>Juang/ଜୁଆଙ୍ଗ-----------------7<br>Munda/ମୁଣ୍ଡା-----------------8<br>Other/ଅନ୍ୟାନ୍ୟ ଦର୍ଶାନ୍ତୁ------- 9	☐
fuel	5.10. What type of material does your family use most of the time for cooking?<br>ରୋଷେଇ ସମୟରେ ଆପଣଙ୍କ ପରିବାରରେ ମୁଖ୍ୟତଃ କି ପ୍ରକାର ଜାଳେଣୀ ବ୍ୟବହାର କରନ୍ତି ?	<single answer><br><br>Electricity/ବିଦ୍ୟୁତ-----------------------------1<br>Liquid Petroleum Gas (LPG)/ଗ୍ୟାସ--------2<br>Biogas/ଗୋବରଗ୍ୟାସ--------------------------3<br>Kerosene/କିରୋସିନୀ-------------------------4<br>Coal/lignite / GULs/କୋଇଲା----------------5<br>Charcoal/ଅଙ୍ଗାର------------------------------ 6<br>Wood/କାଠ------------------------------------ 7<br>Straw/shrubs/grassନଡା/ଝାଟି/ଘାସ---------8<br>Dry leaves/ଶୁଖିଲା ପତ୍ର-----------------------9<br>Agricultural crop waste/ଚାଷ ହୋଇଥିବା ଶସ୍ୟର ଅବଶିଷ୍--- 10<br>Cow dung cakes/ଘଷି----------------------11	☐
floor	5.11. Main material of the floor. Record observation.<br>ଗୃହ ଚଟାଣରେ ବ୍ୟବହୃତ ହୋଇଥିବା ମୁଖ୍ୟ ଉପକରଣ<br>(ପ୍ରତ୍ୟକ୍ଷ ଭାବରେ ଦେଖି ଲେଖନ୍ତୁ)	<single answer><br><br>**Natural floor** (mud, clay, earth; sand; dung)/**ପ୍ରାକୃତିକ ଚଟାଣ** (ମାଟି,କାଦୁଅ,ବାଲି,ଗୋବର--- 0<br>**Rudimentary floor** (raw wood planks; palm,bamboo; brick; stone)/**ମୌଳିକ ଚଟାଣ** (କଞ୍ଚାକାଠର ଫାଳ,ତାଳ,ବାଉଁଶ,ଇଟା,ପଥର)------------ 1<br>**Finished floor** (ceramic, cement, polished wood, carpet, polished stone)/**ଭଲ ଚଟାଣ** (ସେରାମିକ,ସିମେଣ୍ଟ,ପଲିସ ହୋଇଥିବା କାଠ,ଗାଲିଚା,ପଲିସ ହୋଇଥିବା ପଥର-------------------------------------- 2	☐

Variable name	Question	Code	Answer
roof	5.12. Main material of the roof.<br>Record observation.<br>ଗୃହ ଛାତରେ ବ୍ୟବହୃତ ହୋଇଥିବା ମୁଖ୍ୟ ଉପକରଣ (ପ୍ରତ୍ୟକ୍ଷ ଭାବରେ ଦେଖି ଲେଖନ୍ତୁ)	<single answer><br>**Natural roofing** (no roof, thatch, palm, reed, grass, mud, plastic sheeting)/ **ପ୍ରାକୃତିକ ଛାତ(**ଛାତ ନାହିଁ,ନଡା ଛପର,ତାଳ,ଘାସ,କାଦୁଅ,ଜରି ଚାଦର)----------0<br>**Rudimentary roofing** (Khappar, rustic mat, palm, bamboo, raw wood/ timber, unburnt brick, loosely packed stone) / **ମୌଳିକ ଛାତ**(ଖପର,ଦେଶୀ ମସିଣା,ତାଳ,ବାଉଁଶ,କାଠଗଣ୍ଡି,କଞ୍ଚାଇଟା,ଢିଲା ପଥର ଥାକ)-- 1<br>**Finished roofing** (metal / asbestos sheets, cement/ concrete, tiles, burnt brick)/ **ଭଲ ଛାତ(**ଧାତୁ/ଆଜବେଷ୍ଟସ ଚଦର,ସିମେଣ୍ଟ/କଂକ୍ରିଟ,ଟାଇଲ,ଇଟା)-- 2	☐
wall	5.13. Main material of the exterior walls.<br>Record observation.<br>ଗୃହ କାନ୍ଥରେ ବ୍ୟବହୃତ ହୋଇଥିବା ମୁଖ୍ୟ ଉପକରଣ (ପ୍ରତ୍ୟକ୍ଷ ଭାବରେ ଦେଖି ଲେଖନ୍ତୁ)	<single answer><br>**Natural walls** (mud, no walls, cane / trunks / bamboo / palm, grass / reeds, thatch)/ **ପ୍ରାକୃତିକ କାନ୍ଥ (**କାଦୁଅ,କାନ୍ଥ ନାହିଁ,ଝାଟି/ଗଛ ଗଣ୍ଡି/ବାଉଁଶ/ତାଳ,ଘାସ/ନଡା)---------------------- 0<br>**Rudimentary walls** (unburnt brick, bamboo with mud, stone with mud, plywood, cardboard, raw or reused wood)/ **ମୌଳିକ କାନ୍ଥ (**କଞ୍ଚାଇଟା,ବାଉଁଶ ଓ ମାଟି,ପଥର ଓ ମାଟି,ପ୍ଲାଏ,ଶକ୍ତ କାଗଜ ପଟା,କଞ୍ଚା ଓ ଭଙ୍ଗା କାଠ)---------------------------- 1<br>**Finished walls** (cement / concrete, stone with lime / cement, burnt bricks, wood planks)/**ଭଲ କାନ୍ଥ** (ସିମେଣ୍ଟ/କଂକ୍ରିଟ,ପଥର ଓ ଚୂନ/ସିମେଣ୍ଟ,ପୋଡାଇଟା,କାଠଫାଳ)--------------- 2	☐

6. **Abbreviated Women's Empowerment in Agriculture Index /ସଂକ୍ଷେପରେକୃଷି କାର୍ଯ୍ୟରେ ମହିଳା ମାନଙ୍କ ସଶକ୍ତିକରଣ For this part of the interview, try to interview the individual in private or where other members of the household cannot overhear or contribute answers. Do not attempt to make responses between the primary male decision-maker and the primary female decision-maker the same; it is ok for them to be different.** ଏହି ଭାଗର ସାକ୍ଷାତକାର ନିମନ୍ତେ, ସାକ୍ଷାତକାରଟିକୁ ଯଥାସମ୍ଭବ ଏକାନ୍ତରେ କରିବାକୁ ଚେଷ୍ଟା କରନ୍ତୁ ଯେଉଁଠାରେ ପରିବାରର ଅନ୍ୟ ସଦସ୍ୟମାନେ ଶୁଣି ପାରିବେନି ବା ଉପରେ ପଡି ଉତ୍ତର ନଦିଅନ୍ତି । ପରିବାରର ମୁଖ୍ୟ ମହିଳା ସଦସ୍ୟ ଓ ମୁଖ୍ୟ ପୁରୁଷ ସଦସ୍ୟଙ୍କ ଉତ୍ତର ସମାନ କରିବାକୁ ଚେଷ୍ଟା କରନ୍ତୁ ନାହିଁ ସେମାନଙ୍କ ଉତ୍ତର ଭିନ୍ନ ବି ହୋଇପାରେ ।

Question	Options	Answer
weai_int_alone<br><br>6.1 Ability to be interviewed alone:<single answer><br>ଏକାକୀ ସାକ୍ଷାତକାରରେ ଭାଗ ନେବା ପାଇଁ ଦକ୍ଷ (ଗୋଟିଏ ଉତ୍ତର ଲେଖନ୍ତୁ)	1. Alone/ଏକୁଟିଆ<br>2. With adult females present/ ଅନ୍ୟାନ ଉପସ୍ଥିତ ବୟସ୍କ ମହିଳାଙ୍କ ସହିତ<br>3. With adult males present/ଅନ୍ୟାନ୍ୟ ଉପସ୍ଥିତ ବୟସ୍କ ପୁରୁଷଙ୍କ ସହିତ<br>4. With adults mixed sex present/ଚିକିତ୍ସା<br>5. With children present/ଉପସ୍ଥିତ ପିଲାମାନଙ୍କ ସହିତ<br>6. With adults mixed sex and children present/ଉପସ୍ଥିତ ମହିଳା, ପୁରୁଷ ଓ ପିଲାମାନଙ୍କ ସହିତ	☐

weai_asset_house<br>6.2 Do you, your household, or anyone in your household currently own a house or other structures?<br>ବର୍ତମାନ ଆପଣଙ୍କର, କିମ୍ବା ଆପଣଙ୍କ ପରିବାରର ଅନ୍ୟ କୌଣସି ସଦସ୍ୟଙ୍କର ଘର, ଗୁହାଳ,ଖଳା ଘର ଅଛି କି?	Yes/ହଁ----1<br>No/ନା----0<br>→*If no, skip to next asset*	☐	weai_asset_house_own<br>6.2.1 Do you own any of the item, either solely or jointly with any other person?<br>ଏହି ଘର ବା ଗୁହାଳ,ଖଳା ଘର ଆପଣଙ୍କ ନାମରେ ଅଛି କି?(ଏକାକୀ ବା ମିଳିତ ଭାବରେ)	Yes, solely/ହଁ, ଏକାକୀ---------1<br>Yes, jointly/ ହଁ,ମିଳିତ ଭାବେ---2<br>No/ନା------------------------0	☐
weai_asset_livestockl<br>6.3 Do you, your household, or anyone in your household currently own large livestock (e.g. oxen, cattle, buffalo)?<br>ଏବେ ଆପଣଙ୍କର କିମ୍ବା ଆପଣଙ୍କ ପରିବାରର ବଡ ଆକାରର ଗୃହପାଳିତ ପଶୁଅଛନ୍ତି କି ଯେପରୀ ବଳଦ,ଗାଈ,ମଇଁଷୀ?	Yes/ହଁ----1<br>No/ନା----0<br>→*If no, skip to next asset*	☐	weai_asset_livestockl_own<br>6.3.1 Do you own any of the item, either solely or jointly with any other person?<br>ଆପଣଙ୍କର ନିଜେ ଏକୁଟିଆ ହେଉ କିମ୍ବା ମିଳିତ ଭାବରେ ହଉ ଏହି ପଶୁ ଗୁଡିକର ମାଲିକ ଅଟନ୍ତି କି?	Yes, solely/ହଁ, ଏକାକୀ---------1<br>Yes, jointly/ ହଁ,ମିଳିତ ଭାବେ---2<br>No/ନା------------------------0	☐

weai_asset_livestocks<br>6.4 Do you, your household, or anyone in your household currently own small livestock (goats, pigs, sheep, chickens, ducks, pigeons)?<br>ଏବେ ଆପଣଙ୍କର କିମ୍ବା ଆପଣଙ୍କ ପରିବାରର ଛୋଟ ଆକାରରପଶୁପକ୍ଷୀ ଅଛନ୍ତି କି ଯେପରି ଛେଳି,ଘୁଷୁରି,ମେଣ୍ଢା,କୁକୁଡା,ବତକ,ପାରା?	Yes/ହଁ----1<br>No/ନା----0<br>→*If no, skip to next asset*	☐	weai_asset_livestocks_own<br>6.4.1 Do you own any of the item, either solely or jointly with any other person?<br>ଆପଣ ନିଜେ ଏକୁଟିଆ ହେଉ କିମ୍ବା ମିଳିତ ଭାବରେ ହଉ ଏହି ପଶୁପକ୍ଷୀ ଗୁଡିକର ମାଲିକ ଅଟନ୍ତି କି?	Yes, solely/ହଁ, ଏକାକୀ---------1<br>Yes, jointly/ ହଁ,ମିଳିତ ଭାବେ---2<br>No/ନା------------------------0	☐
weai_asset_equip_mechanised<br>6.5 Do you, your household, or anyone in your household currently own mechanised farm equipment? (e.g. tractor, power tiller, treadle pump)?<br>ବର୍ତମାନ ଆପଣକିମ୍ବା ଆପଣଙ୍କ ପରିବାରର ଚାଷ ନିମନ୍ତେ ମେଶିନ(ଯନ୍ତ୍ର ଚାଳିତ) ଅଛି କି ଯଥା; ଟ୍ରାକ୍ଟର, ପାୱାର ଟିଲର, ଟ୍ରିଡଲ ପମ୍ପ?	Yes/ହଁ----1<br>No/ନା----0<br>→*If no, skip to next asset*	☐	weai_asset_equip_mechanised_own<br>6.5.1 Do you own any of the item, either solely or jointly with any other person?<br>ଆପଣ ନିଜେ ଏକୁଟିଆ ହଉ କିମ୍ବା ମିଳିତ ଭାବରେ ହଉ ଏହି ମେଶିନ ଗୁଡିକର ମାଲିକ ଅଟନ୍ତି କି ?	Yes, solely/ହଁ, ଏକାକୀ---------1<br>Yes, jointly/ ହଁ,ମିଳିତ ଭାବେ---2<br>No/ନା------------------------0	☐
weai_asset_equip_nonmechanised<br>6.6 Do you, your household, or anyone in your household currently own farm non-mechanised equipment? (e.g. hand tools or animal-drawn plough or cart)<br>ବର୍ତମାନ ଆପଣକିମ୍ବା ଆପଣଙ୍କ ପରିବାରର ଚାଷନିମନ୍ତେଅଣ-ଯାନ୍ତ୍ରିକ ଉପକରଣ ଅଛି କି ?(ଯେପରି ହଳ, ଲଙ୍ଗଳ କିମ୍ବା ଶଗଡ)	Yes/ହଁ----1<br>No/ନା----0<br>→*If no, skip to next asset*	☐	weai_asset_farm nonmecha equip_own<br>6.6.1 Do you own any of the item, either solely or jointly with any other person?<br>ଆପଣ ନିଜେ ଏକୁଟିଆ ହଉ କିମ୍ବା ମିଳିତ ଭାବରେ ହଉ ଏହି ଜିନିଷ ଗୁଡିକର ମାଲିକ ଅଟନ୍ତି କି?	Yes, solely/ହଁ, ଏକାକୀ---------1<br>Yes, jointly/ ହଁ,ମିଳିତ ଭାବେ---2<br>No/ନା------------------------0	☐
weai_asset_equip_ business<br>6.7 Do you, your household, or anyone in your household currently own any nonfarm business equipment (solar panels used for recharging, sewing machine, brewing equipment, fryers)?<br>ବର୍ତମାନ ଆପଣକିମ୍ବା ଆପଣଙ୍କ ପରିବାରର ଅନ୍ୟ କୌଣସି ସଦସ୍ୟଙ୍କର ଚାଷ ଛଡା ଅନ୍ୟାନ୍ୟ ବୈବସାୟିକ ଉପକରଣର ଅଛି କି ? (ଯଥା; ସୋଲାର ପ୍ୟାନେଲ,ସିଲେଇ ମେସିନ, ମଦ୍ୟ ପ୍ରସ୍ତୁତ ଉପକରଣ ଓ ଭଜା ଭଜି କରିବା ଉପକରଣ)	Yes/ହଁ----1<br>No/ନା----0<br>→*If no, skip to next asset*	☐	weai_asset__equip_ business_own<br>6.7.1 Do you own any of the item, either solely or jointly with any other person?<br>ଆପଣ ନିଜେ ଏକୁଟିଆ ହଉ କିମ୍ବା ମିଳିତ ଭାବରେ ହଉ ଏହି ବୈବସାୟିକ ଉପକରଣର ମାଲିକ ଅଟନ୍ତି କି?	Yes, solely/ହଁ, ଏକାକୀ---------1<br>Yes, jointly/ ହଁ,ମିଳିତ ଭାବେ---2<br>No/ନା------------------------0	☐

weai_asset_highcost_durables<br>6.8 Do you, your household, or anyone in your household currently own any high cost consumer durables e.g. refrigerator, TV, sofa, expensive bed etc.?<br>ବର୍ତମାନ ଆପଣକିମ୍ବା ଆପଣଙ୍କ ପରିବାରର ଅନ୍ୟ କୌଣସି ସଦସ୍ୟଙ୍କର ଦାମୀ ଜିନିଷ ଅଛି କି?<br>(ଯଥା;ଫ୍ରିଜ,ଟିଭି,ସୋଫା,ଦାମୀ ବିଛଣା ଇତ୍ୟାଦି)	Yes/ହଁ----1<br>No/ନା----0<br>→*If no, skip to next asset*	☐	weai_asset_highcost_durables_own<br>6.8.1 Do you own any of the item, either solely or jointly with any other person?<br>ଆପଣ ନିଜେ ଏକୁଟିଆ ହଉ କିମ୍ବା ମିଳିତ ଭାବରେ ହଉ ଏହି ଦାମୀ ଜିନିଷର ମାଲିକ ଅଟନ୍ତି କି?	Yes, solely/ହଁ, ଏକାକୀ---------1<br>Yes, jointly/ ହଁ,ମିଳିତ ଭାବେ---2<br>No/ନା------------------------0	☐
weai_asset_lowcost_durables<br>6.9 Do you, your household, or anyone in your household currently own any low cost consumer durables e.g. mattress (gadda), cot (charpai), radio, fan, watch or clock, or cookware?<br>ବର୍ତମାନ ଆପଣଆପଣଙ୍କ ଘରେ ଅଳ୍ପ ଦାମରଜିନିଷ ରଖିଛନ୍ତି କି ? (ଯଥା;ଗଦି,ଖଟ,ରେଡିଓ,ପଙ୍ଖା,କାନ୍ଥ ଘଣ୍ଟା, ହାତ ଘଣ୍ଟା କିମ୍ବା ରୋଷେଇ ଉପକରଣ ଇତ୍ୟାଦି)	Yes/ହଁ----1<br>No/ନା----0<br>→*If no, skip to next asset*	☐	weai_asset_lowcost_durables_own<br>6.9.1 Do you own any of the item, either solely or jointly with any other person?<br>ଆପଣ ନିଜେ ଏକୁଟିଆ ହଉ କିମ୍ବା ମିଳିତ ଭାବରେ ହଉ ଏହି ଜିନିଷ ଗୁଡିକର ମାଲିକ ଅଟନ୍ତି କି?	Yes, solely/ହଁ, ଏକାକୀ---------1<br>Yes, jointly/ ହଁ,ମିଳିତ ଭାବେ---2<br>No/ନା------------------------0	☐
weai_asset_ jewellery<br>6.10Do you, your household, or anyone in your household currently own any metal jewellery (gold, silver, brass or white metal)?<br>ବର୍ତମାନ ଆପଣକିମ୍ବା ଆପଣଙ୍କ ପରିବାରର କୌଣସି ସଦସ୍ୟଙ୍କର ଗହଣା ଅଛି କି ? (ଯଥା;ସୁନା,ରୁପା,ପିତ୍ତଳ,ଧଳା ଧାତୁ ଇତ୍ୟାଦି)	Yes/ହଁ----1<br>No/ନା----0<br>→*If no, skip to next asset*	☐	weai_asset_ jewellery_own<br>6.10.1 Do you own any of the item, either solely or jointly with any other person?<br>ଆପଣ ନିଜେ ଏକୁଟିଆ ହଉ କିମ୍ବା ମିଳିତ ଭାବରେ ହଉଗହଣାର ମାଲିକ ଅଟନ୍ତି କି?	Yes, solely/ହଁ, ଏକାକୀ---------1<br>Yes, jointly/ ହଁ,ମିଳିତ ଭାବେ---2<br>No/ନା------------------------0	☐
weai_asset_phone<br>6.11Do you, your household, or anyone in your household currently own a mobile phone?<br>ବର୍ତମାନ ଆପଣକିମ୍ବା ଆପଣଙ୍କ ପରିବାରର କୌଣସି ସଦସ୍ୟଙ୍କ ମୋବାଇଲ ଫୋନ ଅଛି କି ?	Yes/ହଁ----1<br>No/ନା----0<br>→*If no, skip to next asset*	☐	weai_asset_phone_own<br>6.11.1 Do you own any of the item, either solely or jointly with any other person?<br>ଆପଣ ନିଜେ ଏକୁଟିଆ ହଉ କିମ୍ବା ମିଳିତ ଭାବରେ ହଉ ମୋବାଇଲ ଫୋନର ମାଲିକ ଅଟନ୍ତି କି?	Yes, solely/ହଁ, ଏକାକୀ---------1<br>Yes, jointly/ ହଁ,ମିଳିତ ଭାବେ---2<br>No/ନା------------------------0	☐

asset_elec	6.12 Electricity/ ବିଦ୍ୟୁତ ସଂଯୋଗ	Yes/ହଁ------------1<br>No/ନା-------------0	☐
asset_comp	6.13 Computer/କମ୍ପୁଟର	Yes/ହଁ------------1<br>No/ନା-------------0	☐
asset_internet	6.14 Internet/ଇଣ୍ଟରନେଟ	Yes/ହଁ------------1<br>No/ନା------------0	☐
asset_cycle	6.15 Bicycle/ସାଇକେଲ	Yes/ହଁ------------1<br>No/ନା-------------0	☐
asset_mbike	6.16 Motorbike/ମୋଟର ସାଇକେଲ	Yes/ହଁ------------1<br>No/ନା------------0	☐

7. **Productive decision making** ଉତ୍ପୋଦନ ଭିତ୍ତିକ ନିଷ୍ପତ୍ତି ଗ୍ରହଣ

**Now I'd like to ask you some questions about your participation in certain types of work activities and on making decisions on various aspects of household life/** ଉତ୍ପାଦନ ଭିତ୍ତିକ ନିଷ୍ପତ୍ତି ଗ୍ରହଣ – ବର୍ତ୍ତମାନ ମୁଁ ଆପଣଙ୍କୁ ଆପଣଙ୍କ ପରିବାରର କାମଦାମରେ ଭାଗୀଦାରୀ ଓଏହାରନିଷ୍ପତ୍ତି ନେବା ବିଷୟରେ ପଚାରିବି ।<br>Did you yourself participate in [activity] in the past 3 agricultural seasons (June 2015 to May 2016)?ଗତ ବର୍ଷ ବର୍ଷାଦିନିଆ ଫସଲ ଠାରୁ ଖରାଟିଆ ଫସଲ ପର୍ଯ୍ୟନ୍ତ(ଜୁନ 2015 ରୁ ମେ 2016 ପର୍ଯ୍ୟନ୍ତ) ହୋଇଥିବା ଚାଷକାମରେ ଆପଣ ଭାଗ ନେଇଥିଲେ କି **?**			How much input or influence did you have in making decisions about [activity], including any decisions about income from [activity]? *Input means gave an opinion, was asked an opinion, or was able to influence an action.* Probe: Did your spouse or others in the household consult / ask with you when making decisions about the activity or what to do with the income from that activity? ପରିବାରର ଆର୍ଥିକ ଆୟ ସମ୍ବନ୍ଧୀୟ ନିଷ୍ପତ୍ତି ନେବାରେ ଆପଣଙ୍କକଥା କେତେ ରହିଥିଲା ଏହାର ଅର୍ଥ ଆପଣଙ୍କୁ ପଚରା ଯାଏକି, ଆପଣଙ୍କ ମତାମତକୁ ବିଚାରକୁ ନିଆଯାଏ କି ? ଦର୍ଶାନ୍ତୁ: ଆପଣଙ୍କ ସ୍ଵାମୀ କିମ୍ବା ପରିବାରର ଅନ୍ୟାନ୍ୟ ସଦସ୍ୟ ପରିବାରର ଆର୍ଥିକ ଆୟ ସମ୍ବନ୍ଧୀୟ ନିଷ୍ପତ୍ତି ନେବାବେଳେ ଆପଣଙ୍କ ସହିତ ପରାମର୍ଶ କରନ୍ତି କି ?	
**Activity**	**Options**	**Answer**	**Options**	**Answer**
7.1 Food production for household consumption: these are crops, livestock or fish etc. that are grown primarily for household food consumption ପରିବାର ନିମନ୍ତେ ଖାଦ୍ୟ ଉତ୍ପାଦନ(ଚାଷ,ପଶୁପାଳନ କିମ୍ବା ମାଛ ଚାଷ)।	Yes/ହଁ---------------------- 1<br>No/ନା----------------------0<br>→ go to next activity ପରବର୍ତୀପର୍ଯ୍ୟାୟକୁଯାଆନ୍ତୁ	☐	No input or input in few decisions/ କୌଣସି ଅଂଶଗ୍ରହଣ ନାହିଁ ବା ଅଳ୍ପକିଛି ନିଷ୍ପତ୍ତିରେ ଅଂଶଗ୍ରହଣ-------- 1<br>Input into some decisions/ କିଛି ନିଷ୍ପତ୍ତିରେ ଅଂଶଗ୍ରହଣ-- 2<br>Input into most or all decisions/ ଅଧିକାଂଶ ବା ସମସ୍ତ ନିଷ୍ପତ୍ତିରେ ଅଂଶଗ୍ରହଣ-------------------------- 3<br>No decision made/ କୌଣସି ନିଷ୍ପତ୍ତି ନିଅନ୍ତି ନାହିଁ -- 98	☐
7.2 Cash crop farming: crops grown primarily for income generation. These can be food or non-food items, and may also be used partly (but not mainly) for home consumption. ଅର୍ଥକାରୀ:ଯେଉଁ ଫସଲ ମୁଖ୍ୟତ ଆୟ ସୃଷ୍ଟି କରେ ସେଗୁଡିକ ଖାଦ୍ୟ ବା ଅନ୍ୟ ଖାଦ୍ୟ ହୋଇପାରେ ।	Yes/ହଁ---------------------- 1<br>No/ନା----------------------0<br>→ go to next activity ପରବର୍ତୀପର୍ଯ୍ୟାୟକୁଯାଆନ୍ତୁ	☐	No input or input in few decisions/ କୌଣସି ଅଂଶଗ୍ରହଣ ନାହିଁ ବା ଅଳ୍ପକିଛି ନିଷ୍ପତ୍ତିରେ ଅଂଶଗ୍ରହଣ-------- 1<br>Input into some decisions/ କିଛି ନିଷ୍ପତ୍ତିରେ ଅଂଶଗ୍ରହଣ-- 2<br>Input into most or all decisions/ ଅଧିକାଂଶ ବା ସମସ୍ତ ନିଷ୍ପତ୍ତିରେ ଅଂଶଗ୍ରହଣ-------------------------- 3<br>No decision made/ କୌଣସି ନିଷ୍ପତ୍ତି ନିଅନ୍ତି ନାହିଁ -- 98	☐
7.3 Livestock raising (large and small livestock) ପଶୁପାଳନ(ବଡ ଏବଂ ସାନ)	Yes/ହଁ---------------------- 1<br>No/ନା----------------------0<br>→ go to next activity ପରବର୍ତୀପର୍ଯ୍ୟାୟକୁଯାଆନ୍ତୁ	☐	No input or input in few decisions/ କୌଣସି ଅଂଶଗ୍ରହଣ ନାହିଁ ବା ଅଳ୍ପକିଛି ନିଷ୍ପତ୍ତିରେ ଅଂଶଗ୍ରହଣ-------- 1<br>Input into some decisions/ କିଛି ନିଷ୍ପତ୍ତିରେ ଅଂଶଗ୍ରହଣ-- 2<br>Input into most or all decisions/ ଅଧିକାଂଶ ବା ସମସ୍ତ ନିଷ୍ପତ୍ତିରେ ଅଂଶଗ୍ରହଣ-------------------------- 3<br>No decision made/ କୌଣସି ନିଷ୍ପତ୍ତି ନିଅନ୍ତି ନାହିଁ -- 98	☐
7.4 Non-farm economic activities: this includes things like running a small business, self-employment, buy-and-sell. ଚାଷ ଛଡା ଅନ୍ୟ କିଛି କାମ : ଯେମିତିକି ଛୋଟ ଦୋକାନ,ବିକା କିଣା,ଅନ୍ୟ କିଛି କାମ କରିବା ।	Yes/ହଁ---------------------- 1<br>No/ନା----------------------0<br>→ go to next activityପରବର୍ତୀପର୍ଯ୍ୟାୟକୁଯାଆନ୍ତୁ	☐	No input or input in few decisions/ କୌଣସି ଅଂଶଗ୍ରହଣ ନାହିଁ ବା ଅଳ୍ପକିଛି ନିଷ୍ପତ୍ତିରେ ଅଂଶଗ୍ରହଣ-------- 1<br>Input into some decisions/ କିଛି ନିଷ୍ପତ୍ତିରେ ଅଂଶଗ୍ରହଣ-- 2<br>Input into most or all decisions/ ଅଧିକାଂଶ ବା ସମସ୍ତ ନିଷ୍ପତ୍ତିରେ ଅଂଶଗ୍ରହଣ-------------------------- 3<br>No decision made/ କୌଣସି ନିଷ୍ପତ୍ତି ନିଅନ୍ତି ନାହିଁ -- 98	☐
7.5 Minor household expenditures e.g. food for daily consumption or other household needs ପରିବାରର ନୀତି ଦିନିଆ ଖର୍ଚ୍ଚଯେପରି ଖାଇବା ଖର୍ଚ୍ଚ ଓ ଅନ୍ୟ ସବୁ ଖର୍ଚ୍ଚ?			No input or input in few decisions/ କୌଣସି ଅଂଶଗ୍ରହଣ ନାହିଁ ବା ଅଳ୍ପକିଛି ନିଷ୍ପତ୍ତିରେ ଅଂଶଗ୍ରହଣ-------- 1<br>Input into some decisions/ କିଛି ନିଷ୍ପତ୍ତିରେ ଅଂଶଗ୍ରହଣ-- 2<br>Input into most or all decisions/ ଅଧିକାଂଶ ବା ସମସ୍ତ ନିଷ୍ପତ୍ତିରେ ଅଂଶଗ୍ରହଣ-------------------------- 3<br>No decision made/ କୌଣସି ନିଷ୍ପତ୍ତି ନିଅନ୍ତି ନାହିଁ -- 98	☐

8 ACCESS TO CREDIT/ଋଣ ପାଇଁ ସୁବିଧା

Question	Options	Answer	Variable name	Question	Answer
Access to credit: “Next I'd like to ask about your household's experience with borrowing money or other items in the past 12 months.”/ଋଣ ପାଇଁ ସୁବିଧା :ଗତ 12 ମାସ ଭିତରେ ଘର ପାଇଁ ଟଙ୍କା ବା ଜିନିଷ ଧାରା ଉଧାର କରିବାର ଅନୁଭୂତି ବିଷୟରେ ମୁ ଆପଣଙ୍କୁ ପଚାରିବାକୁ ଚାହୁଁଛି ।					
weai_credit_formal<br>8.1 Have you taken any loans or borrowed cash/in-kind from a formal institution, such as financial institution, bank or a non-governmental organisation in the past 12 months? ଗତ 12 ମାସରେ ଆପଣକୌଣସି ବ୍ୟାଙ୍କ ରୁ କିମ୍ବା ବେସରକାରୀ ସଂସ୍ଥାରୁ ଋଣ ଆଣିଛନ୍ତି କି?	<single answer/ଗୋଟିଏ ଉତ୍ତର ଲେଖନ୍ତୁ><br>Yes, in my own name/ହଁ, ମୋ ନିଜ ନାମରେ ---1<br>Yes, jointly/ହଁ, ମିଳିତ ଭାବରେ-------------------2<br>No/ନା--0<br>Don't know/ଜାଣିନାହିଁ--------------------------98<br>→If 0 or 98, go to 7.2	☐	weai_credit_formal_decide<br>8.1.1 If yes, who decided how to spend this credit most of the time?<br>ଯଦହଁ,ଅଧିକାଂଶ ସମୟ ସେ ଅର୍ଥକୁ କିପରି ଖର୍ଚ୍ଚ କରାଯିବ ତାର ନିଷ୍ପତି କିଏ ନିଅନ୍ତି ?	<multiple answer/ବହୁ ଉତ୍ତର ସମ୍ଭବ><br>Self/ନିଜେ--------------------------- 1<br>Spouse/ସ୍ୱାମୀ/ସ୍ତ୍ରୀ-------------------- 2<br>Other HH member/ପରିବାରର ଅନ୍ୟ ସଦସ୍ୟ-- 3<br>Other non-HH member/ଅନ୍ୟ ବାହାର ବ୍ୟକ୍ତି---- 4<br>Not applicable/ଉପଯୁକ୍ତ ନୁହେଁ---- 98	☐<br>☐<br>☐<br>☐
weai_credit_group<br>8.2 Have you taken any loans or borrowed cash/in-kind from a group based micro-finance or lending, including informal saving or credit community groups or societies ଗତ 12 ମାସ ମଧ୍ୟରେ ଆପଣ କୌଣସି ମାଇକ୍ରୋଫାଇନାନ୍ସ ବା ଘାରୋଇ ସଞ୍ଚୟକାରୀ ସଙ୍ଘ ଗୋଷ୍ଠୀ ସ୍ତରର ସୋସାଇଟୀ ଠାରୁ ଋଣ କରିଛନ୍ତି କି?	<single answer/ଗୋଟିଏ ଉତ୍ତର ଲେଖନ୍ତୁ><br>Yes, in my own name/ହଁ, ମୋ ନିଜ ନାମରେ ---1<br>Yes, jointly/ହଁ, ମିଳିତ ଭାବରେ-------------------2<br>No/ନା--0<br>Don't know/ଜାଣିନାହିଁ--------------------------98<br>→If 0 or 98, go to 7.3	☐	weai_credit_group_decide<br>8.2.1 If yes, who decided how to spend this credit most of the time?<br>ଯଦି ହଁ,ଅଧିକାଂଶ ସମୟ ସେ ଅର୍ଥକୁ କିପରି ଖର୍ଚ୍ଚ କରାଯିବ ତାର ନିଷ୍ପତି କିଏ ନିଅନ୍ତି ?	<multiple answer/ବହୁ ଉତ୍ତର ସମ୍ଭବ><br>Self/ନିଜେ--------------------------- 1<br>Spouse/ସ୍ୱାମୀ/ସ୍ତ୍ରୀ-------------------- 2<br>Other HH member/ପରିବାରର ଅନ୍ୟ ସଦସ୍ୟ-- 3<br>Other non-HH member/ଅନ୍ୟ ବାହାର ବ୍ୟକ୍ତି---- 4<br>Not applicable/ଉପଯୁକ୍ତ ନୁହେଁ---- 98	☐<br>☐<br>☐<br>☐
weai_credit_informal<br>8.3 Have you taken any loans or borrowed cash/in-kind from an informal lender, including local middleman (mahajan), or friends, neighbours or relatives<br>ଆପଣ କୌଣସି ଘରୋଇ ସୁଧ ବେପାରୀ,ମହାଜନ, ସାଙ୍ଗ ବା ବନ୍ଧୁବାନ୍ଧବଙ୍କ ଠାରୁ ଋଣ କରିଛନ୍ତି କି ?	<single answer/ଗୋଟିଏ ଉତ୍ତର ଲେଖନ୍ତୁ><br>Yes, in my own name/ହଁ, ମୋ ନିଜ ନାମରେ ---1<br>Yes, jointly/ହଁ, ମିଳିତ ଭାବରେ-------------------2<br>No/ନା--0<br>Don't know/ଜାଣିନାହିଁ--------------------------98<br>→If 0 or 98, go to 7.4	☐	weai_credit_informal_decide<br>8.3.1 If yes, who decided how to spend this credit most of the time?<br>ଯଦି ହଁ ,ଅଧିକାଂଶ ସମୟ ସେ ଅର୍ଥକୁ କିପରି ଖର୍ଚ୍ଚ କରାଯିବ ତାର ନିଷ୍ପତି କିଏ ନିଅନ୍ତି?	<multiple answer/ବହୁ ଉତ୍ତର ସମ୍ଭବ><br>Self/ନିଜେ--------------------------- 1<br>Spouse/ସ୍ୱାମୀ/ସ୍ତ୍ରୀ-------------------- 2<br>Other HH member/ପରିବାରର ଅନ୍ୟ ସଦସ୍ୟ-- 3<br>Other non-HH member/ଅନ୍ୟ ବାହାର ବ୍ୟକ୍ତି---- 4<br>Not applicable/ଉପଯୁକ୍ତ ନୁହେଁ---- 98	☐<br>☐<br>☐<br>☐

୨ **TIME USE/ସମୟର ଉପଯୋଗ**

Instructions: If yesterday was a typical day ask the respondent about yesterday. If yesterday was atypical, but the day before typical, please ask the respondent to consider the day before's activities. If both days were atypical, then please ask the respondent to consider yesterday's activities.

ଯଦି ଗତକାଲି ଗୋଟିଏ ବିଶେଷ ଦିନ ହୋଇଥିବ ତେବେ ଉତ୍ତରଦାତାଙ୍କୁ ଗଲା କାଲି ବିଷୟରେ ପଚାରନ୍ତୁ । ଯଦି ଗଲା କାଲିର ପୂର୍ବଦିନଟି ବିଶେଷ ଦିନ ହୋଇ ଥାଏ ତେବେ ଉତ୍ତରଦାତାଙ୍କୁ ସେହି ଦିନର କାର୍ଯ୍ୟକଳାପ ବିଷୟରେ ପଚାରନ୍ତୁ।ଯଦି ଉଭୟ ୨ ଦିନ କୌଣସି ବିଶେଷ ଦିନ ହୋଇଥାଏ, ତେବେ ଉତ୍ତରଦାତାଙ୍କୁ ଗତକାଲି ବିଷୟରେ ପଚାରନ୍ତୁ ।

Please record a log of the activities for the individual in the last complete 24 hours (starting yesterday morning at 4 am, finishing 3:59 am of the current day). The time intervals are marked in 30 min intervals. <u>Mark one activity for each time period</u> by entering the corresponding activity code in the box.Check the box below if the respondent was caring for children while performing each activity.

Now I'd like to ask you about how you spent your time during the past 24 hours. We'll begin from yesterday morning, and continue through to this morning. This will be a detailed accounting. I'm interested in everything you did (i.e. resting, eating, personal care, work inside and outside the home, caring for children, cooking, shopping, socializing, etc.), even if it didn't take you much time.I'm particularly interested in agricultural activities such as farming, gardening, and livestock raising whether in the field or on the homestead. I'm also interested in how much time you spent caring for children, especially if it happened while you did some other activity (e.g., collecting water while carrying a child or cooking while watching after a sleeping child).

ଦୟାକରି ସମ୍ପୂର୍ଣ ୨୪ ଘଣ୍ଟାର କାର୍ଯ୍ୟକଳାପର ଏକ ସାରଣୀ ରଖନ୍ତୁ (ଗତକାଲି ରାତି ୪ଟାରୁ ଆରମ୍ଭହୋଇ ଆଜିର ୩.୫୯ ପର୍ଯ୍ୟନ୍ତ) ସମୟ ନିର୍ଦ୍ଧାରଣ ୩୦ ମିନିଟ ଅନ୍ତରରେ ରଖାଯାଇଅଛି । ପ୍ରତ୍ୟେକ କାର୍ଯ୍ୟକଳାପ ନିମନ୍ତେ ପୃଥକ ପୃଥକ ସମୟ ନିର୍ଦ୍ଧାରଣ ହୋଇଛି ।

ପ୍ରତ୍ୟେକ କାର୍ଯ୍ୟ ନିମନ୍ତେ ନିର୍ଦ୍ଧାରିତ କୋଡକୁ ନିମ୍ନରେ ଦିଆଯାଇଥିବା କୋଠରୀରେ ଲେଖନ୍ତୁ ଓ ଦେଖନ୍ତୁ ଏହି କାର୍ଯ୍ୟ କଲାବେଳେ ସେ ଶିଶୁର ଯତ୍ନ ନେଉଛନ୍ତି କି ?

ବର୍ତ୍ତମାନ ମୁଁ ଆପଣଙ୍କୁ ଗତ ୨୪ ଘଣ୍ଟା ମଧ୍ୟରେ ଆପଣ କେଉଁ କେଉଁ କାମ କରିଛନ୍ତି ସେ ବିଷୟରେ ପଚାରିବି । ଆମେ ଗତକାଲି ସକାଳରୁ ଆରମ୍ଭ କରିବା ଓ ଧୀରେ ଧୀରେ ଆଜି ସକାଳ ପର୍ଯ୍ୟନ୍ତ ଯିବା । ଏହା ଏକ ସମ୍ପୂର୍ଣ ବିବରଣୀ/ଆକଳନ ହେବ । ମୁଁ ଆପଣଙ୍କ ସବୁ ଟିକିନିଖି କାର୍ଯ୍ୟକଳାପ ଜାଣିବାକୁ ଚାହେଁ । ଯଥା: ବିଶ୍ରାମ ନେବା, ଖାଇବା, ନିଜର ଯତ୍ନ ନେବା, ଘରେ ଓ ବାହାରେ କାର୍ଯ୍ୟ କରିବା, ପିଲାମାନଙ୍କ ଯତ୍ନ ନେବା, ରୋଷେଇ କରିବା, ବଜାର କରିବା ଓ ସାମାଜିକ କାମ କରିବା। ଏଗୁଡିକରେ ମୁଁ ବେଶି ସମୟ ନେବି ନାହିଁ । ଆପଣଙ୍କ କୃଷି କାର୍ଯ୍ୟକଳାପ ଯଥା: ଚାଷ କରିବା, ବଗିଚା କରିବା, ପଶୁ ସମ୍ପଦ ପାଳନ କରିବା କାମ ଜାଣିବାରେ ମୁ ବେସୀ ଆଗ୍ରହୀ ଏହା ବିଲରେ ହେଉ ବା ଘରେ ହେଉ । ମୁଁ ଏହା ମଧ୍ୟ ଜାଣିବାକୁ ଚାହେଁ ଆପଣ ପିଲାଙ୍କ ଯତ୍ନ ନେବାରେ କେତେ ସମୟ ଦେଉଛନ୍ତି ବିଶେଷକରି ଆପଣ ଯେତେବେଳେ ଅନ୍ୟକିଛି କାର୍ଯ୍ୟ କରୁଛନ୍ତି ତା ସହିତ ପିଲାର ଯତ୍ନ କିପରି ନେଉଛନ୍ତି । ଯଥା: ପିଲାର ଯତ୍ନ ନେବାବେଳେ ପାଣି ଆଣିବା ବା ରୋଷେଇ କଲାବେଳେ ଶୋଇଲା ପିଲାପ୍ରତି ନଜର ରଖିବା ।

Variable name	Question	Answer
weai_time_yday<br>9.1 Was yesterday (the last 24 hours) a typical day where you worked (either at home or outside the home) about the same as usual? ଗତକାଲି (ଗତ 24 ଘଣ୍ଟା) ଏକ ସାଧାରଣ ଦିନ ଥିଲା କି ଯେଉଁ ଦିନ ଆପଣ ଘରେ ହେଉ ବା ବାହାରେ ହେଉ ଅନ୍ୟ ଦିନ ଭଳି ସମାନ କାମ କରିଥିଲେ ?	Yes/ହଁ----------------- 1<br>→*Go to 9.3*<br>No/ନା---------------- 0<br>→*Go to 9.2*	☐
weai_time_daybefore<br>9.2 Was the day before yesterday a typical day where you worked (either at home or outside the home) about the same as usual? ପହରଦିନ ଏକ ସାଧାରଣ ଦିନ ଥିଲା କି ଯେଉଁ ଦିନ ଆପଣ ଘରେ ହେଉ ବା ବାହାରେ ହେଉ ଅନ୍ୟ ଦିନ ଭଳି ସମାନ କାମ କରିଥିଲେ ?	Yes/ହଁ----------------- 1<br>→*Go to 9.3*<br>No/ନା---------------- 0<br>→*Go to 9.3*	☐

	**Night**				**Morning**		**Day**																	
	04:00		05:00		06:00		07:00		08:00		09:00		10:00		11:00		12:00		13:00		14:00		15:00	
Activity **(WRITE ACTIVITY CODE)** (କାର୍ଯ୍ୟକଳାପର କୋଡ ଲେଖନ୍ତୁ)																								
9.3 Did you also care for children/ଆପଣ ପିଲାଙ୍କ ଯତ୍ନ ମଧ୍ୟ ନିଅନ୍ତି କି?<br>Yes/ହଁ--------------------- 1<br>No/ନା--------------------- 0																								

	**Day**				**Evening**		**Night**																	
	16:00		17:00		18:00		19:00		20:00		21:00		22:00		23:00		24:00		01:00		02:00		03:00	
Activity **(WRITE ACTIVITY CODE)** (କାର୍ଯ୍ୟକଳାପର କୋଡ ଲେଖନ୍ତୁ)																								
Did you also care for children/ଆପଣ ପିଲାଙ୍କ ଯତ୍ନ ମଧ୍ୟ ନିଅନ୍ତି କି?<br>Yes/ହଁ--------------------- 1<br>No/ନା--------------------- 0																								

A= Sleeping and resting/ଶୋଇବା ଏବଂ ବିଶ୍ରାମ ନେବା<br>B= Eating, drinking, or other relaxing leisure activities including social or religious activities/ଖାଇବା, ପିଇବା, ଫୁର୍ତ୍ତି କରିବା, ପୂଜା କରିବା<br>C= Personal care (e.g. dressing, showering)/ନିଜର ଯତ୍ନ (ଯଥା: ସଜବାଜ ହେବା, ଗାଧୋଇବା)<br>D= School / studies/ବିଦ୍ୟାଳୟ/ପଢିବା<br>E= Office / shop business work or other light non-farm wage labour e.g. barber, shop, blacksmith/ଅଫିସ/ଦୋକାନ ବ୍ୟବସାୟ ବା ଅନ୍ୟାନ୍ୟ ଅଣ-କୃଷି ଶ୍ରମ ଯଥା: ଭଣ୍ଡାରୀ, ଦୋକାନ, କମାର<br>F= Physically strenuous non-farm labour, e.g. wage labour like construction, work in mines or delivering heavy goods./ଶାରୀରିକ ବଳ ପ୍ରୟୋଗକରି ଅଣ-କୃଷି ଶ୍ରମ, ଯଥା: ଦିନ ମଜୁରିଆ ଯେପରି ନିର୍ମାଣ କାର୍ଯ୍ୟ, ଖଣିରେ କାମ କରିବା, ଭାରି ଜିନିଷ ଉଠାଇବା<br>G = Traveling / commuting/ଯାତ୍ରା କରିବା/ ଯିବା ଆସିବା କରିବା<br>H = Heavy / strenuous agricultural work (e.g. digging, hoeing, heavy lifting, spraying, threshing)/ଭାରି କୃଷିକାର୍ଯ୍ୟ ଯଥା: ଖୋଳିବା, ଭାରି ଜିନିଷ ଉଠାଇବା, ସ୍ପ୍ରେ କରିବା,ଧାନ କାଟିବା I	I = Mild / moderately strenuous agricultural work (e.g. bundling rice, harvesting, planting / transplanting, weeding, winnowing, ploughing with tractor or bullock)/ଅଳ୍ପ ଶ୍ରମଦ୍ୱାରା କୃଷିକାର୍ଯ୍ୟ(ଯଥା: ଧାନ ଗୋଛା କରିବା, ଅମଳ କରିବା, ଧାନ ରୋଇବା, ତଳି ପକାଇବା, ବଳଦ କିମ୍ବା ଟ୍ରାକ୍ଟର ଦ୍ୱାରା ହଳ କରିବା)<br>J = Livestock raising and fishpond culture/ପଶୁସମ୍ପଦ ପାଳନ କରିବା ଏବଂ ମାଛ ଚାଷ କରିବା<br>K = Foraging or hunting /ଶିକାର କରିବା<br>L = Collecting water / wood / other load/ ପାଣି/କାଠ/ ଅନ୍ୟନ ସାମଗ୍ରୀ ସଂଗ୍ରହ କରିବା<br>M = Food preparation / cooking/ଖାଦ୍ୟ ପ୍ରସ୍ତୁତ କରିବା/ରୋଷେଇ କରିବା<br>N = Other paid or unpaid domestic work (e.g. cleaning, caring for children or others)/ ଅନ୍ୟାନ୍ୟ ଦେୟ ଓ ଅଣଦେୟ ଘରୋଇ କାର୍ଯ୍ୟ (ସଫା କରିବା, ପିଲାଙ୍କ ଯତ୍ନନେବା ଓ ଅନାନ୍ୟ)<br>O= Defecation (include time taken to travel to field if open defecation)/ମଳତ୍ୟାଗ କରିବାକୁ ଯିବା(ଯିବା ଆସିବା ସମୟକୁ ମିଶାଇ)<br>X = Other/ ଅନ୍ୟାନ୍ୟ (ଦର୍ଶାନ୍ତୁ)

10.Group membership/ଗୋଷ୍ଠୀ ସଦସ୍ୟତା

Now I'm going to ask you about groups in the community. These can either be formal or informal or customary groups/ବର୍ତ୍ତମାନ ମୁଁ ଆପଣଙ୍କୁ ଗ୍ରାମରେ ଥିବା ଗୋଷ୍ଠୀ ଗୁଡିକ ବିଷୟରେ ପଚାରିବି । ଏହା ଆନୁଷ୍ଠାନିକ କିମ୍ବା ଅଣ-ଆନୁଷ୍ଠାନିକ ହୋଇପାରେ ।.

Group	Options	Answer	Question	Answer
Are there any of the following groups/cooperatives in your village or nearby?<br>ଆପଣଙ୍କ ଗ୍ରାମରେ/ଗ୍ରାମ ପାଖରେ କୌଣସି ଗୃପବା ସୋସାଇଟିଅଛିକି ?<br><br>Read out all of the options/ ବିକଳ୍ପ ଗୁଡିକୁ ପଢନ୍ତୁ			If yes, have you been an active member of this group in the last 3 agricultural seasons (June 2015 to May 2016)?<br>ଯଦି ହଁ, ଅପଣ ସେଥିରେ ଗତ ଜୁନ, ୨୦୧୫ ରୁ ମେ, ୨୦୧୬ ମଧ୍ୟରେ ଜଣେ ସଦସ୍ୟ ଅଛନ୍ତିକି ? Please explain that "active member" means one who attends meetings, participates in discussions, and volunteers. ସଦସ୍ୟ ଅର୍ଥାତ, ସଭାରେ ଉପସ୍ଥିତ ରହୁଥିବେ, ନିଷ୍ପତ୍ତି ଗ୍ରହଣରେ ଅଂଶଗ୍ରହଣ କରୁଥିବେ ।	
weai_groups_a<br>10.1. Farmers' club (NABARD), milk producer's cooperatives or large area multi-purpose cooperative society (LAMPS)/କୃଶକ ସଙ୍ଘ (ନାବାର୍ଡ),ଦୁଗ୍ଧ ଉତ୍ପାଦନ ସମବାୟ ସଙ୍ଘ କିମ୍ବା ଲ୍ୟାମ୍ପ	Yes/ହଁ--------- 1<br>No/ନା-------- 0<br>Don't know / ଜଣା ନାହିଁ-----98	☐	10.1.1.<br><br>Yes/ହଁ--------- 1<br>No/ନା-------- 0	☐
weai_groups_b<br>10.2 Water or sanitation group (Pani Panchayat), watershed committee or Gaon Kalyan Samiti (GKS) / Village Health Sanitation Committee/ପାଣି ପଞ୍ଚାୟତ, ଜଳବିଭାଜିକା, ଗାଁ କଲ୍ୟାଣ ସମିତି/ସ୍ୱାସ୍ଥ୍ୟ ପରିମଳ ସମିତି	Yes/ହଁ--------- 1<br>No/ନା-------- 0<br>Don't know / ଜଣା ନାହିଁ-----98	☐	10.2.1.<br><br>Yes/ହଁ--------- 1<br>No/ନା-------- 0	☐
weai_groups_c<br>10.3 Forest users' or protection group (including marketing)/ ଜଙ୍ଗଲ ସଂରକ୍ଷଣ ସମିତି (ଜଙ୍ଗଲଜାତ ଦ୍ରବ୍ୟରର ବିକା କିଣା)	Yes/ହଁ--------- 1<br>No/ନା-------- 0<br>Don't know / ଜଣା ନାହିଁ-----98	☐	10.3.1<br><br>Yes/ହଁ--------- 1<br>No/ନା-------- 0	☐
weai_groups_d<br>10.4 Credit or micro-finance institution/ଋଣ ଦେବା ସଂସ୍ଥା କିମ୍ବା ମାଇକ୍ରୋଫାଇନାନ୍ସ ସଂସ୍ଥା	Yes/ହଁ--------- 1<br>No/ନା-------- 0<br>Don't know / ଜଣା ନାହିଁ-----98	☐	10.4.1<br><br>Yes/ହଁ--------- 1<br>No/ନା-------- 0	☐

Group	Options	Answer	Question	Answer
Are there any of the following groups/cooperatives in your village or nearby?<br>ଆପଣଙ୍କ ଗ୍ରାମରେ/ଗ୍ରାମ ପାଖରେ କୌଣସି ଗୃପବା ସୋସାଇଟିଅଛିକି ?<br><br>Read out all of the options/ ବିକଳ୍ପ ଗୁଡିକୁ ପଢନ୍ତୁ			If yes, have you been an active member of this group in the last 3 agricultural seasons (June 2015 to May 2016)?<br>ଯଦି ହଁ, ଅପଣ ସେଥିରେ ଗତ ଜୁନ, ୨୦୧୫ ରୁ ମେ, ୨୦୧୬ ମଧ୍ୟରେ ଜଣେ ସଦସ୍ୟ ଅଛନ୍ତିକି ? Please explain that "active member" means one who attends meetings, participates in discussions, and volunteers. ସଦସ୍ୟ ଅର୍ଥାତ, ସଭାରେ ଉପସ୍ଥିତ ରହୁଥିବେ, ନିଷ୍ପତ୍ତି ଗ୍ରହଣରେ ଅଂଶଗ୍ରହଣ କରୁଥିବେ ।	
weai_groups_e<br>10.5 Village development group/ଗ୍ରାମ ଉନ୍ନୟନ ଗୋଷ୍ଠୀ	Yes/ହଁ--------- 1<br>No/ନା-------- 0<br>Don't know / ଜଣା ନାହିଁ-----98	☐	10.5.1<br><br>Yes/ହଁ--------- 1<br>No/ନା-------- 0	☐
weai_groups_f<br>10.6 Religious groups like Bhajan Mandali or Satsang/ଧାର୍ମିକ ଅନୁଷ୍ଠାନ ଯେପରି ଭଜନ ମଣ୍ଡଳ, ସତସଙ୍ଗ	Yes/ହଁ--------- 1<br>No/ନା-------- 0<br>Don't know / ଜଣା ନାହିଁ-----98	☐	10.6.1<br><br>Yes/ହଁ--------- 1<br>No/ନା-------- 0	☐
weai_groups_g<br>10.7 SHG (Women's / men's self-help group)/ସ୍ୱୟଂ ସହାୟକ ଗୋଷ୍ଠୀ (ମହିଳା/ପୁରୁଷଙ୍କ ସ୍ୱୟଂ ସହାୟକ ଗୋଷ୍ଠୀ)	Yes/ହଁ--------- 1<br>No/ନା-------- 0<br>Don't know / ଜଣା ନାହିଁ-----98	☐	10.7.1<br><br>Yes/ହଁ--------- 1<br>No/ନା-------- 0	☐
weai_groups_h<br>10.8 School-based groups such as mother & teacher association, or parent & teacher association, or a school committee/ବିଦ୍ୟାଳୟ ଭିତ୍ତିକ ଗୋଷ୍ଠୀ ଯେପରି ମା ଏବଂ ଶିକ୍ଷକ ସଂଘ କିମ୍ବା ପିତାମାତା ଏବଂ ଶିକ୍ଷକ ସଂଘ, ସ୍କୁଲ କମିଟି	Yes/ହଁ--------- 1<br>No/ନା-------- 0<br>Don't know / ଜଣା ନାହିଁ-----98	☐	10.8.1<br><br>Yes/ହଁ--------- 1<br>No/ନା-------- 0	☐
weai_groups_i<br>10.9 Nutrition-related groups like mothers' committee or Jaanch (audit) committee/ ପୋଷଣ ସମନ୍ଧୀୟ ଗୋଷ୍ଠି, ମାତୃ କମିଟି/ଯାଞ୍ଚ କମିଟି	Yes/ହଁ--------- 1<br>No/ନା-------- 0<br>Don't know / ଜଣା ନାହିଁ-----98	☐	10.9.1<br><br>Yes/ହଁ--------- 1<br>No/ନା-------- 0	☐
weai_groups_j<br>10.10 Youth club/ଯୁବକ ସଂଘ	Yes/ହଁ--------- 1<br>No/ନା-------- 0<br>Don't know / ଜଣା ନାହିଁ-----98	☐	19.10.1<br><br>Yes/ହଁ--------- 1<br>No/ନା-------- 0	☐

11. Land ownership/ଜମି ମାଲିକାନା-

Variable name	Question	Code	Answer
land_own/ନିଜସ୍ୱ ଜମି	11.1 Does your household currently own any land?<br>ବର୍ତ୍ତମାନ ଆପଣଙ୍କ ପରିବାରର ନିଜସ୍ୱ ଜମି ଅଛି କି ?<br>Note: Land includes homestead land, agriculture land and any other land.<br>ସୂଚନା: ଘରବାରୀ, ଚାଷଜମି ଏବଂ ଅନ୍ୟ ଜମିକୁ ମିଶାଇ ।	Yes, has legal ownership with Record of Rights (RoRs)/ହଁ, ଜମି ପଟ୍ଟାଧାରୀ------1<br>Yes, has a share of land but in ancestral name/ ହଁ, ଭାଗ ଅଛି କିନ୍ତୁ ବାପା/ ଦାଦା/ ଜେଜେବାପା ନାମରେ ରହିଛି ---------- 2<br>Yes, has a share of land (or owns land through other means) but no record/ ହଁ, ଭାଗ ଅଛି କିନ୍ତୁ ପଟ୍ଟା ନାହିଁ-----------------3<br>No/ନା-------------------------------------- 0<br>*→ Go to Q11.9*	<multiple response><br><ଏକାଧିକ ଉତ୍ତର ସମ୍ଭବ><br>☐<br>☐<br>☐
land_size	11.2 In total, how much land does your household currently own (including land with record and without record)?<br>ଆପଣଙ୍କ ପରିବାରର ବର୍ତ୍ତମାନ ସମୁଦାୟ କେତେ ଜମି ଅଛି (ନିଜ ନାଁରେ ରେକର୍ଡ ହୋଇଥିବାଜମି ଏବଂ ରେକର୍ଡ ହୋଇନଥିବା ଜମି ଯୋଗ କରନ୍ତୁ?		☐☐.☐<br>(in acres/ଏକରରେ)
weai_asset_land agri	11.3 Of this total land, is any of it cultivable (including small kitchen garden plots)/ଏହି ସମସ୍ତ ଜମିରୁ କିଛି ଚାଷ ଉପଯୋଗୀ ଜମି କି? (ଘରୋଇ ବାଡି ବଗିଚାକୁ ମିଶାଇ)	Yes/ହଁ-----------------------------1<br>No/ନା-----------------------------0	☐
land_sharecrop	11.4 Did you share / mortgage / lease any land out to anybody in last three agricultural seasons i.e. June 2015 to May 2016?<br>ଗତ 3 କୃଷି ଋତୁରେ ଆପଣ ଏହି ଜମି କୁ ଭାଗ/ବନ୍ଧା/ଲିଜରେ କାହାକୁ ଦେଇଥିଲେ କି?(ଜୁନ 2015 ଠାରୁ ମେ 2016 ପର୍ଯ୍ୟନ୍ତ)	Yes/ହଁ-----------------------------1<br>No/ନା-----------------------------0<br>*→ Go to Q11.6*	☐
land_sharecrop_income	11.5 What was the value of agricultural production or cashyou received from leasing out/ mortgaging out/ sharing out land in last three agricultural seasonsi.e. June 2015 to May 2016?<br>ଗତ 3ଟି କୃଷି ଋତୁରେ ଭାଗ/ବନ୍ଧା/ଲିଜ ଦେଇଥିବା ଜମିର କୃଷି ଉତ୍ପାଦନରୁ ଆପଣ କେତେ ମୂଲ୍ୟ/ଟଙ୍କା ପାଇଥିଲେ? ଜୁନ, ୨୦୧୫ ରୁ ମେ, ୨୦୧୬		☐<br>Rs/ଟଙ୍କା

Variable name	Question	Code	Answer
weai_asset_land agri_own	11.6 Do you personally (jointly or solely) own any of the agriculture / cultivable land that your household has?<br>ଆପଣଙ୍କ ପରିବାରର କୃଷି ଉପଯୋଗୀ ଜମି ରୁ ଆପଣଙ୍କର ବ୍ୟକ୍ତିଗତ/ନିଜସ୍ୱ(ଏକାକୀ/ମିଳିତ ଭାବରେ) ଜମି ଅଛି କି ?	Yes, solely/ ହଁ, ଏକାକୀ------------------- 1<br>Yes, jointly/ ହଁ,ମିଳିତ ଭାବେ ଭାବେ------- 2<br>No/ନା------------------------------------- 0	☐
weai_asset_land _ot	11.7 Does your household own any other land not used for agriculture (pieces/plots, residential or commercial land)?<br>ଆପଣଙ୍କ ପରିବାରର ଏପରି କିଛି ଜମି ରହିଛି କି, ଯାହା କୃଷି କାର୍ଯ୍ୟ ନିମନ୍ତେ ବ୍ୟବହାର ହେଉନାହିଁ ? (ପ୍ଲଟ, ଗୃହ କିମ୍ବା ବ୍ୟବସାୟ ଭିତ୍ତିକ ଜମି)	Yes/ହଁ------------------------------1<br>No/ନା------------------------------0	☐
weai_asset_land _ot_own	11.8 Do you personally (jointly or solely) ownany other land not used for agriculture (pieces/plots, residential or commercial land)<br>ଆପଣଙ୍କର ନିଜର(ଏକାକୀ କିମ୍ବା ମିଳିତ) କୌଣସି ଜମି ଅଛି କି, ଯାହା କୃଷି କାର୍ଯ୍ୟରେ ବ୍ୟବହୃତ ହେଉନାହିଁ?(ପ୍ଲଟ,ଗୃହ କିମ୍ବା ବ୍ୟବସାୟ ଭିତିକ ଜମି)	Yes, solely/ହଁ, ଏକାକୀ------------------ 1<br>Yes, jointly/ହଁ,ମିଳିତ ଭାବେ ଭାବେ------ 2<br>No/ନା----------------------------------- 0	☐
**Land use/**ଜମିର ବ୍ୟବହାର<br>(Recall period is the last 3 seasonsi.e.June 2015 to May 2016/ସେହି ସମୟ କୁ ମନେ ପକାନ୍ତୁ ଜୁନ, ୨୦୧୫ ଠାରୁ ମେ, ୨୦୧୬ ମଧ୍ୟରେ**)**<br><br>***Now involve other household members who are willing and available to input on this/***ବର୍ତ୍ତମାନ ପରିବାରର ଅନ୍ୟ ସଦସ୍ୟଙ୍କୁ ସାକ୍ଷାତକାରରେ ଅଂଶଗ୍ରହଣ କରିବାକୁ ସୁଯୋଗ ଦେଇପାରନ୍ତି (ଯେଉଁମାନେ ଅଂଶଗ୍ରହଣ କରିବାକୁ ଇଚ୍ଛାପ୍ରକାଶ କରିଥିଲେ ଏବଂ ଉପସ୍ଥିତ ଅଛନ୍ତି)			
land_cultivate	11.9 Did you cultivate any land (whether or not it is owned) in the last 3 agricultural seasons?<br>ଗତ ୩ଟି କୃଷି ଋତୁରେ ଆପଣ କୌଣସି ଜମି ଚାଷ କରିଥିଲେ କି (ତାହା ଆପଣଙ୍କ ନିଜସ୍ୱହୋଇ ଥାଉ ବା ନ ଥାଉ)?	Yes/ହଁ-----------------------------1<br>No/ନା------------------------------0<br>→Go to section 13	☐
land_cultivate_own	11.10 How much of the household **owned land** did you cultivate during the last three agricultural seasons?<br>ଆପଣଙ୍କ ନାମରେ ଥିବା କେତେ ଜମି ଗଲା 3 ଟି କୃଷି ଋତୁରେ ଚାଷ କରିଥିଲେ?		☐☐.☐☐<br>(in acres/ଏକରରେ)
land_cultivate_share	11.11 How much **leased / shared out / mortgaged out land** did your household cultivate during the last three agricultural seasons?<br>ଗତ ୩ଟି କୃଷି ଋତୁରେ ବନ୍ଧାପଡିଥିବା ଜମି/ଲିଜ ଜମି ଆପଣ କେତେ ଚାଷ କରିଥିଲେ ?		☐☐.☐☐<br>(in acres/ଏକରରେ)

Variable name	Question	Code	Answer
land_cultivate_commons	11.12 How much **encroached, forest land** or government land did your household cultivate during the last three agricultural seasons?<br>ଆପଣ କେତେ ଅନଧିକାର ଦଖଲ ଜମି/ଜଙ୍ଗଲ ଜମି/ସରକାରୀ ଜମି ଗତ ତିନୋଟି କୃଷି ଋତୁରେ ଚାଷ କରିଥିଲେ ?		☐☐.☐☐<br>(in acres/ଏକରରେ)
land_cultivate_other	11.13 How much **other land** (including extended family and other's land) did your household cultivate during last three agricultural seasons?<br>ଆପଣ କେତେ ଅନ୍ୟାନ୍ୟ ଜମି(ଭାଇ ବନ୍ଧୁ କୁଟୁମ୍ବ କିମ୍ବା ଅନ୍ୟ ମାନଙ୍କ ଜମି)ଗତ ୩ଟି କୃଷି ଋତୁରେ ଚାଷ କରିଥିଲେ ?		☐☐.☐☐<br>(in acres/ଏକରରେ)
land_irrigate	11.14 How much of the land that you cultivated (both owned land and land not owned) in the last three agricultural seasons was irrigated, if any?<br>ଗତ ତିନୋଟି କୃଷି ଋତୁରେ ଆପଣ ଚାଷ କରିଥିବା ଜମିରୁ କେତେ ଜମି ଚାଷ ହୋଇଥିଲା (ଉଭୟ ନିଜର ଜମି ଓ ନିଜର ହୋଇନଥିବା ଜମି)?<br>If no land was irrigated, record '0'.<br>ଯଦି କୌଣସି ଜମି ଜଳସେଚିତ ନୁହେଁ, ୦ ଲେଖନ୍ତୁ		☐☐.☐☐<br>(in acres/ଏକରରେ)

12. Household agriculture production & income from the last year (June 2015 to May 2016)
ଗତ ଏକବର୍ଷ ମଧ୍ୟରେ ପରିବାରର କୃଷି ଉତ୍ପାଦନ ଏବଂ ଆୟ (ଜୁନ,୨୦୧୫ ରୁ ମେ, ୨୦୧୬)

Involve other household members who are willing and available to input on this / ପରିବାରର ଉପସ୍ଥିତ ଥିବା ଏବଂ ଇଛାପ୍ରକାଶ କରୁଥିବା ଅନ୍ୟ ସଦସ୍ୟ ମାନଙ୍କୁ ମଧ୍ୟ ପଚାରନ୍ତୁ

Ask about cereals, pulses, vegetables, and nuts/ଶସ୍ୟ, ଡାଲି, ପନିପରିବା ଓ ବାଦାମ ବିଷୟରେ ପଚାରନ୍ତୁ

Crop / vegetable code /ଶସ୍ୟ/ପନିପରିବା କୋଡ	Total yield	Unit/ *ଏକକ*	HH share of total yield (excluding post harvest losses) ସମସ୍ତ ଉତ୍ପାଦନରୁ ପରିବାରର ଭାଗ (ଅମଳ ପରେ ହୋଇଥିବା କ୍ଷତିକୁ ବାଦଦେଇ)	Quantity consumed or used by household (food or other uses, e.g. fodder)/ ପରିବାରରେ ଖାଇଥିବା କିମ୍ବା ବ୍ୟବହାର ହୋଇଥିବା ସାମଗ୍ରୀର ପରିମାଣ (ଖାଦ୍ୟ କିମ୍ବା ଅନ୍ୟକିଛି ବ୍ୟବହାର)	Quantity stored ଗଚ୍ଛିତ ହୋଇଥିବା ଉତ୍ପାଦନର ପରିମାଣ	Quantity given as gifts or transfers or in-kind payments ଦାନରେ ଦିଆଯାଇଥିବ ବା ପଠାଯାଇଥିବା ର ପରିମାଣ ବା ଜିନିଷ ଆକାରରେ ଦିଆଯାଇଥିବା ପରିମାଣ	Quantity sold (including value added products)/ ବିକ୍ରି ହୋଇଥିବାର ପରିମାଣ (ମୂଲ୍ୟ ଉପଯୋଗ କରାଯାଇଥିବା ଉତ୍ପାଦନକୁ ମିଶାଇ) → *If 0 sold, skip to next crop*	Quantity sold that was value-added, if any/ ମୂଲ୍ୟ ଉପଯୋଗ କରାଯାଇ ବିକ୍ରି ହୋଇଥିବା ଉତ୍ପାଦନର ପରିମାଣ	If any sold, what income did you make? (no value addition) (Rs.) ଯଦି ବିକ୍ରି ହେଲା ସେଥିରୁ କେତେ ଆୟ କଲେ (ବିନା ମୂଲ୍ୟ ଉପଯୋଗ କରି)(ଟଙ୍କାରେ)	If any value-added products sold, what income did you make from the sale? (Rs.) ଯଦି ମୂଲ୍ୟ ଉପଯୋଗ କରାଯାଇଥିବା ଉତ୍ପାଦନ ବିକ୍ରି ହେଲା ସେଥିରୁ କେତେ ଆୟ କଲେ?(ଟଙ୍କାରେ)
**Kharif/** ଖରିଫ(ବର୍ଷା ଦିନିଆ ଫସଲ)										
☐☐		☐☐☐								
☐☐		☐☐☐								
☐☐		☐☐☐								
☐☐		☐☐☐								
☐☐		☐☐☐								
☐☐		☐☐☐								
☐☐		☐☐☐								
☐☐		☐☐☐								
☐☐		☐☐☐								
☐☐		☐☐☐								
☐☐		☐☐☐								

Crop / vegetable code /ଶସ୍ୟ/ପନି ପରିବା କୋଡ	Total yield	Unit/ ଏକକ	HH share of total yield (excluding post harvest losses) ସମସ୍ତ ଉତ୍ପାଦନରୁ ପରିବାରର ଭାଗ (ଅମଳ ପରେ ହୋଇଥିବା କ୍ଷତିକୁ ବାଦଦେଇ)	Quantity consumed or used by household (food or other uses, e.g. fodder)/ ପରିବାରରେ ଖାଇଥିବା କିମ୍ବା ବ୍ୟବହାର ହୋଇଥିବା ସାମଗ୍ରୀର ପରିମାଣ (ଖାଦ୍ୟ କିମ୍ବା ଅନ୍ୟକିଛି ବ୍ୟବହାର)	Quantity stored ଗଚ୍ଛିତ ହୋଇଥିବା ଉତ୍ପାଦନର ପରିମାଣ	Quantity given as gifts or transfers or in-kind payments ଦାନରେ ଦିଆଯାଇଥିବ ବା ପଠାଯାଇଥିବା ର ପରିମାଣ ବା ଜିନିଷ ଆକାରରେ ଦିଆଯାଇଥିବା ପରିମାଣ	Quantity sold (including value added products)/ ବିକ୍ରି ହୋଇଥିବାର ପରିମାଣ (ମୂଲ୍ୟ ଉପଯୋଗ କରାଯାଇଥିବା ଉତ୍ପାଦନକୁ ମିଶାଇ) → *If 0 sold, skip to next crop*	Quantity sold that was value-added, if any/ ମୂଲ୍ୟ ଉପଯୋଗ କରାଯାଇ ବିକ୍ରି ହୋଇଥିବା ଉତ୍ପାଦନର ପରିମାଣ	If any sold, what income did you make? (no value addition) (Rs.) ଯଦି ବିକ୍ରି ହେଲା ସେଥିରୁ କେତେ ଆୟ କଲେ (ବିନା ମୂଲ୍ୟ ଉପଯୋଗ କରି)(ଟଙ୍କାରେ)	If any value-added products sold, what income did you make from the sale? (Rs.) ଯଦି ମୂଲ୍ୟ ଉପଯୋଗ କରାଯାଇଥିବା ଉତ୍ପାଦନ ବିକ୍ରି ହେଲା ସେଥିରୁ କେତେ ଆୟ କଲେ?(ଟଙ୍କାରେ)
**Kharif/** ଖରିଫ(ବର୍ଷା ଦିନିଆ ଫସଲ)										
☐☐		☐☐☐								
☐☐		☐☐☐								
☐☐		☐☐☐								
☐☐		☐☐☐								
☐☐		☐☐☐								
☐☐		☐☐☐								
☐☐		☐☐☐								
☐☐		☐☐☐								
☐☐		☐☐☐								
☐☐		☐☐☐								
☐☐		☐☐☐								
☐☐		☐☐☐								
☐☐		☐☐☐								

Crop / vegetable code /ଶସ୍ୟ/ପନିପରିବା କୋଡ	Total yield	Unit/ ଏକକ	HH share of total yield (excluding post harvest losses) ସମସ୍ତ ଉତ୍ପାଦନରୁ ପରିବାରର ଭାଗ (ଅମଳ ପରେ ହୋଇଥିବା କ୍ଷତିକୁ ବାଦଦେଇ)	Quantity consumed or used by household (food or other uses, e.g. fodder)/ ପରିବାରରେ ଖାଇଥିବା କିମ୍ବା ବ୍ୟବହାର ହୋଇଥିବା ସାମଗ୍ରୀର ପରିମାଣ (ଖାଦ୍ୟ କିମ୍ବା ଅନ୍ୟକିଛି ବ୍ୟବହାର)	Quantity stored ଗଚ୍ଛିତ ହୋଇଥିବା ଉତ୍ପାଦନର ପରିମାଣ	Quantity given as gifts or transfers or in-kind payments ଦାନରେ ଦିଆଯାଇଥିବ ବା ପଠାଯାଇଥିବା ର ପରିମାଣ ବା ଜିନିଷ ଆକାରରେ ଦିଆଯାଇଥିବା ପରିମାଣ	Quantity sold (including value added products)/ ବିକ୍ରି ହୋଇଥିବାର ପରିମାଣ (ମୂଲ୍ୟ ଉପଯୋଗ କରାଯାଇଥିବା ଉତ୍ପାଦନକୁ ମିଶାଇ) → *If 0 sold, skip to next crop*	Quantity sold that was value-added, if any/ ମୂଲ୍ୟ ଉପଯୋଗ କରାଯାଇ ବିକ୍ରି ହୋଇଥିବା ଉତ୍ପାଦନର ପରିମାଣ	If any sold, what income did you make? (no value addition) (Rs.) ଯଦି ବିକ୍ରି ହେଲା ସେଥିରୁ କେତେ ଆୟ କଲେ (ବିନା ମୂଲ୍ୟ ଉପଯୋଗ କରି)(ଟଙ୍କାରେ)	If any value-added products sold, what income did you make from the sale? (Rs.) ଯଦି ମୂଲ୍ୟ ଉପଯୋଗ କରାଯାଇଥିବା ଉତ୍ପାଦନ ବିକ୍ରି ହେଲା ସେଥିରୁ କେତେ ଆୟ କଲେ?(ଟଙ୍କାରେ)
**Kharif/** ଖରିଫ(ବର୍ଷା ଦିନିଆ ଫସଲ)										
☐☐		☐☐☐								
☐☐		☐☐☐								
☐☐		☐☐☐								
☐☐		☐☐☐								
☐☐		☐☐☐								
☐☐		☐☐☐								
☐☐		☐☐☐								
☐☐		☐☐☐								
☐☐		☐☐☐								
☐☐		☐☐☐								
☐☐		☐☐☐								
☐☐		☐☐☐								
☐☐		☐☐☐								

Crop / vegetable code /ଶସ୍ୟ/ପନି ପରିବା କୋଡ	Total yield	Unit/ ଏକକ	HH share of total yield (excluding post harvest losses) ସମସ୍ତ ଉତ୍ପାଦନରୁ ପରିବାରର ଭାଗ (ଅମଳ ପରେ ହୋଇଥିବା କ୍ଷତିକୁ ବାଦଦେଇ)	Quantity consumed or used by household (food or other uses, e.g. fodder)/ ପରିବାରରେ ଖାଇଥିବା କିମ୍ବା ବ୍ୟବହାର ହୋଇଥିବା ସାମଗ୍ରୀର ପରିମାଣ (ଖାଦ୍ୟ କିମ୍ବା ଅନ୍ୟକିଛି ବ୍ୟବହାର)	Quantity stored ଗଚ୍ଛିତ ହୋଇଥିବା ଉତ୍ପାଦନର ପରିମାଣ	Quantity given as gifts or transfers or in-kind payments ଦାନରେ ଦିଆଯାଇଥିବ ବା ପଠାଯାଇଥିବା ର ପରିମାଣ ବା ଜିନିଷ ଆକାରରେ ଦିଆଯାଇଥିବା ପରିମାଣ	Quantity sold (including value added products)/ ବିକ୍ରି ହୋଇଥିବାର ପରିମାଣ (ମୂଲ୍ୟ ଉପଯୋଗ କରାଯାଇଥିବା ଉତ୍ପାଦନକୁ ମିଶାଇ) → *If 0 sold, skip to next crop*	Quantity sold that was value-added, if any/ ମୂଲ୍ୟ ଉପଯୋଗ କରାଯାଇ ବିକ୍ରି ହୋଇଥିବା ଉତ୍ପାଦନର ପରିମାଣ	If any sold, what income did you make? (no value addition) (Rs.) ଯଦି ବିକ୍ରି ହେଲା ସେଥିରୁ କେତେ ଆୟ କଲେ (ବିନା ମୂଲ୍ୟ ଉପଯୋଗ କରି)(ଟଙ୍କାରେ)	If any value-added products sold, what income did you make from the sale? (Rs.) ଯଦି ମୂଲ୍ୟ ଉପଯୋଗ କରାଯାଇଥିବା ଉତ୍ପାଦନ ବିକ୍ରି ହେଲା ସେଥିରୁ କେତେ ଆୟ କଲେ?(ଟଙ୍କାରେ)
**Kharif/** ଖରିଫ(ବର୍ଷା ଦିନିଆ ଫସଲ)										
☐☐		☐☐☐								
☐☐		☐☐☐								

Crop / vegetable code /ଶସ୍ୟ/ପନି ପରିବା କୋଡ	Total yield	Unit/ ଏକକ	HH share of total yield (excluding post harvest losses) ସମସ୍ତ ଉତ୍ପାଦନରୁ ପରିବାରର ଭାଗ (ଅମଳ ପରେ ହୋଇଥିବା କ୍ଷତିକୁ ବାଦଦେଇ)	Quantity consumed or used by household (food or other uses, e.g. fodder)/ ପରିବାରରେ ଖାଇଥିବା କିମ୍ବା ବ୍ୟବହାର ହୋଇଥିବା ସାମଗ୍ରୀର ପରିମାଣ (ଖାଦ୍ୟ କିମ୍ବା ଅନ୍ୟକିଛି ବ୍ୟବହାର)	Quantity stored ଗଚ୍ଛିତ ହୋଇଥିବା ଉତ୍ପାଦନର ପରିମାଣ	Quantity given as gifts or transfers or in-kind payments ଦାନରେ ଦିଆଯାଇଥିବ ବା ପଠାଯାଇଥିବା ର ପରିମାଣ ବା ଜିନିଷ ଆକାରରେ ଦିଆଯାଇଥିବା ପରିମାଣ	Quantity sold (including value-added products)/ ବିକ୍ରି ହୋଇଥିବାର ପରିମାଣ (ମୂଲ୍ୟ ଉପଯୋଗ କରାଯାଇଥିବା ଉତ୍ପାଦନକୁ ମିଶାଇ) → *If 0 sold, skip to next crop*	Quantity sold that was value added, if any/ ମୂଲ୍ୟ ଉପଯୋଗ କରାଯାଇ ବିକ୍ରି ହୋଇଥିବା ଉତ୍ପାଦନର ପରିମାଣ	If any sold, what income did you make? (no value addition) (Rs.) ଯଦି ବିକ୍ରି ହେଲା ସେଥିରୁ କେତେ ଆୟ କଲେ (ବିନା ମୂଲ୍ୟ ଉପଯୋଗ କରି)(ଟଙ୍କାରେ)	If any value-added products sold, what income did you make from the sale? (Rs.) ଯଦି ମୂଲ୍ୟ ଉପଯୋଗ କରାଯାଇଥିବା ଉତ୍ପାଦନ ବିକ୍ରି ହେଲା ସେଥିରୁ କେତେ ଆୟ କଲେ?(ଟଙ୍କାରେ)
**Rabi/** ରବି ଶୀତ ଦିନିଆ ଫସଲ										
☐☐		☐☐☐								
☐☐		☐☐☐								
☐☐		☐☐☐								
☐☐		☐☐☐								

Crop / vegetable code /ଶସ୍ୟ/ପନିପରିବା କୋଡ	Total yield	Unit/ *ଏକକ*	HH share of total yield (excluding post harvest losses) ସମସ୍ତ ଉତ୍ପାଦନରୁ ପରିବାରର ଭାଗ (ଅମଳ ପରେ ହୋଇଥିବା କ୍ଷତିକୁ ବାଦଦେଇ)	Quantity consumed or used by household (food or other uses, e.g. fodder)/ ପରିବାରରେ ଖାଇଥିବା କିମ୍ବା ବ୍ୟବହାର ହୋଇଥିବା ସାମଗ୍ରୀର ପରିମାଣ (ଖାଦ୍ୟ କିମ୍ବା ଅନ୍ୟକିଛି ବ୍ୟବହାର)	Quantity stored ଗଚ୍ଛିତ ହୋଇଥିବା ଉତ୍ପାଦନର ପରିମାଣ	Quantity given as gifts or transfers or in-kind payments ଦାନରେ ଦିଆଯାଇଥିବ ବା ପଠାଯାଇଥିବା ର ପରିମାଣ ବା ଜିନିଷ ଆକାରରେ ଦିଆଯାଇଥିବା ପରିମାଣ	Quantity sold (including value-added products)/ ବିକ୍ରି ହୋଇଥିବାର ପରିମାଣ (ମୂଲ୍ୟ ଉପଯୋଗ କରାଯାଇଥିବା ଉତ୍ପାଦନକୁ ମିଶାଇ) → *If 0 sold, skip to next crop*	Quantity sold that was value added, if any/ ମୂଲ୍ୟ ଉପଯୋଗ କରାଯାଇ ବିକ୍ରି ହୋଇଥିବା ଉତ୍ପାଦନର ପରିମାଣ	If any sold, what income did you make? (no value addition) (Rs.) ଯଦି ବିକ୍ରି ହେଲା ସେଥିରୁ କେତେ ଆୟ କଲେ (ବିନା ମୂଲ୍ୟ ଉପଯୋଗ କରି)(ଟଙ୍କାରେ)	If any value-added products sold, what income did you make from the sale? (Rs.) ଯଦି ମୂଲ୍ୟ ଉପଯୋଗ କରାଯାଇଥିବା ଉତ୍ପାଦନ ବିକ୍ରି ହେଲା ସେଥିରୁ କେତେ ଆୟ କଲେ?(ଟଙ୍କାରେ)
**Rabi/** ରବି ଶୀତ ଦିନିଆ ଫସଲ										
☐☐		☐☐☐								
☐☐		☐☐☐								
☐☐		☐☐☐								
☐☐		☐☐☐								
☐☐		☐☐☐								
☐☐		☐☐☐								
☐☐		☐☐☐								
☐☐		☐☐☐								
☐☐		☐☐☐								
☐☐		☐☐☐								
☐☐		☐☐☐								
☐☐		☐☐☐								
☐☐		☐☐☐								

Crop / vegetable code /ଶସ୍ୟ/ପନିପରିବା କୋଡ	Total yield	Unit/ *ଏକକ*	HH share of total yield (excluding post harvest losses) ସମସ୍ତ ଉତ୍ପାଦନରୁ ପରିବାରର ଭାଗ (ଅମଳ ପରେ ହୋଇଥିବା କ୍ଷତିକୁ ବାଦଦେଇ)	Quantity consumed or used by household (food or other uses, e.g. fodder)/ ପରିବାରରେ ଖାଇଥିବା କିମ୍ବା ବ୍ୟବହାର ହୋଇଥିବା ସାମଗ୍ରୀର ପରିମାଣ (ଖାଦ୍ୟ କିମ୍ବା ଅନ୍ୟକିଛି ବ୍ୟବହାର)	Quantity stored ଗଚ୍ଛିତ ହୋଇଥିବା ଉତ୍ପାଦନର ପରିମାଣ	Quantity given as gifts or transfers or in-kind payments ଦାନରେ ଦିଆଯାଇଥିବ ବା ପଠାଯାଇଥିବା ର ପରିମାଣ ବା ଜିନିଷ ଆକାରରେ ଦିଆଯାଇଥିବା ପରିମାଣ	Quantity sold (including value-added products)/ ବିକ୍ରି ହୋଇଥିବାର ପରିମାଣ (ମୂଲ୍ୟ ଉପଯୋଗ କରାଯାଇଥିବା ଉତ୍ପାଦନକୁ ମିଶାଇ) → *If 0 sold, skip to next crop*	Quantity sold that was value added, if any/ ମୂଲ୍ୟ ଉପଯୋଗ କରାଯାଇ ବିକ୍ରି ହୋଇଥିବା ଉତ୍ପାଦନର ପରିମାଣ	If any sold, what income did you make? (no value addition) (Rs.) ଯଦି ବିକ୍ରି ହେଲା ସେଥିରୁ କେତେ ଆୟ କଲେ (ବିନା ମୂଲ୍ୟ ଉପଯୋଗ କରି)(ଟଙ୍କାରେ)	If any value-added products sold, what income did you make from the sale? (Rs.) ଯଦି ମୂଲ୍ୟ ଉପଯୋଗ କରାଯାଇଥିବା ଉତ୍ପାଦନ ବିକ୍ରି ହେଲା ସେଥିରୁ କେତେ ଆୟ କଲେ?(ଟଙ୍କାରେ)
**Rabi/** ରବି ଶୀତ ଦିନିଆ ଫସଲ										
☐☐		☐☐☐								
☐☐		☐☐☐								
☐☐		☐☐☐								
☐☐		☐☐☐								
☐☐		☐☐☐								
☐☐		☐☐☐								
☐☐		☐☐☐								
☐☐		☐☐☐								
☐☐		☐☐☐								
☐☐		☐☐☐								
☐☐		☐☐☐								
☐☐		☐☐☐								
☐☐		☐☐☐								

Crop / vegetable code /ଶସ୍ୟ/ପନିପରିବା କୋଡ	Total yield	Unit/ *ଏକକ*	HH share of total yield (excluding post harvest losses) ସମସ୍ତ ଉତ୍ପାଦନରୁ ପରିବାରର ଭାଗ (ଅମଳ ପରେ ହୋଇଥିବା କ୍ଷତିକୁ ବାଦଦେଇ)	Quantity consumed or used by household (food or other uses, e.g. fodder)/ ପରିବାରରେ ଖାଇଥିବା କିମ୍ବା ବ୍ୟବହାର ହୋଇଥିବା ସାମଗ୍ରୀର ପରିମାଣ (ଖାଦ୍ୟ କିମ୍ବା ଅନ୍ୟକିଛି ବ୍ୟବହାର)	Quantity stored ଗଚ୍ଛିତ ହୋଇଥିବା ଉତ୍ପାଦନର ପରିମାଣ	Quantity given as gifts or transfers or in-kind payments ଦାନରେ ଦିଆଯାଇଥିବ ବା ପଠାଯାଇଥିବା ର ପରିମାଣ ବା ଜିନିଷ ଆକାରରେ ଦିଆଯାଇଥିବା ପରିମାଣ	Quantity sold (including value-added products)/ ବିକ୍ରି ହୋଇଥିବାର ପରିମାଣ (ମୂଲ୍ୟ ଉପଯୋଗ କରାଯାଇଥିବା ଉତ୍ପାଦନକୁ ମିଶାଇ) → *If 0 sold, skip to next crop*	Quantity sold that was value added, if any/ ମୂଲ୍ୟ ଉପଯୋଗ କରାଯାଇ ବିକ୍ରି ହୋଇଥିବା ଉତ୍ପାଦନର ପରିମାଣ	If any sold, what income did you make? (no value addition) (Rs.) ଯଦି ବିକ୍ରି ହେଲା ସେଥିରୁ କେତେ ଆୟ କଲେ (ବିନା ମୂଲ୍ୟ ଉପଯୋଗ କରି)(ଟଙ୍କାରେ)	If any value-added products sold, what income did you make from the sale? (Rs.) ଯଦି ମୂଲ୍ୟ ଉପଯୋଗ କରାଯାଇଥିବା ଉତ୍ପାଦନ ବିକ୍ରି ହେଲା ସେଥିରୁ କେତେ ଆୟ କଲେ?(ଟଙ୍କାରେ)
**Rabi/** ରବି ଶୀତ ଦିନିଆ ଫସଲ										
☐☐		☐☐☐								
☐☐		☐☐☐								
☐☐		☐☐☐								
☐☐		☐☐☐								
☐☐		☐☐☐								
☐☐		☐☐☐								
☐☐		☐☐☐								
☐☐		☐☐☐								
☐☐		☐☐☐								
☐☐		☐☐☐								
☐☐		☐☐☐								

Crop / vegetable code /ଶସ୍ୟ/ପନିପରିବା କୋଡ	Total yield	Unit/ ଏକକ	HH share of total yield (excluding post harvest losses) ସମସ୍ତ ଉତ୍ପାଦନରୁ ପରିବାରର ଭାଗ (ଅମଳ ପରେ ହୋଇଥିବା କ୍ଷତିକୁ ବାଦଦେଇ)	Quantity consumed or used by household (food or other uses, e.g. fodder)/ ପରିବାରରେ ଖାଇଥିବା କିମ୍ବା ବ୍ୟବହାର ହୋଇଥିବା ସାମଗ୍ରୀର ପରିମାଣ (ଖାଦ୍ୟ କିମ୍ବା ଅନ୍ୟକିଛି ବ୍ୟବହାର)	Quantity stored ଗଚ୍ଛିତ ହୋଇଥିବା ଉତ୍ପାଦନର ପରିମାଣ	Quantity given as gifts or transfers or in-kind payments ଦାନରେ ଦିଆଯାଇଥିବ ବା ପଠାଯାଇଥିବା ର ପରିମାଣ ବା ଜିନିଷ ଆକାରରେ ଦିଆଯାଇଥିବା ପରିମାଣ	Quantity sold (including value-added products)/ ବିକ୍ରି ହୋଇଥିବାର ପରିମାଣ (ମୂଲ୍ୟ ଉପଯୋଗ କରାଯାଇଥିବା ଉତ୍ପାଦନକୁ ମିଶାଇ) → *If 0 sold, skip to next crop*	Quantity sold that was value added, if any/ ମୂଲ୍ୟ ଉପଯୋଗ କରାଯାଇ ବିକ୍ରି ହୋଇଥିବା ଉତ୍ପାଦନର ପରିମାଣ	If any sold, what income did you make? (no value addition) (Rs.) ଯଦି ବିକ୍ରି ହେଲା ସେଥିରୁ କେତେ ଆୟ କଲେ (ବିନା ମୂଲ୍ୟ ଉପଯୋଗ କରି)(ଟଙ୍କାରେ)	If any value-added products sold, what income did you make from the sale? (Rs.) ଯଦି ମୂଲ୍ୟ ଉପଯୋଗ କରାଯାଇଥିବା ଉତ୍ପାଦନ ବିକ୍ରି ହେଲା ସେଥିରୁ କେତେ ଆୟ କଲେ?(ଟଙ୍କାରେ)
**Summer/**ଗ୍ରୀଷ୍ମକାଳୀନ/ଖରା ଦିନିଆ ଫସଲ										
☐☐		☐☐☐								
☐☐		☐☐☐								
☐☐		☐☐☐								
☐☐		☐☐☐								
☐☐		☐☐☐								
☐☐		☐☐☐								
☐☐		☐☐☐								
☐☐		☐☐☐								
☐☐		☐☐☐								
☐☐		☐☐☐								
☐☐		☐☐☐								
☐☐		☐☐☐								
☐☐		☐☐☐								

Crop / vegetable code /ଶସ୍ୟ/ପନିପରିବା କୋଡ	Total yield	Unit/ ଏକକ	HH share of total yield (excluding post harvest losses) ସମସ୍ତ ଉତ୍ପାଦନରୁ ପରିବାରର ଭାଗ (ଅମଳ ପରେ ହୋଇଥିବା କ୍ଷତିକୁ ବାଦଦେଇ)	Quantity consumed or used by household (food or other uses, e.g. fodder)/ ପରିବାରରେ ଖାଇଥିବା କିମ୍ବା ବ୍ୟବହାର ହୋଇଥିବା ସାମଗ୍ରୀର ପରିମାଣ (ଖାଦ୍ୟ କିମ୍ବା ଅନ୍ୟକିଛି ବ୍ୟବହାର)	Quantity stored ଗଚ୍ଛିତ ହୋଇଥିବା ଉତ୍ପାଦନର ପରିମାଣ	Quantity given as gifts or transfers or in-kind payments ଦାନରେ ଦିଆଯାଇଥିବ ବା ପଠାଯାଇଥିବା ର ପରିମାଣ ବା ଜିନିଷ ଆକାରରେ ଦିଆଯାଇଥିବା ପରିମାଣ	Quantity sold (including value-added products)/ ବିକ୍ରି ହୋଇଥିବାର ପରିମାଣ (ମୂଲ୍ୟ ଉପଯୋଗ କରାଯାଇଥିବା ଉତ୍ପାଦନକୁ ମିଶାଇ) → *If 0 sold, skip to next crop*	Quantity sold that was value added, if any/ ମୂଲ୍ୟ ଉପଯୋଗ କରାଯାଇ ବିକ୍ରି ହୋଇଥିବା ଉତ୍ପାଦନର ପରିମାଣ	If any sold, what income did you make? (no value addition) (Rs.) ଯଦି ବିକ୍ରି ହେଲା ସେଥିରୁ କେତେ ଆୟ କଲେ (ବିନା ମୂଲ୍ୟ ଉପଯୋଗ କରି)(ଟଙ୍କାରେ)	If any value-added products sold, what income did you make from the sale? (Rs.) ଯଦି ମୂଲ୍ୟ ଉପଯୋଗ କରାଯାଇଥିବା ଉତ୍ପାଦନ ବିକ୍ରି ହେଲା ସେଥିରୁ କେତେ ଆୟ କଲେ?(ଟଙ୍କାରେ)
**Summer/**ଗ୍ରୀଷ୍ମକାଳୀନ/ଖରା ଦିନିଆ ଫସଲ										
☐☐		☐☐☐								
☐☐		☐☐☐								
☐☐		☐☐☐								
☐☐		☐☐☐								
☐☐		☐☐☐								
☐☐		☐☐☐								
☐☐		☐☐☐								
☐☐		☐☐☐								
☐☐		☐☐☐								
☐☐		☐☐☐								
☐☐		☐☐☐								
☐☐		☐☐☐								
☐☐		☐☐☐								

Crop code/ଶସ୍ୟ କୋଡ:

CEREALS<br>ଶସ୍ୟ	PULSES<br>ଡାଲି ଜାତୀୟ	NUTS / SEEDS / SPICES/<br>ବାଦାମ/ମଞ୍ଜି/ମସଲା ଜାତୀୟ	CASH CROP<br>ଅର୍ଥକାରୀ ଫସଲ	ROOTS/ TUBERS<br>ମୂଳ ଜାତୀୟ	GREEN LEAVES<br>ଶାଗ
Rice/ଚାଉଳ=1,<br>Paddy/ଧାନ=2,<br>Sorghum/ବଜାର ଜାତୀୟ=3,<br>Millet/ବାଜରା=4,<br>Maize/ମକା=5,<br>Ragi/ମଣ୍ଡିଆ=6,<br>Wheat/ଗହମ=7,<br>Barley/ବାର୍ଲି = 8,<br><br>Other cereal/ଅନ୍ୟାନ୍ୟ ସେରେଲା = 98	Black Gram/ବିରି=9,<br>Green Gram/ମୁଗ=10,<br>Red Gram/ହରଡ=11,<br>Lentil/ମସୁର=12,<br><br>Other gram/ଅନ୍ୟାନ୍ୟ ଡାଲି=13,	Sesame/ତିଳ=14,<br>Mustard/ Rape seed/ସୋରିଷ=15,<br>Linseed/ଅଳସୀ=16,<br>Caster seed/ଜଡା=17,<br>Sunflower/ସୂର୍ଯ୍ୟମୁଖୀ=18,<br>Turmeric/ହଳଦୀ=20,<br>Cashew/କାଜୁ=21,<br>Groundnut/ଚୀନାବାଦାମ=22,<br><br>Other oil seeds/ଅନ୍ୟାନ୍ୟ ତୈଳବୀଜ=19,<br>Other seed, nut or spice/ଅନ୍ୟାନ୍ୟ ମଞ୍ଜି,ବାଦାମ ବା ମସଲା = 99	Cotton/କପା=23,<br>Jute/ଝୋଟ=24,<br>Other non-edible fibres/ଅନ୍ୟାନ୍ୟ ଅଣ-ଖାଦ୍ୟ ତନ୍ତୁ=25,<br>Tobacco/ନିଶା ଜାତୀୟ ଦ୍ରବ୍ୟ=26,<br>Sugarcane/ଆଖୁ=27,<br>Ginger/ଅଦା=28,<br><br>Other cash crop/ଅନ୍ୟାନ୍ୟ ଅର୍ଥକାରୀ ଫସଲ = 97	Potato/ଆଳୁ=29,<br>Sweet potato/କନ୍ଦମୂଳ=30,<br>Yam/ମାଟି ଆଳୁ=31,<br>Colocassia Roots=32,<br>Elephant foot (Ol)/ଖମ୍ବଆଳୁ=33,<br>Turnip/ଓଲ କୋବି=34,<br>Carrot/ଗାଜର=35,<br>Radish (Mula)/ମୂଳା=36,<br>Kohirabi/German Turnip/ସାଲଗମ=37,<br>Beet/ବିଟ=38,<br><br>Other white tuber = 96,<br>Other non-white tuber = 96	Coriander/ଧନିଆ ପତ୍ର=72,<br>Mint Leaves/ପୋଦିନା ପତ୍ର=73,<br>Spinach/ପାଳଙ୍ଗ=74,<br>Curry Leaves/ଭୃର୍ସୁଙ୍ଗା ପତ୍ର=75,<br>Indian Spinach/ପୋଇ=76,<br>Green Amarnath/ଖଡା ଶାଗ=77,<br>Fenugreek leaves/ମେଥି ପତ୍ର=78,<br>Spinage/ପୋଇ=79,<br><br>Other green leafy vegetable/ଅନ୍ୟାନ୍ୟ ସବୁଜ ପନିପରିବା = 94

OTHER VEG<br>ଅନ୍ୟାନ୍ୟ ପନିପରିବା	
Chillies/ଲଙ୍କା=39,<br>Aubergine (Brinjal)/ବାଇଗଣ=40,<br>Cauliflower/ଫୁଲକୋବି=41,<br>Onion/ପିଆଜ=42,<br>Garlic/ରସୁଣ=43,<br>Tomato/ଟମାଟୋ=44, Mushroom/ଛତୁ=45,<br>Okra (Bhindi)/ଭେଣ୍ଡି=46,<br>Pumpkin/କଖାରୁ=47,<br>Courgette/ zucchini (turee/ phuti kakudi)/ଫୁଟି କାକୁଡି=48, Turnip (Ola Kobi or Salgum)=49,<br>Bottle gourd (Laou)=50<br>Parbal / Pointed gourd/ପୋଟଳ=51,<br>Peer Gourd (Kankad)/କାଙ୍କଡ=52,<br>Sponge gourd/ଜହ୍ନି=53,<br>Bitter gourd/କଲରା=54,<br>Snake gourd/ଛଚିନ୍ଦ୍ରା=55,<br>Ivy Gourd /Kunduri/କୁନ୍ଦୁରୀ=56,	Ridge Gourd/ଜହ୍ନି=57,<br>Ash Gourd/ପାଣି କଖାରୁ=58,<br>Other gourd/ଅନ୍ୟାନ୍ୟ=59,<br>Green beans/ବିନସ=60,<br>Capsicum/କ୍ୟାପସିକମ=61,<br>Cucumber/କାକୁଡି=62,<br>Cabbage/ବନ୍ଧାକୋବି=63,<br>Green peas/ମଟର=64,<br>Jhudang/ଝୁଡଙ୍ଗ=65,<br>Arum (Saru)/ସାରୁ=66,<br>Jackfruit/ପଣସ=67, Drumstick/ସଜନାଛୁଇଁ=68,<br>Cluster beans/ଗୁଆଁର ଛୁଇଁ=69,<br>Yardlong bean/ବରଗୁଡି ଛୁଇଁ=70,<br>Butter beans/ଶିମ୍ବ=71,<br>Other vegetable/ଅନ୍ୟାନ୍ୟ ପନିପରିବା = 95

13. Livestock holding and income during last one year (June 2015 to May 2016)

ଗତ ବର୍ଷ ଜୁନ ୨୦୧୫ ରୁ ମେ ୨୦୧୬ ମଧ୍ୟରେ ପଶୁସମ୍ପଦ ଲାଳନ ପାଳନ ଓ ଆୟ

Variable name	Question	Tick all that apply	Variable	Question	Answer
lstock	13.1<br><br>In the past 3 agricultural seasons, did you own any of the following (even if you don't currently own any)? (select all that apply)/<br><br>ଗତ ୩ଟି କୃଷି ଋତୁରେ ଆପଣଙ୍କର କୌଣସି ପଶୁସମ୍ପଦ ଥିଲା କି ?(ବର୍ତ୍ତମାନ କୌଣସି ପଶୁ ନଥିଲେ ମଧ୍ୟ)	☐ None/କିଛିନାହିଁ = Y<br>→ *go to 13.2*			
		☐ Bullocks/ବଳଦ = A<br>→ *go to 13.1.1*	13.1.1.<br>lstock_bull_n	If yes, how many do you currently own?<br><br>ଯଦି ହଁ, ବର୍ତ୍ତମାନ କେତୋଟି ଲେଖାଏଁ ଅଛନ୍ତି ?	☐
		☐ Cows/ଗାଈ = B<br>→ *go to 13.1.2*	13.1.2.<br>lstock_cow_n		☐
		☐ Buffalo/ମଇଁଷି = C<br>→ *go to 13.1.3*	13.1.3.<br>lstock_buff_n		☐
		☐ Calves (cow)/ଗାଈବାଛୁରି = D<br>→ *go to 13.1.4*	13.1.4.<br>lstock_calves_n		☐
		☐ Goats/ଛେଳି = E<br>→ *go to 13.1.5*	13.1.5.<br>lstock_goats_n		☐
		☐ Sheep/ମେଣ୍ଢା = F<br>→ *go to 13.1.6*	13.1.6.<br>lstock_sheep_n		☐
		☐ Chicken/କୁକୁଡା = G<br>→ *go to 13.1.7*	13.1.7.<br>lstock_chick_n		☐
		☐ Duck/ବତକ = H<br>→ *go to 13.1.8*	13.1.8.<br>lstock_duck_n		☐
		☐ Pig/ଘୁଷୁରି = I<br>→ *go to 13.1.9*	13.1.9.<br>lstock_pig_n		☐
		☐ Pigeons / swans/ପାରା/ହଂସ = J<br>→ *go to 13.1.10*	13.1.10.<br>lstock_pigeon_n		☐
		☐ Rabbit/ଠେକୁଆ = K<br>→ *go to 13.1.11*	13.1.11.<br>lstock_rabbit_n		☐
		☐ Beehives/ମହୁଫେଣା = L<br>→ *go to 13.1.12*	13.1.12.<br>lstock_bees_n		☐
		☐ Other/ଅନ୍ୟାନ୍ୟ = X<br>→ *go to 13.1.13*	13.1.13.<br>lstock_ot_n		☐

13.2. Meat/ମାଂସ

Did you produce any <animal name> meat in the past year? (June 2015 to May 2016, whether for consumption or sale)<br>ଗତ ଜୁନ, ୨୦୧୫ ଠାରୁ ମେ, ୨୦୧୬ ମଧ୍ୟରେ ଘରେ ଖାଇବା ପାଇଁ ବା ବିକ୍ରି କରିବା ଉଦ୍ଦେଶ୍ୟରେ ମାଂସ ଉତ୍ପାଦନ କରିଥିଲେ କି ?<br>**Yes/ହଁ---------- 1**<br>**No /ନା---------- 0**<br>→ *If 0, skip to next animal*		If yes, what quantity was produced?<br>ଯଦି ହଁ,କେତେ ପରିମାଣର ଉତ୍ପାଦନ କରିଥିଲେ ?	**Unit/**ଏକକ<br>**Kg/**କି.ଗ୍ରା**------1**<br>**Unit item/** ଏକକ ଦ୍ରବ୍ୟ**-----------4**	If any produced: How much did your household consume?<br>ଯଦି ଉତ୍ପାଦନ ହୋଇଥାଏ କେତେ ପରିମାଣ ଘରେ ଖାଇଥିଲେ?	If any produced: How much did you sell?<br>ଯଦି ଉତ୍ପାଦନ ହୋଇଥାଏକେତେ ପରିମାଣ ବିକ୍ରି କରିଥିଲେ ?<br>→ *If 0 sold, skip to next animal*	If any sold: Did you add value to the product before selling?<br>ଯଦି ବିକ୍ରି କରିଥିଲେ ବିକ୍ରି ପୂର୍ବରୁ ସେଥିରେ ମୂଲ୍ୟ ଯୁକ୍ତ କରିଥିଲେ କି ?<br>**Yes/ହଁ-- 1**<br>**No /ନା—0**	If any sold: What income did you make from sale (including value addition) (Rs.)?<br>ସେଥିରୁ କେତେ ଆୟ ହେଲା ? (ମୂଲ୍ୟ ଉପଯୋଗ କରାଯାଇଛି)
13.2.1 Buffalo/ ମଇଁଷି	☐		☐			☐	
13.2.2 Goat/ ଛେଳି	☐		☐			☐	
13.2.3 Sheep/ ମେଣ୍ଢା	☐		☐			☐	
13.2.4 Chicken/ କୁକୁଡା	☐		☐			☐	
13.2.5 Duck/ ବତକ	☐		☐			☐	
13.2.6 Pig/ ଘୁଷୁରୀ	☐		☐			☐	
13.2.7 Pigeon/ ପାରା	☐		☐			☐	
13.2.8 Rabbit/ ଠେକୁଆ	☐		☐			☐	

13.3. Eggs/ଅଣ୍ଡା

Did you produce any eggs in the past 3 agricultural seasons? (June 2015 to May 2016, whether for consumption or sale)<br>ଗତ ଜୁନ, ୨୦୧୫ ଠାରୁ ମେ, ୨୦୧୬ ମଧ୍ୟରେ ଘରେ ଖାଇବା ପାଇଁ ବା ବିକ୍ରି କରିବା ଉଦ୍ଦେଶ୍ୟରେ ଅଣ୍ଡାଉତ୍ପାଦନ କରିଥିଲେ କି ?<br>**Yes/ହଁ---------- 1**<br>**No /ନା---------- 0**<br>→ *If 0, skip to 13.4*	In the last year, in how many months did you produce any eggs?<br>ଗତ ଏକବର୍ଷ ମଧ୍ୟରେ ଆପଣ କେତେ ମାସ ଅଣ୍ଡା ଉତ୍ପାଦନ କରିଥିଲେ ?	In each of those months, how many eggs did you get on average?<br>ସେହି ସବୁ ମାସରେ ହାରାହାରି କେତୋଟି ଅଣ୍ଡା ଉତ୍ପାଦନ ହୋଇଥିଲା ?	Unit/ ଏକକ<br>**Unit item/**ଏକକ ଦ୍ରବ୍ୟ------------**1**<br>**Dozen/**ଡଜନ-----------------**2**<br>**Tray of 30 eggs /** ଗୋଟେ ଟ୍ରେ ରେ ୩୦ଟି ଅଣ୍ଡା**-------3**	In each of those months, how many eggs did you eat on average?<br>ଯଦି ଉତ୍ପାଦନ କରୁଛନ୍ତି ସେହି ସବୁ ମାସରେ ଆପଣ ହାରାହାରି କେତୋଟି ଅଣ୍ଡା ଖାଇଛନ୍ତି ?	In each of those months, how many eggs did you sell on average?<br>ଯଦି ଉତ୍ପାଦନ କରୁଛନ୍ତି ସେହି ସବୁ ମାସରେ ଆପଣ ହାରାହାରି କେତୋଟି ଅଣ୍ଡା ବିକ୍ରୀ କରିଛନ୍ତି ?<br>→ *If 0, skip to 13.4*	In each of those months, what income did you make from sale of eggs on average (Rs.)?<br>ଯଦି ଉତ୍ପାଦନ କରୁଛନ୍ତି ସେହି ସବୁ ମାସରେ ଅଣ୍ଡା ବିକ୍ରୀକରି ଆପଣ ହାରାହାରି କେତେ ଆୟ କରିଛନ୍ତି (ଟଙ୍କାରେ) ?
13.3.1 **Eggs /**ଅଣ୍ଡା						

13.4. Milk/କ୍ଷୀର

Did you produce any <item name>in the past year? (June 2015 to May 2016, whether for consumption or sale)<br>ଗତ ଜୁନ, ୨୦୧୫ ଠାରୁ ମେ, ୨୦୧୬ ମଧ୍ୟରେ ଘରେ ଖାଇବା ପାଇଁ ବା ବିକ୍ରି କରିବା ଉଦ୍ଦେଶ୍ୟରେ ଦୁଗ୍ଧଜାତ ଦ୍ରବ୍ୟ ଉତ୍ପାଦନ କରିଥିଲେ କି ?<br>**Yes/ହଁ--------- 1**<br>**No /ନା--------- 0**<br>→ *If 0, skip to next milk product*	Last year, in how many months did you produce any <item name>?<br>ଗତ ଏକବର୍ଷ ମଧ୍ୟରେ ଆପଣ କେତେ ମାସ ଦୁଗ୍ଧଜାତ ଦ୍ରବ୍ୟ ଉତ୍ପାଦନ କରିଥିଲେ ?	In those months, how much did your household produce on average?<br>ସେହି ସବୁ ମାସରେ ଆପଣଙ୍କ ପରିବାରର ହାରାହାରି କେତେ ଉତ୍ପାଦନ ହୋଇଥିଲା ?	**Unit/**ଏକକ	In those months, how much did your household consume on average?<br>ଯଦି ଉତ୍ପାଦନ ହୋଇଥିଲା ସେହି ସବୁ ମାସରେ ଆପଣ ପରିବାରରେ ହାରାହାରି କେତେ ପରିମାଣ ଖାଇଥିଲେ?	In those months, how much did you sell on average?<br>ଯଦି ଉତ୍ପାଦନ ହୋଇଥିଲା ସେହି ସବୁ ମାସରେ ଆପଣ ହାରାହାରି କେତେ ପରିମାଣ ବିକ୍ରୀ କରିଛନ୍ତି ?<br>→ *If 0, skip to next milk product*	In those months, what income did you make from sale on average (Rs.)? ଯଦି ଉତ୍ପାଦନ ହୋଇଥିଲା ସେହି ସବୁ ମାସରେ ବିକ୍ରୀକରି ଆପଣ ହାରାହାରି କେତେ ଆୟ କରିଛନ୍ତି (ଟଙ୍କାରେ) ?
13.4.1 Milk/କ୍ଷୀର						
13.4.2 Curd/ଦହି						
13.4.3 Chhaas/ (watery curd)/ ଘୋଳଦହି						
13.4.4 Paneer/ଛେନା						

13.4.5 Cream/ଲହୁଣୀ							
13.4.6 Condensed milk/ରାବିଡି							
13.4.7 Milky sweets/ମିଠା/ସନ୍ଦେଶ							
13.4.8 Other (specify)/ଅନ୍ୟାନ୍ୟ (ଦର୍ଶାଅ)							

13.5. Animal on rent or sale/ପଶୁସମ୍ପଦ ଭଡାରେ ଦେବା କିମ୍ବା ବିକ୍ରି କରିବା

	Did you generate any income from renting out or selling <animal name>in the past year? (June 2015 to May 2016)<br>ଗତ ଜୁନ, ୨୦୧୫ ଠାରୁ ମେ, ୨୦୧୬ ମଧ୍ୟରେ ପଶୁସମ୍ପଦକୁ ଭଡାରେ ଦେଇ ବା ବିକ୍ରି କରି କିଛି ରୋଜଗାର କରିଛନ୍ତି କି ?<br><br>Yes/ହଁ----------------------------- 1<br>No/ନା----------------------------- 0<br>→ *If 0, skip to next animal*	If yes, how much did you earn (Rs.)?<br><br>ଯଦି ହଁ କେତେ ରୋଜଗାର କରିଛନ୍ତି (ଟଙ୍କାରେ) ?
13.5.1. Bullocks/ବଳଦ		
13.5.2. Cows/ଗାଈ		
13.5.3. Buffalo/ମହିଷୀ		
13.5.4. Calves/ବାଛୁରି		
13.5.5. Goats/ଛେଳି		
13.5.6. Sheep/ମେଣ୍ଢା		
13.5.7. Chicken /କୁକୁଡା		
Duck/ବତକ		
Pig/ଘୁସୁରୀ		

Pigeons/ପାରା	☐	☐
Rabbit/ଠେକୁଆ	☐	☐

13.6. Fish / honey/ ମାଛ / ମହୁ

13.6.1.<br>Did you catch any fish, prawns, crabs, or other shellfishin the past 3 agricultural seasons? (June 2015 to May 2016, whether for consumption or sale)<br>ଗତ ଜୁନ, ୨୦୧୫ ଠାରୁ ମେ, ୨୦୧୬ ମଧ୍ୟରେ ଘରେ ବ୍ୟବହାର ପାଇଁ ବା ବିକ୍ରି ପାଇଁ କିଛି ମାଛ, ଚିଙ୍ଗୁଡି, କଙ୍କଡା କିମ୍ବା ଅନ୍ୟ କୌଣସି ସାମୁଦ୍ରିକ ମାଛ ଧରିଥିଲେ କି ?<br>**Yes/ହଁ-------------- 1**<br>**No/ନା-------------- 0**<br>→ *If 0, skip to 13.6.2*	How much fish prawns, crabs, or other shellfish did your household catch (kg)?<br>ଯଦି ହଁ, ଆପଣଙ୍କ ପରିବାର କେତେ ମାଛ, ଚିଙ୍ଗୁଡି, କଙ୍କଡା କିମ୍ବା ଅନ୍ୟ କୌଣସି ସାମୁଦ୍ରିକ ମାଛ ଧରିଥିଲେ (କି.ଗ୍ରାରେ) ?	How much did your household consume (kg)?<br>ଯଦି ହଁ, ଆପଣଙ୍କ ପରିବାରରେ କେତେ ମାଛ ଖାଇଥିଲେ (କି.ଗ୍ରାରେ) ?	How much did you sell (kg)?<br>ଯଦି ହଁ କେତେ ବିକ୍ରି କରିଥିଲେ(କି.ଗ୍ରାରେ)?<br>→ *If 0, skip to 13.6.2*	What income did you make from sale (including value addition) (Rs.)<br>ଯଦି କୌଣସି ବିକ୍ରି କାରାଯାଇଥିଲା ସେଥିରୁ କେତେ ଆୟ କରିଥିଲେ (ଟଙ୍କାରେ) ?(ମୂଲ୍ୟ ଉପାଯୋଗ କୁ ମିଶାଇ)	Did you add value to the product before selling?<br>ବିକ୍ରି ପୂର୍ବରୁ ସେଥିରେ କିଛି ମୂଲ୍ୟ ଯୋଗ କରିଥିଲେ କି ?<br>Yes/ହଁ------ 1<br>No/ନା------ 0
☐	☐	☐	☐	☐	☐

13.6.2<br>Did you produce / gather any honey in the past year?<br>ଗଲା ବର୍ଷ ଆପଣ କିଛିୟ ମହୁ ସଂଗ୍ରହ କରିଥିଲେ କି ?<br>**Yes, produced from own beehive/ ହଁ, ନିଜ ମହୁଫେଣାରୁ ---2**<br>**Yes, gathered honey from wild/ ହଁ, ଜଙ୍ଗଲରୁ------------------------ 1**<br>**No/ନା------------------------------ 0**<br>→ *If 0, skip to 14*	How much honey did your household produce or gather (kg)?<br>ଯଦି ହଁ, ଆପଣଙ୍କ ପରିବାର କେତେ ମହୁ ସଂଗ୍ରହ କରିଥିଲେ (କି.ଗ୍ରାରେ)?	How much did your household consume (kg)?<br>ଯଦି ହଁ,ସେଥିରୁ ଆପଣଙ୍କ ଘରେ କେତେ ମହୁ ଖାଇଥିଲେ (କି.ଗ୍ରାରେ)?	How much did you sell (kg)?<br>ଯଦି ହଁ, କେତେ ମହୁ ବିକ୍ରି କରିଛନ୍ତି (କି.ଗ୍ରାରେ)?	What income did you make from sale (including value addition) (Rs.)<br>ଯଦି ବିକ୍ରି ହୋଇଥିଲା ସେଥିରୁ କେତେ ଆୟ କରିଥିଲେ (ଟଙ୍କାରେ) ?(ମୂଲ୍ୟ ଉପଯୋଗ କରାଯାଇ)	Did you add value to the product before selling?<br>ଯଦି ବିକ୍ରି ହୋଇଥିଲା ବିକ୍ରି ପୂର୍ବରୁ ସେଥିରେ କିଛି ମୂଲ୍ୟ ଯୋଗ କରିଥିଲେ କି ?<br>Yes/ହଁ--- 1<br>No/ନା---- 0
☐	☐	☐	☐	☐	☐

14. Fruit and edible Non-Timber Forest Produces (NTFPs)/ଜଙ୍ଗଲଜାତଖାଇବାଦ୍ରବ୍ୟ

Did you grow or gather any fruit or any other edible products from the wild, in the past 3 agricultural seasons? (June 2015 to May 2016, whether for consumption or sale)? *Select from list using free recall* ପରିବାରରେ ଉପଯୋଗ ବା ବିକ୍ରି ଉଦ୍ଦେଶରେ ହେଉ, ଗତ ତିନୋଟି କୃଷି ଋତୁ ମଧ୍ୟରେ ଆପଣ କୌଣସି ଜଙ୍ଗଲ ଜାତ ଫଳମୂଳ ଓ ଖାଇବା ଦ୍ରବ୍ୟ ଉତ୍ପାଦନ କରିଥିଲେ ବା ଜଙ୍ଗଲରୁ ସଂଗ୍ରହ କରିଥିଲେ କି? (ଜୁନ, ୨୦୧୫ ଠାରୁ ମେ, ୨୦୧୬)	**Yes/ହଁ--- 1** **No/ନା--- 0**	Quantity collected or grown/ ସଂଗ୍ରହ କରିଥିବା କିମ୍ବା ବଢାଇଥିବା ଦ୍ରବ୍ୟର ପରିମାଣ	Unit/ଏକକ	Was it gathered / foraged from wild, or produced at home? ଏହାକୁ ଆପଣ ଜଙ୍ଗଲରୁ ଆଣିଥିଲେ ନା ଘରେ ଉତ୍ପାଦନ କରିଥିଲେ ? **Gathered/ସଂଗ୍ରହ କରିଥିଲେ -----------0** **Produced/ଉତ୍ପାଦନ କରିଥିଲେ ------------ 1** **Gathered and produced/ସଂଗ୍ରହ ଏବଂ ଉତ୍ପାଦନ କରିଥିଲେ ---2**	Did you add value to the product? ଆପଣ ଏଥିରେ ମୂଲ୍ୟ ଯୁକ୍ତ କରିଥିଲେ କି ? **Yes/ହଁ---1** **No/ନା---0**	Quantity consumed by household ଘରେ ହୋଇଥିବା ଉପଯୋଗର ପରିମାଣ	Quantity sold including value added ମୂଲ୍ୟ ଯୁକ୍ତକରି ବିକ୍ରିର ପରିମାଣ	What was the income from sale, including any value addition? (Rs.) ସମସ୍ତ ମୂଲ୍ୟ ଯୋଗକରି ବିକ୍ରୀରୁ କେତେ ଟଙ୍କା ଆୟ ହୋଇଥିଲା (ଟଙ୍କାରେ)
Pomegranate/ଡାଳିମ୍ବ	☐	☐	☐☐☐	☐	☐	☐	☐	☐
Bael fruit/ବେଲ	☐	☐	☐☐☐	☐	☐	☐	☐	☐
Chikku / Sapota / Sapeta/ସପେଟା	☐	☐	☐☐☐	☐	☐	☐	☐	☐
Watermelon/ତରଭୁଜ	☐	☐	☐☐☐	☐	☐	☐	☐	☐
Pineapple/ସପୁରୀ	☐	☐	☐☐☐	☐	☐	☐	☐	☐
Banana/କଦଳୀ	☐	☐	☐☐☐	☐	☐	☐	☐	☐
Lemon/ଲେମ୍ବୁ	☐	☐	☐☐☐	☐	☐	☐	☐	☐

Did you grow or gather any fruit or any other edible products from the wild, in the past 3 agricultural seasons? (June 2015 to May 2016, whether for consumption or sale)? *Select from list using free recall*<br>ପରିବାରରେ ଉପଯୋଗ ବା ବିକ୍ରି ଉଦ୍ୟେଶରେ ହେଉ,<br>ଗତ ତିନୋଟି କୃଷି ଋତୁ ମଧ୍ୟରେ ଆପଣ କୌଣସି ଜଙ୍ଗଲ ଜାତ ଫଳମୂଳ ଓ ଖାଇବା ଦ୍ରବ୍ୟ ଉତ୍ପାଦନ କରିଥିଲେ ବା ଜଙ୍ଗଲରୁ ସଂଗ୍ରହ କରିଥିଲେ କି?<br>(ଜୁନ, ୨୦୧୫ ଠାରୁ ମେ, ୨୦୧୬)	**Yes/ହଁ--- 1**<br>**No/ନା--- 0**	Quantity collected or grown/ ସଂଗ୍ରହ କରିଥିବା କିମ୍ବା ବଢାଇଥିବା ଦ୍ରବ୍ୟର ପରିମାଣ	Unit/ଏକକ	Was it gathered / foraged from wild, or produced at home?<br>ଏହାକୁ ଆପଣ ଜଙ୍ଗଲରୁ ଆଣିଥିଲେ ନା ଘରେ ଉତ୍ପାଦନ କରିଥିଲେ ?<br>**Gathered/**ସଂଗ୍ରହ କରିଥିଲେ **-----------0**<br>**Produced/**ଉତ୍ପାଦନ କରିଥିଲେ **------------ 1**<br>**Gathered and produced/**ସଂଗ୍ରହ ଏବଂ ଉତ୍ପାଦନ କରିଥିଲେ **---2**	Did you add value to the product?<br>ଆପଣ ଏଥିରେ ମୂଲ୍ୟ ଯୁକ୍ତ କରିଥିଲେ କି ?<br>**Yes/ହଁ---1**<br>**No/ନା---0**	Quantity consumed by household<br>ଘରେ ହୋଇଥିବା ଉପଯୋଗର ପରିମାଣ	Quantity sold including value added<br>ମୂଲ୍ୟ ଯୁକ୍ତକରି ବିକ୍ରିର ପରିମାଣ	What was the income from sale, including any value addition? (Rs.)<br>ସମସ୍ତ ମୂଲ୍ୟ ଯୋଗକରି ବିକ୍ରୀରୁ କେତେ ଟଙ୍କା ଆୟ ହୋଇଥିଲା (ଟଙ୍କାରେ)
Mushrooms/ଛତୁ	☐	☐	☐☐☐	☐	☐	☐	☐	☐
Amla/ଅଁଳା	☐	☐	☐☐☐	☐	☐	☐	☐	☐
Jamu/ଜାମୁ	☐	☐	☐☐☐	☐	☐	☐	☐	☐
Huckleberry/ବଣକୋଳି	☐	☐	☐☐☐	☐	☐	☐	☐	☐
Mango/ଆମ୍ବ	☐	☐	☐☐☐	☐	☐	☐	☐	☐
Citrus/କମଳା	☐	☐	☐☐☐	☐	☐	☐	☐	☐
Papaya/ଅମୃତଭଣ୍ଡା	☐	☐	☐☐☐	☐	☐	☐	☐	☐

Did you grow or gather any fruit or any other edible products from the wild, in the past 3 agricultural seasons? (June 2015 to May 2016, whether for consumption or sale)? *Select from list using free recall*<br>ପରିବାରରେ ଉପଯୋଗ ବା ବିକ୍ରି ଉଦ୍ୟେଶରେ ହେଉ,<br>ଗତ ତିନୋଟି କୃଷି ଋତୁ ମଧ୍ୟରେ ଆପଣ କୌଣସି ଜଙ୍ଗଲ ଜାତ ଫଳମୂଳ ଓ ଖାଇବା ଦ୍ରବ୍ୟ ଉତ୍ପାଦନ କରିଥିଲେ ବା ଜଙ୍ଗଲରୁ ସଂଗ୍ରହ କରିଥିଲେ କି?<br>(ଜୁନ, ୨୦୧୫ ଠାରୁ ମେ, ୨୦୧୬)	**Yes/ହଁ--- 1**<br>**No/ନା--- 0**	Quantity collected or grown/ ସଂଗ୍ରହ କରିଥିବା କିମ୍ବା ବଢାଇଥିବା ଦ୍ରବ୍ୟର ପରିମାଣ	Unit/ଏକକ	Was it gathered / foraged from wild, or produced at home?<br>ଏହାକୁ ଆପଣ ଜଙ୍ଗଲରୁ ଆଣିଥିଲେ ନା ଘରେ ଉତ୍ପାଦନ କରିଥିଲେ ?<br>**Gathered/ସଂଗ୍ରହ କରିଥିଲେ -----------0**<br>**Produced/ଉତ୍ପାଦନ କରିଥିଲେ ------------ 1**<br>**Gathered and produced/ସଂଗ୍ରହ ଏବଂ ଉତ୍ପାଦନ କରିଥିଲେ ---2**	Did you add value to the product?<br>ଆପଣ ଏଥିରେ ମୂଲ୍ୟ ଯୁକ୍ତ କରିଥିଲେ କି ?<br>**Yes/ହଁ---1**<br>**No/ନା---0**	Quantity consumed by household<br>ଘରେ ହୋଇଥିବା ଉପଯୋଗର ପରିମାଣ	Quantity sold including value added<br>ମୂଲ୍ୟ ଯୁକ୍ତକରି ବିକ୍ରିର ପରିମାଣ	What was the income from sale, including any value addition? (Rs.)<br>ସମସ୍ତ ମୂଲ୍ୟ ଯୋଗକରି ବିକ୍ରୀରୁ କେତେ ଟଙ୍କା ଆୟ ହୋଇଥିଲା (ଟଙ୍କାରେ)
Starfruit/କରମଙ୍ଗା								
Jackfruit/ପଣସ								
Palm/ତାଳ								
Mahula/ମହୁଲ								
Coconut/ନଡିଆ								
Date/ଖଜୁରୀ								
Guava/ପିଜୁଳି								

Did you grow or gather any fruit or any other edible products from the wild, in the past 3 agricultural seasons? (June 2015 to May 2016, whether for consumption or sale)? *Select from list using free recall*<br>ପରିବାରରେ ଉପଯୋଗ ବା ବିକ୍ରି ଉଦ୍ଦେଶ୍ୟରେ ହେଉ,<br>ଗତ ତିନୋଟି କୃଷି ଋତୁ ମଧ୍ୟରେ ଆପଣ କୌଣସି ଜଙ୍ଗଲ ଜାତ ଫଳମୂଳ ଓ ଖାଇବା ଦ୍ରବ୍ୟ ଉତ୍ପାଦନ କରିଥିଲେ ବା ଜଙ୍ଗଲରୁ ସଂଗ୍ରହ କରିଥିଲେ କି?<br>(ଜୁନ, ୨୦୧୫ ଠାରୁ ମେ, ୨୦୧୬)	**Yes/ହଁ--- 1**<br>**No/ନା--- 0**	Quantity collected or grown/ ସଂଗ୍ରହ କରିଥିବା କିମ୍ବା ବଢାଇଥିବା ଦ୍ରବ୍ୟର ପରିମାଣ	Unit/ଏକକ	Was it gathered / foraged from wild, or produced at home?<br>ଏହାକୁ ଆପଣ ଜଙ୍ଗଲରୁ ଆଣିଥିଲେ ନା ଘରେ ଉତ୍ପାଦନ କରିଥିଲେ ?<br>**Gathered/ସଂଗ୍ରହ କରିଥିଲେ -----------0**<br>**Produced/ଉତ୍ପାଦନ କରିଥିଲେ ------------ 1**<br>**Gathered and produced/ସଂଗ୍ରହ ଏବଂ ଉତ୍ପାଦନ କରିଥିଲେ ---2**	Did you add value to the product?<br>ଆପଣ ଏଥିରେ ମୂଲ୍ୟ ଯୁକ୍ତ କରିଥିଲେ କି ?<br>**Yes/ହଁ---1**<br>**No/ନା---0**	Quantity consumed by household<br>ଘରେ ହୋଇଥିବା ଉପଯୋଗର ପରିମାଣ	Quantity sold including value added<br>ମୂଲ୍ୟ ଯୁକ୍ତକରି ବିକ୍ରିର ପରିମାଣ	What was the income from sale, including any value addition? (Rs.)<br>ସମସ୍ତ ମୂଲ୍ୟ ଯୋଗକରି ବିକ୍ରୀରୁ କେତେ ଟଙ୍କା ଆୟ ହୋଇଥିଲା (ଟଙ୍କାରେ)
Wood apple/କଇଥ	☐	☐	☐☐☐	☐	☐	☐	☐	☐
Burokoli berries/ବରକୋଳି (Jujube, Ziziphus jujuba)	☐	☐	☐☐☐	☐	☐	☐	☐	☐
Custard apple/ଆତ	☐	☐	☐☐☐	☐	☐	☐	☐	☐
Kusum/କୁସୁମ	☐	☐	☐☐☐	☐	☐	☐	☐	☐
Kendu fruit/କେନ୍ଦୁ	☐	☐	☐☐☐	☐	☐	☐	☐	☐
Neem flowers/ନିମ୍ବ ଫୁଲ	☐	☐	☐☐☐	☐	☐	☐	☐	☐
Cashew/କାଜୁ	☐	☐	☐☐☐	☐	☐	☐	☐	☐

Did you grow or gather any fruit or any other edible products from the wild, in the past 3 agricultural seasons? (June 2015 to May 2016, whether for consumption or sale)? *Select from list using free recall*<br>ପରିବାରରେ ଉପଯୋଗ ବା ବିକ୍ରି ଉଦ୍ଦ୍ୟେଶରେ ହେଉ,<br>ଗତ ତିନୋଟି କୃଷି ଋତୁ ମଧ୍ଯରେ ଆପଣ କୌଣସି ଜଙ୍ଗଲ ଜାତ ଫଳମୂଳ ଓ ଖାଇବା ଦ୍ରବ୍ୟ ଉତ୍ପାଦନ କରିଥିଲେ ବା ଜଙ୍ଗଲରୁ ସଂଗ୍ରହ କରିଥିଲେ କି?<br>(ଜୁନ, ୨୦୧୫ ଠାରୁ ମେ, ୨୦୧୬)	**Yes/ହଁ--- 1**<br>**No/ନା--- 0**	Quantity collected or grown/ ସଂଗ୍ରହ କରିଥିବା କିମ୍ବା ବଢାଇଥିବା ଦ୍ରବ୍ୟର ପରିମାଣ	Unit/ଏକକ	Was it gathered / foraged from wild, or produced at home?<br>ଏହାକୁ ଆପଣ ଜଙ୍ଗଲରୁ ଆଣିଥିଲେ ନା ଘରେ ଉତ୍ପାଦନ କରିଥିଲେ ?<br>**Gathered/ସଂଗ୍ରହ କରିଥିଲେ -----------0**<br>**Produced/ଉତ୍ପାଦନ କରିଥିଲେ ------------ 1**<br>**Gathered and produced/ସଂଗ୍ରହ ଏବଂ ଉତ୍ପାଦନ କରିଥିଲେ ---2**	Did you add value to the product?<br>ଆପଣ ଏଥିରେ ମୂଲ୍ୟ ଯୁକ୍ତ କରିଥିଲେ କି ?<br>**Yes/ହଁ---1**<br>**No/ନା---0**	Quantity consumed by household<br>ଘରେ ହୋଇଥିବା ଉପଯୋଗର ପରିମାଣ	Quantity sold including value added<br>ମୂଲ୍ୟ ଯୁକ୍ତକରି ବିକ୍ରିର ପରିମାଣ	What was the income from sale, including any value addition? (Rs.)<br>ସମସ୍ତ ମୂଲ୍ୟ ଯୋଗକରି ବିକ୍ରୀରୁ କେତେ ଟଙ୍କା ଆୟ ହୋଇଥିଲା (ଟଙ୍କାରେ)
Groundnut/ବାଦାମ								
Indian gallnut/ହରିଡା/ବାହାଡା								
Roots including arrow root/ମୂଳ								
Spices / seeds including huckleberry seeds/ମସଲା/ମଞ୍ଜି								
Items sold as oil e.g. karanja, kusum/କରଞ୍ଜ/କୁସୁମ								
Herbs /ଚେରମୂଳୀ								
Tamarind/ତେନ୍ତୁଳୀ								

Did you grow or gather any fruit or any other edible products from the wild, in the past 3 agricultural seasons? (June 2015 to May 2016, whether for consumption or sale)? *Select from list using free recall*<br>ପରିବାରରେ ଉପଯୋଗ ବା ବିକ୍ରି ଉଦ୍ଦେଶରେ ହେଉ,<br>ଗତ ତିନୋଟି କୃଷି ଋତୁ ମଧ୍ୟରେ ଆପଣ କୌଣସି ଜଙ୍ଗଲ ଜାତ ଫଳମୂଳ ଓ ଖାଇବା ଦ୍ରବ୍ୟ ଉତ୍ପାଦନ କରିଥିଲେ ବା ଜଙ୍ଗଲରୁ ସଂଗ୍ରହ କରିଥିଲେ କି?<br>(ଜୁନ, ୨୦୧୫ ଠାରୁ ମେ, ୨୦୧୬)	**Yes/ହଁ--- 1**<br>**No/ନା--- 0**	Quantity collected or grown/ ସଂଗ୍ରହ କରିଥିବା କିମ୍ବା ବଢାଇଥିବା ଦ୍ରବ୍ୟର ପରିମାଣ	Unit/ଏକକ	Was it gathered / foraged from wild, or produced at home?<br>ଏହାକୁ ଆପଣ ଜଙ୍ଗଲରୁ ଆଣିଥିଲେ ନା ଘରେ ଉତ୍ପାଦନ କରିଥିଲେ ?<br>**Gathered/ସଂଗ୍ରହ କରିଥିଲେ -----------0**<br>**Produced/ଉତ୍ପାଦନ କରିଥିଲେ ------------ 1**<br>**Gathered and produced/ସଂଗ୍ରହ ଏବଂ ଉତ୍ପାଦନ କରିଥିଲେ ---2**	Did you add value to the product?<br>ଆପଣ ଏଥିରେ ମୂଲ୍ୟ ଯୁକ୍ତ କରିଥିଲେ କି ?<br>**Yes/ହଁ---1**<br>**No/ନା---0**	Quantity consumed by household<br>ଘରେ ହୋଇଥିବା ଉପଯୋଗର ପରିମାଣ	Quantity sold including value added<br>ମୂଲ୍ୟ ଯୁକ୍ତକରି ବିକ୍ରିର ପରିମାଣ	What was the income from sale, including any value addition? (Rs.)<br>ସମସ୍ତ ମୂଲ୍ୟ ଯୋଗକରି ବିକ୍ରୀରୁ କେତେ ଟଙ୍କା ଆୟ ହୋଇଥିଲା (ଟଙ୍କାରେ)
Herbal medicines e.g. arjuna, neem/ ଚେରମୂଳୀ ଔଷଧ ଯଥା ଅର୍ଜୁନ, ନିମ୍ବ	☐	☐	☐☐☐	☐	☐	☐	☐	☐
Other edible NTFP,<br>ଅନ୍ୟାନ୍ୟ ଖାଦ୍ୟ ଉପଯୋଗୀ ଜଙ୍ଗଲଜାତ ଦ୍ରବ୍ୟ	☐	☐	☐☐☐	☐	☐	☐	☐	☐

15. Non-edible Non-Timber Forest Produces (NTFPs) /ଅଣ-ଖାଦ୍ୟ କାଠ ବ୍ୟତୀତ ଅନ୍ୟାନ୍ୟ ଜଙ୍ଗଲ ଜାତ ଦ୍ରବ୍ୟ

Variable name	Question	Code	Answer
ntpf_nonfood	Did you collect / gather / forage any non-edible forest products like wood, leaves, brooms, frankincense<br>ଆପଣ ଅଣ-ଖାଦ୍ୟ ଜଙ୍ଗଲ ଜାତ ଦ୍ରବ୍ୟ ଯଥା: କାଠ, ପତ୍ର, ଝାଡୁ, ଝୁଣା ଆଦି ସଂଗ୍ରହ କରିଥିଲେକି ?	Yes/ହଁ---------------1<br>No/ନା----------------0<br>→ *If 0, skip to section 15*	☐

Item/ଦ୍ରବ୍ୟ	Quantity collected ସଂଗ୍ରହର ପରିମାଣ	Unit/ଏକକ<br>**Kg/**କି.ଗ୍ରା-----------------1<br>**Bundle/**ବିଡା **----------------2**<br>**Bags / net/**ବସ୍ତା-----------**3**<br>**Unit item/**ଏକକ ଦ୍ରବ୍ୟ-----**4**<br>**Mana (basket)** ମାଣ-------**5**	Did you add value to the product? ଆପଣ ଏହି ଉତ୍ପାଦରେ ମୂଲ୍ୟ ଯୁକ୍ତ କରିଥିଲେ କି ?<br>**Yes /**ହଁ-------------------**1**<br>**No/**ନା---------------------**0**	Quantity sold ବିକ୍ରିର ପରିମାଣ<br>→ *If 0, skip to next NTFP*	If any sold, what was the income from sale? (Rs.)<br>ଯଦି କୌଣସି ବିକ୍ରି ହୋଇଥିଲା ବିକ୍ରିରୁ ମୋଟ ଆୟକେତେ ହୋଇଥିଲା
Brooms/ଝାଡୁ	☐	☐	☐	☐	☐
Wood / sticks/କାଠ	☐	☐	☐	☐	☐
Cotton (simili)/ଶିମିଳି ତୁଳା	☐	☐	☐	☐	☐
Leaves/ପତ୍ର	☐	☐	☐	☐	☐

Item/ଦ୍ରବ୍ୟ	Quantity collected ସଂଗ୍ରହର ପରିମାଣ	Unit/ଏକକ<br><br>**Kg/କି.ଗ୍ରା------------------1**<br>**Bundle/ବିଡା ----------------2**<br>**Bags / net/ବସ୍ତା------------3**<br>**Unit item/ଏକକ ଦ୍ରବ୍ୟ-----4**<br>**Mana (basket) ମାଣ-------5**	Did you add value to the product? ଆପଣ ଏହି ଉତ୍ପାଦରେ ମୂଲ୍ୟ ଯୁକ୍ତ କରିଥିଲେ କି ?<br><br>**Yes /ହଁ--------------------1**<br>**No/ନା---------------------0**	Quantity sold ବିକ୍ରିର ପରିମାଣ<br><br>*→ If 0, skip to next NTFP*	If any sold, what was the income from sale? (Rs.)<br><br>ଯଦି କୌଣସି ବିକ୍ରି ହୋଇଥିଲା ବିକ୍ରିରୁ ମୋଟ ଆୟକେତେ ହୋଇଥିଲା
Potash/ପଟାଶ					
Other non-edible NTFP ଅନ୍ୟାନ୍ୟ ଖାଦ୍ୟ ଅନୁପଯୋଗୀ ଜଙ୍ଗଲଜାତ ଦ୍ରବ୍ୟ					

15. COSTS/ ମୂଲ୍ୟଖର୍ଚ୍ଚ/

Cost incurred in agriculture (crops, animal husbandry / fishing, fruit and minor forest products) during last one year (June 2015 to May 2016) for the following items/ ଗତ ଜୁନ,୨୦୧୫ ଠାରୁ ମେ, ୨୦୧୬ ମଧ୍ୟରେ ଆପଣ କୃଷିକାର୍ଯ୍ୟ ପାଇଁ ନିମ୍ନଲିଖିତ ଦ୍ରବ୍ୟରେ କେତେ ଖର୍ଚ୍ଚ କରିଥିଲେ (ଶସ୍ୟ, ପଶୁ ପାଳନ,ମତ୍ସ୍ୟ,ଫଳ, ଚାଷ ଓ ଅନ୍ୟାନ୍ୟ ଜଙ୍ଗଲ ଜାତ ଦ୍ରବ୍ୟ)

For food processing costs, do not include costs of food processing for own consumption by household. Only include food processing costs for products that were sold/ ଖାଦ୍ୟ ପ୍ରକ୍ରିୟାକରଣ ଖର୍ଚ୍ଚରେ ପରିବାର ନିଜଘରେ ଖର୍ଚ୍ଚ କରିଥିବା ଖାଦ୍ୟ ପ୍ରକ୍ରିୟାକରଣକୁ ଅନ୍ତର୍ଭୁକ୍ତ କରନ୍ତୁ ନାହିଁ, ବିକ୍ରି ହୋଇଥିବା ଉତ୍ପାଦନ ଗୁଡିକର ଖାଦ୍ୟ ପ୍ରକ୍ରିୟାକରଣ ଖର୍ଚ୍ଚକୁ ଅନ୍ତର୍ଭୁକ୍ତ କରନ୍ତୁ ।.

Variable name	Question	Answer
cost_seed	15.2. Seeds / Plants/ବିହନ/ଚାରା	Rs/ଟଙ୍କା.
cost_fertilizer	15.3. Fertilizers/ରାସାୟନିକ ସାର	Rs/ଟଙ୍କା.
cost_pesticide	15.4. Pesticides/ କୀଟନାଶକ	Rs/ଟଙ୍କା.
cost_livestock	15.5. Purchase of livestock/ପଶୁସମ୍ପଦ କିଣିବା	Rs/ଟଙ୍କା.
cost_vacc	15.6. Vaccinations for livestock/ପଶୁମାନଙ୍କ ଟୀକାକରଣ	Rs/ଟଙ୍କା.
cost_labour	15.7. Wage labour/ଦିନ ମଜୁରିଆ	Rs/ଟଙ୍କା.
cost_labour_hh	15.8. How many household members (including yourself) were involved in agriculture during last one year (June 2015 to May 2016)? ଆପଣଙ୍କ ପରିବାରର କେତେଜଣ ସଦସ୍ୟ (ଆପଣଙ୍କୁ ମିଶାଇ) ଗତ ଜୁନ, ୨୦୧୫ ଠାରୁ ମେ, ୨୦୧୬ ମଧ୍ୟରେ ଚାଷ କାର୍ଯ୍ୟରେ ଅଂଶଗ୍ରହଣ କରିଥିଲେ ?	
cost_labour_hhmonths	15.9. On average, how many months in the last year (June 2015 to May 2016) did household members participate in agricultural work? ଗତ ଜୁନ, ୨୦୧୫ ଠାରୁ ମେ, ୨୦୧୬ ମଧ୍ୟରେ ଆପଣଙ୍କ ପରିବାରର ସଦସ୍ୟମାନେ ହାରାହାରି କେତେ ମାସ ଚାଷକାର୍ଯ୍ୟରେ ଅଂଶଗ୍ରହଣ କରିଥିଲେ ?	

cost_labour_hhdays	15.10. On average, how many days per month did household members participate in agricultural work?<br>ଆପଣଙ୍କ ପରିବାରର ସଦସ୍ୟମାନେ ଏକ ମାସରୁ ହାରାହାରି କେତେ ଦିନ ଚାଷକାର୍ଯ୍ୟରେ ଅଂଶଗ୍ରହଣ କରିଥିଲେ ?	
cost_labour_hhhours	15.11. On average, how many hours per day did household members participate in agricultural work?<br>ଆପଣଙ୍କ ପରିବାରର ସଦସ୍ୟମାନେ ଗୋଟିଏ ଦିନରେ ହାରାହାରି କେତେ ଘଣ୍ଟା ଚାଷକାର୍ଯ୍ୟରେ ଅଂଶଗ୍ରହଣ କରିଥିଲେ ?	
cost_transport	15.12. Transportation costs for all agricultural related activities/ଚାଷବାସ ପାଇଁ ଯିବା ଆସିବା ଖର୍ଚ୍ଚ (including purchase of inputs, accessing agriculture services and training and marketing) for (ଚାଷ ନିମନ୍ତେ ଉପକରଣ କ୍ରୟ, କୃଷି ସେବା ପାଇବା, ତାଲିମ ଓ ବିକା କିଣା)	Rs/ଟଙ୍କା.
cost_plough	15.13. Ploughs (rented individually or through sharing)/ଲଙ୍ଗଳ (ନିଜେ କିମ୍ବା ଅନ୍ୟ କାହାସହ ଭାଗରେ ଭଡା ନେଇଥିଲେ)	Rs/ଟଙ୍କା.
cost_cart	15.14. Bullock carts (rented individually or through sharing)<br>ଶଗଡଗାଡି (ନିଜେ କିମ୍ବା ଅନ୍ୟ କାହାସହ ଭାଗରେ ଭଡା ନେଇଥିଲେ)	Rs/ଟଙ୍କା.
cost_tractor	15.15. Tractor (rented individually or through sharing)<br>ଟ୍ରାକ୍ଟର(ନିଜେ କିମ୍ବା ଅନ୍ୟ କାହାସହ ଭାଗରେ ଭଡା ନେଇଥିଲେ)	Rs/ଟଙ୍କା.
cost_spraypump	15.16. Spray pump (rented individually or through sharing)<br>ସ୍ପ୍ରେ ପମ୍ପ (ନିଜେ କିମ୍ବା ଅନ୍ୟ କାହାସହ ଭାଗରେ ଭଡା ନେଇଥିଲେ)	Rs/ଟଙ୍କା.
cost_pumpset	15.17. Pump set (rented individually or through sharing)<br>ପମ୍ପ ସେଟ (ନିଜେ କିମ୍ବା ଅନ୍ୟ କାହାସହ ଭାଗରେ ଭଡା ନେଇଥିଲେ)	Rs/ଟଙ୍କା.
cost_thresher	15.18. Thresher (rented individually or through sharing)<br>ଧାନ ଅମଳ ଯନ୍ତ୍ର(ନିଜେ କିମ୍ବା ଅନ୍ୟ କାହାସହ ଭାଗରେ ଭଡା ନେଇଥିଲେ)	Rs/ଟଙ୍କା.
cost_tiller	15.19. Power Tiller (rented individually or through sharing)<br>ପାୱାର ଟିଲର(ନିଜେ କିମ୍ବା ଅନ୍ୟ କାହାସହ ଭାଗରେ ଭଡା ନେଇଥିଲେ)	Rs/ଟଙ୍କା.
cost_equip	15.20. Other Equipment (rented individually or through sharing) ଅନ୍ୟାନ୍ୟ ଉପକରଣ (ନିଜେ କିମ୍ବା ଅନ୍ୟ କାହାସହ ଭାଗରେ ଭଡା ନେଇଥିଲେ)	Rs/ଟଙ୍କା.
cost_equip_bought	15.21. Purchased equipment (mentioned above such as plough, tractor, spray pump etc)<br>କ୍ରୟ କରିଥିବା ଉପକରଣ (ଲଙ୍ଗଳ, ଟ୍ରାକ୍ଟର, ସ୍ପ୍ରେ ପମ୍ପ ଇତ୍ୟାଦି)	Rs/ଟଙ୍କା.

cost_irrigate	15.22. Water/Irrigation/ଜଳ ସେଚିତ କରିବାରେ	Rs/ଟଙ୍କା.
cost_elec	15.23. Electricity/ବିଦ୍ୟୁତ	Rs/ଟଙ୍କା.
cost_fuel	15.24. Fuel costs for farming, irrigation and processing/ଚାଷ ପାଇଁ ଜଳ ସେଚନ ଓ ଧାନ ଅମଳ ନିମନ୍ତେ ଜାଳେଣୀ	Rs/ଟଙ୍କା.
cost_loan	15.25. Interest on outstanding agricultural loan/କୃଷିଋଣ ଉପରେ ସୁଧ	Rs/ଟଙ୍କା.
cost_tax	15.26. Taxes/କର/ଶୁଳ୍କ	Rs/ଟଙ୍କା.
cost_insure	15.27. Insurance/ବୀମା	Rs/ଟଙ୍କା.
cost_marketing	15.28. Costs for any other marketing activities/କୌଣସି ବିକା କିଣା ପାଇଁ ଖର୍ଚ୍ଚ	Rs/ଟଙ୍କା.
cost_lease	15.29. Cost for leasing-in/Share cropping-in/Mortgage-in (excluding the agriculture produce given back) ଲିଜ/ଭାଗ ଚାଷ/ଜମି ବନ୍ଧା ଖର୍ଚ୍ଚ(କୃଷି ଉତ୍ପାଦିତ ଦ୍ରବ୍ୟକୁ ବାଦଦେଇ)	Rs/ଟଙ୍କା.
cost_storage	15.30. Storage/ସରକ୍ଷଣ	Rs/ଟଙ୍କା.____________

16. Entitlements / Agriculture Extension Services/ହିତାଧିକାରୀ କୃଷି ସମ୍ପ୍ରସାରଣ ସେବା

Variable	Question	Code	Answer
Card/କାର୍ଡ	16.1.<br><br>Does your household have any of the following cards? (read out the list)<br>ନିମ୍ନଲିଖିତ କାର୍ଡଗୁଡିକ ମଧ୍ୟରୁ ଆପଣଙ୍କ ପରିବାରର କୌଣସି କାର୍ଡ ରହିଛି କି ?	No card/କୌଣସି କାର୍ଡ ନାହିଁ ---------------------------- 0<br>APL/ଏ.ପି.ଏଲ-- 1<br>Ration card/ରାସନ କାର୍ଡ------------------------------- 2<br>Annapurna Card/ଅନ୍ନପୂର୍ଣ୍ଣା କାର୍ଡ----------------------- 3<br>Antyodaya Anna Yozana Card/ଅନ୍ତୋଦୟ କାର୍ଡ ଯୋଜନା-- 4<br>Job card (MGNREGA)/ଜବ କାର୍ଡ --------------------- 5<br>Labourer ID card/ଶ୍ରମିକ କାର୍ଡ ------------------------ 6<br>Aadhar card/ଆଧାର କାର୍ଡ଼------------------------------ 7<br>Kishan credit card (provides crop insurance) କିଷାନ କ୍ରେଡିଟ କାର୍ଡ଼(ଶସ୍ୟ ବୀମା)--------------------------------- 8	☐
mgnrega_days	16.2 During last one year before this interview, how many days of work did you get under MGNREGA?<br>ଗତବର୍ଷ ଏହି ସାକ୍ଷାତକାର ପୂର୍ବରୁ ଆପଣ MGNREGA ରେ କେତେଦିନ କାମ କରିଛନ୍ତି ?		☐ days/ଦିନ<br><br>→ *If 0, skip to 16.4*
mgnrega_cash	16.3 During last one year before this interview, how much (in Rs.) did you receive the work you did under MGNREGA?<br>ଗତବର୍ଷ ଏହି ସାକ୍ଷାତକାର ପୂର୍ବରୁ ଆପଣ MGNREGA ରେ କାମକରି କେତେ ଟଙ୍କା ପାଇଛନ୍ତି ?		Rs/ଟଙ୍କା. ☐

16.4 Did you or any household member receive any of the following agriculture extension services during last one year (June 2015 to May 2016)?
ଗତ ଜୁନ, ୨୦୧୫ ଠାରୁ ମେ, ୨୦୧୬ ମଧ୍ୟରେ ଆପଣଙ୍କ ପରିବାରର କୌଣସି ସଦସ୍ୟ ନିମ୍ନଲିଖିତ କୃଷି ଜନିତ ସେବା ପାଇଛନ୍ତି କି ?

Multiple response options/ ବହୁ ଉତ୍ତର ସମ୍ଭବ:

Variable	Question	Code	Answer
extension_demo	Demonstrations / awareness generation on cropping practices or technology use<br>ଚାଷ ପ୍ରଥା କିମ୍ବା ମେସିନ ବ୍ୟବହାର ଉପରେ ପ୍ରଦର୍ଶନ ବା ସଚେତନତା<br>(ATMA-Agriculture technology Management Agency)(ଆତ୍ମା)କୃଷି ବୈଷୟକ ପରିଚାଳନା ସଂସ୍ଥା	Yes/ହଁ---1<br>No/ନା--- 0	
extension_soil	Soil Testing Services<br>ମୃତ୍ତିକା ପରୀକ୍ଷା ସେବା<br>Rashtriya Krishi Vikas Yojana (RKVY),<br>*ରାଷ୍ଟ୍ରିୟ କୃଷି ବିକାଶ ଯୋଜନା* ATMA and (NFSM)National food security mission<br>*ରାଷ୍ଟ୍ରୀୟ ଖାଦ୍ୟ ସୁରକ୍ଷା ମିସନ*	Yes/ହଁ---1<br>No/ନା-- 0	
extension_subsidy	Subsidy for seed, fertiliser, bio-fertiliser or insurance<br>ବିହନ, ସାର, ଜୈବିକ ସାର କିମ୍ବା ବୀମା ନିମନ୍ତେ ରିହାତି e.g. NODP-National organic development program ରାଷ୍ଟ୍ରିୟ ଜୈବିକ ବିକାଶ କାର୍ଯକ୍ରମ<br>/ RKBY (Rashtriya Krishi Bima Yojana) /ରାଷ୍ଟ୍ରୀୟ କୃଷି ବୀମା ଯୋଜନା Kissan Credit Cardକିଶାନ କ୍ରେଡିଟ କାର୍ଡ	Yes/ହଁ---1<br>No/ନା-- 0	
extension_loans	Provision of agriculture loans<br>କୃଷି ଋଣ ନିମନ୍ତେ ବ୍ୟବସ୍ଥା e.g. (NABARD- National Bank of agriculture and Rural Development. ରାଷ୍ଟ୍ରିୟ କୃଷି ଏବଂ ଗ୍ରାମୀଣ ବିକାଶ ବ୍ୟାଙ୍କ	Yes/ହଁ---1<br>No/ନା-- 0	
extension_irrigate	Irrigationଜଳସେଚନ<br>OLIC (Odisha Lift Irrigation Corporation) ଓଡିଶା ଉଠା ଜଳଶେଚନ ନିଗମ	Yes/ହଁ---1<br>No/ନା-- 0	
extension_market	Marketing of produce (buyer and seller linkages)<br>ଉତ୍ପାଦନର ବଜାରକରଣ (କ୍ରେତା ଓ ବିକ୍ରେତା ମଧ୍ୟରେ ଯୋଗ ସୂତ୍ର)<br>Krusi Bazr, RMC (Regulated Market Committee) ନିୟନ୍ତ୍ରିତ ବଜାର କମିଟି	Yes/ହଁ---1<br>No/ନା-- 0	
extension_storage	Storage services<br>(Department of Co-operation)<br>ଭଣ୍ଡାରରେ ରଖିବା ସୁବିଧା	Yes/ହଁ---1<br>No/ନା-- 0	

Variable	Question	Code	Answer
extension_train	Trainingତାଲିମ, e.g. from Department of Agriculture and Dept of Horticulture କୃଷି ଓ ଉଦ୍ୟାନ ବିଭାଗ	Yes/ହଁ---1<br>No/ନା--- 0	☐<br>→ *If 0, skip to section 17.*
extension_train_topics	16.4.1 What topics did you / the household member receive training on?<br>କେଉଁ ବିଷୟରେ ଆପଣଙ୍କ ପରିବାରର ସଦସ୍ୟ ତାଲିମ ପ୍ରାପ୍ତ ହୋଇଥିଲେ ?	<tick all that apply><br>☐ Crop selection or rotation advice/ଶସ୍ୟ ଚୟନ ଉପଦେଶ =A<br>☐ Improved seeds or crop varieties/ଉନ୍ନତ ବିହନ କିମ୍ବା ଶସ୍ୟର ପ୍ରକାର = B<br>☐ Pest management/କୀଟ ସଂଚାଳନ = C<br>☐ Soil improvement/ମୃତ୍ତିକାର ଉନ୍ନତିକରଣ =D<br>☐ Veterinary/animal training/ପଶୁ ଚିକିତ୍ସା/ପଶୁ ପାଳନ ପ୍ରଶିକ୍ଷଣ = E<br>☐ Other/ଅନ୍ୟାନ୍ୟ = X	
extension_train_provider	16.4.2 Who provided this training?<br>କିଏ ତାଲିମ ପ୍ରଦାନ କରିଥିଲେ ?	- Government/ସରକାର----------------------- A<br>- NGO / community organisation/ଏନ.ଜି.ଓ/ଗୋଷ୍ଠୀ ସଂଗଠନ----- B<br>- Religious Group/ଧାର୍ମିକ ଗୋଷ୍ଠୀ-------------- C<br>- Private company / business, e.g. NABARD bank/ବେସରକାରୀ ସଂଗଠନ/ ବ୍ୟବସାୟ/ନାବାର୍ଡ ବ୍ୟାଙ୍କ--- D<br>- Other/ଅନ୍ୟାନ୍ୟ------------------------------- X	☐

18. Debts and loans

account	18.1 Does any household member have a bank account or post office account/ଆପଣ ପରିବାରର କୌଣସି ସଦସ୍ୟଙ୍କର ବ୍ୟାଙ୍କ ବା ଡାକଘରେ ଖାତା ଖୋଲା ଯାଇଛି କି ?	Yes/ହଁ--1<br>No/ନା--- 0<br>If no, go to 18.3/ଯଦି ନାହିଁ, ତେବେ 18.3କୁ ଯାଆନ୍ତୁ	☐
account_name	18.2 In whose name is the account/ଖାତାଟି କାହା ନାମରେ ଖୋଲା ଯାଇଅଛି ?	Select all that apply<br>☐ Self (Respondent)/ ନିଜ ନାଁରେ (ଉତ୍ତରଦାତା)<br>☐ Primary caregiver of the index child/ଚୟନ ଶିଶୁର ପ୍ରାଥମିକ ଯତ୍ନକାରୀ<br>☐ Other male household member(s)/ପରିବାରର ଅନ୍ୟ ପୁରୁଷ ସଦସ୍ୟଙ୍କ ନାମରେ<br>☐ Other female household member(s)/ ପରିବାରର ଅନ୍ୟ ମହିଳା ସଦସ୍ୟଙ୍କ ନାମରେ	
debt	18.3 What is the value of your household's outstanding debts or loans (with formal and informal sources), if any/ଆପଣଙ୍କ ପରିବାରର ଆନୁଷ୍ଠାନିକ ଏବଂ ଅଣ-ଆନୁଷ୍ଠାନିକ ଋଣର ମୂଲ୍ୟ କେତେ?	☐ Rs (ଟଙ୍କା)<br>Write '0' if no outstanding debts/ଯଦି କୌଣସି ଋଣ ନାହିଁ ତେବେ 0 ଲେଖନ୍ତୁ<br>→ if 0, go to 18.5	
loan_repay_howmuch	18.4 How much do you pay towards your loan repayment per month/ ଋଣ ବାବଦରେ ଆପଣ ମାସିକ କେତେ ଟଙ୍କା ପଇଠ କରନ୍ତି?	☐ Rs (ଟଙ୍କା)	
savings	18.5 What is the value of your household's savings, if any/ଆପଣଙ୍କ ପରିବାରର ସଞ୍ଚୟ କେତେ?	☐ Rs (ଟଙ୍କା)<br>Write '0' if no savings/ଯଦି କୌଣସି ସଞ୍ଚୟ ନାହିଁ ତେବେ 0 ଲେଖନ୍ତୁ	

19. Shocks

19. Now I'm going to ask you about events that might have happened in the last 90 days that may have affected your household standard of living. These events might have affected your expenditure on foods and non-food items. Overall, did household's food-related and non-food related expenditures increase, decrease, or remain about the same due to these events/ ଗତ ୯୦ ଦିନ ମଧ୍ୟରେ ଏପରି କୌଣସି ଘଟଣା ଘଟିଛି ଯାହା ଆପଣଙ୍କ ପରିବାରର ସାଧାରଣ ଜୀବନଯାପନ ଉପରେ ପ୍ରଭାବ ପକାଇ ଅଛି । ଏହି ଘଟଣା ଆପଣଙ୍କ ପରିବାରର ଖାଦ୍ୟ ଓ ଅଣ-ଖାଦ୍ୟ ଦ୍ରବ୍ୟର ଖର୍ଚ୍ଚ ଉପରେ ପ୍ରଭାବ ପକାଇ ଥାଇପାରେ । ଏହି ଘଟଣା ଯୋଗୁଁ ମୋଟାମୋଟି ଆପଣଙ୍କ ପରିବାରର ଖାଦ୍ୟ ଏବଂ ଅଣ- ଖାଦ୍ୟ ସମ୍ବନ୍ଧୀୟ ଖର୍ଚ୍ଚ ବଢିଲା, ନା କମିଲା ନା ସମ ପରିମାଣ ରହିଲା?					
Variable name	Event	Food items related expenditures/ ଖାଦ୍ୟ ସମ୍ବନ୍ଧୀୟ ଖର୍ଚ୍ଚ		Non-food items expenditures<br>ଅଣ- ଖାଦ୍ୟ ସମ୍ବନ୍ଧୀୟ ଖର୍ଚ୍ଚ	
shock_death	19.1 Death or serious illness of a household member/ ପରିବାରର ସଦସ୍ୟଙ୍କର ମୃତ୍ୟୁ ବା ଗୁରୁତର ଅସୁସ୍ଥତା	Increased/ବଢିଲା ------------1<br>Decreased/କମିଲା ----------2<br>About the same/ପ୍ରାୟ ସମାନ--3<br>No death or illness/ କୌଣସି ମୃତ୍ୟୁ କିମ୍ବା ଅସୁସ୍ଥତା ନାହିଁ---------0<br>→ if 0, go to next shock		Increased/ବଢିଲା ------------1<br>Decreased/କମିଲା ----------2<br>About the same/ପ୍ରାୟ ସମାନ--3	
shock_livelihoods	19.2 Loss of livelihood(e.g. job loss, crop failure, harvest loss)/ ବୃତ୍ତିର କ୍ଷତି (ଯଥା: କାମ ହରାଇବା, ଚାଷରେ ଅସଫଳ, ଅମଳରେ କ୍ଷତି)	Increased/ବଢିଲା ------------1<br>Decreased/କମିଲା ----------2<br>About the same/ପ୍ରାୟ ସମାନ—3<br>No loss of livelihood/ ବୃତ୍ତିର କୌଣସି କ୍ଷତି ନାହିଁ---------------0<br>→ if 0, go to next shock		Increased/ବଢିଲା ------------1<br>Decreased/କମିଲା ----------2<br>About the same/ପ୍ରାୟ ସମାନ--3	
shock_celebrations	19.3 Celebrations/ ଉତ୍ସବ ପାଳନ କରିବା	Increased/ବଢିଲା ------------1<br>Decreased/କମିଲା ----------2<br>About the same/ପ୍ରାୟ ସମାନ—3<br>No celebrations/ କୌଣସି ଉତ୍ସବ ପାଳନ କରିନାହାନ୍ତି --------------0<br>→ if 0, go to next shock		Increased/ବଢିଲା ------------1<br>Decreased/କମିଲା ----------2<br>About the same/ପ୍ରାୟ ସମାନ--3	

shock_gov	19.4 New government policy affecting the use of 500 and 1000 INR notes/ ୫୦୦ ଓ ୧୦୦୦ ଟଙ୍କାର ବ୍ୟବହାର ଉପରେ ସରକାରଙ୍କ ନୂଆ ନିୟମ ପ୍ରଭାବିତ କରୁଛି	Increased/ବଢ଼ିଲା ------------1<br>Decreased/କମିଲା ----------2<br>About the same/ପ୍ରାୟ ସମାନ--3		Increased/ବଢ଼ିଲା ------------1<br>Decreased/କମିଲା ----------2<br>About the same/ପ୍ରାୟ ସମାନ--3		If 3, ask: Did you know/have you heard about this policy?<br>Yes -----1<br>No------0	
shock_calamity	19.5 Natural calamity, and calamity related damage to crop, livestock , food stored, homestead and productive assets/ ପ୍ରାକୃତିକ ବିପର୍ଯ୍ୟୟ, ଏବଂ ଫସଲ, ପଶୁ ସମ୍ପଦ, ଖାଦ୍ୟ ସଂରକ୍ଷଣ, ଘରବାରି ଏବଂ ଉତ୍ପାଦନକ୍ଷମ ସାମଗ୍ରୀର ବିପର୍ଯ୍ୟୟ ସମ୍ବନ୍ଧୀୟ କ୍ଷତି	Increased/ବଢ଼ିଲା ------------1<br>Decreased/କମିଲା ----------2<br>About the same/ପ୍ରାୟ ସମାନ—3<br>No natural calamity/ କୌଣସି ପ୍ରାକୃତିକ ବିପର୍ଯ୍ୟୟ--------0<br>→ if 0, go to next shock		Increased/ବଢ଼ିଲା ------------1<br>Decreased/କମିଲା ----------2<br>About the same/ପ୍ରାୟ ସମାନ--3			

spouse_livesaway	In the past year, how many complete months per year, if any, did you stay away from home for work-based migration?<br>ଗତ ବର୍ଷର କେତୋଟି ମାସ ଆପଣ କାମ କରିବା ନିମନ୍ତେ ଘର ଠାରୁ ବାହାରେ ଯାଇ ରହିଥିଲେ?	<integer>	Display if hh_gender==0<br>**Not more than 12**

***

Note: After the interview, cross check the years of education that was recorded in this interview with the value recorded in the mother / primary caregiver.
ନୋଟ: ସାକ୍ଷାତକାର ପରେ, ଏହି ପ୍ରଶ୍ନାବଳୀରେ ଥିବା ପାଠପଢ଼ାର ସମ୍ପୂର୍ଣ ବର୍ଷକୁ, ମହିଳା ପ୍ରଶ୍ନାବଳୀ ରେ ଥିବା ଉତ୍ତର ସହ ଚେକ କରନ୍ତୁ।
