# Supplementary material for: Upscaling Participatory Action and Videos for Agriculture and Nutrition (UPAVAN) trial comparing three variants of a nutrition-sensitive agricultural extension intervention to improve maternal and child nutritional outcomes in rural Odisha, India: study protocol for a cluster randomised controlled trial
Source: Trials. 2018 Mar 9;19:176. doi: 10.1186/s13063-018-2521-y (PMC5845188; doi:10.1186/s13063-018-2521-y)
Supplement: Supplementary file 4 — Consent forms. (ZIP 1740 kb) [file 13063_2018_2521_MOESM4_ESM.zip › AF4_Cluster consent form.pdf]

LONDON SCHOOL of HYGIENE &TROPICAL MEDICINE

PARTICIPANT CONSENT FORM

Title of Project: UPAVAN: Upscaling Participation and Videos for Agriculture and Nutrition

Name of PI/Researcher responsible for project: Suneetha Kadiyala

**Statement**	**Please initial or thumbprint* each box**
I confirm that I have read the information sheet dated…………………(version…….) for the above named study. I have had the opportunity to consider the information, ask questions and have these answered satisfactorily.<br>**<u>OR</u>**<br>I have had the information explained to by study personnel in a language that I understand. I have had the opportunity to consider the information, ask questions and have these answered satisfactorily.	
I understand that the participation of [cluster name] is voluntary and that [cluster name] is free to withdraw at any time without giving any reason, without my / the community's medical care or legal rights being affected.	
I give permission for London School of Hygiene and Tropical Medicine, and Digital Green, to have access to this record.	
I understand that the participation and information may be used to support other research in the future, and may be shared anonymously with other researchers, for their ethically-approved projects.	
I, am suitably placed to represent and sign permission on behalf of [cluster name], for the participation in the above-named study.	
I, on behalf of [cluster name], agree for the village / surrounding areas to take part in the above-named study.	

Printed name of participant	Signature of participant	Date

Printed name of impartial witness*	Signature of impartial witness*	Date

I attest that I have explained the study information accurately in _________________ to, and was understood to the best of my knowledge by, the participant and that he/she has freely given their consent for the village to participate* in the presence of the above named impartial witness (where applicable).

Printed name of person obtaining consent	Signature of person obtaining consent	Date

[*Only required if the participant is unable to read or write.]

A copy of this informed consent document has been provided to the participant.

Centre Number:
Study Number:
Participant Identification Number:

[Informed Consent for Participant with Impartial witness_19.02.15_v1]
