# Supplementary material for: Upscaling Participatory Action and Videos for Agriculture and Nutrition (UPAVAN) trial comparing three variants of a nutrition-sensitive agricultural extension intervention to improve maternal and child nutritional outcomes in rural Odisha, India: study protocol for a cluster randomised controlled trial
Source: Trials. 2018 Mar 9;19:176. doi: 10.1186/s13063-018-2521-y (PMC5845188; doi:10.1186/s13063-018-2521-y)
Supplement: Supplementary file 4 — Consent forms. (ZIP 1740 kb) [file 13063_2018_2521_MOESM4_ESM.zip › AF4_Cluster information sheet.pdf]

UPAVAN: Upscaling Participation and Videos for Agriculture and Nutrition

Village level information sheet

INVITATION AND SUMMARY

We'd like to invite your village and surrounding hamlets to take part in our research study: "UPAVAN: Upscaling Participation and Videos for Agriculture and Nutrition". The village's participation in the study is entirely up to you [village level head] and whomever you choose to consult in this decision.

Before you decide, we would like you to understand why the research is being done and what the project would involve for you. A member of our team will go through this information sheet with you, to help you decide whether or not you would like the village to take part and to answer any questions you may have.

We will speak to all relevant village heads, so we are only asking you about the area that you are responsible for. You may like to discuss this with other people in your community, before you decide. Please let us know if you would like us to come back again in a few days, to give you/ your community time to make a decision.

The information sheet tells you the purpose of the study, what the study involves, how villages will be selected, and any risks and benefits of the study. This should take about 15 minutes.

- If you have questions about anything in this form, ask the research team for more information.
- You may wish to talk to other community members about the village's participation in this study. We can come back again, after we have explained the study to you.
- Do not agree on behalf of your village to participate in this study unless the research team has answered your questions and you decide that you want the village to be part of this study.

By signing this form (or putting your thumbprint), you are agreeing for the village to participate in this study.

WHAT IS THE PURPOSE OF THIS STUDY?

This is a research study. The purpose of this study is to understand whether and how new interventions to improve agriculture practices in Keonjhar can result in improved health and nutrition for women and children. If we can improve health and nutrition for women and children here, the same approach may also help women and children in other regions.

A lot of work has been done to understand whether agricultural programs like kitchen gardening and agricultural extension programs can improve nutrition outcomes, such as dietary quality, body weight, and growth of children. We know that improvements in nutrition are more likely when agriculture interventions target women and include activities to boost their empowerment in agriculture and nutrition-related decisions. We don't know enough about what exactly works to improve maternal and child nutrition - or how - and we don't know enough about how to design innovative approaches to stimulate agriculture's contribution to nutrition.

The intervention will particularly focus on mothers of children under 2 years of age, and pregnant women. This focus is because the nutrition of young children and pregnant women has been identified by researchers and policy makers as particularly important. We hope that, by targeting these groups, children will grow up to be healthier adults, leading to improved nutrition of the whole community.

Why Keonjhar?

Undernutrition is particularly high in Keonjhar. Almost half (48%) of children under 5 years old are short for their age because they have inadequate nutrition, and quarter of children under 5 years are too thin. This is worse than Odisha's average, which is already low compared to the national average. Furthermore, the percentage of underweight children has actually increased since 2002, so work is urgently needed to discover how we can improve nutrition in Keonjhar.

WHO IS RUNNING THIS RESEARCH PROJECT?

This project will be implemented by three non-governmental organisations that are working in agriculture and rural development in India: VARRAT, Digital Green, and Ekjut.

- **VARRAT** (Voluntary Association for Rural Reconstruction and Appropriate Technology) is a not-for-profit organisation from Odisha that supports rural communities by strengthening people's organisations, innovating appropriate technology, and upgrading skills of groups and workers.
- **Digital Green** is a not-for-profit international development organisation that uses videos to engage communities and to improve lives of rural communities across South Asia and Sub-Saharan Africa. They are currently implementing projects in collaboration with over 20 partner organisations across 9 states in India and parts of Ethiopia, Afghanistan, Ghana, Niger and Tanzania.
- **Ekjut** is a not-for-profit organisation working in Odisha and Jharkhand to improve maternal, newborn and child health of partnering underserved, marginalised communities. Ejkut works by empowering communities with community-based interventions, and influencing good governance to improve access and quality of services.

Interventions will be implemented and monitored in consultation with:

- **London School of Hygiene & Tropical Medicine** (a centre for research and education in public and global health, based in London, UK), and
- **Institute for Global Health, University College London** (a centre of research and teaching in global health, also based in London, UK).
- **SPRING** (Strengthening Partnerships, Results, and Innovations in Nutrition Globally), a project based in USA that is dedicated to strengthening global and country efforts to scale up high-impact nutrition practices and policies.
- **DCOR** (a non-governmental consultancy organisation that specialises in conducting interviews and data collection in Odisha).

WHAT WILL HAPPEN DURING THIS STUDY?

Women in your village will be invited to participate in one of four study arms:

Arm 1 = what is happening in your village at present

Community members receive standard agriculture (e.g. subsidies and extension), health (e.g. Vitamin A supplementation; immunizations) and nutrition related services (e.g. iron and folic acid supplementation; supplementary feeding through the Integrated Child Development Services [ICDS]) provided by the Government (or other organisations) in this area.

There is no additional intervention provided by us in this arm, because it will be used as the comparison group. However, we will refer severely undernourished women and children to the nearest health facility.

Arm 2 = Agriculture videos, group discussion and home visits:

In addition to any existing programs from the government/other organisations, women's self-help groups will receive an agriculture extension intervention from VARRAT and Digital Green. Women's groups will meet around twice per month for approximately 36 months - at a date, time and venue decided by the group. At the groups, a VARRAT frontline worker will screen locally produced videos addressing agriculture topics prioritised by the community. Topics might include: access to credit, crop diversification, use of fertiliser, ways to add value to agricultural production, or strategies to reduce post-harvest losses. Where appropriate, topics will also be linked to nutrition, by showing how improved or new agricultural practices can also improve nutrition. At the screening, the VARRAT frontline worker will pause the video at strategic points, to facilitate a discussion about what the viewers see in the video.

A few days after the screening, the VARRAT frontline worker will visit group members at their home to verify whether they have tried the recommended practice. VARRAT frontline workers will selectively visit pregnant women and women with children under 2 years of age.

Attendance to the groups, video screenings and home visits are completely voluntary – group members will not have to pay anything to attend and will also not be paid for their attendance.

Arm 3 = Agriculture and nutrition videos, group discussion and home visits:

Women's self-help groups will receive a similar agriculture extension intervention from VARRAT and Digital Green, but around half of the agriculture videos will be replaced with videos on maternal, infant and young child nutrition. Nutrition topics may include: breastfeeding practices, handwashing, and dietary intakes. For this intervention, women will be invited to attend 2 screenings per month for approximately 36 months (roughly one agriculture and one nutrition video per month, although some months may have 2 agriculture videos and other months may have 2 nutrition videos) VARRAT frontline workers will selectively visit pregnant women and women with children under 2 years of age.

Attendance to the groups, video screenings and home visits is completely voluntary – group members will not have to pay anything to attend and will also not be paid for their attendance.

Arm 4 = Agriculture videos and nutrition videos, group discussion, home visits, and further enhanced participatory women's groups:

Women's self-help groups will receive a similar agriculture extension intervention from VARRAT and Digital Green, with agriculture and nutrition video screening and discussion through women's groups, home visits to monitor uptake of new practices, as in arm 3. As in arms 2 and 3, there will be 2 meetings per month in each women's group, over a 36-month period.

In addition, there will also be some participatory group meetings that are run by VARRAT, with support from Ekjut. There will only be one of these participatory groups per village cluster, rather than one per existing self-help women's group. This component of participatory group meetings involves group discussion of maternal and child nutrition through interactive methods like games and role play. This is followed by the prioritisation of local issues relating to nutrition, consultation with the wider community, and implementation of strategies to address these barriers to improved nutrition. Strategies that these types of participatory women's groups have implemented in other parts of India and other countries include: group funds, social dramas, kitchen gardening, and cooking demonstrations.

Attendance to any of the groups, video screenings and home visits is completely voluntary – group members will not have to pay anything to attend and will also not be paid for their attendance.

Interviews

Before and at the end of the interventions, we will interview mothers and fathers of children under 2 years old (0-23 completed months at the time of the interview), or the main caregivers and decision-makers in the household if the mothers and fathers are not available. We will conduct interviews before and after the intervention so we can compare differences in nutrition, health and agricultural outcomes over time.

We may also conduct further in-depth interviews whilst the intervention is ongoing, to collect more detail on diet and food habits, social networks, household expenditures or agricultural practices, and/or to monitor the progress and quality of the interventions.

If the respondents give us permission, we will ask them questions about their health, nutrition, hygiene and sanitation practices, agriculture and household consumption. We will also measure weight, height and mid-upper arm circumference of mothers and children under 2 years, and we will use a sterilized needle to collect a drop of blood by pricking the end of their finger (for mothers and children). The blood will be tested using a haemocue machine to assess haemoglobin/iron in the blood and they will know if the mother or child has anaemia. It is not so painful and will not cause any harm.

We may also ask some respondents about their experiences of the interventions.

HOW MANY PEOPLE AND VILLAGES WILL PARTICIPATE?

Village clusters are defined as village and surrounding hamlets, and in some cases two villages have been merged together to ensure that the clusters have approximately equal population size.

We are inviting 148 village clusters in Keonjhar to participate in the study, and these village clusters will be randomly allocated to one of these four arms (the three different intervention plus the comparison 'control' area that only has existing programs). This means that there will be 37 villages allocated to each arm.

We will also interview approximately 32 households per village cluster (households with children under 2 years of age), so approximately 4,736 households in Keonjhar will respond to the interviews.

HOW WILL THE VILLAGES BE ALLOCATED TO THE STUDY ARMS?

The allocation of village clusters to one of the 4 study arms will be conducted by a researcher in London. The researcher will use computer software to ensure that the allocation is completely random. In other words, the allocation is completely by chance and there is nothing that can influence this allocation decision.

CAN THE ALLOCATION OF VILLAGES CHANGE?

After this random, allocation, nothing can affect how the village clusters are allocated. The allocation will be fixed until the end of the study in December 2019.

HOW LONG THIS STUDY LAST?

The intervention is planned to run for 3 years, from January 2017 to December 2019.

WHAT ARE THE RISKS OF THIS STUDY?

There are no known risks associated with this research other than the potential for mild boredom or fatigue from the interviews.

An external review board will monitor the progress of this project. If we identify any risks posed by the intervention, we will report this to the review board and they will determine if we have any need to end the study before the intended end date.

WHAT ARE THE BENEFITS OF THIS STUDY?

We do not know what the benefits to villages participating in the study are, or if there will be any benefit at all. This is why we are testing the interventions. We are conducting this study to find out if the interventions will improve agricultural practices and nutrition of children and mothers, so it is possible that these outcomes will improve.

In the interviews, we will refer severely undernourished women and children to the nearest health facility, so this will be some small benefit for all study arms.

The participation of this village may lead to future benefits for families in Keonjhar. If the interventions prove to be successful, we will try to secure additional funding to deliver one of the interventions after the 36-month study period. In this case, priority would be given to the villages that did not receive any additional intervention during the study. However, we cannot be certain if this will happen or not.

Participation will be highly appreciated, and the results that we obtain from this study will be used for planning agriculture and nutrition related programs and services.

WHAT OTHER OPTIONS ARE THERE?

The alternative is to not participate in the study. Participation of the village cluster in the study is completely voluntary.

However, we cannot change the plans for these interventions or the random allocation of villages to the study arms.

There will be no risk as a result of the village / surrounding areas participating in the study, or if the village cluster refuses to participate in the study.

WILL IT COST ME OR THE COMMUNITY MEMBERS ANYTHING TO BE IN THIS STUDY?

There is no cost to anyone in the village cluster for participating in this study.

WILL I (OR THE COMMUNITY) BE PAID FOR PARTICIPATION?

Neither you nor the community will be paid for being in this research study.

WHO IS FUNDING THIS STUDY?

The Bill & Melinda Gates Foundation have provided funds for this study.

FUTURE STUDY PARTICIPATION

There is the possibility that we will conduct more studies in Keonjhar in the future. Please tell us if you would like to be contacted again about participating in other projects.

_____Yes _____No

HOW WILL YOU KEEP THE STUDY INFORMATION CONFIDENTIAL?

We are unable to hide whether or not the village cluster has participated or which study arm the village has been allocated to. We will keep individual respondents' participation in interviews confidential to the extent permitted by law.

However, it is possible that other people such as those indicated below, may become aware of the village's participation in this study and may inspect and copy records pertaining to this research. Some of these records could contain information that personally identifies you [village head] and this village.

- Federal government regulatory agencies,
- University representatives, to complete University responsibilities
- London School of Hygiene & Tropical Medicine's Institutional Review Board (a committee that reviews and approves research studies)

Any information we obtain during the study will be used only for the research. To help protect your confidentiality, we will keep the data file of this consent form with your name in a locked, secure location in Bhubaneshwar. All other files will have only codes that are not identifiable to anyone but the PI and the research team. If we write a report or article about this study or share the study data set with others, we will do so in such a way that you cannot be directly identified.

We will disclose, to the proper authority, information you share with us concerning child abuse, child sexual abuse, or harming yourself or others.

IS BEING IN THIS STUDY VOLUNTARY?

The village's participation in this research study is completely voluntary. The village may choose not to take part at all. If you decide not to be in this study, or if you stop participating at any time, you won't be penalized or lose any benefits for which you otherwise qualify.

What if we decide to withdraw from the study?

Villages may withdraw by telling the study team the village is no longer interested in participating in the study. We would still use any research information collected before consent was withdrawn, unless you specifically direct us not to.

If the village decides to leave the study early, we will ask you to directly inform the study coordinator (to be recruited) by phone or in person. You would not be obligated to make a visit to the study coordinator, but we may ask you the reasons for this decision.

Will we receive new information about the study while participating?

If we obtain any new information during this study that might affect your willingness to continue participating in the study, we'll promptly provide you with that information.

Can someone else end our participation in this study?

Under certain circumstances, the researchers might decide to end the village's participation in this research study earlier than planned. This might happen, for example, in cases of extreme weather events (e.g. flooding or earthquakes) or due to severe political unrest that threatens the security of the program implementers.

WHAT IF I HAVE QUESTIONS?

We encourage you to ask questions. If you have any questions about the research study itself, please contact: the study coordinator, [PHONE]. If you feel that you have been harmed in any way by your participation in this study, please contact: the study coordinator [PHONE].

If you have questions, concerns, or complaints about your rights as a research participant please contact X.

This consent form is not a contract. It is a written explanation of what will happen during the study if you decide to participate. You are not waiving any legal rights by agreeing to participate in this study.

Your signature or thumbprint indicates that this research study has been explained to you, that your questions have been answered, and that you agree for your village to take part in this study. You will receive a signed copy of this form.
