# Supplementary material for: Upscaling Participatory Action and Videos for Agriculture and Nutrition (UPAVAN) trial comparing three variants of a nutrition-sensitive agricultural extension intervention to improve maternal and child nutritional outcomes in rural Odisha, India: study protocol for a cluster randomised controlled trial
Source: Trials. 2018 Mar 9;19:176. doi: 10.1186/s13063-018-2521-y (PMC5845188; doi:10.1186/s13063-018-2521-y)
Supplement: Supplementary file 4 — Consent forms. (ZIP 1740 kb) [file 13063_2018_2521_MOESM4_ESM.zip › AF4_PIS_IRB.pdf]

LONDON SCHOOL of HYGIENE & TROPICAL MEDICINE

PARTICIPANT INFORMATION SHEET

Invitation and Summary

We'd like to invite you to take part in our research study, UPAVAN: Upscaling Participation and Videos for Agriculture and Nutrition. Joining the study is entirely up to you, before you decide we would like you to understand why the research is being done and what it would involve for you. A member of our team will go through this information sheet with you, to help you decide whether or not you would like to take part and answer any questions you may have. The first part of the Participant Information Sheet tells you the purpose of the study and what will happen to you if you take part. Then we give you more detailed information about the conduct of the study. We'd suggest this should take about 10 minutes.

- If you have any questions about anything in this form, you should ask the research team for more information.
- You may also wish to talk to your family or friends about your participation in this study.
- Do not agree to participate in this study unless the research team has answered your questions and you decide that you want to be part of this study.

By signing this form, you are agreeing to participate in this study.

WHAT IS THE PURPOSE OF THIS STUDY?

This is a research study. The purpose of this study is to understand whether and how new interventions to improve agriculture practices in Keonjhar can result in improved health and nutrition for women and children. If we can improve health and nutrition for women and children here, the same approach may also help women and children in other regions.

A lot of work has been done to understand whether agricultural programs improve nutrition outcomes. What we know is that improvements in nutrition are more likely when agriculture interventions target women and include activities to boost their empowerment in agriculture and the health-nutrition. We don't know enough about what exactly works to improve maternal and child nutrition - or how - and we don't know enough about how to design innovative approaches to stimulate agriculture's contribution to nutrition.

As a mother of a child under two years of age living in this village,you and your householdhave been chosen by a random selection process to be invited to participate in the study.

WHAT WILL HAPPEN DURING THIS STUDY?

You will be invited to participate in a woman's group assigned to one of three conditions. For the next four years, women's groups in your village will either:

1) Receive standard agriculture (e.g. subsidies and extension), health (e.g. Vitamin A supplementation; immunizations) and nutrition related services (e.g. iron and folic acid supplementation; supplementary feeding through the Integrated Child Development Services [ICDS]) provided by the Government (or other organizations) in this area.

2) Receive an agriculture extension intervention from VARRAT and Digital Green. Women's groups will meet at least once a month -at a date, time and venue decided by the group - to screen locally produced videos addressing agriculture topics prioritized by the community, and share updates on credit and savings activities. At the screening, the VARRAT frontline workerwillpauses

the video at strategic points, to facilitate a discussion about what you see in the video. A few days after the screening, the VARRAT frontline work may visit you at your home to verify whether you have tried the recommended practice. Or

3) Receive the Digital Green agriculture extension intervention, plus videos on maternal, infant and young child nutrition with participatory learning activities focused on maternal and child nutrition.

Your participation will involve answering questions related to your household's health, wellbeing, and agriculture practices. During this study, the interviewer will speak with the mother and/or primary caregiver, and one male member of your household. The interviewer will ask you questions related to diverse topics including your and your child's health and nutrition; your household's economic and food security; water, sanitation, and hygiene practices; infant and young child feeding practices; empowerment; your child's diet; and your social network. We will also measure your height and weight and that of your child aged 0-23 months selected randomly as the index child. We will use a sterilized needle to collect a drop of blood by pricking the end of your finger and the finger of your child. The blood will be tested using a hemocue machine to assess haemoglobin/iron in the blood and you will know if either of you have anemia. It is not so painful and will not cause any harm.

We are inviting you to be a participant in this study. We value your opinion and there are no wrong answers to the questions we will be asking in the interview.

The interviewer will not take pictures of you or your family. The interview will take place at your home. The amount of time involved to collect all the information will be approximately 2.5-3 hours. As a participant in this study, you will be asked to do the following:

- Participate in a 3-hour one-on-one interview with research staff.
- The interview will take place at your home.
- During the interview, we will ask you questions about your household's health, wellbeing, and agriculture practices.
- You may be asked to walk with the interviewer in order to show him/her the location various health-related features in your home such as the the latrine, hand-washing station, etc.

HOW MANY PEOPLE WILL PARTICIPATE?

Approximately 3,200 households will take part in this study conducted by investigators at London School of Hygiene & Tropical Medicine.

HOW LONG WILL I BE IN THIS STUDY?

If you agree to take part in this study, your involvement will last for just the amount of time that it takes to complete the interview.

WHAT ARE THE RISKS OF THIS STUDY?

There are no known risks associated with this research other than the potential for mild boredom or fatigue.

WHAT ARE THE BENEFITS OF THIS STUDY?

There are no known benefits to you for participating in this study aside from knowing that your participation may lead to future benefits for families in Keonjhar.Your participation will be highly appreciated. The answers you give will be used for planning agriculture and nutrition related programs and services.

WHAT OTHER OPTIONS ARE THERE?

There is no alternative to participating in this study. There will be no risk as a result of your participating in the study, or if you refuse to participate in the study. Your participation in the study is completely voluntary. You are free to withdraw your consent and discontinue participation at any time and/or to refuse to answer any question. You may also ask any questions concerning the study at any time.

WILL IT COST ME ANYTHING TO BE IN THIS STUDY?

There is no cost to you to participate in this study.

WILL I BE PAID FOR PARTICIPATING?

You will not be paid for being in this research study.

WHO IS FUNDING THIS STUDY?

The Bill & Melinda Gates Foundation have provided funds for this study.

FUTURE STUDY PARTICIPATION

There is the possibility that we will conduct more studies in Keonjhar in the future. Please tell us if you would like to be contacted again about participating in other projects. _____Yes _____No

HOW WILL YOU KEEP MY INFORMATION CONFIDENTIAL?

We will keep your participation in this research study confidential to the extent permitted by law. However, it is possible that other people such as those indicated below may become aware of your participation in this study and may inspect and copy records pertaining to this research. Some of these records could contain information that personally identifies you.

- Federal government regulatory agencies,
- University representatives, to complete University responsibilities
- London School of Hygiene & Tropical Medicine's Institutional Review Board (a committee that reviews and approves research studies)

Any information we obtain from you during the study will be used only for the research. To help protect your confidentiality, we will keep the data file with your name in a locked, secure location in Bhubaneshwar. All other files will have only codes that are not identifiable to anyone but the PI and the research team. If we write a report or article about this study or share the study data set with others, we will do so in such a way that you cannot be directly identified.

We will disclose, to the proper authority, information you share with us concerning child abuse, child sexual abuse, or harming yourself or others.

IS BEING IN THIS STUDY VOLUNTARY?

Taking part in this research study is completely voluntary. You may choose not to take part at all. If you decide to be in this study, you may stop participating at any time. If you decide not to be in this study, or if you stop participating at any time, you won't be penalized or lose any benefits for which you otherwise qualify.

What if I decide to withdraw from the study?

You may withdraw by telling the study team you are no longer interested in participating in the study.

If you decide to leave the study early, we will ask you to directly inform the study coordinator (to be recruited) by phone or in person. You would not be obligated to make a visit to the study coordinator, but we may ask you the reasons for your decision.

Will I receive new information about the study while participating?

If we obtain any new information during this study that might affect your willingness to continue participating in the study, we'll promptly provide you with that information.

Can someone else end my participation in this study?

Under certain circumstances, the researchers might decide to end your participation in this research study earlier than planned. This might happen because your child becomes very ill or if you move away from Keonjhar.

WHAT IF I HAVE QUESTIONS?

We encourage you to ask questions. If you have any questions about the research study itself, please contact: the study coordinator, [PHONE]. If you feel that you have been harmed in any way by your participation in this study, please contact: the study coordinator [PHONE].

If you have questions, concerns, or complaints about your rights as a research participant please contact X.

This consent form is not a contract. It is a written explanation of what will happen during the study if you decide to participate. You are not waiving any legal rights by agreeing to participate in this study.

Your signature indicates that this research study has been explained to you, that your questions have been answered, and that you agree to take part in this study. You will receive a signed copy of this form.
